# Dearomative Ring Expansion of Polycyclic Arenes

**DOI:** 10.1002/anie.202208014

**Published:** 2022-07-26

**Authors:** Paolo Piacentini, Tanner W. Bingham, David Sarlah

**Affiliations:** ^1^ Department of Chemistry University of Pavia Viale Taramelli 12 27100 Pavia Italy; ^2^ Department of Chemistry University of Illinois Urbana IL 61801 USA

**Keywords:** Arenophiles, Benzocycloheptatrienes, Buchner Reaction, Dearomatization, Ring Expansion

## Abstract

Benzocycloheptenes constitute a common structural motif embedded in many natural products and biologically active compounds. Herein, we report their concise preparation from non‐activated polycyclic arenes using a two‐step sequence involving dearomative [4+2]‐cycloaddition with arenophile in combination with palladium‐catalyzed cyclopropanation, followed by cycloreversion‐initiated ring expansion. The described strategy provides a working alternative to the Buchner reaction, which is limited to monocyclic arenes. Overall, this methylene‐insertion molecular editing approach enables rapid and direct conversion of simple (hetero)arenes into a range of substituted (aza)benzocycloheptatrienes, which can undergo a myriad of downstream functionalizations.

## Graphical abstract

A missing link between dearomative cyclopropanation and ring expansion of polycyclic (hetero)arenes has been established by utilizing an arenophile‐based strategy. This two‐step protocol enables the direct conversion of aromatic compounds into (aza)benzocycloheptatrienes, which are important structural motifs in natural products and biologically active compounds.

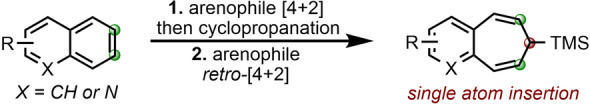

Seven‐membered carbocycles occupy a unique chemical space and are valuable intermediates in molecular sciences, including medicinal and materials chemistry. Particularly, compounds encompassing benzo‐fused unsaturated seven‐membered rings are often featured as bioactive natural products and important drug leads (Scheme 1a). For example, colchicine (**1**)^[1]^ is a well‐known natural product used to treat gout as well as familial Mediterranean fever;^[2]^ and purpurogallin (**2**)^[3]^ is a central motif to the benzotropolone class of antioxidants possessing multiple bioactivities.^[4]^ Furthermore, this scaffold is commonly used in medicinal chemistry, as exemplified with amcenestrant (**3**),^[5]^ an investigational oral selective estrogen receptor degrader (SERD), currently evaluated with hormone receptor‐positive advanced breast cancer patients. Due to their importance, several strategies have been developed to access benzocycloheptenes,^[6]^ ranging from annulation reactions to ring expansions of tetralone and dihydronaphthalene derivatives. Since these approaches require multistep sequences and tailored starting materials, their general use has been limited to only a few specific examples.

**Scheme 1:**
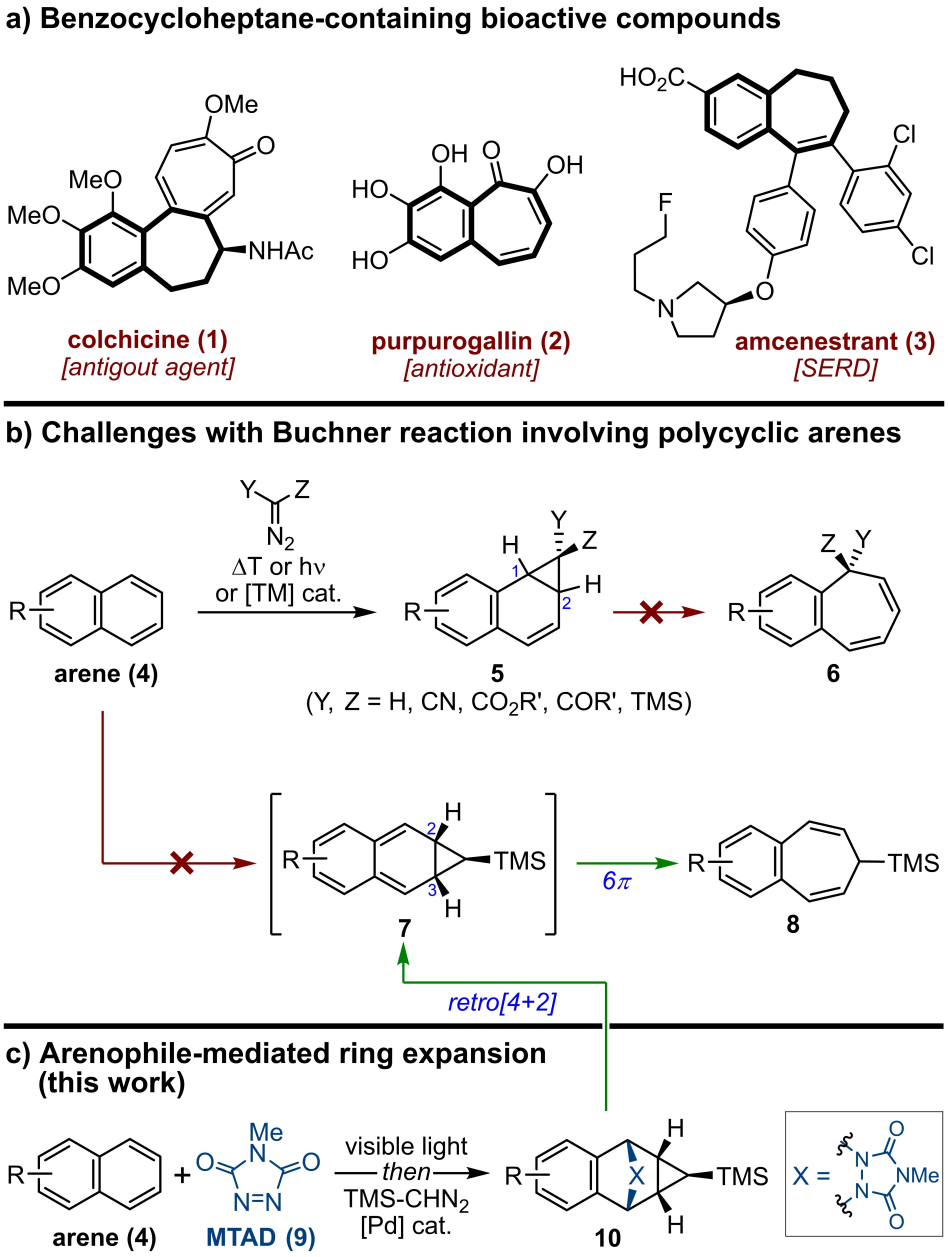
a) Selected examples of benzocycloheptane‐containing bioactive natural products and drug leads. b) Challenges associated with Buchner ring expansion of polycyclic arenes. c) This work: dearomative arenophile‐based ring expansion of polycyclic arenes. TMS=trimethylsilane, SERD=selective estrogen receptor degrader, MTAD=4‐methyl‐1,2,4‐triazoline‐3,5‐dione.

On the other hand, the dearomative ring expansion of readily available polycyclic arenes could provide more direct access to such structural motifs; albeit, no such strategy exists to date. The venerable Buchner ring expansion,^[7]^ involving carbene addition/dearomative cyclopropanation followed by 6π electrocyclic opening of norcaradienes to cycloheptatrienes has been synthetically very useful;^[8]^ however this strategy works efficiently only for benzene derivatives.^[9]^ The main limitation in translating this approach to polycyclic arenes is their inherent reactivity, which guides cyclopropanation to the 1,2‐position (**4**→**5**, Scheme 1b).^[10]^ Due to geometrical constraints and loss of aromaticity during the valence bond tautomerization,^[11]^ the corresponding 1,2‐benzonorcaradienes **5** cannot readily undergo further ring expansion to benzofused cycloheptatrienes **6**.^[12]^ To address this gap, we hypothesized that if cyclopropanation could be diverted to the C2−C3 position (i.e., **4**→**7**), such intermediate would rapidly undergo electrocyclic ring expansion (**7**→**8**), driven by concurrent rearomatization. Herein, we showcase that 2,3‐chemoselective cyclopropanation is feasible through the intermediacy of arenophile‐based chemistry (Scheme 1c).^[13]^ Specifically, visible‐light‐mediated cycloaddition of polycyclic arenes with arenophile 4‐methyl‐1,2,4‐triazoline‐3,5‐dione (MTAD, **9**), followed by in situ Pd‐catalyzed cyclopropanation with TMS‐diazomethane, provided bench‐stable polycycles **10**, which serve as synthons to otherwise inaccessible intermediates of type **7**.

Our investigation into the synthesis of benzocycloheptatrienes began with the in situ cyclopropanation of the cycloadduct, formed between MTAD (**9**, 1.0 equiv) and naphthalene (**4 a**, 2.0 equiv), as shown in Table 1. We initially explored a variety of well‐established strategies for cyclopropanation, including the Simmons–Smith^[14]^ reaction and Rh‐catalyzed cyclopropanation with α‐diazoesters;^[15]^ however, unfortunately, neither of these reactions led to the formation of the product (see the Supporting Information for full details). Gratifyingly, we found that palladium‐catalyzed cyclopropanation^[16]^ with freshly prepared diazomethane gave the desired product **10 a′** in 16 % yield (Entry 1). Moreover, the use of a less dangerous and commercially available TMS‐CHN_2_ also proved viable (5 % yield of **10 a**, Entry 2,) and led to higher conversions by conducting the cyclopropanation at −50 °C with slow warming to room temperature over 18 h (28 %, Entry 3). Changing the solvent to ethyl acetate provided additional improvements, delivering the product in 50 % yield (Entry 4). Further optimization, involving screening of different palladium sources (see the Supporting Information for full details), revealed that Pd(dba)_2_ and Pd_2_(dba)_3_⋅CHCl_3_ were particularly effective, furnishing cyclopropanation product **10 a** in 65 % and 79 % yield (Entries 5 and 6). Interestingly, keeping the reaction constantly at a lower temperature (−50 °C, Entry 7) or reducing equivalents of the diazo reagent (Entry 8) resulted in significantly diminished yields.

**Table 1:** Optimization of the reaction conditions for dearomative cycloaddition/Pd‐catalyzed cyclopropanation.^[a]^

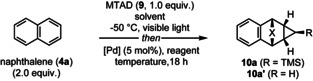					
Entry	[Pd]	Reagent (equiv.)	Solvent	*T* [°C]	Yield [%]^[b]^
1	Pd(OAc)_2_	CH_2_N_2_ (3)	CH_2_Cl_2_	−78→25	16
2	Pd(OAc)_2_	TMSCHN_2_ (3)	CH_2_Cl_2_	−78→25	5
3	Pd(OAc)_2_	TMSCHN_2_ (3)	CH_2_Cl_2_	−50→25	28
4	Pd(OAc)_2_	TMSCHN_2_ (3)	EtOAc	−50→25	50
5	Pd(dba)_2_	TMSCHN_2_ (3)	EtOAc	−50→25	65
6	Pd_2_(dba)_3_	TMSCHN_2_ (3)	EtOAc	−50→25	79
7	Pd_2_(dba)_3_	TMSCHN_2_ (3)	EtOAc	−50	5
8	Pd_2_(dba)_3_	TMSCHN_2_ (1.5)	EtOAc	−50→25	35

[a] Standard reaction conditions: MTAD (**9**, 1.0 mmol, 1.0 equiv), naphthalene (**4 a**, 2.0 mmol, 2.0 equiv), solvent (0.2 M), visible light, −50 °C, 12 h; then addition of a solution of catalyst, diazo reagent, temperature, 18 h. Pd_2_(dba)_3_ was used as the Pd_2_(dba)_3_⋅CHCl_3_ adduct. [b] Isolated yield of **10 a′** (Entry 1) and **10 a** (Entries 2–8) after purification by flash chromatography. dba=dibenzylideneacetone.

Having identified optimal conditions for the cyclopropanation, we turned our attention towards exploring the scope and subsequent retrocycloaddition step to reveal the desired benzocycloheptatrienes (Table 2). The cycloreversion proceeds smoothly by exposing polycyclic intermediates **10** to KOH in *i*‐PrOH at 40 °C, followed by copper‐catalyzed oxidation under an oxygen atmosphere.^[17]^ During this sequence, urazole undergoes partial hydrolysis/decarboxylation and the resulting cyclic semicarbazide intermediate is subjected to further oxidation,^[18]^ resulting in extrusion of dinitrogen and ring expansion. Utilizing this protocol, the naphthalene‐derived cyclopropanated intermediate gave the product **8 a** in 75 % yield. To explore the compatibility of this chemistry with different functionality, several 1‐ and 2‐substituted naphthalene derivatives were tested, furnishing functionalized 3,4‐benzocycloheptatriene analogs. We found that a variety of substituents were tolerated, such as nitrile (**8 b** and **8 c**), chlorine and fluorine (**8 d**–**8 f**), trifluoromethyl (**8 g**), trimethylsilyl (**8 h**), nitro (**8 i**), and phenyl group (**8 j**). Furthermore, ketones and aldehydes (**8 k**–**8 n**), initially protected as the corresponding acetals during the cycloaddition step, worked as well, and were deprotected during the acidic workup. In all cases, the Pd‐catalyzed cyclopropanation step delivered polycyclic intermediates **10** as a single diastereoisomer (see Table 2 for X‐ray structure of **10 a**),^[19]^ which readily underwent spontaneous cycloreversion to expanded products **8**. Finally, we tested the scalability of this two‐step sequence on a gram scale using naphthalene (**4 a**), which delivered products with comparable yields.

**Table 2:**
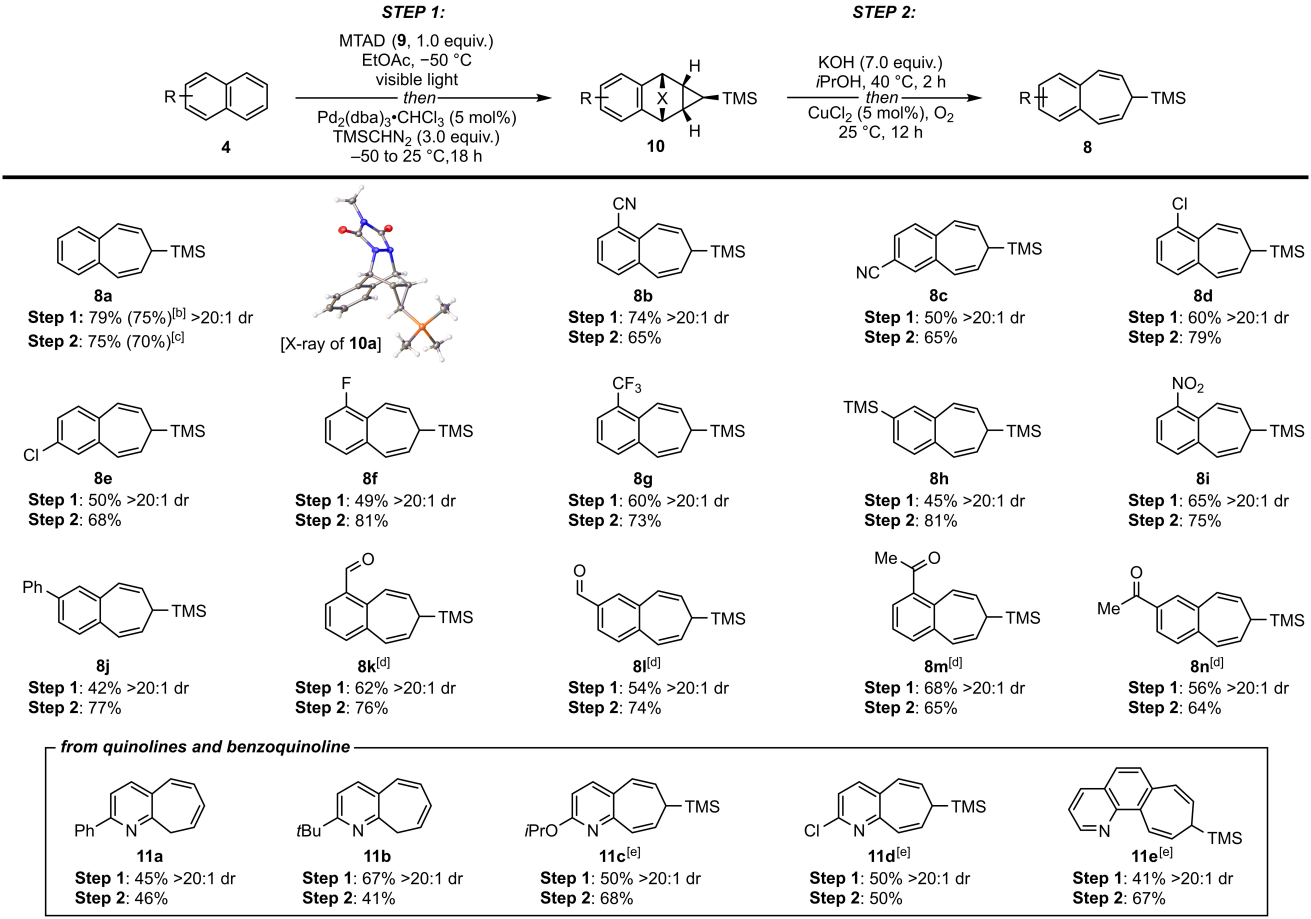
Substrate scope of dearomative ring expansion of polycyclic (hetero)arenes.^[a]^

[a] Standard reaction conditions: ***STEP 1***: MTAD (**9**, 1.0 mmol, 1.0 equiv), arene (**4**, 2.0 mmol, 2.0 equiv), EtOAc (0.2 M), visible light, −50 °C, 12 h; then addition of a solution of Pd_2_(dba)_3_⋅CHCl_3_ (5 mol %), TMSCHN_2_ (3.0 equiv), −50 to 25 °C, 18 h. ***STEP 2***: substrate **10** (0.5 mmol, 1.0 equiv), KOH (5.0 equiv) *i*‐PrOH (0.1 M), 40 °C, 2 h; then AcOH (until pH 5), CuCl_2_ (5 mol %), O_2_ (1 atm), 25 °C, 12 h. Reported yields are of isolated products and the ratio of diastereoisomers was determined by ^1^H NMR of the crude reaction mixtures. [b] Reaction conducted on 1 g (8.8 mmol) scale. [c] Reaction conducted on 1 g (3.0 mmol) scale. [d] Substrate with protected carbonyl (dimethoxyacetal) was used. [e] Ni_2_O_3_ (3.0 equiv) was used instead of CuCl_2_/O_2_.

To further broaden the scope of the dearomative ring expansion, we tested polycyclic heteroarenes, such as quinolines and their derivatives. In addition to probing their compatibility, we were driven by the fact that the resulting azabenzocycloheptatrienes are largely unexplored, and not an easy class of heterocycles to access.^[20]^ Therefore this strategy could provide a direct pathway for their single‐atom skeletal editing.^[21]^ Gratifyingly, optimized conditions translated well to quinolines, as showcased with analogs encompassing phenyl (**11 a**), *tert*‐butyl (**11 b**), alkoxy (**11 c**), and chloro (**11 d**) substituents.^[22]^ Also, benzo[*h*]quinoline proved a viable substrate, delivering ring‐expanded product **11 e**. Notably, during the Cu‐catalyzed cycloreversion step, a complete protodesilylation and olefin isomerization occurred in the case of **11 a** and **11 b**, furnishing products as 9*H*‐cyclohepta[*b*]pyridine derivatives. On the other hand, the use of Ni_2_O_3_ was required to secure products **11 c**–**11 e**, as inseparable mixtures of silylated and protodesilylated products were observed under Cu‐mediated oxidation. Importantly, no nitrogen oxidation at the heterocyclic nucleus was observed under these conditions.

Silyl‐substituted benzocycloheptatrienes of type **8** are not known, therefore presenting a unique opportunity for exploration of their reactivity and mapping downstream functionalization options (Scheme 2). Thus, experiments revealed that the exposure of **8 a** to BF_3_⋅OEt_2_ resulted in olefin isomerization (**12**), and standard hydrogenation could completely reduce the diene motif (**13**). Moreover, oxidation with chromium(VI) oxide/pyridine^[23]^ resulted in 2,3‐benzotropone derivative **14**. On the other side, **8 a** could readily undergo cycloaddition chemistry, as demonstrated with nitrile oxide [3+2] dipolar cycloaddition,^[24]^ which gave diastereomeric mixture of isoxazoline **15 a** and **15 b**. Also, a diastereoselective Upjohn dihydroxylation^[25]^ provided diol **16** as a single diastereoisomer, which could undergo desilylation upon treatment with KH (**16**→**17**).^[26]^ Similarly, the direct TMS‐group removal proved possible with compound **8 a**,^[27]^ albeit concurrent olefin isomerization to 1,2‐benzotropilidene **18** was observed under required conditions.^[28]^ Also, this diene substrate can undergo oxidation with chromium(VI) oxide/pyridine, providing 2,3‐benzotropone (**19**) and 4,5‐benzotropone (**20**). Finally, in order to secure unsubstituted 3,4‐benzotropilidene (**21**), the desilylation was executed concurrently with the cycloreversion step on intermediate **10 a** (Scheme 2, inset), constituting the first direct and practical CH_2_ insertion strategy into the 2,3‐position of the polycyclic arenes.

**Scheme 2:**
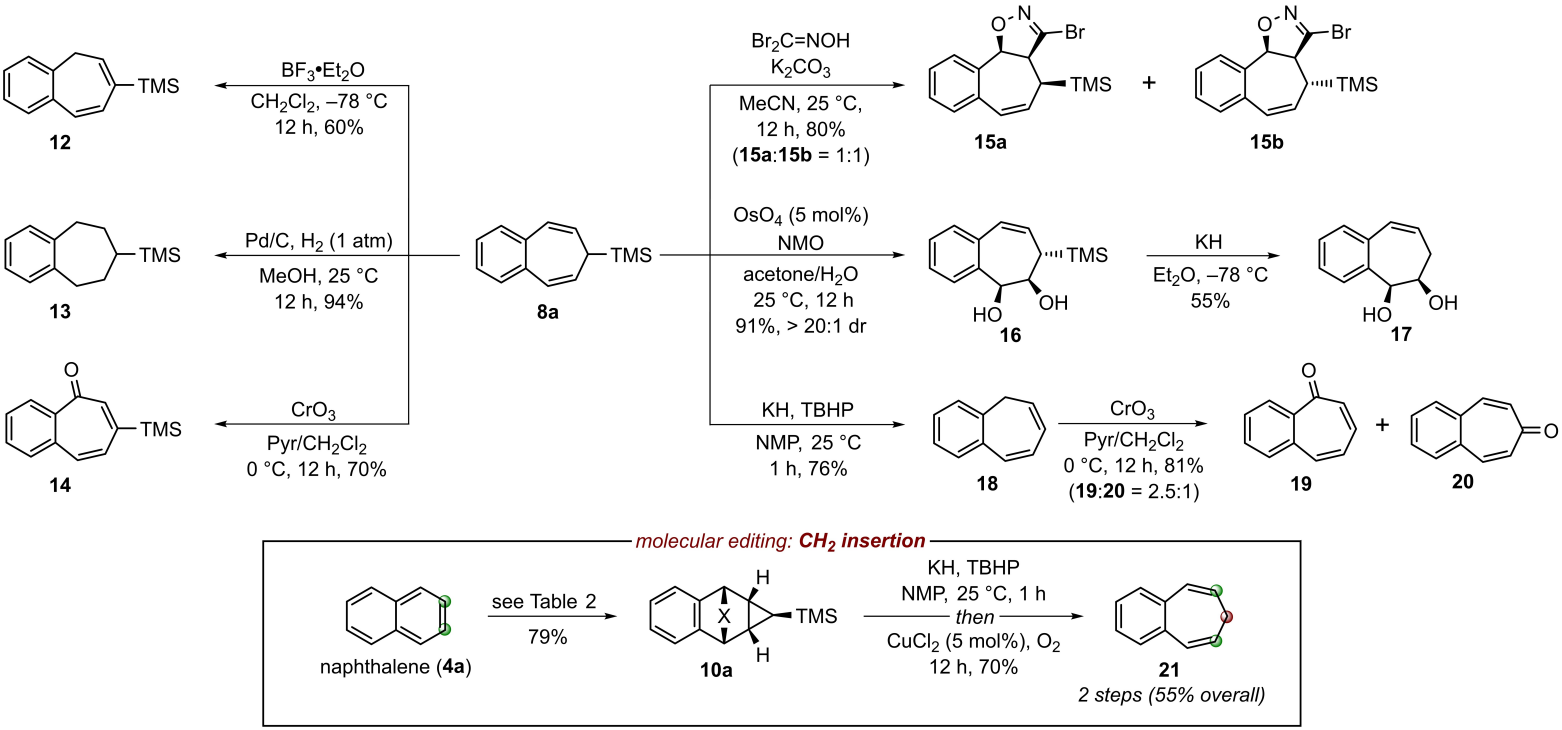
Diversification of product **8 a** and two‐step methylene 2,3‐insertion into naphthalene (**4 a**→**21**). NMO=4‐methylmorpholine *N*‐oxide, TBHP=*tert*‐butyl hydroperoxide, Pyr=pyridine, NMP=*N*‐methyl‐2‐pyrrolidone.

In summary, a dearomative ring‐expansion of simple and nonactivated polycyclic arenes and heteroarenes was developed involving an arenophile‐based cycloaddition and palladium‐catalyzed cyclopropanation strategy. This approach resulted in cyclopropanation and ring‐expansion on 2,3‐site of arenes, enabling Buchner‐like reaction to occur, providing direct access to (aza)benzocycloheptatrienes that are amenable to a host of functionalization reactions, including the preparation of benzotropones and other elaborated benzocycloheptanes. Given the common occurrence of these structural motifs in secondary metabolites and biologically active compounds, as well as the lack of synthetic approaches for their rapid preparation, this method is expected to find application in medicinal and natural products chemistry.

## Conflict of interest

The authors declare no conflict of interest.

## Supporting information

As a service to our authors and readers, this journal provides supporting information supplied by the authors. Such materials are peer reviewed and may be re‐organized for online delivery, but are not copy‐edited or typeset. Technical support issues arising from supporting information (other than missing files) should be addressed to the authors.

Supporting Information

Supporting Information

Supporting Information

Supporting Information

Supporting Information

## Data Availability

The data that support the findings of this study are available in the Supporting Information of this article.
