# Supplementary material for: Dearomative Ring Expansion of Polycyclic Arenes
Source: Angew Chem Int Ed Engl. 2022 Jul 26;61(36):e202208014. doi: 10.1002/anie.202208014 (PMC9543877; doi:10.1002/anie.202208014)

checkCIF/PLATON report

Structure factors have been supplied for datablock(s) I

THIS REPORT IS FOR GUIDANCE ONLY. IF USED AS PART OF A REVIEW PROCEDURE FOR PUBLICATION, IT SHOULD NOT REPLACE THE EXPERTISE OF AN EXPERIENCED CRYSTALLOGRAPHIC REFEREE.

No syntax errors found. CIF dictionary Interpreting this report

Datablock: I

Bond precision:	C-C = 0.0016 A		Wavelength=0.71073
Cell:	a=26.2536(4)	b=11.6384(2)	c=29.4236(4)
	alpha=90	beta=107.0100	gamma=90
Temperature:	100 K		

	Calculated	Reported
Volume	8597.1(2)	8596.9(2)
Space group	P 21/c	P 1 21/c 1
Hall group	-P 2ybc	-P 2ybc
Moiety formula	C17 H21 N3 O2 Si	C17 H21 N3 O2 Si
Sum formula	C17 H21 N3 O2 Si	C17 H21 N3 O2 Si
Mr	327.46	327.46
Dx,g cm-3	1.265	1.265
Z	20	20
Mu (mm-1)	0.149	0.149
F000	3480.0	3480.0
F000'	3482.86	
h,k,lmax	35,15,39	35,15,39
Nref	21370	21341
Tmin,Tmax	0.918,0.946	0.717,0.746
Tmin'	0.910	

Correction method= # Reported T Limits: Tmin=0.717 Tmax=0.746
AbsCorr = MULTI-SCAN

Data completeness= 0.999 Theta(max)= 28.298

R(reflections)= 0.0343(18466) wR2(reflections)= 0.0940(21341)

S = 1.024 Npar= 1056

The following ALERTS were generated. Each ALERT has the format
test-name_ALERT_alert-type_alert-level.
Click on the hyperlinks for more details of the test.

Alert level C

```
PLAT150_ALERT_1_C Volume as Calculated Differs from that Given ...    8596.90 Ang-3
PLAT220_ALERT_2_C NonSolvent   Resd 5  C   Ueq(max)/Ueq(min) Range       3.7 Ratio
PLAT222_ALERT_3_C NonSolvent Resd 5  H   Uiso(max)/Uiso(min) Range       4.6 Ratio
PLAT230_ALERT_2_C Hirshfeld Test Diff for    Si1      --C13      .       5.2 s.u.
PLAT230_ALERT_2_C Hirshfeld Test Diff for    O2       --C15      .       5.3 s.u.
PLAT230_ALERT_2_C Hirshfeld Test Diff for    O2A      --C15A     .       5.3 s.u.
PLAT230_ALERT_2_C Hirshfeld Test Diff for    O2B      --C15B     .       5.7 s.u.
PLAT230_ALERT_2_C Hirshfeld Test Diff for    O2D      --C15D     .       5.5 s.u.
PLAT242_ALERT_2_C Low    'MainMol' Ueq as Compared to Neighbors of      Si1D Check
PLAT910_ALERT_3_C Missing # of FCF Reflection(s) Below Theta(Min).         9 Note
PLAT911_ALERT_3_C Missing FCF Refl Between Thmin & STh/L=    0.600        11 Report
PLAT913_ALERT_3_C Missing # of Very Strong Reflections in FCF ....         7 Note
```

Alert level G

```
PLAT003_ALERT_2_G Number of Uiso or Uij Restrained non-H Atoms ...         3 Report
PLAT063_ALERT_4_G Crystal Size Possibly too Large for Beam Size ..      0.63 mm
PLAT145_ALERT_4_G s.u. on beta     Small or Missing .............    0.0000 Degree
PLAT186_ALERT_4_G The CIF-Embedded .res File Contains ISOR Records         1 Report
PLAT793_ALERT_4_G Model has Chirality at C1          (Centro SPGR)         R Verify
PLAT793_ALERT_4_G Model has Chirality at C1A         (Centro SPGR)         S Verify
PLAT793_ALERT_4_G Model has Chirality at C1B         (Centro SPGR)         S Verify
PLAT793_ALERT_4_G Model has Chirality at C1C         (Centro SPGR)         S Verify
PLAT793_ALERT_4_G Model has Chirality at C1D         (Centro SPGR)         S Verify
PLAT793_ALERT_4_G Model has Chirality at C2          (Centro SPGR)         S Verify
PLAT793_ALERT_4_G Model has Chirality at C2A         (Centro SPGR)         R Verify
PLAT793_ALERT_4_G Model has Chirality at C2B         (Centro SPGR)         R Verify
PLAT793_ALERT_4_G Model has Chirality at C2C         (Centro SPGR)         R Verify
PLAT793_ALERT_4_G Model has Chirality at C2D         (Centro SPGR)         R Verify
PLAT793_ALERT_4_G Model has Chirality at C3          (Centro SPGR)         R Verify
PLAT793_ALERT_4_G Model has Chirality at C3A         (Centro SPGR)         S Verify
PLAT793_ALERT_4_G Model has Chirality at C3B         (Centro SPGR)         S Verify
PLAT793_ALERT_4_G Model has Chirality at C3C         (Centro SPGR)         S Verify
PLAT793_ALERT_4_G Model has Chirality at C3D         (Centro SPGR)         S Verify
PLAT793_ALERT_4_G Model has Chirality at C4          (Centro SPGR)         S Verify
PLAT793_ALERT_4_G Model has Chirality at C4A         (Centro SPGR)         R Verify
PLAT793_ALERT_4_G Model has Chirality at C4B         (Centro SPGR)         R Verify
PLAT793_ALERT_4_G Model has Chirality at C4C         (Centro SPGR)         R Verify
PLAT793_ALERT_4_G Model has Chirality at C4D         (Centro SPGR)         R Verify
PLAT860_ALERT_3_G Number of Least-Squares Restraints .............        18 Note
PLAT912_ALERT_4_G Missing # of FCF Reflections Above STh/L=  0.600         8 Note
PLAT933_ALERT_2_G Number of HKL-OMIT Records in Embedded .res File         3 Note
PLAT978_ALERT_2_G Number C-C Bonds with Positive Residual Density.        16 Info
```

0 **ALERT level A** = Most likely a serious problem - resolve or explain
0 **ALERT level B** = A potentially serious problem, consider carefully
12 **ALERT level C** = Check. Ensure it is not caused by an omission or oversight
28 **ALERT level G** = General information/check it is not something unexpected

```
 1 ALERT type 1 CIF construction/syntax error, inconsistent or missing data
10 ALERT type 2 Indicator that the structure model may be wrong or deficient
 5 ALERT type 3 Indicator that the structure quality may be low
24 ALERT type 4 Improvement, methodology, query or suggestion
 0 ALERT type 5 Informative message, check
```

checkCIF publication errors

Alert level A

```
PUBL004_ALERT_1_A  The contact author's name and address are missing,
            _publ_contact_author_name and _publ_contact_author_address.
PUBL005_ALERT_1_A  _publ_contact_author_email, _publ_contact_author_fax and
            _publ_contact_author_phone are all missing.
            At least one of these should be present.
PUBL006_ALERT_1_A  _publ_requested_journal is missing
            e.g. 'Acta Crystallographica Section C'
PUBL008_ALERT_1_A  _publ_section_title is missing. Title of paper.
PUBL009_ALERT_1_A  _publ_author_name is missing. List of author(s) name(s).
PUBL010_ALERT_1_A  _publ_author_address is missing. Author(s) address(es).
PUBL012_ALERT_1_A  _publ_section_abstract is missing.
            Abstract of paper in English.
```

7 **ALERT level A** = Data missing that is essential or data in wrong format
0 **ALERT level G** = General alerts. Data that may be required is missing

Publication of your CIF

You should attempt to resolve as many as possible of the alerts in all categories. Often the minor alerts point to easily fixed oversights, errors and omissions in your CIF or refinement strategy, so attention to these fine details can be worthwhile. In order to resolve some of the more serious problems it may be necessary to carry out additional measurements or structure refinements. However, the nature of your study may justify the reported deviations from journal submission requirements and the more serious of these should be commented upon in the discussion or experimental section of a paper or in the "special_details" fields of the CIF. *checkCIF* was carefully designed to identify outliers and unusual parameters, but every test has its limitations and alerts that are not important in a particular case may appear. Conversely, the absence of alerts does not guarantee there are no aspects of the results needing attention. It is up to the individual to critically assess their own results and, if necessary, seek expert advice.

If level A alerts remain, which you believe to be justified deviations, and you intend to submit this CIF for publication in a journal, you should additionally insert an explanation in your CIF using the Validation Reply Form (VRF) below. This will allow your explanation to be considered as part of the review process.

Validation response form

Please find below a validation response form (VRF) that can be filled in and pasted into your CIF.

```
# start Validation Reply Form
_vrf_PUBL004_GLOBAL
;
PROBLEM: The contact author's name and address are missing,
RESPONSE: ...
;
_vrf_PUBL005_GLOBAL
;
PROBLEM: _publ_contact_author_email, _publ_contact_author_fax and
RESPONSE: ...
;
_vrf_PUBL006_GLOBAL
;
PROBLEM: _publ_requested_journal is missing
RESPONSE: ...
;
_vrf_PUBL008_GLOBAL
;
PROBLEM: _publ_section_title is missing. Title of paper.
RESPONSE: ...
;
_vrf_PUBL009_GLOBAL
;
PROBLEM: _publ_author_name is missing. List of author(s) name(s).
RESPONSE: ...
;
_vrf_PUBL010_GLOBAL
;
PROBLEM: _publ_author_address is missing. Author(s) address(es).
RESPONSE: ...
;
_vrf_PUBL012_GLOBAL
;
PROBLEM: _publ_section_abstract is missing.
RESPONSE: ...
;
# end Validation Reply Form
```

If you wish to submit your CIF for publication in Acta Crystallographica Section C or E, you should upload your CIF via the web. If you wish to submit your CIF for publication in IUCrData you should upload your CIF via the web. If your CIF is to form part of a submission to another IUCr journal, you will be asked, either during electronic submission or by the Co-editor handling your paper, to upload your CIF via our web site.

PLATON version of 19/02/2022; check.def file version of 19/01/2022

Datablock I - ellipsoid plot

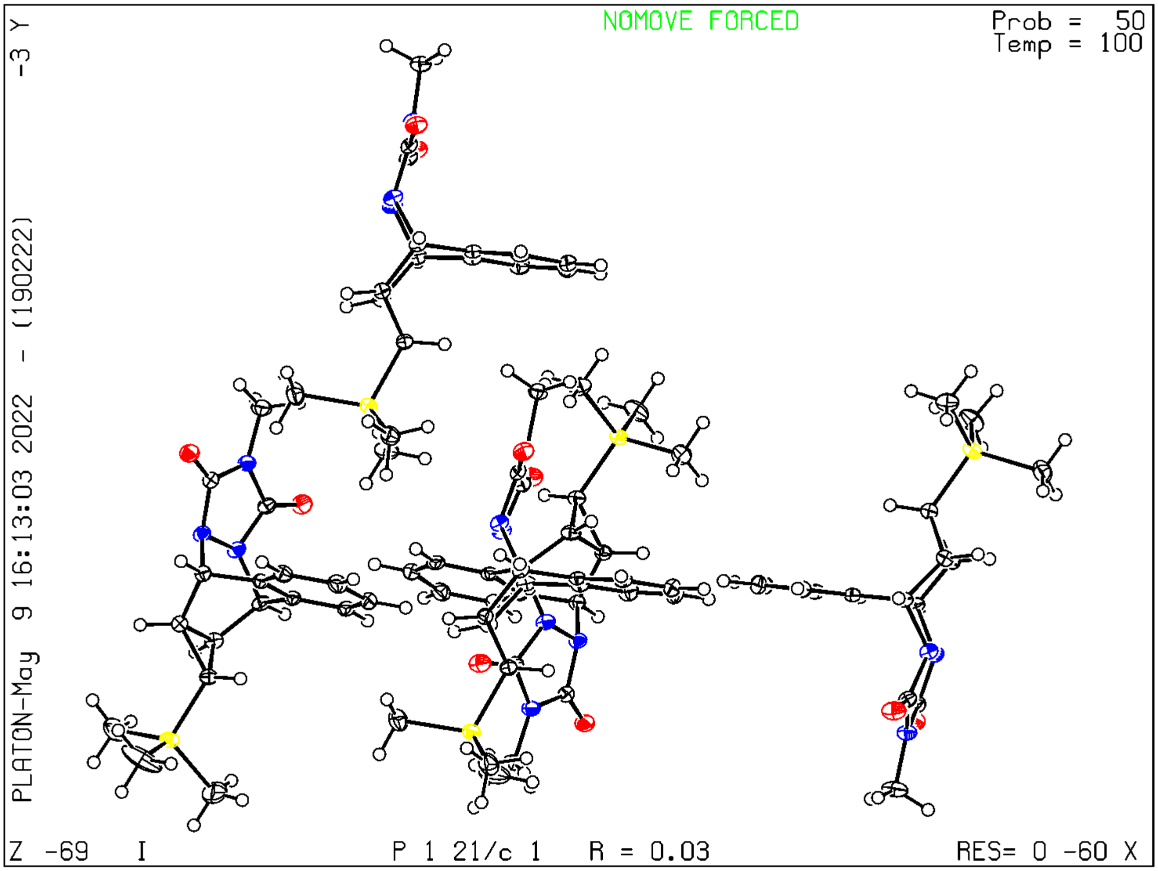

Supplement: Supplementary file 4 — Supporting Information [file ANIE-61-0-s004.pdf]
