# Supplementary material for: Dearomative Ring Expansion of Polycyclic Arenes
Source: Angew Chem Int Ed Engl. 2022 Jul 26;61(36):e202208014. doi: 10.1002/anie.202208014 (PMC9543877; doi:10.1002/anie.202208014)
Supplement: Supplementary file 5 — Supporting Information [file ANIE-61-0-s003.pdf]

Angewandte Chemie
Eine Zeitschrift der Gesellschaft Deutscher Chemiker

Supporting Information

# Dearomative Ring Expansion of Polycyclic Arenes

*P. Piacentini, T. W. Bingham, D. Sarlah**

## Table of Contents

**1. General Experimental** S2

**2. Experimental Set-up** S3

2-1. LED light source S3

2-2. Set-up for small scale reactions (up to 2.0 mmol scale) S4

2-3. Set-up for large scale reactions (up to 10 mmol scale) S4

**3. Experimental procedures** S5

3-1. Optimization of arenophile-mediated cyclopropanation of naphthalene S5

3-2. General procedure for dearomative cyclopropanation with polycyclic arenes S5

3-3. General procedure A for the synthesis of benzocycloheptatrienes S13

3-4. General procedure B for the synthesis of benzocycloheptatrienes S13

3-5. Large-scale procedures S24

**4. Crystallographic data** S25

**5. References** S27

**6. $^1$H and $^{13}$C NMR spectra** S28

## 1. General Experimental

Unless otherwise noted, all reactions were carried out under an ambient atmosphere. All chemicals were purchased from commercial suppliers and used as received. *N*-methyl-1,2,4-triazoline-3,5-dione (MTAD **12**) was prepared based on the literature procedures[1] and was resublimed before use. $Pd_2(dba)_3{\cdot}CHCl_3$ was purchased from Strem Chemicals. (Trimethylsilyl)diazomethane solution 2.0 M in hexanes was purchased from Sigma Aldrich. Nickel oxide[2] and hydroxycarbonimidic dibromide,[3] were synthesized according to literature procedures. Dry dichloromethane ($CH_2Cl_2$), ethyl acetate (EtOAc) and tetrahydrofuran (THF) were obtained by passing commercially available anhydrous, oxygen-free HPLC-grade solvents through activated alumina columns. Analytical thin-layer chromatography was performed on Merck silica gel 60 F254 aluminum plates. Visualization was accomplished with UV light and/or potassium permanganate ($KMnO_4$). Retention factor ($R_f$) values reported were measured using a 5 × 2 cm TLC plate in a developing chamber containing the solvent system described. Flash column chromatography was performed using Silicycle SiliaFlash® P60 ($SiO_2$, 40-63 µm particle size, 230-400 mesh). $^1$H and $^{13}$C NMR spectra were recorded on Bruker 500 (500 MHz, $^1$H; 126 MHz, $^{13}$C) or Varian Unity Inova 500 (500 MHz, $^1$H) spectrometers. Spectra are referenced to residual chloroform (δ = 7.26 ppm, $^1$H; 77.16 ppm, $^{13}$C), residual methanol (δ = 3.31 ppm, $^1$H; 49.00 ppm, $^{13}$C), residual benzene (δ = 7.16 ppm, $^1$H; 128.06 ppm, $^{13}$C), residual $H_2O$ (δ = 4.76 ppm, $^1$H) or residual dimethyl sulfoxide (δ = 2.50 ppm, $^1$H; 39.5 ppm, $^{13}$C). Chemical shifts are reported in parts per million (ppm). Multiplicities are indicated by s (singlet), d (doublet), t (triplet), q (quartet), m (multiplet), and br (broad). Coupling constants *J* are reported in Hertz (Hz). Mass spectrometry (MS) was performed by the University of Illinois Mass Spectrometry Laboratory. Electrospray ionization (ESI+) spectra were performed using a time-of-flight (TOF) mass analyzer. Data are reported in the form of m/z (intensity relative to the base peak = 100). Infrared spectra were measured neat on a Perkin-Elmer spectrum BX FT-IR spectrometer. Peaks are reported in $cm^{-1}$ with indicated relative intensities: s (strong, 0–33% T); m (medium, 34–66% T), w (weak, 67–100% T), and br (broad). Visible-light spectrum of LED was recorded using an Avantes Sensline Avaspec-ULS TEC Spectrometer. Melting points of solids, compounds that solidified after chromatography, were measured on a Buchi B-540 melting point apparatus and are uncorrected. The x-ray diffraction experiments were conducted using Bruker D8 Venture/Photon 100 diffractometer or Bruker APEX-II CCD diffractometer. Using Olex2,[6] the structure was solved with ShelXT[7] structure solution program using Intrinsic Phasing solution method, and the XL[8] refinement package using Least Squares minimization.

## 2. Experimental set-up

### 2-1. LED light source:

Generic cool white light LED corn bulbs were used for the photochemical experiments. These can be obtained from several manufacturers over amazon.com and proved to give consistent results as well as identical visible spectra. Detailed info:

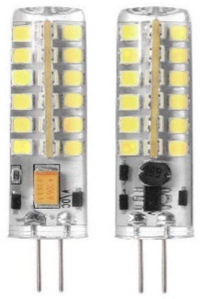

Socket: G4
LED Chip: 48 LEDs SMD 2835
Consume wattage: 4W
Input voltage: AC / DC 12V
Beam degree: 360 degrees
Color temperature: 6500K (Cool White)
Initial Lumens (lm): 290

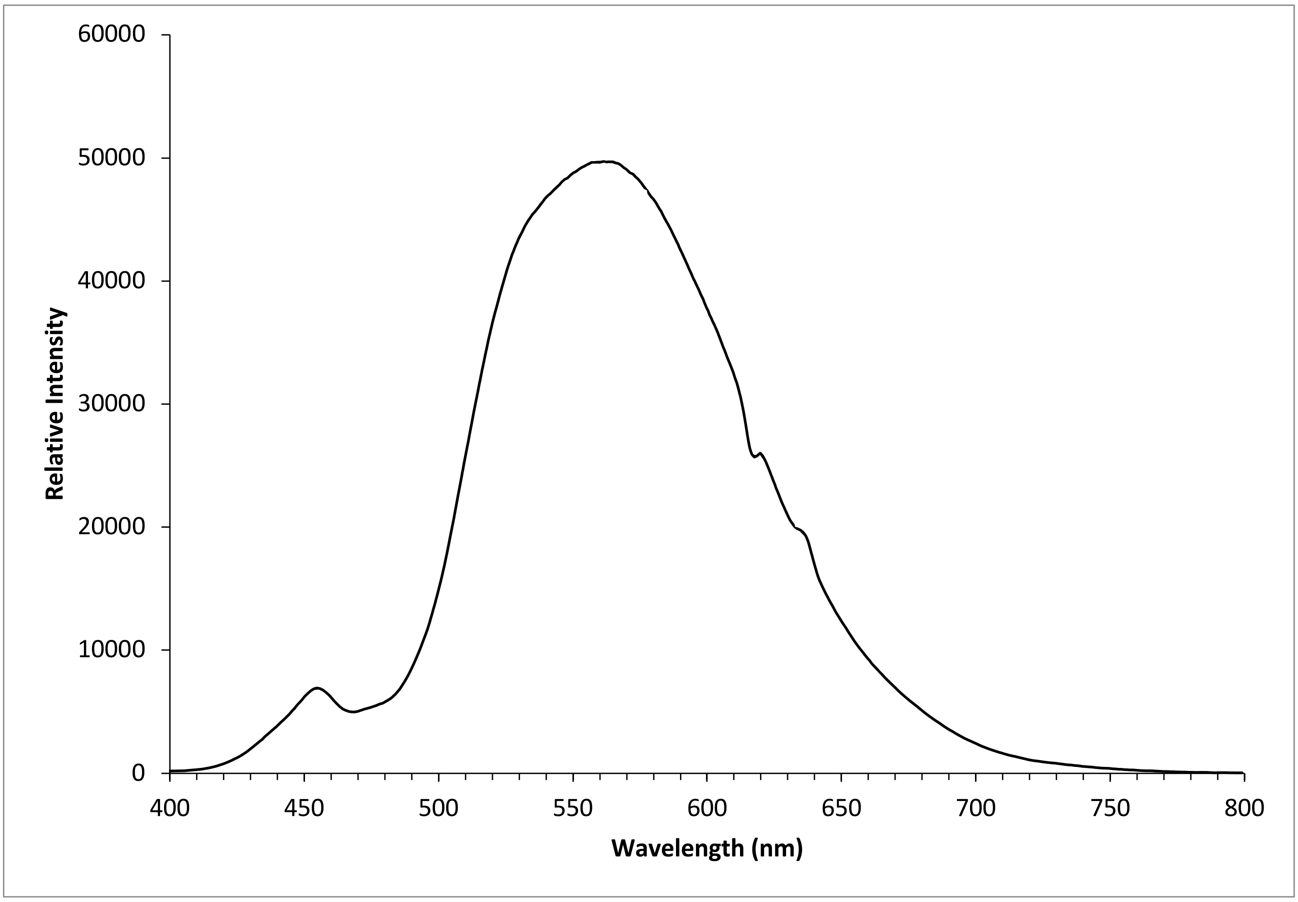


**Spectra S1.** Spectrum of a LED bulb used.

## 2-2. Set-up for small scale reactions (<2.0 mmol scale)

Six 4 W LED corn bulbs (12V, cool white light 6500K) were wired to a suitable 12V power supply, then sealed into test tubes and capped with septa (Picture S1). Lights and reaction tubes were arranged in a merry-go-round fashion for maximal exposure of each reaction vessel to light source and submerged in a −78 °C bath. Generally, up to four 1.0 mmol scale reactions can be run in the same bath using five 4 W lamps positioned around them.

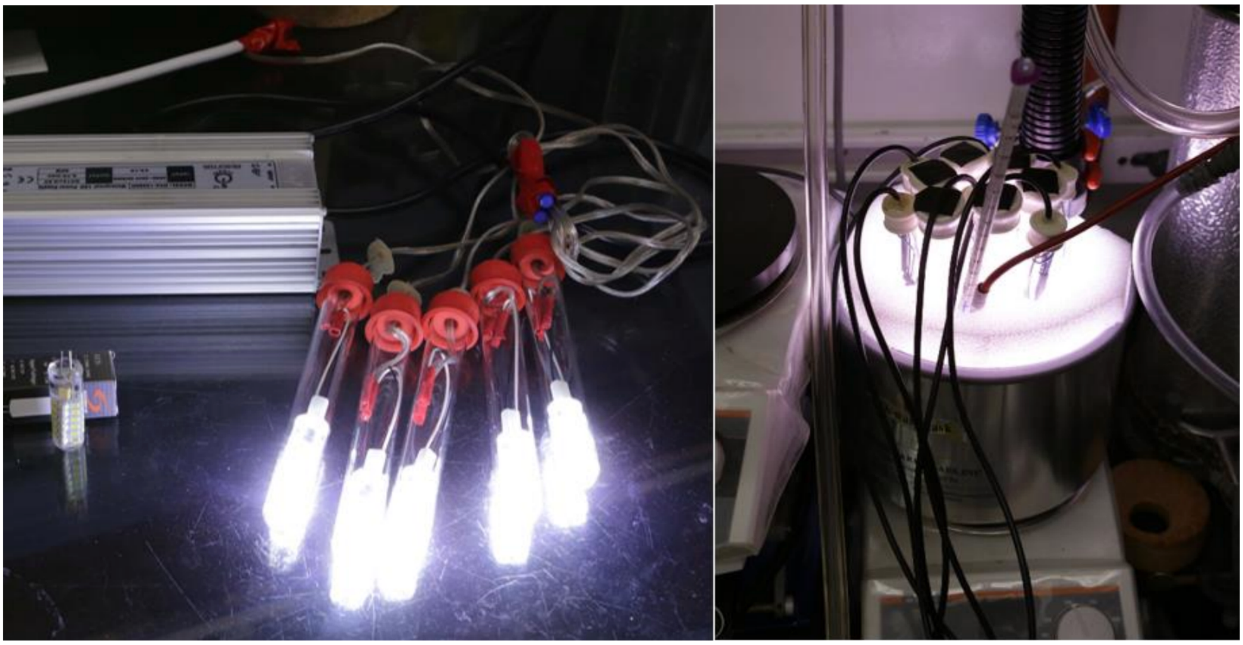

**Picture S1.** Assembly of LED bulbs from small-scale photochemical reactions.

## 2-3. Set-up for large scale reactions (2 - 10 mmol scale)

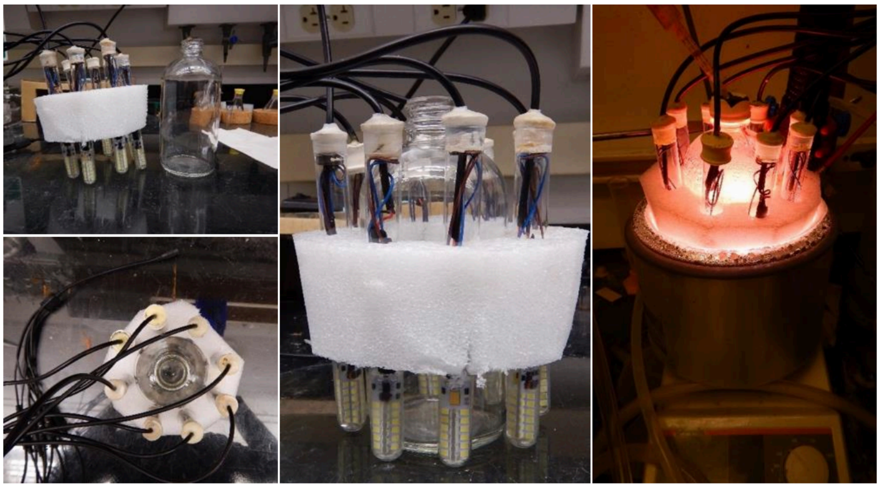

**Picture S2.** Photochemical set-up for large scale reactions.

## 3. Experimental procedures

### 3-1. Optimization of arenophile-mediated cyclopropanation of naphthalene

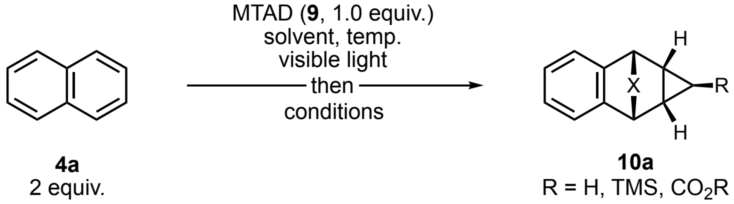


| Entry | TM [mol%] | Reagent [equiv.] | Solvent | Temp. [°C] | Yield [%] |
|---|---|---|---|---|---|
| 1 | - | $EtZn_2$ (5) | $CH_2Cl_2$ | -78 to 25 | 0 |
| 2 | $Rh_2(OAc)_4$ (5) | $N_2CHCO_2Et$ (3) | $CH_2Cl_2$ | -78 to 25 | 0 |
| 3 | $[Rh_2(cap)_4]$ (5) | $N_2CHCO_2Et$ (3) | $CH_2Cl_2$ | -78 to 25 | 0 |
| 4 | Fe(TPP) (10) | $N_2CHCO_2Et$ (3) | $CH_2Cl_2$ | -78 to 25 | 0 |
| 5 | $(Ph_3P)AuCl$ (5) | $N_2CHCO_2Et$ (3) | $CH_2Cl_2$ | -78 to 25 | 0 |
| 6 | JohnPhosAuCl (5) | $N_2CHCO_2Et$ (3) | $CH_2Cl_2$ | -78 to 25 | 0 |
| 7 | [(IPr)AuCl] (5) | $N_2CHCO_2Et$ (3) | $CH_2Cl_2$ | -78 to 25 | 0 |
| 8 | CuOTf (2) | $N_2CHCO_2Et$ (3) | $CH_2Cl_2$ | -78 to 25 | 0 |
| 9 | $Pd(OAc)_2$ (5) | $CH_2N_2$ (3) | $CH_2Cl_2$ | -78 to 25 | 16 |
| 10 | $Pd(OAc)_2$ (5) | $TMSCHN_2$ (3) | $CH_2Cl_2$ | -78 to 25 | 5 |
| 11 | $Pd(OAc)_2$ (5) | $TMSCHN_2$ (3) | $CH_2Cl_2$ | -50 to 25 | 28 |
| 12 | $Pd(TFA)_2$ (5) | $TMSCHN_2$ (3) | $CH_2Cl_2$ | -50 to 25 | 45 |
| 13 | $Pd(Piv)_2$ (5) | $TMSCHN_2$ (3) | $CH_2Cl_2$ | -50 to 25 | 51 |
| 14 | $PdCl_2$ (5) | $TMSCHN_2$ (3) | $CH_2Cl_2$ | -50 to 25 | 52 |
| 15 | $PdCl_2$ (5) | $TMSCHN_2$ (3) | EtCN | -50 to 25 | 0 |
| 16 | $PdCl_2$ (5) | $TMSCHN_2$ (3) | acetone | -50 to 25 | 51 |
| 17 | $PdCl_2$ (5) | $TMSCHN_2$ (3) | EtOAc | -50 to 25 | 58 |
| 18 | $Pd(OAc)_2$ (5) | $TMSCHN_2$ (3) | EtOAc | -50 to 25 | 50 |
| 19 | $Pd(Piv)_2$ (5) | $TMSCHN_2$ (3) | EtOAc | -50 to 25 | 57 |
| 20 | $Pd(TFA)_2$ (5) | $TMSCHN_2$ (3) | EtOAc | -50 to 25 | 60 |
| 21 | $[Pd(allyl)Cl]_2$ (5) | $TMSCHN_2$ (3) | EtOAc | -50 to 25 | 35 |
| 22 | $PdCl_2(PPh_3)_2$ (5) | $TMSCHN_2$ (3) | EtOAc | -50 to 25 | 0 |
| 23 | $Pd_2dba_3$ (5) | $TMSCHN_2$ (3) | EtOAc | -50 to 25 | 64 |
| 24 | $Pd_2dba_3{\cdot}CHCl_3$ (5) | $TMSCHN_2$ (3) | EtOAc | -50 to 25 | 79 |
| 25 | $Pd_2dba_3{\cdot}CHCl_3$ (5) | $TMSCHN_2$ (1.5) | EtOAc | -50 to 25 | 35 |

## 3-2. General procedure for dearomative cyclopropanation with polycyclic arenes

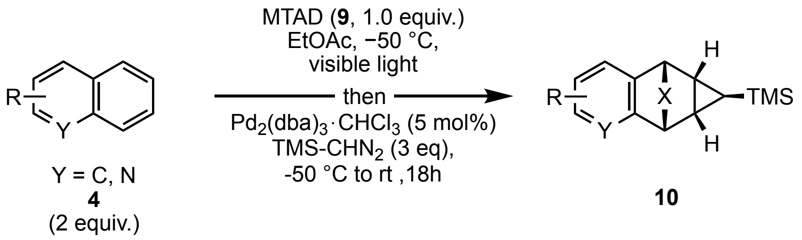


A solution of *N*-methyl-1,2,4-triazoline-3,5-dione (**9**, MTAD, 113.4 mg, 1.0 mmol, 1.0 equiv.) and arene (**4**, 2.0 mmol, 2.0 equiv.) in dry and degassed ethyl acetate (5.0 mL) was irradiated with LED lights at −50 °C under a nitrogen atmosphere. Upon decolorization, which generally proceeds within 8 – 12 hours with the described setup, a suspension of $Pd_2(dba)_3 \cdot CHCl_3$ (51.76 mg, 0.05 mmol, 0.05 equiv.) in dry and degassed ethyl acetate (2 mL, sonicated for 1 minute), was added dropwise to the solution, followed by dropwise addition of (trimethylsilyl)diazomethane (2.0 M solution in hexanes, 1.5 mL, 3.0 mmol, 3.0 equiv.). After addition, the cold bath was allowed to slowly warm up from −50 °C to room temperature, over 18 hours. The reaction was quenched by adding acetic acid (350 µL, 6 equiv.) and left to stir for 15 minutes. The solution was filtered through celite and concentrated under reduced pressure. The residue was purified by flash chromatography to give the desired compound **10**.

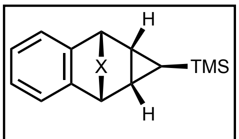


**10a**

**2-methyl-14-(trimethylsilyl)-5,10-dihydro-1H-5,10-[1,2]epicyclopropa[1,2,4]triazolo[1,2-b]phthalazine-1,3(2H)-dione (10a):**

Following General Procedure (1.0 mmol scale) the title compound was isolated by flash chromatography ($SiO_2$, hexanes:ethyl acetate = 9:1 to 4:1) as a yellow solid (259 mg, 79%).

**$R_f$** = 0.25 ($SiO_2$, hexanes:ethyl acetate = 4:1, UV, $KMnO_4$)

**$^1$H NMR:** (500 MHz, $CDCl_3$) δ 7.36 (dd, *J* = 5.4, 3.2 Hz, 2H), 7.20 (dd, *J* = 5.4, 3.2 Hz, 2H), 5.54 (dd, *J* = 3.2, 2.0 Hz, 2H), 2.83 (s, 3H), 1.63 (ddd, *J* = 5.2, 3.3, 2.0 Hz, 2H), -0.09 (s, 9H), -1.16 (t, *J* = 5.2 Hz, 1H)

**$^{13}$C NMR:** (126 MHz, $CDCl_3$) δ 157.34, 131.09, 129.23, 124.02, 57.89, 25.22, 12.61, -2.26

**HRMS**: (ESI-TOF, m/z) calcd. for $C_{17}H_{21}N_3O_2Si$ $[M + H]^+$, 328.1481; found: 328.1476

**IR:** (ATR, neat, $cm^{-1}$) = 2954 (w), 1771 (m), 1704 (s), 1446 (m), 1388 (m), 1246 (m), 1227 (w), 1202 (w), 1170 (w), 1047 (w), 964 (m), 923 (w), 837 (s), 755 (m), 701 (w), 666 (w), 555 (m), 505 (w)

**m.p.** = 166 – 169 °C

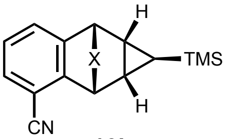


**10b**

**2-methyl-1,3-dioxo-14-(trimethylsilyl)-2,3,5,10-tetrahydro-1*H*-5,10-[1,2]epicyclopropa[1,2,4]triazolo[1,2-*b*]phthalazine-6-carbonitrile (10b):**

Following General Procedure (1.0 mmol scale) the title compound was isolated by flash chromatography ($SiO_2$, hexanes:ethyl acetate = 9:1) as a yellow solid (261 mg, 74%).

**$R_f$** = 0.25 ($SiO_2$, hexanes:ethyl acetate = 9:1, UV, $KMnO_4$)

**$^1$H NMR** (500 MHz, $CDCl_3$) δ 7.67 (dd, *J* = 7.8, 1.3 Hz, 1H), 7.56 – 7.39 (m, 2H), 5.91 (d, *J* = 4.8 Hz, 1H), 5.57 (d, *J* = 4.8 Hz, 1H), 2.83 (s, 3H), 1.71 (dt, *J* = 7.4, 5.0 Hz, 1H), 1.66 (dt, *J* = 7.5, 4.9 Hz, 1H), -0.09 (s, 10H), -1.28 (t, *J* = 5.1 Hz, 1H).

**$^{13}$C NMR** (126 MHz, $CDCl_3$) δ 157.34, 157.05, 134.23, 132.62, 132.32, 129.90, 128.55, 115.80, 108.62, 57.40, 55.72, 25.42, 12.06, 11.88, 8.85, -2.26.

**HRMS**: (ESI-TOF, m/z) calcd. for $C_{18}H_{20}N_4O_2Si$ $[M + H]^+$, 353.1434; found: 353.1430

**IR:** (ATR, neat, $cm^{-1}$) = 2228 (w), 1771 (m), 1705 (s), 1447 (m), 1396 (m), 1247 (m), 1228 (w), 1201 (w), 1174 (w), 1046 (w), 963 (m), 926 (w), 837 (s), 767 (m), 710 (w), 656 (w), 522 (m), 477 (w)

**m.p.** = 229 – 232 °C

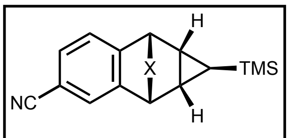


**10c**

**2-methyl-1,3-dioxo-14-(trimethylsilyl)-2,3,5,10-tetrahydro-1H-5,10-[1,2]epicyclopropa[1,2,4]triazolo[1,2-b]phthalazine-7-carbonitrile (10c):**

Following General Procedure (1.0 mmol scale) the title compound was isolated by flash chromatography ($SiO_2$, hexanes:ethyl acetate = 9:1) as a yellow solid (176 mg, 50%).

**$R_f$** = 0.25 ($SiO_2$, hexanes:ethyl acetate = 9:1, UV, $KMnO_4$)

**$^1$H NMR** (500 MHz, $CDCl_3$) δ 7.69 (dd, *J* = 7.7, 1.5 Hz, 1H), 7.49 (d, *J* = 1.4 Hz, 1H), 7.32 (d, *J* = 7.7 Hz, 1H), 5.57 (q, *J* = 2.9 Hz, 2H), 2.83 (s, 3H), 1.67 (dt, *J* = 5.3, 2.5 Hz, 2H), -0.08 (s, 9H), -1.23 (t, *J* = 5.1 Hz, 1H).

**$^{13}$C NMR** (126 MHz, $CDCl_3$) δ 157.18, 135.55, 133.60, 132.21, 127.57, 125.06, 117.98, 113.46, 57.37, 57.18, 25.42, 12.24, 12.07, 9.05, -2.26.

**HRMS**: (ESI-TOF, m/z) calcd. for $C_{18}H_{20}N_4O_2Si$ $[M + H]^+$, 353.1434; found: 353.1432

**IR:** (ATR, neat, $cm^{-1}$) = 2229 (w), 1770 (m), 1705 (s), 1448 (m), 1394 (m), 1245 (m), 1199 (w), 1177 (w), 1001 (w), 972 (w), 962 (m), 869 (s), 830 (s), 767 (m), 710 (w), 656 (w), 542 (m), 489 (w)

**m.p.** = 182 – 184 °C

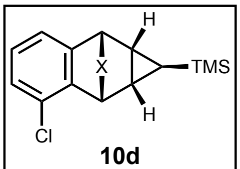


**10d**

**6-chloro-2-methyl-14-(trimethylsilyl)-5,10-dihydro-1*H*-5,10-[1,2]epicyclopropa[1,2,4]triazolo[1,2-*b*]phthalazine-1,3(2*H*)-dione (10d):**

Following General Procedure (1.0 mmol scale) the title compound was isolated by flash chromatography ($SiO_2$, hexanes:ethyl acetate = 9:1) as a yellow solid (217 mg, 60%).

**$R_f$** = 0.25 ($SiO_2$, hexanes:ethyl acetate = 4:1, UV, $KMnO_4$)

$^1$H NMR (500 MHz, $CDCl_3$) δ 7.35 (dd, *J* = 8.2, 1.1 Hz, 1H), 7.26 (dd, *J* = 8.2, 7.4 Hz, 1H), 7.09 – 7.05 (m, 1H), 5.98 (d, *J* = 4.8 Hz, 1H), 5.49 (d, *J* = 4.8 Hz, 1H), 2.81 (s, 3H), 1.63 (dt, *J* = 7.6, 5.0 Hz, 1H), 1.59 (dt, *J* = 7.6, 4.9 Hz, 1H), -0.11 (s, 9H), -1.20 (t, *J* = 5.1 Hz, 1H).

**$^{13}$C NMR** (126 MHz, $CDCl_3$) δ 157.18, 157.05, 132.82, 130.08, 129.79, 129.61, 129.14, 122.48, 57.49, 54.49, 25.27, 11.99, 11.95, 8.56, -2.35.

**HRMS**: (ESI-TOF, m/z) calcd. for $C_{17}H_{20}ClN_3O_2Si$ $[M + H]^+$, 362.1092; found: 362.1080

**IR:** (ATR, neat, $cm^{-1}$) = 2229 (w), 1771 (m), 1705 (s), 1457 (m), 1449 (m), 1395 (m), 1246 (w), 1200 (w), 1174 (w), 1035 (w), 965 (m), 869 (m), 840 (s), 764 (m), 712 (w), 675 (w), 585 (m), 489 (w)

**m.p.** = 184 – 185 °C

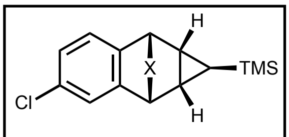


**10e**

**7-chloro-2-methyl-14-(trimethylsilyl)-5,10-dihydro-1*H*-5,10-[1,2]epicyclopropa[1,2,4]triazolo[1,2-*b*]phthalazine-1,3(2*H*)-dione (10e):**

Following General Procedure (1.0 mmol scale) the title compound was isolated by flash chromatography ($SiO_2$, hexanes:ethyl acetate = 9:1) as a yellow solid (181 mg, 50%).

**$R_f$** = 0.25 ($SiO_2$, hexanes:ethyl acetate = 4:1, UV, $KMnO_4$)

**$^1$H NMR** (500 MHz, $CDCl_3$) δ 7.34 (dd, *J* = 8.1, 2.0 Hz, 1H), 7.20 (d, *J* = 2.0 Hz, 1H), 7.13 (d, *J* = 7.9 Hz, 1H), 5.50 (dt, *J* = 10.4, 2.5 Hz, 2H), 2.84 (s, 3H), 1.62 (td, *J* = 7.2, 6.1, 3.2 Hz, 3H), -0.08 (s, 9H), -1.15 (t, *J* = 5.2 Hz, 1H).

**$^{13}$C NMR** (126 MHz, $CDCl_3$) δ 157.37, 157.32, 135.09, 132.64, 129.52, 129.46, 125.52, 124.55, 57.52, 57.39, 25.39, 12.61, 12.42, 8.89, -2.20.

**HRMS**: (ESI-TOF, m/z) calcd. for $C_{17}H_{20}ClN_3O_2Si$ $[M + H]^+$, 362.1092; found: 362.1085.

**IR:** (ATR, neat, $cm^{-1}$) = 2958 (w), 1765 (m), 1705 (s), 1451 (m), 1394 (m), 1245 (m), 1200 (w), 1174 (w), 999 (w), 967 (m), 869 (m), 837 (s), 815 (w), 765 (m), 748 (w), 637 (w), 530 (m), 506 (w).

**m.p.** = 190 – 192 °C

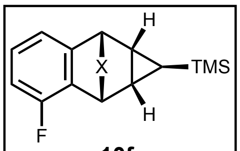


**10f**

**6-fluoro-2-methyl-14-(trimethylsilyl)-5,10-dihydro-1*H*-5,10-[1,2]epicyclopropa[1,2,4]triazolo[1,2-*b*]phthalazine-1,3(2*H*)-dione (10f):**

Following General Procedure (1.0 mmol scale) the title compound was isolated by flash chromatography ($SiO_2$, hexanes:ethyl acetate = 10:1 to 9:1) as a yellow solid (169 mg, 49%).

**$R_f$** = 0.30 ($SiO_2$, hexanes:ethyl acetate = 9:1, UV, $KMnO_4$)

**$^1$H NMR** (500 MHz, $CDCl_3$) δ 7.32 (ddd, *J* = 8.4, 7.4, 5.2 Hz, 1H), 7.12 – 7.05 (m, 1H), 6.98 (d, *J* = 7.4 Hz, 1H), 5.90 (d, *J* = 4.6 Hz, 1H), 5.53 (dd, *J* = 4.6, 1.7 Hz, 1H), 2.84 (s, 3H), 1.63 (ddt, *J* = 12.0, 7.7, 3.9 Hz, 2H), -0.09 (s, 9H), -1.14 (t, *J* = 5.1 Hz, 1H).

**$^{13}$C NMR** (126 MHz, $CDCl_3$) δ 157.24, 157.18, 156.66 (d, *J* = 250.7 Hz), 133.47 (d, *J* = 4.6 Hz), 130.66 (d, *J* = 7.5 Hz), 119.81 (d, *J* = 3.6 Hz), 117.96 (d, *J* = 18.3 Hz), 116.35 (d, *J* = 20.7 Hz), 57.26, 51.66, 25.34, 12.32, 8.74, -2.25.

**$^{19}$F NMR** (471 MHz, $CDCl_3$) δ -122.86.

**HRMS**: (ESI-TOF, m/z) calcd. for $C_{17}H_{20}FN_3O_2Si$ $[M + H]^+$, 346.1387; found: 346.1380

**IR:** (ATR, neat, $cm^{-1}$) = 2959 (w), 1772 (m), 1705 (s), 1476 (m), 1448 (m), 1392 (m), 1247 (m), 1204 (w), 1154 (w), 994 (w), 962 (m), 834 (s), 815 (w), 758 (m), 746 (w), 738 (w), 731 (m), 472 (w)

**m.p.** = 144 – 147 °C

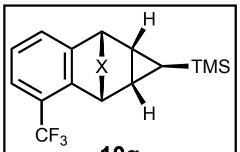


**10g**

**2-methyl-6-(trifluoromethyl)-14-(trimethylsilyl)-5,10-dihydro-1*H*-5,10-[1,2]epicyclopropa[1,2,4]triazolo[1,2-*b*]phthalazine-1,3(2*H*)-dione (10g):**

Following General Procedure (1.0 mmol scale) the title compound was isolated by flash chromatography ($SiO_2$, hexanes:ethyl acetate = 9:1) as a yellow solid (237 mg, 60%).

**$R_f$** = 0.25 ($SiO_2$, hexanes:ethyl acetate = 4:1, UV, $KMnO_4$)

**$^1$H NMR** (500 MHz, $CDCl_3$) δ 7.66 (d, *J* = 8.0 Hz, 1H), 7.48 (t, *J* = 7.7 Hz, 1H), 7.39 (d, *J* = 7.5 Hz, 1H), 5.97 (d, *J* = 4.7 Hz, 1H), 5.58 (d, *J* = 4.4 Hz, 1H), 2.85 (s, 3H), 1.67 (tt, *J* = 8.0, 3.9 Hz, 2H), -0.11 (s, 9H), -1.33 (t, *J* = 5.1 Hz, 1H).

**$^{13}$C NMR** (126 MHz, $CDCl_3$) δ 157.13, 156.39, 132.75, 129.62, 129.25, 127.89, 125.95 (q, *J* = 4.7 Hz), 125.9 (q, *J* = 27 Hz), 123.37 (q, *J* = 274 Hz), 57.15, 54.32, 25.43, 12.04, 9.01, -2.41.

**$^{19}$F NMR** (471 MHz, $CDCl_3$) δ -60.10.

**HRMS**: (ESI-TOF, m/z) calcd. for $C_{18}H_{20}F_3N_3O_2Si$ $[M + H]^+$, 396.1355; found: 396.1342.

**IR:** (ATR, neat, $cm^{-1}$) = 2961 (w), 1766 (m), 1716 (s), 1457 (m), 1397 (m), 1320 (m), 1249 (m), 1211 (w), 1176 (w), 1163 (m), 1124 (s), 968 (m), 871 (w), 841 (m), 758 (m), 743 (w), 731 (m), 555 (w)

**m.p.** = 213 – 215 °C

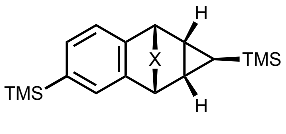


**10h**

**2-methyl-7,14-bis(trimethylsilyl)-5,10-dihydro-1*H*-5,10-[1,2]epicyclopropa[1,2,4]triazolo[1,2-*b*]phthalazine-1,3(2*H*)-dione (10h):**

Following General Procedure (1.0 mmol scale) the title compound was isolated by flash chromatography ($SiO_2$, hexanes:ethyl acetate = 10:1) as a yellow solid (180 mg, 45%).

**$R_f$** = 0.3 ($SiO_2$, hexanes:ethyl acetate = 9:1, UV, $KMnO_4$)

**$^1$H NMR** (500 MHz, $CDCl_3$) δ 7.52 – 7.46 (m, 1H), 7.26 (d, *J* = 3.6 Hz, 1H), 7.13 (d, *J* = 7.3 Hz, 1H), 5.50 (t, *J* = 4.2 Hz, 2H), 2.82 (s, 3H), 1.59 (q, *J* = 4.7 Hz, 2H), 0.24 (s, 9H), -0.11 (s, 10H), -1.20 (t, *J* = 5.1 Hz, 1H).

**$^{13}$C NMR** (126 MHz, $CDCl_3$) δ 157.02, 142.21, 134.31, 131.60, 130.40, 128.49, 123.13, 76.93, 57.97, 57.69, 25.27, 12.88, 12.69, 8.51, -1.02, -2.21.

**HRMS**: (ESI-TOF, m/z) calcd. for $C_{20}H_{29}N_3O_2Si_2$ $[M + H]^+$, 400.1877; found: 400.1867.

**IR:** (ATR, neat, $cm^{-1}$) = 2950 (w), 1766 (m), 1704 (s), 1449 (m), 1392 (m), 1248 (w), 1206 (m), 1170 (w), 995 (m), 954 (w), 868 (m) 828 (s), 779 (m), 751 (m), 733 (m), 693 (w), 634 (m).

**m.p.** = 67 – 70 °C

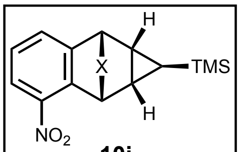


**10i**

**2-methyl-6-nitro-14-(trimethylsilyl)-5,10-dihydro-1*H*-5,10-[1,2]epicyclopropa[1,2,4]triazolo[1,2-*b*]phthalazine-1,3(2*H*)-dione (10i):**

Following General Procedure (1.0 mmol scale) the title compound was isolated by flash chromatography ($SiO_2$, hexanes:ethyl acetate = 9:1) as a yellow solid (242 mg, 65%).

**R$_f$** = 0.30 ($SiO_2$, hexanes:ethyl acetate = 4:1, UV, $KMnO_4$)

**$^1$H NMR** (500 MHz, $CDCl_3$) δ 8.11 (dd, *J* = 8.3, 1.3 Hz, 1H), 7.57 – 7.46 (m, 2H), 6.61 (d, *J* = 5.2 Hz, 1H), 5.60 (d, *J* = 4.7 Hz, 1H), 2.82 (s, 3H), 1.75 (dt, *J* = 7.6, 5.2 Hz, 1H), 1.68 (dt, *J* = 7.5, 4.9 Hz, 1H), -0.09 (d, *J* = 1.2 Hz, 9H), -1.19 (t, *J* = 5.1 Hz, 1H).

**$^{13}$C NMR** (126 MHz, $CDCl_3$) δ 157.19, 156.65, 144.19, 133.54, 129.84, 126.49, 124.87, 57.20, 53.48, 25.38, 11.71, 11.49, 9.11, -2.33.

**HRMS**: (ESI-TOF, m/z) calcd. for $C_{17}H_{20}N_4O_4Si$ $[M + H]^+$, 373.1332; found: 373.1323

**IR:** (ATR, neat, $cm^{-1}$) = 2958 (w), 1770 (m), 1704 (s), 1555 (m), 1455 (m), 1401 (m), 1370 (m), 1201 (w), 1180 (w), 1001 (w), 966 (m), 869 (m), 840 (s), 820 (w), 766 (m), 747 (w), 640 (w), 531 (m), 505 (w)

**m.p.** = 218 – 220 °C

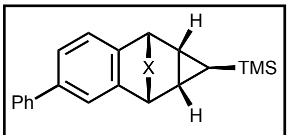


**10j**

**2-methyl-7-phenyl-14-(trimethylsilyl)-5,10-dihydro-1*H*-5,10-[1,2]epicyclopropa[1,2,4]triazolo[1,2-*b*]phthalazine-1,3(2*H*)-dione (10j):**

Following General Procedure (1.0 mmol scale) the title compound was isolated by flash chromatography ($SiO_2$, hexanes:ethyl acetate = 9:1) as a yellow solid (169 mg, 42%).

**R$_f$** = 0.3 ($SiO_2$, hexanes:ethyl acetate = 4:1, UV, $KMnO_4$)

**$^1$H NMR** (500 MHz, $CDCl_3$) δ 7.58 (dd, *J* = 9.2, 7.4 Hz, 3H), 7.46 – 7.39 (m, 3H), 7.35 (t, *J* = 7.3 Hz, 1H), 7.28 – 7.23 (m, 1H), 5.58 (dt, *J* = 9.4, 2.6 Hz, 2H), 2.84 (s, 3H), 1.69 – 1.61 (m, 2H), -0.08 (s, 9H), -1.07 (t, *J* = 5.1 Hz, 1H).

**$^{13}$C NMR** (126 MHz, $CDCl_3$) δ 157.36, 157.29, 142.22, 140.05, 131.69, 130.07, 129.01, 127.92, 127.79, 127.22, 124.47, 122.75, 58.11, 57.70, 25.34, 12.86, 12.79, 8.77, -2.19.

**HRMS**: (ESI-TOF, m/z) calcd. for $C_{23}H_{25}N_3O_2Si$ $[M + H]^+$, 404.1770; found: 404.1781.

**IR:** (ATR, neat, $cm^{-1}$) = 2952 (w), 1764 (m), 1698 (s), 1446 (m), 1388 (m), 1253 (w), 1244 (m), 1218 (w), 1171 (m), 993 (w), 967 (m) 955 (m), 834 (s), 761 (s), 751 (m), 735 (w), 568 (m).

**m.p.** = 190 – 193 °C

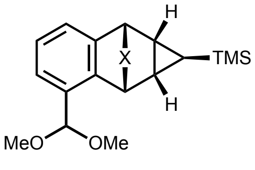


**10k**

**6-(dimethoxymethyl)-2-methyl-14-(trimethylsilyl)-5,10-dihydro-1*H*-5,10-[1,2]epicyclopropa[1,2,4]triazolo[1,2-*b*]phthalazine-1,3(2*H*)-dione (10k):**

Following General Procedure (1.0 mmol scale) the title compound was isolated by flash chromatography ($SiO_2$, hexanes:ethyl acetate = 9:1) as a yellow solid (283 mg, 62%).

**R$_f$** = 0.25 ($SiO_2$, hexanes:ethyl acetate = 4:1, UV, $KMnO_4$)

**$^1$H NMR** (500 MHz, $CDCl_3$) δ 7.55 (d, *J* = 7.9 Hz, 1H), 7.32 (t, *J* = 7.7 Hz, 1H), 7.13 (d, *J* = 7.4 Hz, 1H), 6.06 (d, *J* = 4.5 Hz, 1H), 5.61 (s, 1H), 5.50 (d, *J* = 4.2 Hz, 1H), 3.45 (s, 3H), 3.02 (s, 3H), 2.80 (s, 3H), 1.61 (q, *J* = 4.5 Hz, 2H), -0.12 (s, 9H), -1.24 (t, *J* = 5.1 Hz, 1H).

**$^{13}$C NMR** (126 MHz, $CDCl_3$) δ 157.47, 157.35, 133.17, 131.34, 129.73, 128.50, 127.61, 124.32, 100.16, 57.87, 54.85, 53.92, 49.91, 25.25, 12.28, 12.08, 8.93, -2.35.

**HRMS**: (ESI-TOF, m/z) calcd. for $C_{20}H_{27}N_3O_4Si$ $[M + Na]^+$, 424.1669; found: 424.1655.

**IR:** (ATR, neat, $cm^{-1}$) = 2951 (w), 1769 (m), 1704 (s), 1449 (m), 1393 (m), 1247 (w), 1199 (m), 1172 (w), 1144 (m), 1030 (w), 1000 (m), 964 (m), 868 (m) 836 (s), 778 (m), 764 (m), 732 (m).

**m.p.** = 73 – 74 °C

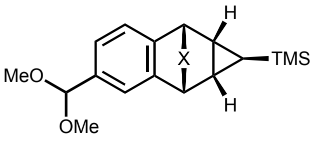


10l

**7-(dimethoxymethyl)-2-methyl-14-(trimethylsilyl)-5,10-dihydro-1*H*-5,10-[1,2]epicyclopropa[1,2,4]triazolo[1,2-*b*]phthalazine-1,3(2*H*)-dione (10l):**

Following General Procedure (1.0 mmol scale) the title compound was isolated by flash chromatography ($SiO_2$, hexanes:ethyl acetate = 9:1) as a yellow solid (217 mg, 54%).

**$R_f$** = 0.3 ($SiO_2$, hexanes:ethyl acetate = 4:1, UV, $KMnO_4$)

**$^1$H NMR** (500 MHz, $CDCl_3$) δ 7.47 – 7.42 (m, 1H), 7.28 (d, *J* = 1.6 Hz, 1H), 7.18 (d, *J* = 7.7 Hz, 1H), 5.52 (dt, *J* = 3.8, 1.4 Hz, 2H), 5.36 (s, 1H), 3.31 (s, 3H), 3.29 (s, 3H), 2.81 (s, 3H), 1.63 – 1.57 (m, 2H), -0.12 (s, 8H), -1.20 (t, *J* = 5.1 Hz, 1H).

**$^{13}$C NMR** (126 MHz, $CDCl_3$) δ 157.25, 157.21, 139.35, 131.32, 131.06, 127.53, 123.99, 122.61, 102.73, 57.99, 57.67, 53.09, 53.04, 25.25, 12.64, 12.62, 8.65, -2.23.

**HRMS**: (ESI-TOF, m/z) calcd. for $C_{20}H_{27}N_3O_4Si$ [M + Na]$^+$, 424.1669; found: 424.1661.

**IR:** (ATR, neat, cm$^{-1}$) = 2952 (w), 1768 (m), 1710 (s), 1455 (m), 1388 (m), 1245 (w), 1201 (m), 1174 (w), 1140 (m), 1025 (w), 998 (m), 965 (m), 868 (m) 837 (s), 778 (m), 764 (m), 732 (m).

**m.p.** = 69 – 71 °C

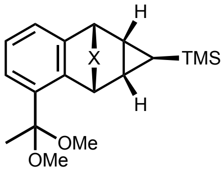


10m

**6-(1,1-dimethoxyethyl)-2-methyl-14-(trimethylsilyl)-5,10-dihydro-1*H*-5,10-[1,2]epicyclopropa[1,2,4]triazolo[1,2-*b*]phthalazine-1,3(2*H*)-dione (10m):**

Following General Procedure (1.0 mmol scale) the title compound was isolated by flash chromatography ($SiO_2$, hexanes:ethyl acetate = 9:1) as a yellow solid (283 mg, 68%).

**$R_f$** = 0.30 ($SiO_2$, hexanes:ethyl acetate = 4:1, UV, $KMnO_4$)

**$^1$H NMR** (500 MHz, $CDCl_3$) δ 7.64 (d, *J* = 8.1 Hz, 1H), 7.32 (t, *J* = 7.7 Hz, 1H), 7.12 (d, *J* = 7.3 Hz, 1H), 6.45 (d, *J* = 4.3 Hz, 1H), 5.51 (d, *J* = 4.0 Hz, 1H), 3.25 (s, 3H), 3.10 (s, 3H), 2.88 (s, 3H), 1.60 (s, 4H), -0.14 (s, 9H), -1.27 (t, *J* = 5.1 Hz, 1H).

**$^{13}$C NMR** (126 MHz, $CDCl_3$) δ 156.35, 155.88, 138.56, 132.14, 129.26, 128.65, 128.35, 124.07, 101.58, 57.37, 54.13, 49.30, 48.85, 25.92, 25.35, 12.83, 12.56, 9.01, -2.33.

**HRMS**: (ESI-TOF, m/z) calcd. for $C_{21}H_{29}N_3O_4Si$ [M + Na]$^+$, 438.1849; found: 438.1835.

**IR:** (ATR, neat, cm$^{-1}$) = 2950 (w), 1766 (m), 1704 (s), 1451 (m), 1393 (m), 1275 (w), 1249 (m), 1201 (w), 1148 (m), 1037 (w), 958 (m), 872 (m), 868 (m) 837 (s), 766 (m), 763 (m), 570 (w).

**m.p.** = 70 – 71 °C

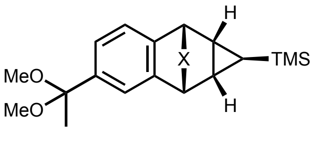


10n

**7-(1,1-dimethoxyethyl)-2-methyl-14-(trimethylsilyl)-5,10-dihydro-1*H*-5,10-[1,2]epicyclopropa[1,2,4]triazolo[1,2-*b*]phthalazine-1,3(2*H*)-dione (10n):**

Following General Procedure (1.0 mmol scale) the title compound was isolated by flash chromatography ($SiO_2$, hexanes:ethyl acetate = 9:1) as a yellow solid (233 mg, 56%).

**$R_f$** = 0.30 ($SiO_2$, hexanes:ethyl acetate = 4:1, UV, $KMnO_4$)

**$^1$H NMR** (600 MHz, $CDCl_3$) δ 7.46 (dd, *J* = 7.8, 1.7 Hz, 1H), 7.33 (d, *J* = 1.7 Hz, 1H), 7.15 (d, *J* = 7.8 Hz, 1H), 5.51 (dd, *J* = 4.0, 1.3 Hz, 2H), 3.12 (d, *J* = 3.6 Hz, 6H), 2.80 (s, 3H), 1.65 – 1.55 (m, 2H), 1.48 (s, 3H), -0.13 (s, 8H), -1.24 (t, *J* = 5.2 Hz, 1H).

**$^{13}$C NMR** (151 MHz, $CDCl_3$) δ 157.21, 157.13, 144.12, 130.95, 130.51, 127.11, 123.81, 122.17, 101.48, 58.13, 57.62, 49.07, 49.05, 26.06, 25.21, 12.73, 12.64, 8.53, -2.22.

**HRMS**: (ESI-TOF, m/z) calcd. for $C_{21}H_{29}N_3O_4Si$ [M + Na]$^+$, 438.1849; found: 438.1821.

**IR:** (ATR, neat, cm$^{-1}$) = 2952 (w), 1768 (m), 1704 (s), 1449 (m), 1392 (m), 1264 (w), 1198 (m), 1168 (m), 1143 (w), 1037 (w), 958 (m), 873 (m), 867 (m) 835 (s), 761 (m), 733 (m), 561 (w).

**m.p.** = 67 – 70 °C

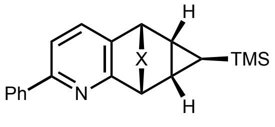


22a

**8-methyl-2-phenyl-14-(trimethylsilyl)-5,11-dihydro-7*H*-5,11-[1,2]epicyclopropapyrido[2,3-*d*][1,2,4]triazolo[1,2-*a*]pyridazine-7,9(8*H*)-dione (22a):**

Following General Procedure (1.0 mmol scale) the title compound was isolated by flash chromatography ($SiO_2$, hexanes:ethyl acetate = 9:1) as a yellow solid (182 mg, 45%).

**$R_f$** = 0.25 ($SiO_2$, hexanes:ethyl acetate = 4:1, UV, $KMnO_4$)

**$^1$H NMR** (500 MHz, $CDCl_3$) δ 8.05 – 7.97 (m, 2H), 7.74 (d, *J* = 7.8 Hz, 1H), 7.59 (d, *J* = 7.8 Hz, 1H), 7.49 – 7.44 (m, 2H), 7.44 – 7.39 (m, 1H), 5.77 (d, *J* = 4.9 Hz, 1H), 5.63 (d, *J* = 4.8 Hz, 1H), 2.88 (s, 3H), 1.73 (dt, *J* = 7.5, 5.1 Hz, 1H), 1.67 (dt, *J* = 7.6, 5.0 Hz, 1H), -0.09 (s, 8H), -1.10 (t, *J* = 5.1 Hz, 1H).

**$^{13}$C NMR** (126 MHz, $CDCl_3$) δ 158.09, 157.01, 156.53, 151.31, 138.43, 132.58, 129.60, 128.96, 127.18, 124.64, 120.81, 59.74, 56.68, 25.45, 12.89, 12.12, 8.47, -2.21.

**HRMS**: (ESI-TOF, m/z) calcd. for $C_{22}H_{24}N_4O_2Si$ $[M + H]^+$, 405.1747; found: 405.1727.

**IR:** (ATR, neat, $cm^{-1}$) = 2950 (w), 1767 (m), 1695 (s), 1456 (m), 1438 (s), 1391 (m), 1263 (m), 1249 (m), 1164 (m), 990 (w), 961 (m) 878 (m), 835 (s), 820 (m), 759 (s), 691 (s), 576 (m).

**m.p.** = 155 – 157 °C

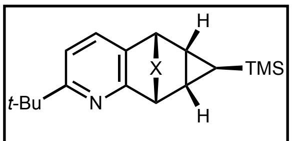


22b

**2-(*tert*-butyl)-8-methyl-14-(trimethylsilyl)-5,11-dihydro-7*H*-5,11-[1,2]epicyclopropapyrido[2,3-*d*][1,2,4]triazolo[1,2-*a*]pyridazine-7,9(8*H*)-dione (22b):**

Following General Procedure (1.0 mmol scale) the title compound was isolated by flash chromatography ($SiO_2$, hexanes:ethyl acetate = 9:1) as a yellow solid (258 mg, 67%).

**$R_f$** = 0.25 ($SiO_2$, hexanes:ethyl acetate = 4:1, UV, $KMnO_4$)

**$^1$H NMR** (500 MHz, $CDCl_3$) δ 7.41 (d, *J* = 7.9 Hz, 1H), 7.30 (d, *J* = 7.9 Hz, 1H), 5.65 (d, *J* = 4.9 Hz, 1H), 5.56 (d, *J* = 4.7 Hz, 1H), 2.88 (s, 3H), 1.66 (dt, *J* = 7.4, 5.1 Hz, 1H), 1.60 (dt, *J* = 7.5, 4.9 Hz, 1H), 1.32 (s, 9H), -0.11 (s, 9H), -1.28 (t, *J* = 5.1 Hz, 1H).

**$^{13}$C NMR** (126 MHz, $CDCl_3$) δ 170.45, 156.81, 156.34, 150.23, 131.73, 123.24, 119.32, 59.72, 56.60, 37.78, 30.29, 25.41, 12.83, 12.17, 8.06, -2.22.

**HRMS**: (ESI-TOF, m/z) calcd. for $C_{20}H_{28}N_4O_2Si$ $[M + H]^+$, 385.2060; found: 385.2052.

**IR:** (ATR, neat, $cm^{-1}$) = 2957 (w), 1769 (m), 1702 (s), 1443 (m), 1389 (s), 1263 (m), 1248 (m), 1212 (m), 1166 (m), 1026 (w), 995 (m) 963 (m), 867 (w), 832 (s), 760 (m), 746 (m), 624 (m), 543 (m).

**m.p.** = 149 – 150 °C

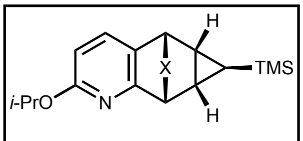


22c

**2-isopropoxy-8-methyl-14-(trimethylsilyl)-5,11-dihydro-7*H*-5,11-[1,2]epicyclopropapyrido[2,3-*d*][1,2,4]triazolo[1,2-*a*]pyridazine-7,9(8*H*)-dione (22c):**

Following General Procedure (1.0 mmol scale) the title compound was isolated by flash chromatography ($SiO_2$, hexanes:ethyl acetate = 10:1) as a yellow solid (193 mg, 50%).

**$R_f$** = 0.25 ($SiO_2$, hexanes:ethyl acetate = 9:1, UV, $KMnO_4$)

**$^1$H NMR** (500 MHz, $CDCl_3$) δ 7.34 (d, *J* = 8.2 Hz, 1H), 6.59 (d, *J* = 8.2 Hz, 1H), 5.49 (d, *J* = 4.5 Hz, 2H), 5.25 (dp, *J* = 12.3, 6.2 Hz, 1H), 2.84 (s, 3H), 1.57 (q, *J* = 5.0 Hz, 2H), 1.27 (t, *J* = 6.5 Hz, 6H), -0.12 (s, 9H), -1.20 (t, *J* = 5.2 Hz, 1H).

**$^{13}$C NMR** (126 MHz, $CDCl_3$) δ 164.14, 157.14, 156.73, 148.74, 134.69, 118.34, 111.39, 68.85, 59.36, 56.59, 25.31, 21.99, 21.97, 12.95, 11.68, 8.17, -2.29.

**HRMS**: (ESI-TOF, m/z) calcd. for $C_{19}H_{26}N_4O_3Si$ $[M + H]^+$, 387.1852; found: 387.1847.

**IR:** (ATR, neat, $cm^{-1}$) = 2950 (w), 1770 (m), 1705 (s), 1600 (m), 1452 (s), 1430 (m), 1393 (m), 1299 (m), 1250 (m), 1168 (w), 1105 (m) 972 (m), 955 (w), 868 (m), 834 (s), 808 (m), 765 (m), 726 (m), 569 (m).

**m.p.** = 150 – 151 °C

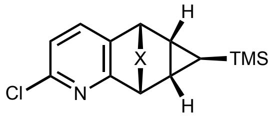


**22d**

**2-chloro-8-methyl-14-(trimethylsilyl)-5,11-dihydro-7*H*-5,11-[1,2]epicyclopropapyrido[2,3-*d*][1,2,4]triazolo[1,2-*a*]pyridazine-7,9(8*H*)-dione (22d):**

Following General Procedure (1.0 mmol scale) the title compound was isolated by flash chromatography ($SiO_2$, hexanes:ethyl acetate = 4:1) as a yellow solid (181 mg, 50%).

**$R_f$** = 0.2 ($SiO_2$, hexanes:ethyl acetate = 4:1, UV, $KMnO_4$)

**$^1$H NMR** (500 MHz, $CDCl_3$) δ 7.51 (d, *J* = 7.9 Hz, 1H), 7.36 (d, *J* = 7.9 Hz, 1H), 5.66 (d, *J* = 4.8 Hz, 1H), 5.60 (d, *J* = 4.7 Hz, 1H), 2.89 (s, 3H), 1.72 – 1.68 (m, 1H), 1.66 (dt, *J* = 7.5, 5.0 Hz, 1H), -0.08 (s, 9H), -1.21 (t, *J* = 5.2 Hz, 1H).

**$^{13}$C NMR** (126 MHz, $CDCl_3$) δ 157.03, 156.48, 152.07, 151.67, 134.60, 125.08, 125.03, 58.98, 56.18, 25.53, 12.45, 11.66, 8.67, -2.25.

**HRMS**: (ESI-TOF, m/z) calcd. for $C_{16}H_{19}ClN_4O_2Si$ $[M + H]^+$, 363.1044; found: 363.1035.

**IR:** (ATR, neat, $cm^{-1}$) = 2953 (w), 1775 (m), 1706 (s), 1567 (w), 1457 (m), 1424 (m), 1399 (m), 1253 (w), 1200 (m), 1170 (w), 1114 (m) 996 (m), 961 (m), 869 (m), 834 (s), 766 (m), 656 (m).

**m.p.** = 203 – 206 °C

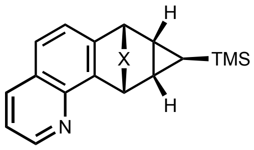


**22e**

**10-methyl-16-(trimethylsilyl)-7,13-dihydro-9*H*-7,13-[1,2]epicyclopropapyrido[2,3-*f*][1,2,4]triazolo[1,2-*b*]phthalazine-9,11(10*H*)-dione (22e):**

Following General Procedure (1.0 mmol scale) the title compound was isolated by flash chromatography ($SiO_2$, hexanes:ethyl acetate = 4:1) as a yellow solid (155 mg, 41%).

**$R_f$** = 0.25 ($SiO_2$, hexanes:ethyl acetate = 7:3, UV, $KMnO_4$)

**$^1$H NMR** (500 MHz, $CDCl_3$) δ 9.01 (dd, *J* = 4.2, 1.7 Hz, 1H), 8.17 (dd, *J* = 8.4, 1.7 Hz, 1H), 7.84 (d, *J* = 8.1 Hz, 1H), 7.45 (dd, *J* = 8.3, 4.2 Hz, 1H), 7.41 (d, *J* = 8.1 Hz, 1H), 6.87 (d, *J* = 4.8 Hz, 1H), 5.73 (d, *J* = 4.8 Hz, 1H), 2.76 (s, 3H), 1.78 (dt, *J* = 7.5, 5.0 Hz, 1H), 1.68 (dt, *J* = 7.5, 5.0 Hz, 1H), -0.13 (s, 9H), -1.26 (t, *J* = 5.1 Hz, 1H).

**$^{13}$C NMR** (126 MHz, $CDCl_3$) δ 157.70, 157.24, 151.80, 142.63, 136.47, 131.64, 129.10, 128.75, 128.41, 122.71, 121.84, 58.10, 52.77, 25.33, 12.15, 12.07, 8.75, -2.13.

**HRMS**: (ESI-TOF, m/z) calcd. for $C_{20}H_{22}N_4O_2Si$ $[M + H]^+$, 379.1590; found: 379.1584.

**IR:** (ATR, neat, $cm^{-1}$) = 2955 (w), 1769 (m), 1699 (s), 1455 (m), 1441 (m), 1391 (m), 1247 (m), 1211 (w), 1169 (m) 972 (m), 955 (w), 868 (m), 835 (s), 808 (m), 765 (m), 726 (m), 551 (m).

**m.p.** = 203 – 206 °C

## 3-3. General procedure A for the synthesis of benzocycloheptatrienes

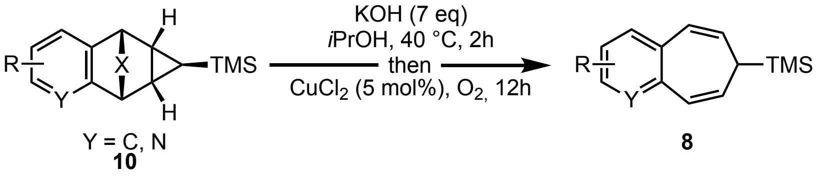


To a vial containing finely ground KOH (156 mg, 5.0 equiv., 90 wt%), and substrate (**13**, 0.500 mmol, 1.0 equiv.) under nitrogen was added *i*-PrOH (5.0 mL, 0.1 M) and degassed with nitrogen/sonication for 15 min. The reaction was heated to 40 °C with vigorous stirring (700 rpm) and progress was monitored by TLC in 30 min intervals. Upon completion, the reaction was cooled in an ice bath and $H_2O$ (5.0 mL) was added. AcOH is then carefully added dropwise until pH = 5, upon which gas evolution is observed. $CuCl_2$ dihydrate (4.3 mg, 25 μmol, 5.0 mol%). was then added as a solid, followed by sparging with oxygen, and the reaction was stirred for 10-16 h under an atmosphere of oxygen (balloon). Upon completion, the reaction was partitioned between $CH_2Cl_2$ (10 mL) and saturated brine (10 mL) and the organic layer was extracted with $CH_2Cl_2$ (3 × 10 mL). The combined organics were washed with brine, dried over $MgSO_4$, filtered, concentrated under vacuum, and isolated by flash chromatography ($SiO_2$, hexanes:ethyl acetate mixtures).

## 3-4. General procedure B for the synthesis of benzocycloheptatrienes

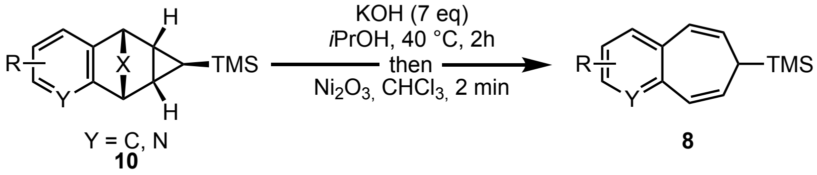


To a vial containing finely ground KOH (156 mg, 5.0 equiv., 90 wt%), and substrate (**13**, 0.500 mmol, 1.0 equiv.) under nitrogen was added *i*-PrOH (5.0 mL, 0.1 M) and degassed with nitrogen/sonication for 15 min. The reaction was heated to 40 °C with vigorous stirring (700 rpm) and progress was monitored by TLC in 30 min intervals. Upon completion, the reaction was cooled in an ice bath and $H_2O$ (5.0 mL) was added. AcOH is then carefully added dropwise until pH = 5, upon which gas evolution is observed. The semicarbazide intermediate was then extracted out with ethyl acetate (3 × 5 mL). The organic layers were combined, dried with $MgSO_4$, and concentrated under reduced pressure. This mixture containing the semicarbazide was added to the vial, followed by $CHCl_3$ (5.0 mL, 0.1 M), and sparged with nitrogen for 15 minutes. Next, nickel oxide ($Ni_2O_3$, 30% active basis, 830 mg, 3.0 equiv.) was added as a solid under a stream of nitrogen (note: vigorous gas evolution was observed). The solution was agitated 2 minutes, filtered through a celite plug, washed thoroughly with $CH_2Cl_2$, concentrated under vacuum, and isolated by flash chromatography ($SiO_2$, hexanes:ethyl acetate mixtures).

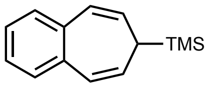


**8a**

**(7*H*-benzo[7]annulen-7-yl)trimethylsilane (8a):**

Following General Procedure A (0.5 mmol scale) the title compound was isolated by flash chromatography ($SiO_2$, hexanes:ethyl acetate = 100:0 to 50:1) as a yellow oil (81 mg, 75%).

$\mathbf{R_f}$ = 0.4 ($SiO_2$, hexanes:ethyl acetate = 50:1, UV, $KMnO_4$)

**$^{1}$H NMR** (500 MHz, $CDCl_3$) δ 7.00 (dd, *J* = 5.7, 3.3 Hz, 2H), 6.89 (dd, *J* = 5.6, 3.4 Hz, 2H), 6.09 (d, *J* = 11.3 Hz, 2H), 5.46 (dd, *J* = 11.3, 8.3 Hz, 2H), 2.34 (t, *J* = 8.3 Hz, 1H), -0.01 (s, 9H).

**$^{13}$C NMR** (126 MHz, $CDCl_3$) δ 137.92, 132.83, 130.71, 128.70, 126.60, 37.05, -2.88.

**HRMS**: (ESI-TOF, m/z) calcd. for $C_{14}H_{19}Si$ $[M + H]^+$, 214.1178; found: 214.1177.

**IR:** (ATR, neat, $cm^{-1}$) = 2953 (w), 1770 (w), 1449 (w), 1439 (w), 1314 (w), 1247 (m), 1114 (w), 1058 (w) 1038 (w), 1001 (w), 834 (s), 790 (m), 747 (m), 728 (m), 630 (m), 484 (m).

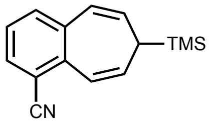


**8b**

**7-(trimethylsilyl)-7*H*-benzo[7]annulene-1-carbonitrile (8b):**

Following General Procedure A (0.5 mmol scale) the title compound was isolated by flash chromatography ($SiO_2$, hexanes:ethyl acetate = 100:0 to 50:1) as a yellow oil (78 mg, 65%).

$\mathbf{R_f}$ = 0.4 ($SiO_2$, hexanes:ethyl acetate = 50:1, UV, $KMnO_4$)

**$^{1}$H NMR** (500 MHz, $CDCl_3$) δ 7.34 (dd, *J* = 6.0, 3.2 Hz, 1H), 7.11 – 7.05 (m, 2H), 6.53 (d, *J* = 11.5 Hz, 1H), 6.10 (d, *J* = 11.4 Hz, 1H), 5.75 (ddd, *J* = 11.6, 8.6, 1.5 Hz, 1H), 5.58 (ddd, *J* = 11.6, 8.4, 1.5 Hz, 1H), 2.43 (t, *J* = 8.5 Hz, 1H), -0.03 (s, 9H).

**$^{13}$C NMR** (126 MHz, $CDCl_3$) δ 140.95, 139.15, 138.49, 136.05, 134.68, 131.00, 127.23, 126.72, 124.43, 118.36, 112.47, 37.65, -2.81.

**HRMS**: (ESI-TOF, m/z) calcd. for $C_{15}H_{18}NSi$ $[M + H]^+$, 240.1209; found: 240.1207.

**IR:** (ATR, neat, $cm^{-1}$) = 2953 (w), 2225 (m), 1460 (w), 1248 (m), 1102 (w), 1080 (w), 1061 (w) 1009 (w), 983 (w), 835 (s), 805 (w), 782 (m), 757 (m), 732 (m), 650 (m), 544 (w), 484 (m).

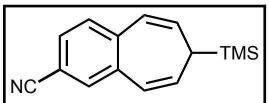


**8c**

**7-(trimethylsilyl)-7*H*-benzo[7]annulene-2-carbonitrile (8c):**

Following General Procedure A (0.5 mmol scale) the title compound was isolated by flash chromatography ($SiO_2$, hexanes:ethyl acetate = 100:0 to 50:1) as a yellow oil (78 mg, 65%).

$\mathbf{R_f}$ = 0.4 ($SiO_2$, hexanes:ethyl acetate = 50:1, UV, $KMnO_4$)

**$^{1}$H NMR** (500 MHz, $CDCl_3$) δ 7.42 – 7.36 (m, 1H), 7.06 (d, *J* = 7.9 Hz, 1H), 6.18 (dd, *J* = 23.0, 11.5 Hz, 2H), 5.73 (ddt, *J* = 11.2, 8.3, 1.2 Hz, 1H), 5.69 – 5.60 (m, 1H), 2.56 (t, *J* = 8.2 Hz, 1H), 0.14 (d, *J* = 1.1 Hz, 9H).

**$^{13}$C NMR** (126 MHz, $CDCl_3$) δ 140.95, 139.15, 138.49, 136.05, 134.68, 131.00, 127.23, 126.72, 124.43, 118.36, 112.47, 37.65, -2.81.

**HRMS**: (ESI-TOF, m/z) calcd. for $C_{15}H_{18}NSi$ $[M + H]^+$, 240.1209; found: 240.1208.

**IR:** (ATR, neat, $cm^{-1}$) = 2952 (w), 2224 (m), 1494 (w), 1246 (m), 1126 (w), 1081 (w), 1060 (w) 1009 (w), 983 (w), 830 (s), 805 (w), 789 (m), 757 (m), 728 (m), 650 (m), 544 (w), 497 (m).

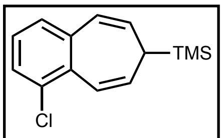


**8d**

**(1-chloro-7*H*-benzo[7]annulen-7-yl)trimethylsilane (8d):**

Following General Procedure A (0.5 mmol scale) the title compound was isolated by flash chromatography ($SiO_2$, hexanes:ethyl acetate = 100:0 to 50:1) as a yellow oil (99 mg, 79%).

$\mathbf{R_f}$ = 0.4 ($SiO_2$, hexanes:ethyl acetate = 50:1, UV, $KMnO_4$)

**$^{1}$H NMR** (500 MHz, $CDCl_3$) δ 7.14 (dd, *J* = 8.0, 1.3 Hz, 1H), 6.99 (t, *J* = 7.8 Hz, 1H), 6.88 (d, *J* = 7.6 Hz, 1H), 6.57 (d, *J* = 11.4 Hz, 1H), 6.19 (d, *J* = 11.2 Hz, 1H), 5.72 (dd, *J* = 11.5, 8.8 Hz, 1H), 5.68 – 5.61 (m, 1H), 2.37 (t, *J* = 8.7 Hz, 1H), -0.03 – -0.07 (m, 9H).

**$^{13}C$ NMR** (126 MHz, $CDCl_3$) δ 139.87, 135.42, 135.40, 135.37, 134.83, 133.95, 129.26, 127.87, 127.45, 127.26, 124.55, 36.32, -2.57.

**HRMS**: (ESI-TOF, m/z) calcd. for $C_{14}H_{18}ClSi$ $[M + H]^+$, 247.0710; found: 247.0715.

**IR:** (ATR, neat, $cm^{-1}$) = 2954 (w), 1556 (w), 1459 (w), 1432 (m), 1248 (s), 1131 (w), 1030 (w), 899 (w), 834 (s), 808 (s), 794 (m), 763 (m), 730 (m), 716 (m), 691 (w), 654 (m), 520 (w).

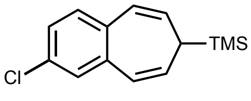


**8e**

**(2-chloro-7*H*-benzo[7]annulen-7-yl)trimethylsilane (8e):**

Following General Procedure A (0.5 mmol scale) the title compound was isolated by flash chromatography ($SiO_2$, hexanes:ethyl acetate = 100:0 to 50:1) as a yellow oil (85 mg, 68%).

**$R_f$** = 0.4 ($SiO_2$, hexanes:ethyl acetate = 50:1, UV, $KMnO_4$)

**$^1H$ NMR** (500 MHz, $CDCl_3$) δ 6.95 (dd, *J* = 8.2, 2.3 Hz, 1H), 6.87 (d, *J* = 2.3 Hz, 1H), 6.80 (d, *J* = 8.1 Hz, 1H), 6.01 (dd, *J* = 19.1, 11.5 Hz, 2H), 5.54 – 5.42 (m, 2H), 2.36 (t, *J* = 8.2 Hz, 1H), -0.00 (s, 9H).

**$^{13}C$ NMR** (126 MHz, $CDCl_3$) δ 139.58, 136.41, 134.47, 133.22, 131.97, 131.94, 130.25, 127.65, 127.41, 126.44, 37.29, -2.88.

**HRMS**: (ESI-TOF, m/z) calcd. for $C_{14}H_{18}ClSi$ $[M + H]^+$, 247.0710; found: 247.0718.

**IR:** (ATR, neat, $cm^{-1}$) = 2954 (w), 1586 (w), 1485 (w), 1409 (m), 1247 (s), 1125 (w), 1098 (m), 878 (w), 834 (s), 808 (s), 788 (m), 749 (m), 730 (m), 706 (m), 692 (w), 649 (m), 495 (w).

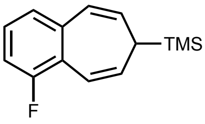


**8f**

**(1-fluoro-7*H*-benzo[7]annulen-7-yl)trimethylsilane (8f):**

Following General Procedure A (0.5 mmol scale) the title compound was isolated by flash chromatography ($SiO_2$, hexanes:ethyl acetate = 100:0 to 50:1) as a yellow oil (94 mg, 81%).

**$R_f$** = 0.4 ($SiO_2$, hexanes:ethyl acetate = 50:1, UV, $KMnO_4$)

**$^1H$ NMR** (500 MHz, $CDCl_3$) δ 6.99 (td, *J* = 7.9, 5.6 Hz, 1H), 6.73 – 6.68 (m, 1H), 6.37 (d, *J* = 11.5 Hz, 1H), 6.14 (d, *J* = 11.4 Hz, 1H), 5.60 (dd, *J* = 11.6, 8.5 Hz, 1H), 5.54 (dd, *J* = 11.4, 8.5 Hz, 1H), 2.37 (t, *J* = 8.4 Hz, 1H), -0.01 (s, 9H).

**$^{13}C$ NMR** (126 MHz, $CDCl_3$) δ 160.17 (d, *J* = 246.6 Hz), 139.92, 134.44, 134.17, 128.03 (d, *J* = 3.4 Hz), 127.66 (d, *J* = 9.7 Hz), 126.22 (d, *J* = 3.1 Hz), 125.71 (d, *J* = 11.9 Hz), 119.32 (d, *J* = 9.4 Hz), 113.13 (d, *J* = 23.6 Hz), 36.87, -2.81.

**$^{19}F$ NMR** (471 MHz, $CDCl_3$) δ -119.31.

**HRMS**: (ESI-TOF, m/z) calcd. for $C_{14}H_{18}FSi$ $[M + H]^+$, 232.1084; found: 232.1085.

**IR:** (ATR, neat, $cm^{-1}$) = 2970 (w), 2953 (w), 1738 (s), 1446 (m), 1421 (m), 1365 (s), 1228 (s), 1217 (s), 836 (m), 823 (s), 806 (s), 782 (m), 758 (m), 731 (m), 540 (m), 527 (w), 513 (m)

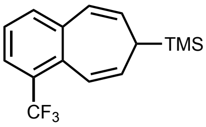


**8g**

**trimethyl(1-(trifluoromethyl)-7*H*-benzo[7]annulen-7-yl)silane (8g):**

Following General Procedure A (0.5 mmol scale) the title compound was isolated by flash chromatography ($SiO_2$, hexanes:ethyl acetate = 100:0 to 50:1) as a yellow oil (103 mg, 73%).

**$R_f$** = 0.4 ($SiO_2$, hexanes:ethyl acetate = 50:1, UV, $KMnO_4$)

**$^1H$ NMR** (500 MHz, $CDCl_3$) δ 7.42 (dd, *J* = 5.7, 3.5 Hz, 1H), 7.15 (q, *J* = 3.3, 2.2 Hz, 2H), 6.54 (dq, *J* = 11.3, 2.5 Hz, 1H), 6.26 (d, *J* = 11.0 Hz, 1H), 5.75 (dddd, *J* = 13.1, 10.1, 8.8, 1.2 Hz, 2H), 2.37 (t, *J* = 8.9 Hz, 1H), -0.10 (s, 9H).

**$^{13}C$ NMR** (126 MHz, $CDCl_3$) δ 139.72, 136.65, 136.62, 136.17, 134.20, 128.38 (q, *J* = 28.7 Hz), 127.89, 125.98, 124.6 (q, *J* = 274,7 Hz), 124.01 (q, *J* = 6.1 Hz), 123.40 (q, *J* = 2.5 Hz), 36.28, -2.46.

**$^{19}F$ NMR** (471 MHz, $CDCl_3$) δ -60.62.

**HRMS**: (ESI-TOF, m/z) calcd. for $C_{15}H_{18}F_3Si$ $[M + H]^+$, 282.1052; found: 282.1047.

**IR:** (ATR, neat, $cm^{-1}$) = 2970 (w), 1467 (w), 1449 (w), 1395 (w), 1311 (s), 1249 (m), 1155 (m), 1117 (s), 1081 (m), 908 (w), 836 (s), 824 (s), 803 (m), 781 (m), 766 (m), 733 (m), 651 (w), 602 (w), 514 (m).

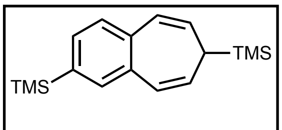


**8h**

**(7*H*-benzo[7]annulene-1,7-diyl)bis(trimethylsilane) (8h):**

Following General Procedure A (0.5 mmol scale) the title compound was isolated by flash chromatography ($SiO_2$, hexanes:ethyl acetate = 100:0 to 50:1) as a yellow oil (114 mg, 81%).

**$R_f$** = 0.4 ($SiO_2$, hexanes:ethyl acetate = 50:1, UV, $KMnO_4$)

**$^1$H NMR** (500 MHz, $CDCl_3$) δ 7.15 (dd, *J* = 7.5, 1.3 Hz, 1H), 7.01 (s, 1H), 6.86 (d, *J* = 7.4 Hz, 1H), 6.08 (dd, *J* = 15.4, 11.5 Hz, 2H), 5.50 – 5.41 (m, 2H), 2.35 (t, *J* = 8.1 Hz, 1H), 0.23 (s, 8H), -0.00 (s, 8H).

**$^{13}$C NMR** (126 MHz, $CDCl_3$) δ 138.51, 138.27, 136.87, 135.88, 133.01, 132.45, 131.68, 129.93, 129.03, 128.76, 37.21, -1.03, -2.91.

**HRMS**: (ESI-TOF, m/z) calcd. for $C_{17}H_{27}Si_2$ $[M + H]^+$, 286.1573; found: 286.1581.

**IR:** (ATR, neat, $cm^{-1}$) = 2970 (w), 1738 (m), 1419 (w), 1365 (s), 1246 (s), 1229 (m), 1217 (s), 1112 (m), 1100 (w), 894 (w), 833 (s), 789 (m), 728 (m), 691 (w), 613 (w), 514 (w).

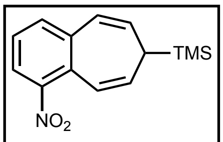


**8i**

**trimethyl(1-nitro-7*H*-benzo[7]annulen-7-yl)silane (8i):**

Following General Procedure A (0.5 mmol scale) the title compound was isolated by flash chromatography ($SiO_2$, hexanes:ethyl acetate = 100:0 to 50:1) as a yellow oil (98 mg, 75%).

**$R_f$** = 0.4 ($SiO_2$, hexanes:ethyl acetate = 50:1, UV, $KMnO_4$)

**$^1$H NMR** (500 MHz, $CDCl_3$) δ 7.40 (dt, *J* = 7.5, 3.8 Hz, 1H), 7.18 – 7.11 (m, 2H), 6.25 (d, *J* = 11.3 Hz, 1H), 6.20 (d, *J* = 11.4 Hz, 1H), 5.79 – 5.69 (m, 2H), 2.41 (t, *J* = 8.8 Hz, 1H), -0.03 (s, 9H).

**$^{13}$C NMR** (126 MHz, $CDCl_3$) δ 150.47, 139.98, 137.53, 137.03, 133.95, 131.49, 127.19, 126.69, 121.59, 121.50, 36.72, -2.50.

**HRMS**: (ESI-TOF, m/z) calcd. for $C_{14}H_{18}NO_2Si$ $[M + H]^+$, 258.0950; found: 258.0944.

**IR:** (ATR, neat, $cm^{-1}$) = 2970 (w), 1522 (s), 1446 (m), 1450 (w), 1351 (m), 1248 (m), 1101 (w), 1063 (w), 1008 (w), 835 (s), 818 (m), 788 (m), 736 (m), 610 (w), 540 (w), 485 (w).

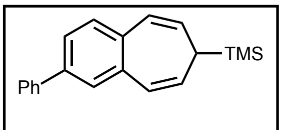


**8j**

**trimethyl(1-phenyl-7*H*-benzo[7]annulen-7-yl)silane (8j):**

Following General Procedure A (0.5 mmol scale) the title compound was isolated by flash chromatography ($SiO_2$, hexanes:ethyl acetate = 100:0 to 50:1) as a yellow oil (112 mg, 77%).

**$R_f$** = 0.4 ($SiO_2$, hexanes:ethyl acetate = 50:1, UV, $KMnO_4$)

**$^1$H NMR** (500 MHz, $CDCl_3$) δ 7.60 – 7.56 (m, 2H), 7.41 (t, *J* = 7.7 Hz, 2H), 7.35 – 7.29 (m, 1H), 7.28 – 7.23 (m, 1H), 7.15 (d, *J* = 2.0 Hz, 1H), 6.96 (d, *J* = 7.9 Hz, 1H), 6.14 (dd, *J* = 19.4, 11.5 Hz, 2H), 5.53 – 5.45 (m, 2H), 2.39 (t, *J* = 8.2 Hz, 1H), 0.02 (s, 9H).

**$^{13}$C NMR** (126 MHz, $CDCl_3$) δ 140.67, 139.11, 138.22, 137.01, 133.03, 132.84, 131.31, 129.42, 128.82, 128.69, 128.29, 127.31, 126.83, 125.12, 37.28, -2.85.

**HRMS**: (ESI-TOF, m/z) calcd. for $C_{20}H_{23}Si$ $[M + H]^+$, 290.1491; found: 290.1497.

**IR:** (ATR, neat, $cm^{-1}$) = 2971 (w), 1738 (m), 1482 (m), 1366 (s), 1355 (s), 1229 (m), 1217 (s), 1112 (m), 831 (s), 791 (m), 756 (s), 693 (w), 650 (w), 486 (w).

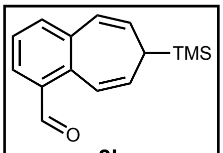


**8k**

**7-(trimethylsilyl)-7*H*-benzo[7]annulene-1-carbaldehyde (8k):**

Following General Procedure A (0.5 mmol scale) the title compound was isolated by flash chromatography ($SiO_2$, hexanes:ethyl acetate = 100:0 to 50:1) as a yellow oil (92 mg, 76%).

**$R_f$** = 0.4 ($SiO_2$, hexanes:ethyl acetate = 50:1, UV, $KMnO_4$)

**$^1$H NMR** (500 MHz, $CDCl_3$) δ 10.34 (s, 1H), 7.58 (dd, *J* = 7.2, 1.9 Hz, 1H), 7.22 – 7.19 (m, 1H), 7.04 (d, *J* = 11.3 Hz, 1H), 6.28 (d, *J* = 11.1 Hz, 1H), 5.83 (ddd, *J* = 11.4, 8.9, 1.2 Hz, 1H), 5.71 (ddd, *J* = 11.2, 8.5, 1.2 Hz, 1H), 2.37 (t, *J* = 8.8 Hz, 1H), -0.07 (s, 10H).

**$^{13}$C NMR** (126 MHz, $CDCl_3$) δ 192.65, 139.88, 139.25, 136.97, 136.23, 136.05, 133.66, 129.92, 128.03, 126.59, 123.32, 36.47, -2.38.

**HRMS**: (ESI-TOF, m/z) calcd. for $C_{15}H_{19}OSi$ $[M + H]^+$, 243.1205; found: 243.1201.

**IR:** (ATR, neat, $cm^{-1}$) = 2955 (w), 1738 (m), 1693 (m), 1595 (w), 1365 (s), 1247 (s), 1229 (s), 1217 (s), 1111 (m), 1051 (m), 862 (s), 834 (s), 789 (m), 728 (m), 691 (w), 613 (w), 514 (w).

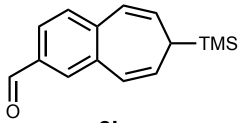


**8l**

**7-(trimethylsilyl)-7*H*-benzo[7]annulene-2-carbaldehyde (8l):**

Following General Procedure A (0.5 mmol scale) the title compound was isolated by flash chromatography ($SiO_2$, hexanes:ethyl acetate = 100:0 to 50:1) as a yellow oil (90 mg, 74%).

**$R_f$** = 0.4 ($SiO_2$, hexanes:ethyl acetate = 50:1, UV, $KMnO_4$)

**$^1$H NMR** (500 MHz, $CD_2Cl_2$) δ 9.85 (s, 1H), 7.49 (dd, $J$ = 7.8, 1.8 Hz, 1H), 7.35 (d, $J$ = 1.8 Hz, 1H), 7.02 (d, $J$ = 7.8 Hz, 1H), 6.13 (dd, $J$ = 11.5, 6.6 Hz, 2H), 5.64 (ddd, $J$ = 11.6, 8.3, 1.5 Hz, 1H), 5.52 (ddd, $J$ = 11.5, 8.2, 1.5 Hz, 1H), 2.45 (t, $J$ = 8.2 Hz, 1H), 0.00 (s, 8H).

$^{13}$C NMR (126 MHz, $CD_2Cl_2$) δ 191.99, 191.97, 191.96, 144.61, 139.13, 137.76, 135.14, 134.95, 132.14, 131.71, 128.30, 128.17, 127.97, 38.55, -2.91.

**HRMS**: (ESI-TOF, m/z) calcd. for $C_{15}H_{19}OSi$ $[M + H]^+$, 243.1205; found: 243.1200.

**IR:** (ATR, neat, $cm^{-1}$) = 2950 (w), 1740 (m), 1700 (m), 1593 (w), 1362 (s), 1250 (s), 1230 (s), 1216 (s), 1116 (m), 898 (s), 836 (s), 789 (m), 728 (m), 691 (w), 613 (w), 514 (w).

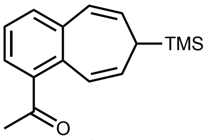


**8m**

**1-(7-(trimethylsilyl)-7*H*-benzo[7]annulen-1-yl)ethan-1-one (8m):**

Following General Procedure A (0.5 mmol scale) the title compound was isolated by flash chromatography ($SiO_2$, hexanes:ethyl acetate = 100:0 to 50:1) as a yellow oil (83 mg, 65%).

**$R_f$** = 0.4 ($SiO_2$, hexanes:ethyl acetate = 50:1, UV, $KMnO_4$)

**$^1$H NMR** (500 MHz, $CDCl_3$) δ 7.18 (dd, $J$ = 7.5, 1.5 Hz, 1H), 7.10 (t, $J$ = 7.6 Hz, 1H), 7.05 (dd, $J$ = 7.7, 1.5 Hz, 1H), 6.31 (d, $J$ = 11.4 Hz, 1H), 6.24 (d, $J$ = 11.2 Hz, 1H), 5.70 – 5.61 (m, 2H), 2.53 (s, 3H), 2.34 (t, $J$ = 8.7 Hz, 1H), -0.04 (s, 9H).

**$^{13}$C NMR** (126 MHz, $CDCl_3$) δ 204.65, 141.02, 138.85, 135.70, 135.51, 134.45, 133.01, 128.31, 126.25, 125.61, 125.33, 36.24, 31.19, -2.43.

**HRMS**: (ESI-TOF, m/z) calcd. for $C_{16}H_{21}OSi$ $[M + H]^+$, 257.1362; found: 257.1362.

**IR:** (ATR, neat, $cm^{-1}$) = 2955 (w), 1738 (m), 1678 (s), 1595 (m), 1355 (m), 1272 (s), 1247 (m), 1225 (m) 1218 (m), 1192 (m), 1125 (m), 1081 (w), 976 (w), 862 (s), 834 (s), 789 (m), 728 (m), 698 (w), 613 (w), 487 (w).

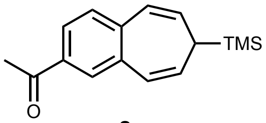


**8n**

**1-(7-(trimethylsilyl)-7*H*-benzo[7]annulen-2-yl)ethan-1-one (8n):**

Following General Procedure A (0.5 mmol scale) the title compound was isolated by flash chromatography ($SiO_2$, hexanes:ethyl acetate = 100:0 to 50:1) as a yellow oil (82 mg, 64%).

**$R_f$** = 0.4 ($SiO_2$, hexanes:ethyl acetate = 50:1, UV, $KMnO_4$)

$^1$H NMR (500 MHz, $CDCl_3$) δ 7.57 (dt, $J$ = 8.0, 1.4 Hz, 1H), 7.46 (d, $J$ = 1.9 Hz, 1H), 6.94 (d, $J$ = 7.9 Hz, 1H), 6.11 (dd, $J$ = 11.5, 8.9 Hz, 2H), 5.62 – 5.55 (m, 1H), 5.48 (dd, $J$ = 11.6, 8.3 Hz, 1H), 2.54 (d, $J$ = 1.0 Hz, 3H), 2.41 (t, $J$ = 8.2 Hz, 1H), -0.00 (d, $J$ = 1.0 Hz, 9H).

**$^{13}$C NMR** (126 MHz, $CDCl_3$) δ 197.69, 142.79, 138.08, 136.36, 135.14, 133.88, 130.90, 130.69, 128.08, 127.90, 126.81, 37.90, 26.63, -2.92.

**HRMS**: (ESI-TOF, m/z) calcd. for $C_{16}H_{21}OSi$ $[M + H]^+$, 257.1362; found: 257.1355.

**IR:** (ATR, neat, $cm^{-1}$) = 2960 (w), 1740 (m), 1682 (s), 1590 (m), 1350 (m), 1281 (s), 1255 (m), 1250 (m) 1212 (m), 1191 (m), 1122 (m), 1084 (w), 981 (w), 866 (s), 835 (s), 789 (m), 725 (m), 690 (w), 615 (w), 487 (w).

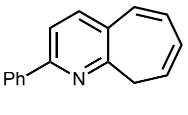


**11a**

**2-phenyl-9*H*-cyclohepta[*b*]pyridine (11a):**

Following General Procedure A (0.5 mmol scale) the title compound was isolated by flash chromatography ($SiO_2$, hexanes:ethyl acetate = 100:0 to 50:1) as a yellow oil (50 mg, 46%).

**$R_f$** = 0.4 ($SiO_2$, hexanes:ethyl acetate = 50:1, UV, $KMnO_4$)

**$^1$H NMR** (500 MHz, $CDCl_3$) δ 8.04 – 7.99 (m, 2H), 7.70 (d, *J* = 8.0 Hz, 1H), 7.61 (d, *J* = 8.0 Hz, 1H), 7.50 – 7.44 (m, 2H), 7.44 – 7.37 (m, 1H), 6.99 (d, *J* = 11.3 Hz, 1H), 6.59 (dd, *J* = 11.3, 5.5 Hz, 1H), 6.21 (dd, *J* = 9.8, 5.4 Hz, 1H), 5.90 (dt, *J* = 9.8, 6.9 Hz, 1H), 3.39 (d, *J* = 6.9 Hz, 2H).

**$^{13}$C NMR** (126 MHz, $CDCl_3$) δ 157.55, 153.14, 139.42, 136.39, 130.20, 129.72, 129.32, 128.98, 128.90, 128.88, 127.07, 126.62, 126.24, 118.08, 38.05.

**HRMS**: (ESI-TOF, m/z) calcd. for $C_{16}H_{14}N$ $[M + H]^+$, 220.1126; found: 220.1124.

**IR:** (ATR, neat, $cm^{-1}$) = 3016 (w), 2970 (w), 1738 (s), 1579 (w), 1447 (m), 1455 (m), 1366 (s), 1228 (s), 1217 (s), 761 (w), 727 (w), 712 (w), 691 (m), 653 (w).

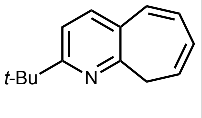


**11b**

**2-(*tert*-butyl)-9*H*-cyclohepta[*b*]pyridine (11b):**

Following General Procedure A (0.5 mmol scale) the title compound was isolated by flash chromatography ($SiO_2$, hexanes:ethyl acetate = 100:0 to 50:1) as a yellow oil (41 mg, 41%).

**$R_f$** = 0.4 ($SiO_2$, hexanes:ethyl acetate = 50:1, UV, $KMnO_4$)

**$^1$H NMR** (500 MHz, $CDCl_3$) δ 7.54 (d, *J* = 8.1 Hz, 1H), 7.20 (d, *J* = 8.0 Hz, 1H), 6.93 (d, *J* = 11.3 Hz, 1H), 6.51 (dd, *J* = 11.4, 5.4 Hz, 1H), 6.16 (dd, *J* = 9.8, 5.4 Hz, 1H), 5.86 (dt, *J* = 10.0, 6.9 Hz, 1H), 3.27 (d, *J* = 6.9 Hz, 2H), 1.35 (s, 9H).

**$^{13}$C NMR** (126 MHz, $CDCl_3$) δ 169.69, 152.01, 135.70, 130.54, 128.87, 127.72, 126.65, 125.99, 116.43, 38.14, 37.40, 30.35, 29.86.

**HRMS**: (ESI-TOF, m/z) calcd. for $C_{14}H_{18}N$ $[M + H]^+$, 200.1439; found: 200.1439.

**IR:** (ATR, neat, $cm^{-1}$) = 3016 (w), 2970 (w), 1738 (s), 1586 (w), 1456 (m), 1427 (m), 1365 (s), 1228 (s), 1217 (s), 1205 (s), 1143 (w), 1092 (w), 834 (w), 671 (w).

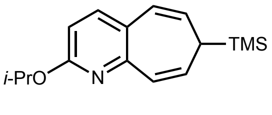


**11c**

**2-isopropoxy-7-(trimethylsilyl)-7*H*-cyclohepta[*b*]pyridine (11c):**

Following General Procedure B (0.5 mmol scale) the title compound was isolated by flash chromatography ($SiO_2$, hexanes:ethyl acetate = 100:0 to 50:1) as a yellow oil (93 mg, 68%).

**$R_f$** = 0.4 ($SiO_2$, hexanes:ethyl acetate = 50:1, UV, $KMnO_4$)

**$^1$H NMR** (500 MHz, $CDCl_3$) δ 7.08 (d, *J* = 8.3 Hz, 1H), 6.33 (d, *J* = 8.3 Hz, 1H), 6.19 (d, *J* = 11.6 Hz, 1H), 5.96 (d, *J* = 11.2 Hz, 1H), 5.65 (ddd, *J* = 11.3, 8.6, 1.3 Hz, 1H), 5.37 (ddd, *J* = 11.0, 8.3, 1.3 Hz, 1H), 5.29 – 5.20 (m, *J* = 6.2 Hz, 1H), 2.36 (t, *J* = 8.4 Hz, 1H), 1.31 (dd, *J* = 6.2, 3.4 Hz, 7H), -0.02 (s, 10H).

**$^{13}$C NMR** (126 MHz, $CDCl_3$) δ 160.83, 154.11, 140.64, 135.64, 130.69, 130.45, 127.36, 125.75, 108.31, 67.82, 36.67, 22.20, 22.14, -2.91.

**HRMS**: (ESI-TOF, m/z) calcd. for $C_{16}H_{24}NOSi$ $[M + H]^+$, 274.1627; found: 274.1626.

**IR:** (ATR, neat, $cm^{-1}$) = 3015 (w), 1587 (s), 1558 (m), 1458 (s), 1381 (w), 1370 (w), 1263 (s), 1248 (s), 1139 (w), 1101 (s), 988 (m), 862 (s), 833 (s), 806 (m), 751 (m), 732 (m), 693 (w).

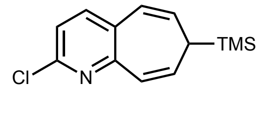


**11d**

**2-chloro-7-(trimethylsilyl)-7*H*-cyclohepta[*b*]pyridine (11d):**

Following General Procedure B (0.5 mmol scale) the title compound was isolated by flash chromatography ($SiO_2$, hexanes:ethyl acetate = 100:0 to 50:1) as a yellow oil (62 mg, 50%).

**$R_f$** = 0.4 ($SiO_2$, hexanes:ethyl acetate = 50:1, UV, $KMnO_4$)

**$^1$H NMR** (500 MHz, $CDCl_3$) δ 7.09 (d, *J* = 8.0 Hz, 1H), 6.91 (d, *J* = 8.0 Hz, 1H), 6.23 (d, *J* = 11.8 Hz, 1H), 5.95 (d, *J* = 11.4 Hz, 1H), 5.71 (ddd, *J* = 11.7, 8.5, 1.4 Hz, 1H), 5.51 (ddd, *J* = 11.4, 8.3, 1.4 Hz, 1H), 2.46 (t, *J* = 8.4 Hz, 1H), 0.00 (s, 9H).

**$^{13}$C NMR** (126 MHz, $CDCl_3$) δ 157.16, 147.66, 140.04, 138.39, 135.08, 133.26, 129.58, 124.68, 121.28, 37.99, -3.00.

**HRMS**: (ESI-TOF, m/z) calcd. for $C_{13}H_{17}ClNSi$ $[M + H]^+$, 250.0819; found: 250.0814.

**IR:** (ATR, neat, $cm^{-1}$) = 2968 (w), 1573 (m), 1548 (m), 1438 (s), 1423 (m), 1249 (s), 1178 (w), 1146 (w), 1132 (w), 1109 (m), 1080 (w), 925 (m), 862 (s), 835 (s), 808 (m), 761 (s), 735 (w), 699 (w).

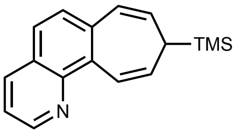


**11e**

**9-(trimethylsilyl)-9*H*-cyclohepta[*h*]quinoline (11e):**

Following General Procedure B (0.5 mmol scale) the title compound was isolated by flash chromatography ($SiO_2$, hexanes:ethyl acetate = 100:0 to 50:1) as a yellow oil (89 mg, 67%).

**$R_f$** = 0.4 ($SiO_2$, hexanes:ethyl acetate = 50:1, UV, $KMnO_4$)

**$^1$H NMR** (500 MHz, $CDCl_3$) δ 8.89 (dd, *J* = 4.1, 1.9 Hz, 1H), 8.02 (dd, *J* = 8.1, 1.8 Hz, 1H), 7.60 (d, *J* = 11.1 Hz, 1H), 7.54 (d, *J* = 8.5 Hz, 1H), 7.33 (dd, *J* = 8.1, 4.2 Hz, 1H), 7.22 (d, *J* = 8.4 Hz, 1H), 6.40 (d, *J* = 10.8 Hz, 1H), 5.77 (dddd, *J* = 29.1, 10.8, 8.5, 1.2 Hz, 2H), 2.30 (t, *J* = 8.5 Hz, 1H), -0.09 (s, 9H).

**$^{13}$C NMR** (126 MHz, $CDCl_3$) δ 149.39, 146.61, 138.84, 136.08, 135.56, 135.42, 132.54, 129.75, 128.66, 126.64, 125.96, 124.44, 120.84, 35.34, -2.59.

**HRMS**: (ESI-TOF, m/z) calcd. for $C_{17}H_{20}NSi$ $[M + H]^+$, 266.1365; found: 266.1359.

**IR:** (ATR, neat, $cm^{-1}$) = 2952 (w), 1599 (m), 1496 (w), 1453 (w), 1411 (w), 1372 (w), 1247 (s), 1134 (w), 1002 (w), 976 (w), 889 (w), 864 (s), 835 (s), 771 (m), 745 (m), 647 (w).

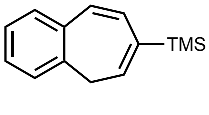


**12**

**(5*H*-benzo[7]annulen-7-yl)trimethylsilane (12):**

To a stirred solution of benzocycloheptatriene **8a** (50 mg, 0.23 mmol, 1.0 equiv.) dissolved in dry DCM (2.3 mL, 0.1 M) at -78 °C, was added $BF_3{\cdot}Et_2O$ (0.086 mL, 0.70 mmol, 3 equiv.) dropwise. After 2 h of stirring at -78 °C the reaction was quenched with water (5 mL), extracted with DCM (3 x 5 mL), dried over $MgSO_4$, filtered, and concentrated under reduced pressure. The resulting crude mixture was dry-loaded onto celite and isolated by flash chromatography ($SiO_2$, hexanes: ethyl acetate = 50:1) to give the desired compounds as a colorless liquid (30 mg, 60%).

**$R_f$** = 0.3 ($SiO_2$, hexanes:ethyl acetate = 50:1, UV, $KMnO_4$)

**$^1$H NMR** (500 MHz, $CDCl_3$) δ 7.33 (ddd, *J* = 13.2, 7.5, 1.4 Hz, 2H), 7.22 (td, *J* = 7.5, 1.3 Hz, 1H), 7.18 (dd, *J* = 7.5, 1.3 Hz, 1H), 7.07 (d, *J* = 11.3 Hz, 1H), 6.63 (d, *J* = 11.3 Hz, 1H), 5.97 (t, *J* = 6.6 Hz, 1H), 3.03 (d, *J* = 6.6 Hz, 2H), 0.09 (s, 8H).

**$^{13}$C NMR** (126 MHz, $CDCl_3$) δ 138.26, 136.68, 136.30, 133.88, 131.77, 131.72, 128.50, 128.07, 127.38, 125.51, 36.49, -1.36.

**HRMS**: (ESI-TOF, m/z) calcd. For $C_{14}H_{18}Si$ $[M + H]^+$, 214.1178; found: 214.1181.

**IR:** (ATR, neat, $cm^{-1}$) = 2970 (w), 1738 (s), 1435 (w), 1365 (s), 1228 (s), 1216 (s), 1206 (s), 1092 (w), 894 (w), 835 (w), 788 (w), 746 (w), 696 (m), 654 (m).

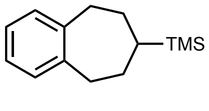


**13**

**trimethyl(6,7,8,9-tetrahydro-5*H*-benzo[7]annulen-7-yl)silane (13):**

Benzocycloheptatriene **8a** (22 mg, 0.1 mmol) was charged into a 4 mL vial and methanol (1.0 mL, 0.1 M) was added. The solution was sparged with nitrogen under sonication for 15 minutes, followed by the addition of Pd/C (8 mg, 5 mol%). And sparged for an additional 10 minutes. The reaction mixture was sparged with hydrogen (1 atm) for 3 minutes and left to stir overnight. The reaction was filtered through celite and concentrated under reduced pressure to yield the desired compound as a colorless oil (21 mg, 94%).

**$R_f$** = 0.3 ($SiO_2$, hexanes:ethyl acetate = 50:1, UV, $KmnO_4$)

**$^1$H NMR** (500 MHz, $CDCl_3$) δ 7.10 (s, 4H), 2.95 – 2.79 (m, 5H), 1.98 (dddd, *J* = 13.7, 6.8, 2.9, 1.5 Hz, 2H), 1.28 – 1.16 (m, 3H), 0.99 (tt, *J* = 12.7, 2.8 Hz, 1H), -0.05 (s, 9H).

**$^{13}$C NMR** (126 MHz, $CDCl_3$) δ 143.74, 128.97, 126.00, 38.87, 32.84, 28.70, -3.20.

**HRMS**: (ESI-TOF, m/z) calcd. For $C_{14}H_{22}Si$ $[M + H]^+$, 218.1491; found: 218.1493.

**IR:** (ATR, neat, $cm^{-1}$) = 3016 (w), 2970 (w), 2910 (w), 1738 (s), 1448 (m), 1365 (s), 1246 (s), 1228 (m), 1217 (s), 1206 (s), 940 (w), 859 (m), 832 (m), 747 (m), 696 (m), 527 (w).

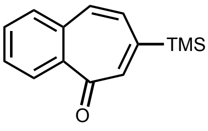


**14**

**7-(trimethylsilyl)-5*H*-benzo[7]annulen-5-one (14):**

To a stirring solution of $CrO_3$ (319 mg, 3.19 mmol, 6 equiv.) in pyridine: DCM (4 ml, 1:1) at 0 °C was added benzocycloheptatriene **8a** (114 mg, 0.532 mmol. 1 equiv.) dissolved in DCM (0.8 mL) and the resulting mixture was allowed to warm to room temperature and stirred overnight until complete conversion as judged by TLC. The reagents were quenched with a 10% aqueous solution of $Na_2S_2O_3$ (5 mL), and the resulting solution was stirred for 30 min. The aqueous phase was extracted with DCM (3 × 5 mL), dried over $MgSO_4$, filtered, and concentrated under reduced pressure. The resulting crude mixture was dry-loaded onto celite and isolated by flash chromatography ($SiO_2$, hexanes: ethyl acetate = 20:1) to give the desired compounds as a brown oil (85 mg, 70%).

**$R_f$** = 0.3 ($SiO_2$, hexanes: ethyl acetate = 20:1, UV, $KMnO_4$)

**$^1$H NMR** (500 MHz, $CDCl_3$) δ 8.02 (dd, *J* = 7.9, 1.5 Hz, 1H), 7.32 – 7.20 (m, 3H), 6.87 (d, *J* = 11.4 Hz, 1H), 6.74 (s, 1H), 6.41 (d, *J* = 11.5 Hz, 1H), -0.10 (s, 10H).

**$^{13}$C NMR** (126 MHz, $CDCl_3$) δ 188.69, 150.33, 141.62, 139.07, 137.67, 135.99, 133.24, 132.33, 130.66, 130.28, 129.91, -1.62.

**HRMS**: (ESI-TOF, m/z) calcd. for $C_{14}H_{17}OSi$ $[M + H]^+$, 229.1049; found: 229.1046.

**IR:** (ATR, neat, $cm^{-1}$) = 2954 (w), 1738 (s), 1625 (m), 1584 (s), 1427 (w), 1366 (m), 1338 (m), 1304 (m), 1249 (s), 1229 (m), 1217 (s), 1206 (s), 989 (s), 840 (s), 822 (s), 778 (m), 729 (w).

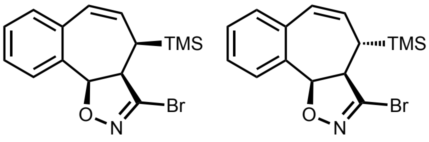


1.1:1

**15a** **15b**

**(3a*S**,4*S**,10b*R**)-3-bromo-4-(trimethylsilyl)-3a,10b-dihydro-4*H*-benzo[3,4]cyclohepta[1,2-*d*]isoxazole (15a) and (3a*S*,4*R*,10b*R*)-3-bromo-4-(trimethylsilyl)-3a,10b-dihydro-4*H*-benzo[3,4]cyclohepta[1,2-*d*]isoxazole (15b):**

A 4 mL vial was charged with benzocycloheptatriene **8a** (22 mg, 0.1 mmol, 1.0 equiv.), hydroxycarbonimidic dibromide (28 mg, 1.5 equiv., 0.15 mmol), potassium carbonate (39 mg, 3.0 equiv., 0.3 mmol), and acetonitrile (1 mL). The reaction mixture was left to stir at room temperature overnight. Upon completion, the reaction was partitioned with $H_2O$ (3 mL) and ethyl acetate (3 mL). The aqueous phase was extracted with ethyl acetate (3 × 3 mL), dried with $MgSO_4$, and concentrated under reduced pressure. The $^1$H NMR of the crude reaction mixture revealed 1:1 mixture of diastereisomers **15a** and **15b**. The resulting crude mixture was dry-loaded onto celite and isolated by flash chromatography ($SiO_2$, hexanes: ethyl acetate = 1:0 to 5:1) to give the desired compounds as colorless solids (14 + 13 mg, 80% combined yield).

**$R_f$** = 0.3, 0.25 ($SiO_2$, hexanes:ethyl acetate = 9:1, UV, $KMnO_4$)

**$^1$H NMR 15a** (500 MHz, $CDCl_3$) δ 7.34 (ddt, *J* = 8.7, 6.8, 3.4 Hz, 1H), 7.26 (d, *J* = 3.4 Hz, 1H), 7.22 (q, *J* = 2.8 Hz, 2H), 7.15 (dd, *J* = 7.8, 3.8 Hz, 1H), 6.48 (dt, *J* = 11.2, 2.9 Hz, 1H), 6.21 (tt, *J* = 11.5, 3.0 Hz, 1H), 5.45 (dt, *J* = 10.7, 3.2 Hz, 1H), 4.55 (dt, *J* = 10.9, 2.9 Hz, 1H), 2.25 – 2.19 (m, 1H), -0.26 (t, *J* = 3.0 Hz, 9H).

**$^{13}$C NMR 15a** (126 MHz, $CDCl_3$) δ 140.93, 139.38, 133.25, 133.13, 131.99, 130.15, 128.68, 128.54, 127.14, 97.05, 60.68, 37.05, -1.39.

**$^1$H NMR 15b** (500 MHz, $CDCl_3$) δ 7.32 (td, *J* = 7.5, 1.4 Hz, 1H), 7.27 (d, *J* = 7.0 Hz, 2H), 7.18 (dd, *J* = 8.2, 6.8 Hz, 1H), 7.12 (d, *J* = 7.6 Hz, 1H), 6.50 (d, *J* = 11.3 Hz, 1H), 6.04 (dd, *J* = 11.3, 9.6 Hz, 1H), 5.48 (d, *J* = 10.8 Hz, 1H), 4.00 (dd, *J* = 10.8, 1.8 Hz, 1H), 2.04 (dd, *J* = 9.6, 1.6 Hz, 1H), -0.26 (d, *J* = 0.9 Hz, 9H).

**$^{13}$C NMR 15b** (126 MHz, $CDCl_3$) δ 144.71, 139.77, 134.26, 132.52, 130.91, 130.88, 130.78, 129.75, 127.09, 87.62, 66.81, 30.33, -1.41.

**HRMS15a**: (ESI-TOF, m/z) calcd. for $C_{15}H_{19}BrNOSi$ $[M + H]^+$, 336.0419; found: 336.0415.

**HRMS15b**: (ESI-TOF, m/z) calcd. for $C_{15}H_{19}BrNOSi$ $[M + H]^+$, 336.0419; found: 336.0421.

**IR 15a:** (ATR, neat, $cm^{-1}$) = 2966 (w), 1738 (s), 1448 (m), 1366 (m), 1249 (s), 1230 (m), 1216 (m), 1209 (m), 881 (m), 872 (m), 834 (s), 783 (s), 749 (s), 531 (w).

**IR 15b:** (ATR, neat, $cm^{-1}$) = 2968 (w), 1743 (s), 1449 (m), 1366 (m), 1253 (s), 1229 (m), 1217 (m), 1209 (m), 880 (m), 870 (m), 834 (s), 783 (s), 749 (s), 531 (w).

**m.p. 15a** = 182 – 184 °C

**m.p. 15b** = 179 – 182 °C

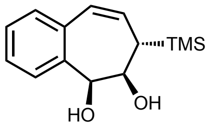


16

**(5*S**,6*S**,7*S**)-7-(trimethylsilyl)-6,7-dihydro-5*H*-benzo[7]annulene-5,6-diol (16):**

To a stirred solution of benzocycloheptatriene **8a** (200 mg, 0.98 mmol, 1.0 equiv.) and NMO (115 mg, 0.98 mmol, 1.0 equiv.), in acetone:$H_2O$ (10 mL, 4:1) at 25 °C was added $OsO_4$ (240 $\mu$L, 0.2 M in MeCN, 0.05 mmol, 5.0 mol%) and the resulting mixture was stirred overnight until complete conversion as judged by TLC. The reagents were quenched with a 10% aqueous solution of $Na_2S_2O_3$ (5 mL), and the resulting solution was stirred for 30 min. The aqueous phase was extracted with DCM (3 × 5 mL), dried over $MgSO_4$, filtered, and concentrated under reduced pressure. The resulting crude mixture was dry-loaded onto celite and isolated by flash chromatography ($SiO_2$, hexanes: ethyl acetate = 7:3) to give the desired compounds as a brown oil (211 mg, 91%).

**$R_f$** = 0.25 ($SiO_2$, hexanes:ethyl acetate = 7:3, UV, $KMnO_4$)

**$^1$H NMR** (500 MHz, $CDCl_3$) δ 7.25 (d, *J* = 7.5 Hz, 1H), 7.12 (t, *J* = 7.4 Hz, 1H), 7.07 (t, *J* = 7.4 Hz, 1H), 7.03 (d, *J* = 7.5 Hz, 1H), 6.27 (dd, *J* = 12.0, 2.1 Hz, 1H), 5.68 (dd, *J* = 12.0, 5.7 Hz, 1H), 4.67 (s, 1H), 4.03 (d, *J* = 6.8 Hz, 1H), 3.24 – 2.87 (m, 1H), 2.53 (s, 1H), 2.00 – 1.80 (m, 1H), -0.07 (s, 10H).

**$^{13}$C NMR** (126 MHz, $CDCl_3$) δ 137.40, 134.48, 130.72, 130.54, 128.68, 127.83, 126.94, 79.01, 76.57, 39.18, -2.21.

**HRMS**: (ESI-TOF, m/z) calcd. For $C_{14}H_{20}O_2NaSi$ $[M + Na]^+$, 271.1130; found: 271.1124.

**IR:** (ATR, neat, $cm^{-1}$) = 3266 (m), 1360 (m), 1247 (s), 1090 (m), 1063 (m), 1048 (m), 988 (m), 888 (m), 875 (m), 833 (s), 785 (m), 747 (m), 696 (m), 527 (w) 485 (m).

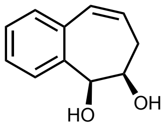


17

**(5*S**,6*R**)-6,7-dihydro-5*H*-benzo[7]annulene-5,6-diol (17):**

To a stirred solution of diol **16** (54 mg, 0.22 mmol, 1 equiv.) dissolved in dry THF (2.2 mL), at -78 °C was added KH (80 mg, 0.65 mmol, 3 equiv., 35% in mineral oil). After 2 h of stirring the reaction was quenched with a 1.0 M solution of HCl (5 mL), ), extracted with DCM (3 x 5 mL), dried over $MgSO_4$, filtered, and concentrated under reduced pressure. The resulting crude mixture was dry-loaded onto celite and isolated by flash chromatography ($SiO_2$, hexanes: ethyl acetate = 7:3 to 1:1) to give the desired compounds as a colorless liquid (21 mg, 55%).

**$R_f$** = 0.25 ($SiO_2$, hexanes:ethyl acetate = 1:1, UV, $KMnO_4$)

**$^1$H NMR** (500 MHz, $CD_3OH$) δ 7.42 (dd, *J* = 7.2, 1.7 Hz, 1H), 7.19 (d, *J* = 1.7 Hz, 0H), 7.16 (dd, *J* = 7.2, 1.8 Hz, 1H), 6.41 (dt, *J* = 11.9, 1.9 Hz, 1H), 5.80 (dt, *J* = 11.9, 5.0 Hz, 1H), 4.77 (d, *J* = 1.7 Hz, 1H), 4.16 (ddd, *J* = 8.5, 6.8, 1.8 Hz, 1H), 2.55 (ddd, *J* = 8.9, 4.6, 2.1 Hz, 2H).

$^{13}$C NMR (126 MHz, $CD_3OH$) δ 139.71, 136.37, 131.96, 130.69, 130.24, 128.58, 127.88, 79.02, 74.64, 35.32.

**HRMS**: (ESI-TOF, m/z) calcd. for $C_{11}H_{13}O_2$ $[M + H]^+$, 177.0916; found: 177.0918.

**IR:** (ATR, neat, $cm^{-1}$) = 2970 (w), 2415 (w), 1738 (s), 1450 (m), 1366 (s), 1229 (s), 1217 (s), 1206 (s), 1036 (m), 976 (m), 954 (m), 921 (w), 782 (m), 761 (m), 701 (m), 630 (m), 507 (m).

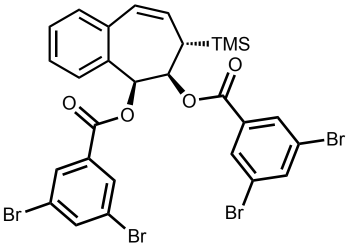


23

**(5*S**,6*S**,7*S**)-7-(trimethylsilyl)-6,7-dihydro-5*H*-benzo[7]annulene-5,6-diyl bis(3,5-dibromobenzoate) (23):**

To a stirred solution of diol **16** (40 mg, 0.16 mmol, 1 equiv.) dissolved in dry DCM (1.6 mL), were added sequentially 3,5-dibromobenzoic acid (110 mg, 0.40 mmol, 2.5 equiv.), 4-DMAP (2.0 mg, 0.016 mmol, 0.1 equiv) and DCC (73 mg, 0.35 mmol, 2.2 equiv.). After 1 h of stirring the reaction was diluted with hexanes (5 mL), washed with water (2 x 2 mL), dried over $MgSO_4$, filtered and concentrated under reduced pressure. The resulting crude mixture was dry-loaded onto celite and isolated by flash chromatography ($SiO_2$, hexanes: ethyl acetate = 10:1 to 9:1) to give the desired compounds as a white solid (108 mg, 87%).

**$R_f$** = 0.25 ($SiO_2$, hexanes:ethyl acetate = 9:1, UV, $KMnO_4$)

**¹H NMR** (500 MHz, $CDCl_3$) δ 8.11 (d, *J* = 1.8 Hz, 2H), 7.87 (d, *J* = 1.9 Hz, 1H), 7.80 (d, *J* = 1.8 Hz, 1H), 7.76 (s, 2H), 7.39 – 7.33 (m, 2H), 7.32 – 7.24 (m, 3H), 6.55 (dd, *J* = 12.4, 2.3 Hz, 1H), 6.36 (s, 1H), 5.91 (dd, *J* = 12.3, 4.8 Hz, 1H), 5.69 (d, *J* = 6.4 Hz, 1H), 2.56 – 2.51 (m, 1H), 0.16 (s, 9H).

**¹³C NMR** (126 MHz, $CDCl_3$) δ 163.32, 163.26, 138.75, 138.54, 133.28, 132.32, 131.74, 131.52, 130.88, 129.10, 128.70, 127.22, 127.10, 123.29, 123.14, -2.14.

**HRMS**: (ESI-TOF, m/z) calcd. for $C_{28}H_{25}Br_4O_4Si$ [M + H]$^+$, 773.1011; found: 773.1009.

**IR:** (ATR, neat, cm$^{-1}$) = 1717 (s), 1557 (m), 1272 (m), 1250 (s), 1139 (m), 1127 (m), 1003 (m), 970 (m), 914 (m), 876 (m), 841 (m), 780 (m), 741 (m), 696 (m), 654 (m).

**m.p.** = 150 – 151 °C

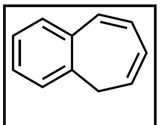

**18**

**5*H*-benzo[7]annulene (18):**

To a solution of KH (650 mg, 5.68 mmol, 6 equiv., 35% in mineral oil) in dry NMP (4.0 mL) was added TBHP (1.032 mL, 5.68 mmol, 6 equiv., 5.0 – 6.0 M in decane) at 0 °C dropwise (caution! Gas evolution). After stirring for 15 minutes at room temperature, a solution of benzocycloheptatriene **8a** (200 mg, 0.95 mmol, 1.0 equiv.) in dry NMP (4.0 mL) was added dropwise. After 1 h of stirring at room temperature, the reaction was quenched with a 10% aqueous solution of $Na_2S_2O_3$ (10 mL) followed by water (10 mL), extracted with DCM (3 x 10 mL), dried over $MgSO_4$, filtered, and concentrated under reduced pressure. The resulting crude mixture was isolated by flash chromatography ($SiO_2$, pentane: diethyl ether = 50:1) to give the desired compounds as a colorless liquid (102 mg, 76%). (caution! The product is slightly volatile).

**R$_f$** = 0.3 ($SiO_2$, pentane: diethyl ether = 50:1, UV, $KMnO_4$)

**¹H NMR** (500 MHz, $CDCl_3$) δ 7.39 – 7.31 (m, 2H), 7.27 – 7.20 (m, 1H), 7.20 – 7.16 (m, 1H), 7.10 (d, *J* = 11.5 Hz, 1H), 6.49 (ddd, *J* = 11.5, 5.4, 1.3 Hz, 1H), 6.13 – 6.06 (m, 1H), 5.81 (dtd, *J* = 10.1, 6.9, 1.3 Hz, 1H), 3.06 (d, *J* = 6.8 Hz, 2H).

**¹³C NMR** (126 MHz, $CDCl_3$) δ 136.57, 136.25, 133.63, 128.91, 128.76, 128.04, 127.65, 127.32, 126.04, 125.70, 34.69.

**HRMS**: (ESI-TOF, m/z) calcd. for $C_{11}H_{11}$ [M + H]$^+$, 143.1105; found: 143.1101.

**IR:** (ATR, neat, cm$^{-1}$) = 3016 (m), 2970 (w), 1738 (s), 1486 (w), 1450 (w), 1433 (w), 1366 (s), 1228 (s), 1217 (s), 1206 (s), 824 (w), 790 (m), 768 (m), 744 (w), 689 (s), 597 (w).

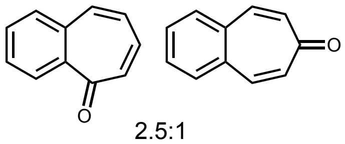

2.5:1

**19** **20**

**5*H*-benzo[7]annulen-5-one (19) and 7*H*-benzo[7]annulen-7-one (20):**

To a stirring solution of $CrO_3$ (220 mg, 2.2 mmol, 6 equiv.) in pyridine:DCM (3.4 ml, 1:1) at 0 °C was added compound **18** (52 mg, 0.37 mmol. 1 equiv.) dissolved in DCM (0.7 mL) and the resulting mixture was allowed to warm to room temperature and stirred overnight until complete conversion as judged by TLC. The reagents were quenched with a 10% aqueous solution of $Na_2S_2O_3$ (5 mL), and the resulting solution was stirred for 30 min. The aqueous phase was extracted with DCM (3 × 5 mL), dried over $MgSO_4$, filtered, and concentrated under reduced pressure. The resulting crude mixture was dry-loaded onto celite and isolated by flash chromatography ($SiO_2$, hexanes: ethyl acetate = 10:1) to give the desired compounds as brown oil (33 mg + 13 mg, 81%).

**R$_f$** = 0.3, 0.1 ($SiO_2$, hexanes: ethyl acetate = 10:1, UV, $KMnO_4$)

**¹H NMR 1** (500 MHz, $CDCl_3$) δ 8.54 – 8.46 (m, 1H), 7.75 – 7.62 (m, 4H), 7.32 (d, *J* = 11.6 Hz, 1H), 7.06 (ddd, *J* = 12.2, 7.8, 1.1 Hz, 1H), 6.95 (dt, *J* = 12.1, 1.1 Hz, 1H), 6.70 (ddd, *J* = 11.6, 7.8, 1.2 Hz, 1H).

**¹³C NMR 1** (126 MHz, $CDCl_3$) δ 188.54, 139.64, 139.08, 136.43, 136.06, 135.61, 134.00, 132.47, 131.01, 130.63, 126.82.

**¹H NMR 2** (500 MHz, $CDCl_3$) δ 7.69 (dt, *J* = 7.4, 3.7 Hz, 2H), 7.60 (dd, *J* = 5.8, 3.4 Hz, 2H), 7.47 (d, *J* = 12.2 Hz, 2H), 6.88 – 6.77 (m, 2H).

**¹³C NMR 2** (126 MHz, $CDCl_3$) δ 188.52, 141.61, 136.15, 135.13, 134.15, 130.62.

**HRMS 1**: (ESI-TOF, m/z) calcd. for $C_{11}H_9O$ [M + H]$^+$, 157.0653; found: 157.0650.

**HRMS 2**: (ESI-TOF, m/z) calcd. for $C_{11}H_9O$ [M + H]$^+$, 157.0653; found: 157.0649.

**IR 1:** (ATR, neat, $cm^{-1}$) = 2970 (w), 1738 (s), 1676 (w), 1639 (w), 1585 (m), 1574 (m), 1467 (w), 1447 (w), 1366 (s), 1308 (w), 1229 (s), 1217 (m), 1207 (s), 1171 (w), 800 (w), 770 (m), 712 (w).

**IR 2:** (ATR, neat, $cm^{-1}$) = 2968 (w), 1740 (s), 1677 (w), 1637 (w), 1586 (m), 1564 (m), 1470 (w), 1435 (w), 1355 (s), 1312 (w), 1232 (s), 1215 (m), 1204 (s), 1173 (w), 822 (w), 772 (m), 732 (w).

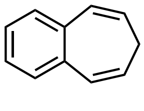

**21**

**7*H*-benzo[7]annulene (21):**

To a solution of KH (367 mg, 8.25 mmol, 18 equiv., 90%) in dry NMP (2.5 mL) was added TBHP (0.5 mL, 2.75 mmol, 6 equiv., 5.0 – 6.0 M in decane) at 0 °C dropwise (caution! Gas evolution). After stirring for 15 minutes at room temperature, a solution of cyclopropane **10a** (150 mg, 0.458 mmol, 1.0 equiv.) in dry NMP (2.0 mL) was added dropwise. Upon completion, the reaction was cooled in an ice bath and water (4.5 mL) was added. AcOH is then carefully added dropwise until pH = 5, upon which gas evolution is observed. $CuCl_2$ dihydrate (4.0 mg, 25 µmol, 5.0 mol%). was then added as a solid, followed by sparging with oxygen, and the reaction was stirred for 10-16 h under an atmosphere of oxygen (balloon). Upon completion, the reaction was partitioned between $CH_2Cl_2$ (10 mL) and saturated brine (10 mL), and the organic layer was extracted with $CH_2Cl_2$ (3 × 10 mL). The resulting crude mixture was isolated by flash chromatography ($SiO_2$, pentane: diethyl ether = 50:1) to give the desired compounds as a colorless liquid (46 mg, 70%). (caution! The product is slightly volatile).

$\mathbf{R_f}$ = 0.3 ($SiO_2$, pentane: diethyl ether = 50:1, UV, $KMnO_4$)

**$^1$H NMR** (500 MHz, $CDCl_3$) δ 7.31 (dt, *J* = 7.3, 3.7 Hz, 1H), 7.26 – 7.21 (m, 3H), 6.60 (d, *J* = 10.3 Hz, 1H), 5.85 (dt, *J* = 10.2, 6.5 Hz, 1H), 2.49 (t, *J* = 6.5 Hz, 1H).

**$^{13}$C NMR** (126 MHz, $CDCl_3$) δ 137.24, 130.61, 130.39, 128.55, 126.17, 26.40.

**HRMS**: (ESI-TOF, m/z) calcd. for $C_{11}H_{11}$ $[M + H]^+$, 143.1105; found: 143.1102.

**IR:** (ATR, neat, $cm^{-1}$) = 3014 (m), 2966 (w), 1738 (s), 1480 (w), 1448 (w), 1435 (w), 1360 (m), 1230 (s), 1216 (s), 1203 (s), 830 (w), 812 (w), 792 (m), 769 (m), 740 (w), 694 (s), 600 (w).

## 3-5. Large-scale procedures:

**Gram-scale synthesis of 10a:**

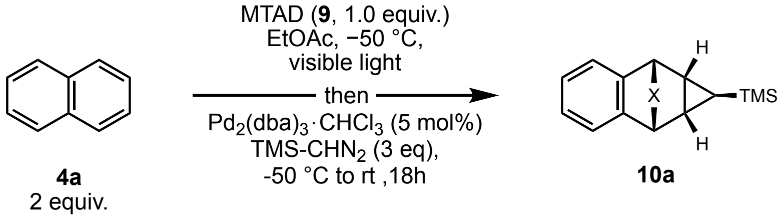


A solution of N-methyl-1,2,4-triazoline-3,5-dione (**9**, MTAD, 1 g, 8.8 mmol, 1.0 equiv.) and arene (**4a**, 2.26 g, 17.6 mmol, 2.0 equiv.) in dry and degassed ethyl acetate (44.0 mL, 0.2 M) was irradiated with LED lights at −50 °C under a nitrogen atmosphere. Upon decolorization, which generally proceeds within 8 – 12 hours with the described setup, a suspension of $Pd_2(dba)_3$·$CHCl_3$ (455.44 mg, 0.44 mmol, 0.05 equiv.) in dry and degassed ethyl acetate (10 mL, sonicated for 1 minute), was added dropwise to the solution, followed by dropwise addition of (trimethylsilyl)diazomethane (2.0 M solution in hexanes, 13.2 mL, 3.0 equiv.). After addition, the cold bath was allowed to slowly warm up from −50 °C to room temperature, over 18 hours. The reaction was quenched by adding acetic acid (3.2 mL, 6 equiv.) and left to stir for 15 minutes. The solution was filtered through celite and concentrated under reduced pressure. The residue was purified by flash chromatography to give the desired compound **10a**. The title compound was isolated by flash chromatography ($SiO_2$, hexanes:ethyl acetate = 9:1 to 4:1) as a yellow solid (2.16 g, 75%).

**Gram-scale synthesis of 8a:**

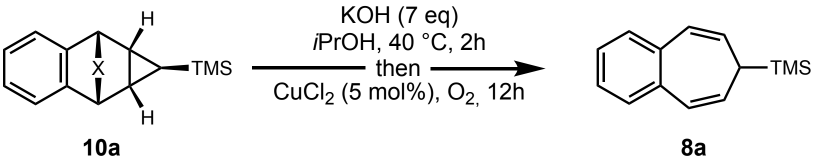


To a vial containing finely ground KOH (770 mg, 5.0 equiv., 90 wt%), and substrate (**10a**, 1 g, 3.05 mmol, 1.0 equiv.) under nitrogen was added *i*-PrOH (30.5 mL, 0.1 M) and degassed with nitrogen/sonication for 15 min. The reaction was heated to 40 °C with vigorous stirring (700 rpm) and progress was monitored by TLC in 30 min intervals. Upon completion, the reaction was cooled in an ice bath and $H_2O$ (30.5 mL) was added. AcOH is then carefully added dropwise until pH = 5, upon which gas evolution is observed. $CuCl_2$ dihydrate (26.0 mg, 152.5 μmol, 5.0 mol%). was then added as a solid, followed by sparging with oxygen, and the reaction was stirred for 12 h under an atmosphere of oxygen (balloon). Upon completion, the reaction was partitioned between $CH_2Cl_2$ (30 mL) and saturated brine (30 mL) and the organic layer was extracted with $CH_2Cl_2$ (3 × 30 mL). The combined organics were washed with brine, dried over $MgSO_4$, filtered, and concentrated under vacuum. The title compound was isolated by flash chromatography ($SiO_2$, hexanes:ethyl acetate = 100:0 to 50:1) as a yellow oil (458 mg, 70%).

## 4. Crystallographic data

X-ray diffraction experiments were carried out on single crystals mounted on Cryo-loops using Paratone-N or Krytox oils. The data was collected on Bruker D8 Venture / Photon II or on a Bruker APEX II diffractometers in George L. Clark X-ray Facility at UIUC. Multi-scan absorption correction was applied. The space group was determined in XPREP (Bruker AXS). The solutions were obtained using Intrinsic Phasing method, as implemented in SHELXT,[12] and refined using full-matrix least squares against $F^2$ , as implemented in SHELXL,[13] using OLEX2[14] as the graphical user interface.

**Crystallographic data for compound 10a**

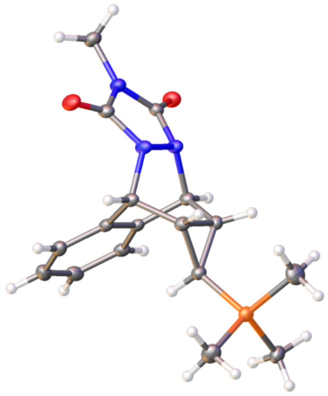

**Table 10a Crystal data and structure refinement.**

| | |
|---|---|
| CCDC | 2129869 |
| Empirical formula | $C_{17}H_{21}N_3O_2Si$ |
| Formula weight | 327.46 |
| Temperature/K | 99.99 |
| Crystal system | monoclinic |
| Space group | $P2_1/c$ |
| a/Å | 26.2536(4) |
| b/Å | 11.6384(2) |
| c/Å | 29.4236(4) |
| α/° | 90 |
| β/° | 107.01 |
| γ/° | 90 |
| Volume/Å$^3$ | 8596.9(2) |
| Z | 20 |
| $\rho_{calc}$g/cm$^3$ | 1.265 |
| μ/mm$^{-1}$ | 0.149 |
| F(000) | 3480.0 |
| Crystal size/mm$^3$ | 0.631 × 0.48 × 0.372 |
| Radiation | MoKα (λ = 0.71073) |
| 2Θ range for data collection/° | 4.284 to 56.596 |
| Index ranges | -34 ≤ h ≤ 35, -15 ≤ k ≤ 15, -39 ≤ l ≤ 37 |
| Reflections collected | 278930 |
| Independent reflections | 21341 [$R_{int}$ = 0.0513, $R_{sigma}$ = 0.0202] |
| Data/restraints/parameters | 21341/18/1056 |
| Goodness-of-fit on $F^2$ | 1.024 |
| Final R indexes [I>=2σ (I)] | $R_1$ = 0.0343, $wR_2$ = 0.0876 |
| Final R indexes [all data] | $R_1$ = 0.0422, $wR_2$ = 0.0940 |
| Largest diff. peak/hole / e Å$^{-3}$ | 0.35/-0.27 |

**Crystallographic data for compound 23**

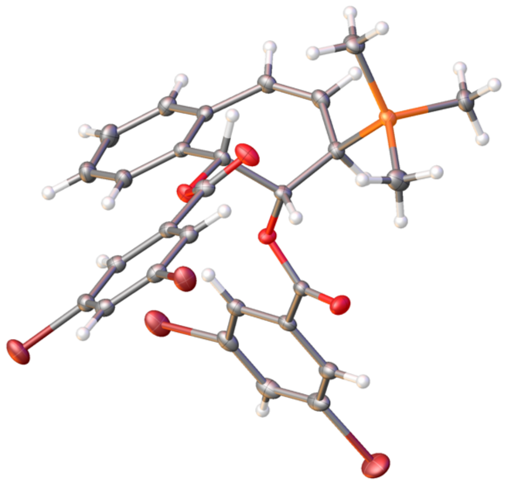

**Table 23 Crystal data and structure refinement.**

| | |
|---|---|
| CCDC | 2129868 |
| Empirical formula | $C_{28}H_{24}Br_4O_4Si$ |
| Formula weight | 772.20 |
| Temperature/K | 100.00 |
| Crystal system | monoclinic |
| Space group | C2/c |
| a/Å | 38.408(2) |
| b/Å | 9.4430(4) |
| c/Å | 16.6160(8) |
| α/° | 90 |
| β/° | 108.776(2) |
| γ/° | 90 |
| Volume/Å$^3$ | 5705.7(5) |
| Z | 8 |
| $\rho_{calc}$g/cm$^3$ | 1.798 |
| μ/mm$^{-1}$ | 5.717 |
| F(000) | 3024.0 |
| Crystal size/mm$^3$ | 0.306 × 0.264 × 0.172 |
| Radiation | MoKα (λ = 0.71073) |
| 2Θ range for data collection/° | 4.456 to 56.592 |
| Index ranges | -51 ≤ h ≤ 47, -12 ≤ k ≤ 12, -22 ≤ l ≤ 22 |
| Reflections collected | 87936 |
| Independent reflections | 7099 [$R_{int}$ = 0.0340, $R_{sigma}$ = 0.0150] |
| Data/restraints/parameters | 7099/0/338 |
| Goodness-of-fit on F$^2$ | 1.027 |
| Final R indexes [I>=2σ (I)] | $R_1$ = 0.0187, w$R_2$ = 0.0447 |
| Final R indexes [all data] | $R_1$ = 0.0212, w$R_2$ = 0.0456 |
| Largest diff. peak/hole / e Å$^{-3}$ | 0.59/-0.43 |

## 5. References


[1] G. W. Breton, M. Turlington, *Tetrahedron Lett*. **2014**, 55, 4661.

[2] K. Nakagawa, R. Konaka, T. Nakata, *J. Org. Chem*. **1962,** 27, 1597.

[3] D.M. Vyas, Y. Chiang, T.W. Doyle, *Tetrahedron Lett*. **1984**, 25, 487.

[4] C. Zarate, R. Martin, *J. Am. Chem. Soc.* **2014**, 136, 2236−2239.

[5] R. A. Moretti, J. Du Bois, T. D. P. Stack, *Org. Lett.* **2016**, 18, 2528.

[6] J. R. Gillard, M. J. Newlands, J. N. Bridson, D. Burnell, *J. Can. J. Chem*. **1991**, 69, 1337.

[7] C. G. Kreiter, S. J. Özkar, *Organomet. Chem*. **1977**, 129, C14.

[8] T. J. Maimone, J. Shi, S. Ashida, P. S. Baran, *J. Am. Chem. Soc*. **2009**, 131, 17066.

[9] A. Varela-Fernandez, C. García-Yebra, J. A. Varela, M. A. Esteruelas, C. Saa, *Angew. Chem., Int. Ed*. **2010**, 49, 4278.

[10] G. Ahmed, A. Bohnstedt, H. J. Breslin, J. Burke, M. A. Curry, J. L. Diebold, B. Dorsey, B. J. Dugan, D. Feng, D. E. Gingrich, T. Guo, K.-K. Ho, K. S. Learn, J. G. Lisko, R.-Q. Liu, E. F. Mesaros, K. Milkiewicz, G. R. Ott, J. Parrish, J. P. Theroff, T. V. Thieu, R. Tripathy, T. L. Underiner, J. C. Wagner, L. Weinberg, G. J. Wells, M. You, C. A. Zificsak, PCT Int. Appl. WO 2008051547 February 5, 2008.

[11] T. Haidzinskaya, H. A. Kerchner, J. Liu, M. P. Watson, *Org. Lett*. **2015**, 17, 3857.

[12] G. M. Sheldrick, *Acta Cryst*. **2015**, A71, 3-8.

[13] G. M. Sheldrick, *Acta Cryst*. **2008**, A64, 112-122.

[14] O.V. Dolomanov, L. J. Bourhis, R. J. Gildea, J. A. K. Howard, H. J. Puschmann, *Appl. Cryst*. **2009**, 42, 339-341

## 6. $^{1}H$ and $^{13}C$ NMR spectra

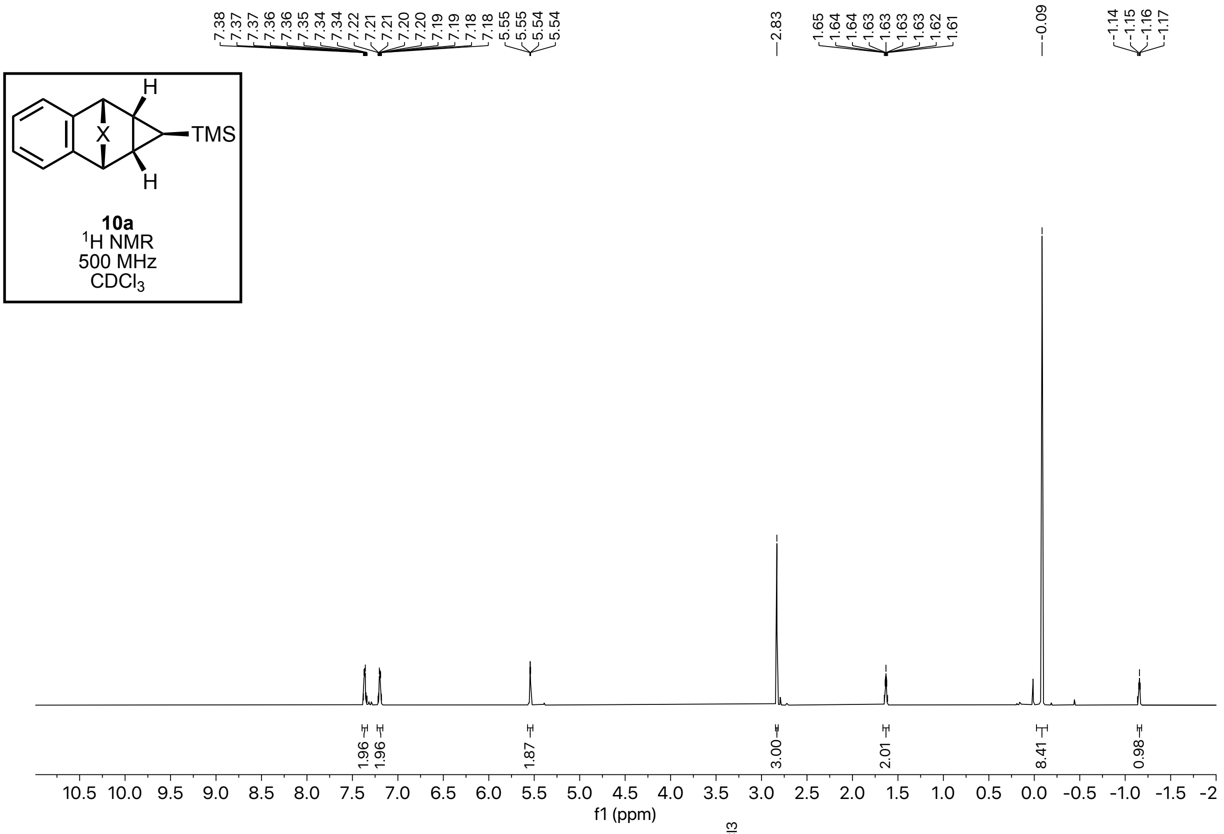


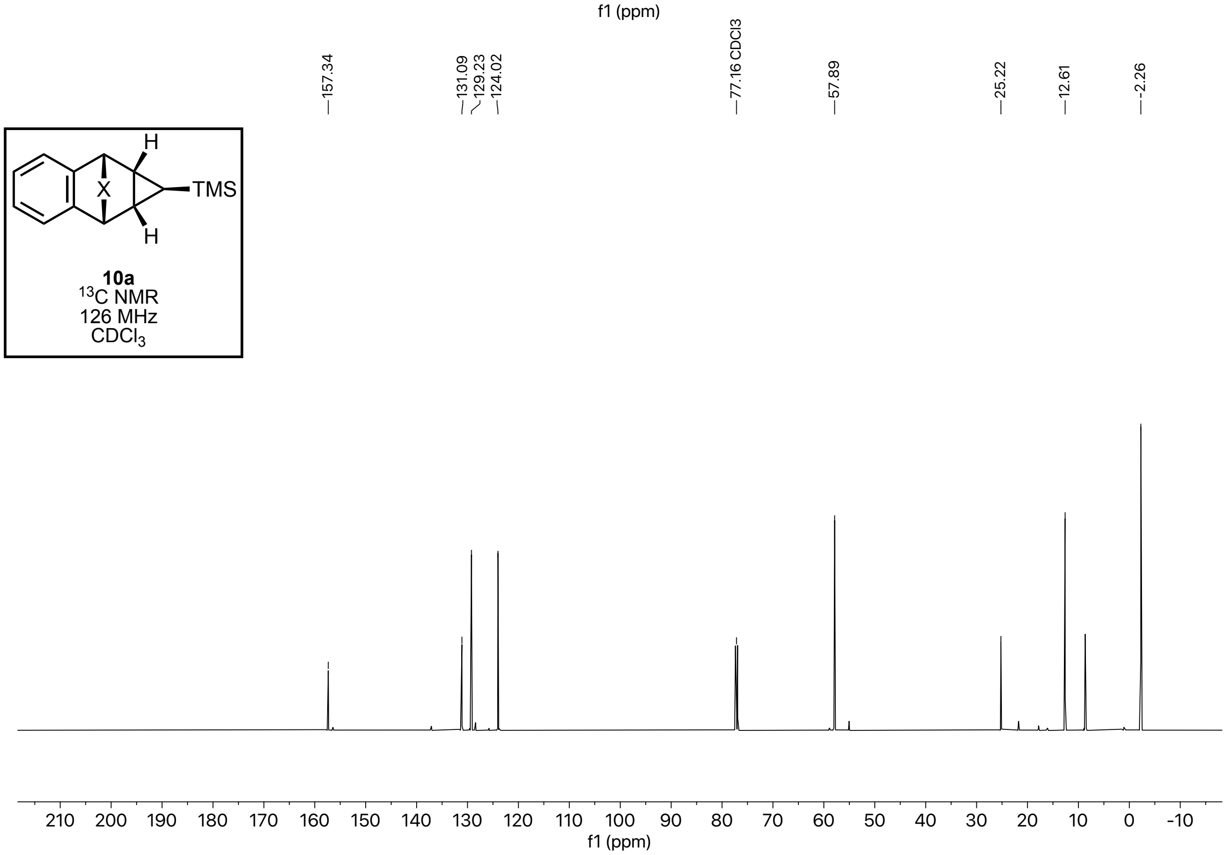

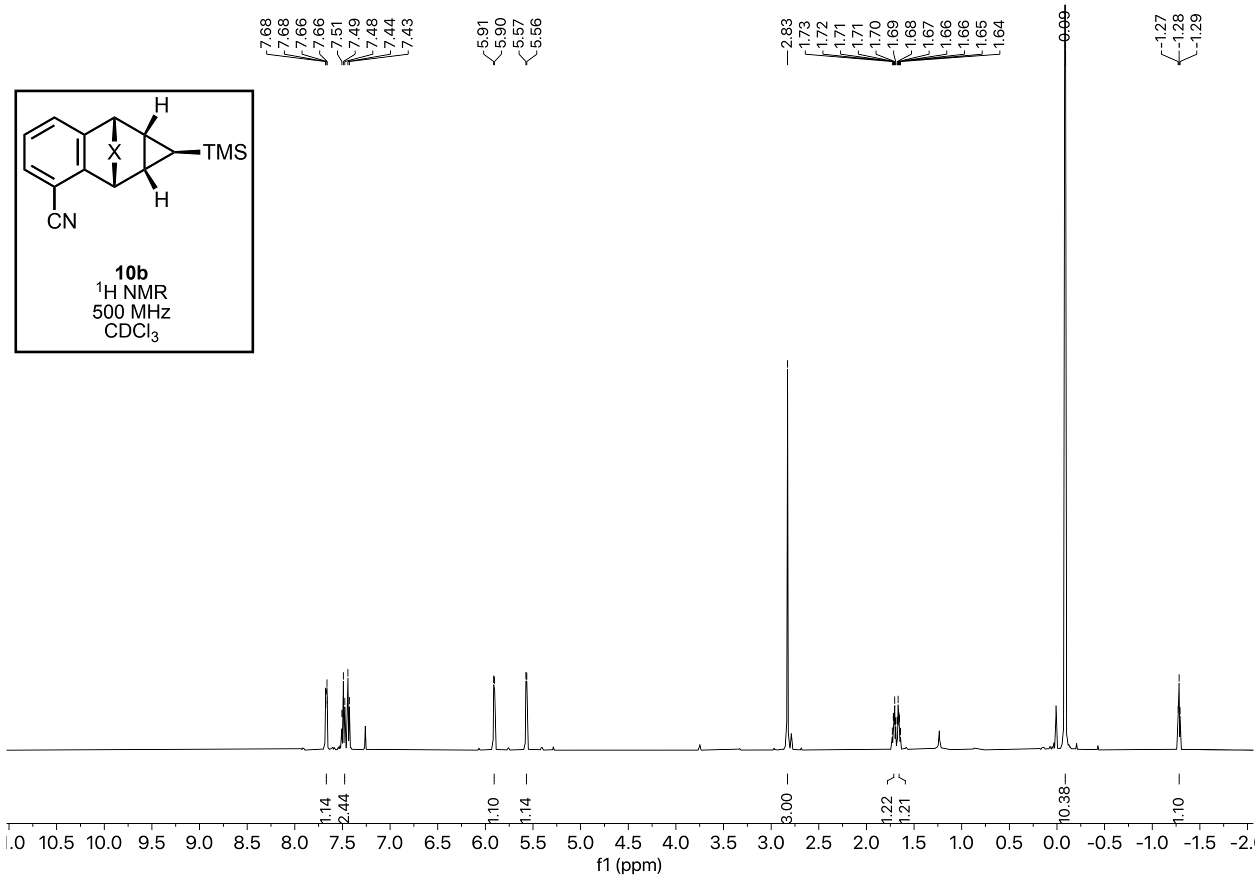
7.68
7.68
7.66
7.66
7.51
7.49
7.48
7.44
7.43
5.91
5.90
5.57
5.56
2.83
1.73
1.72
1.71
1.71
1.70
1.69
1.68
1.67
1.66
1.66
1.65
1.64
0.00
-1.27
-1.28
-1.29
H
X
TMS
H
CN
10b
1H NMR
500 MHz
CDCl3
1.14
2.44
1.10
1.14
3.00
1.22
1.21
10.38
1.10
f1 (ppm)

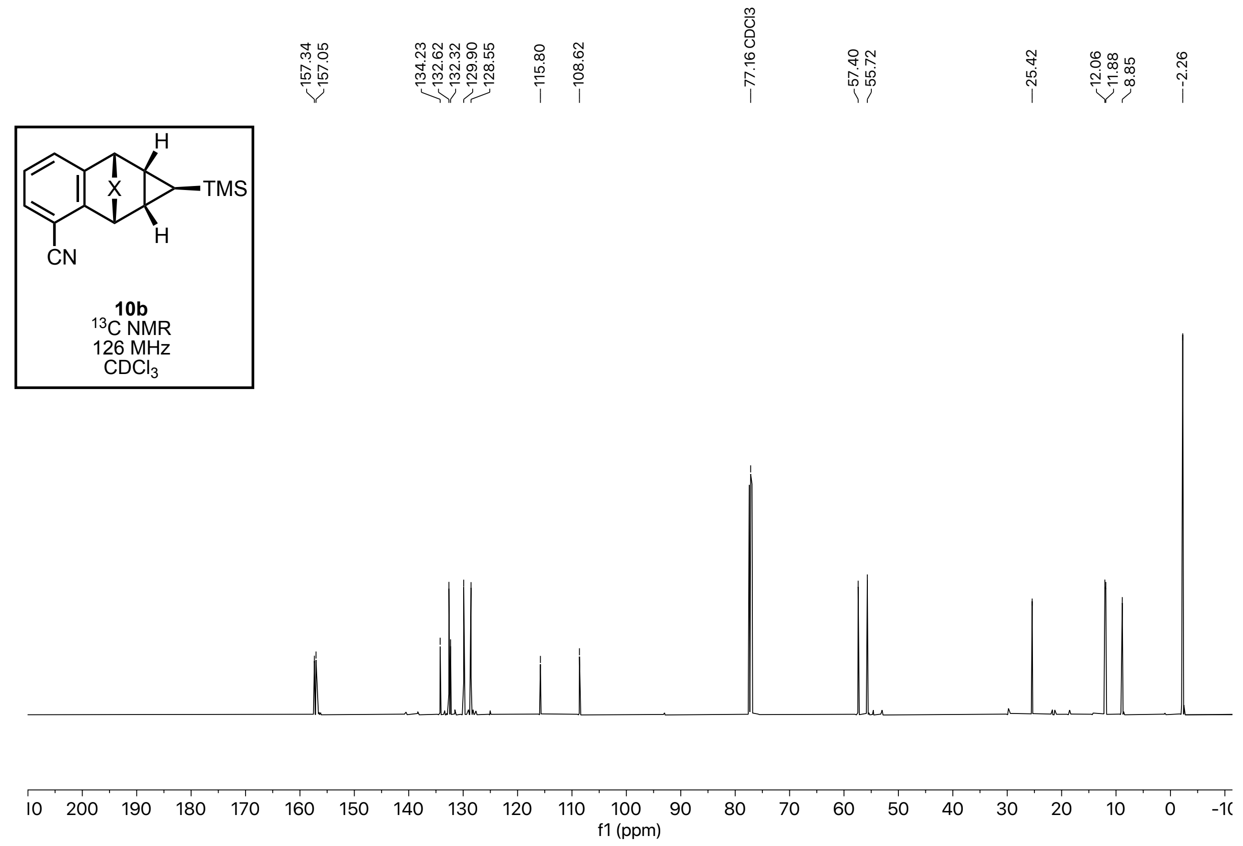
157.34
157.05
134.23
132.62
132.32
129.90
128.55
115.80
108.62
77.16 CDCl3
57.40
55.72
25.42
12.06
11.88
8.85
-2.26
H
X
TMS
H
CN
10b
13C NMR
126 MHz
CDCl3
f1 (ppm)

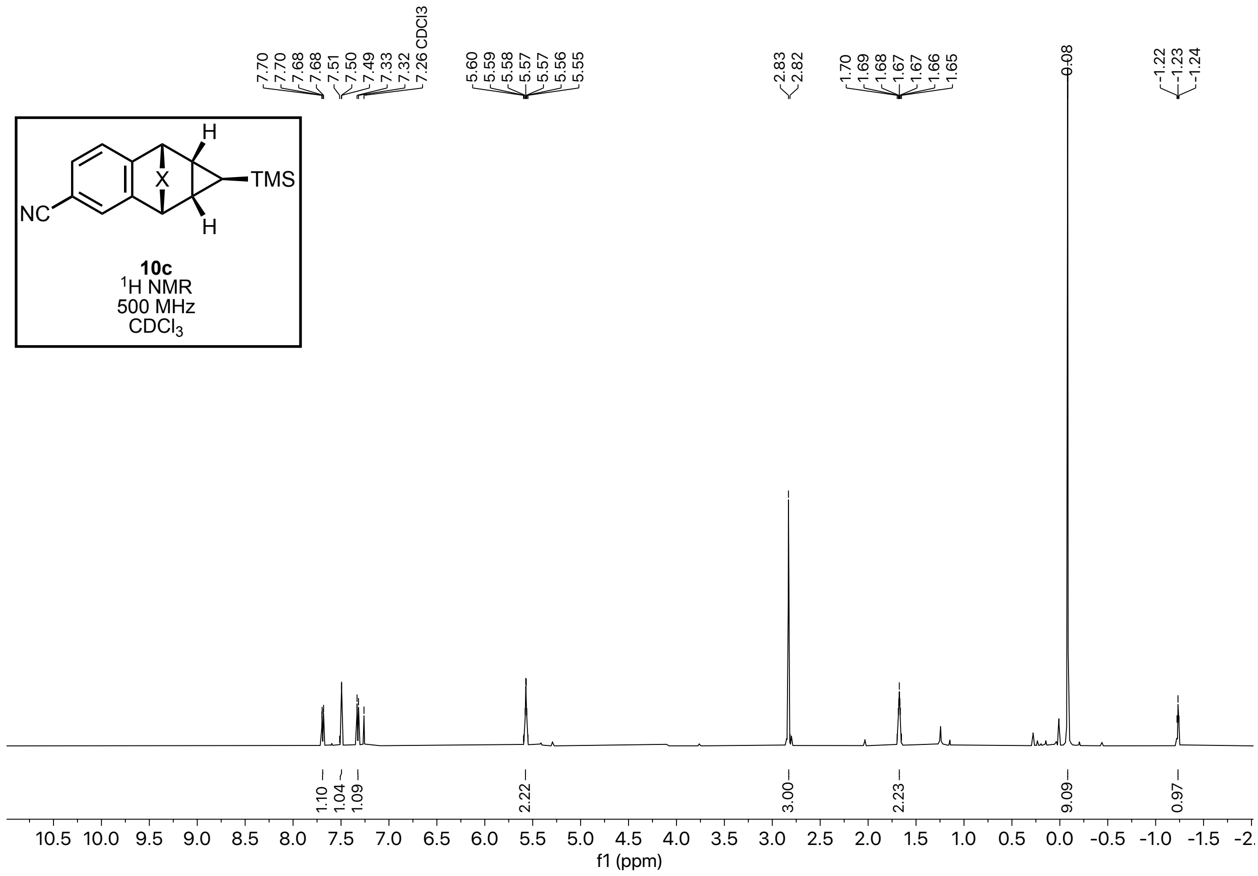
7.70
7.70
7.68
7.68
7.51
7.50
7.49
7.33
7.32
7.26 CDCl3
5.60
5.59
5.58
5.57
5.57
5.56
5.55
2.83
2.82
1.70
1.69
1.68
1.67
1.67
1.66
1.65
0.08
-1.22
-1.23
-1.24
NC
X
H
H
TMS
10c
$^1$H NMR
500 MHz
$CDCl_3$
1.10
1.04
1.09
2.22
3.00
2.23
9.09
0.97
f1 (ppm)

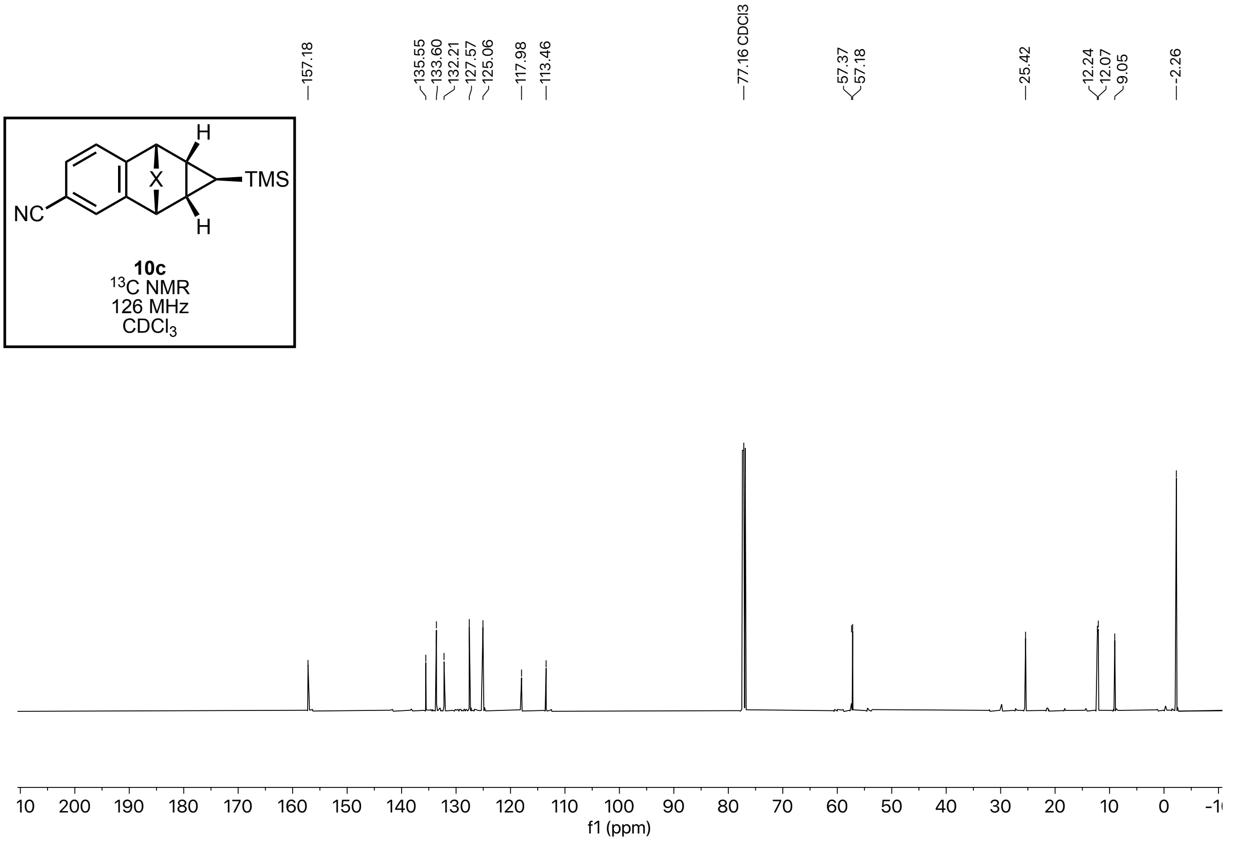
157.18
135.55
133.60
132.21
127.57
125.06
117.98
113.46
77.16 CDCl3
57.37
57.18
25.42
12.24
12.07
9.05
-2.26
NC
X
H
H
TMS
10c
$^{13}$C NMR
126 MHz
$CDCl_3$
f1 (ppm)

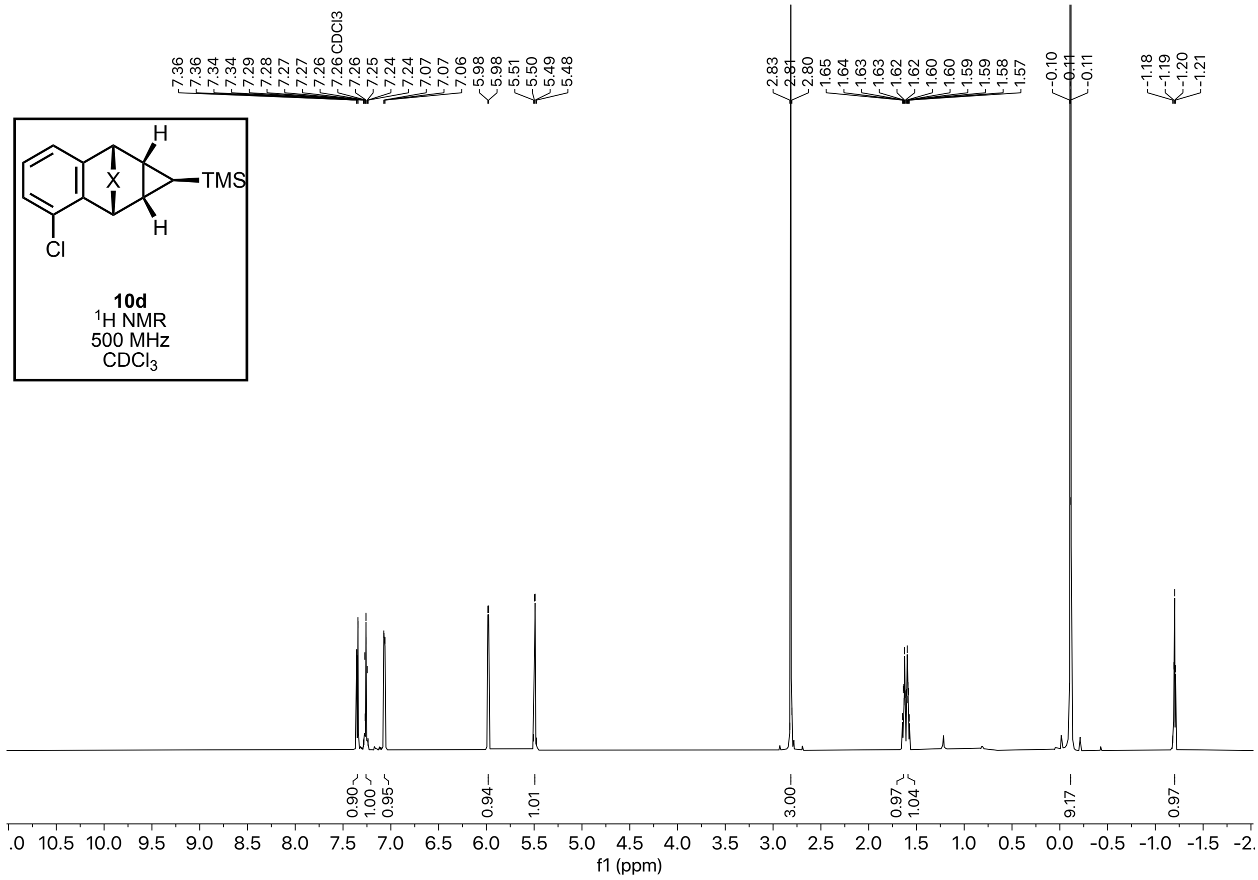
7.36
7.36
7.34
7.34
7.29
7.28
7.27
7.27
7.26
7.26 CDCl3
7.26
7.25
7.24
7.24
7.07
7.07
7.06
5.98
5.98
5.51
5.50
5.49
5.48
2.83
2.81
2.80
1.65
1.64
1.63
1.63
1.62
1.62
1.60
1.60
1.59
1.59
1.58
1.57
-0.10
-0.11
-0.11
-1.18
-1.19
-1.20
-1.21
H
X
TMS
H
Cl
10d
1H NMR
500 MHz
CDCl3
0.90
1.00
0.95
0.94
1.01
3.00
0.97
1.04
9.17
0.97
f1 (ppm)

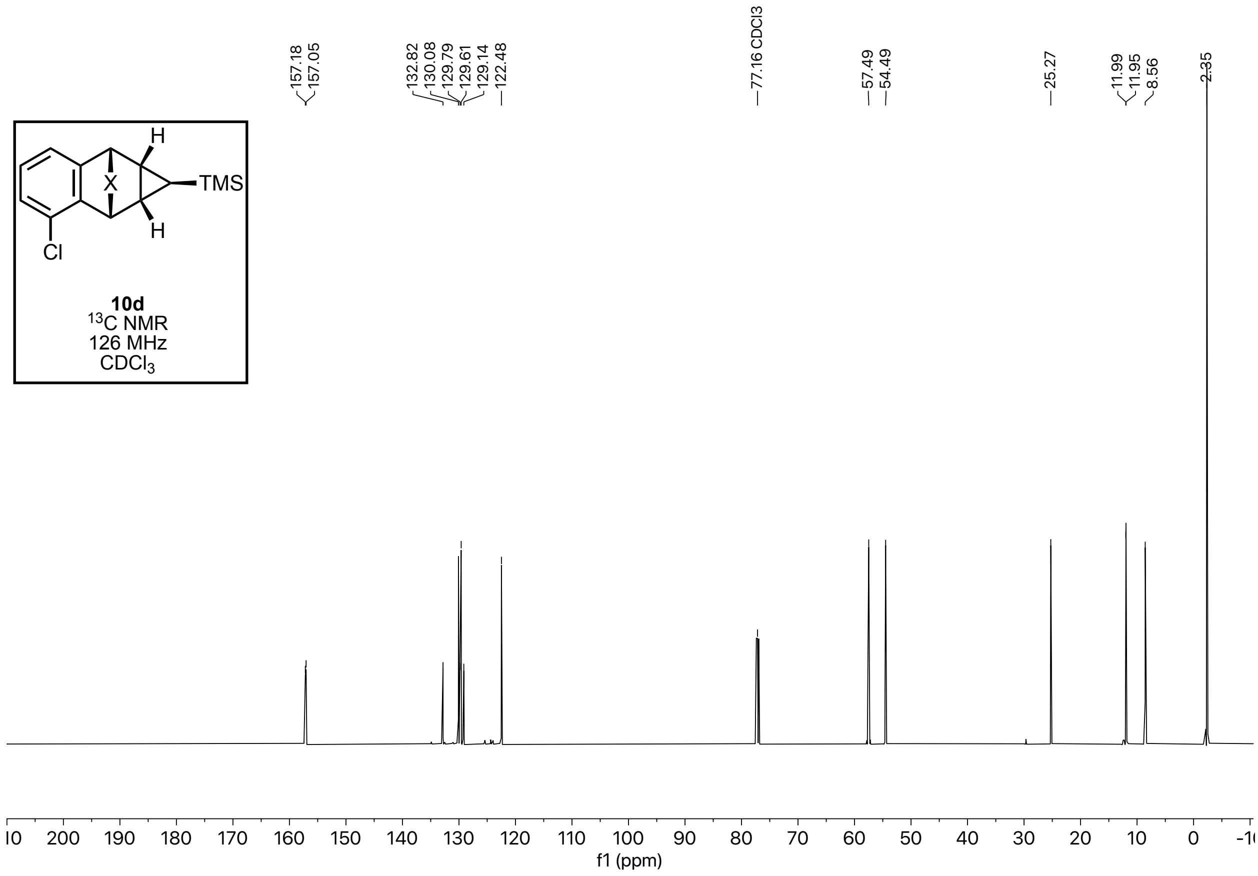
157.18
157.05
132.82
130.08
129.79
129.61
129.14
122.48
77.16 CDCl3
57.49
54.49
25.27
11.99
11.95
8.56
-2.35
H
X
TMS
H
Cl
10d
13C NMR
126 MHz
CDCl3
f1 (ppm)

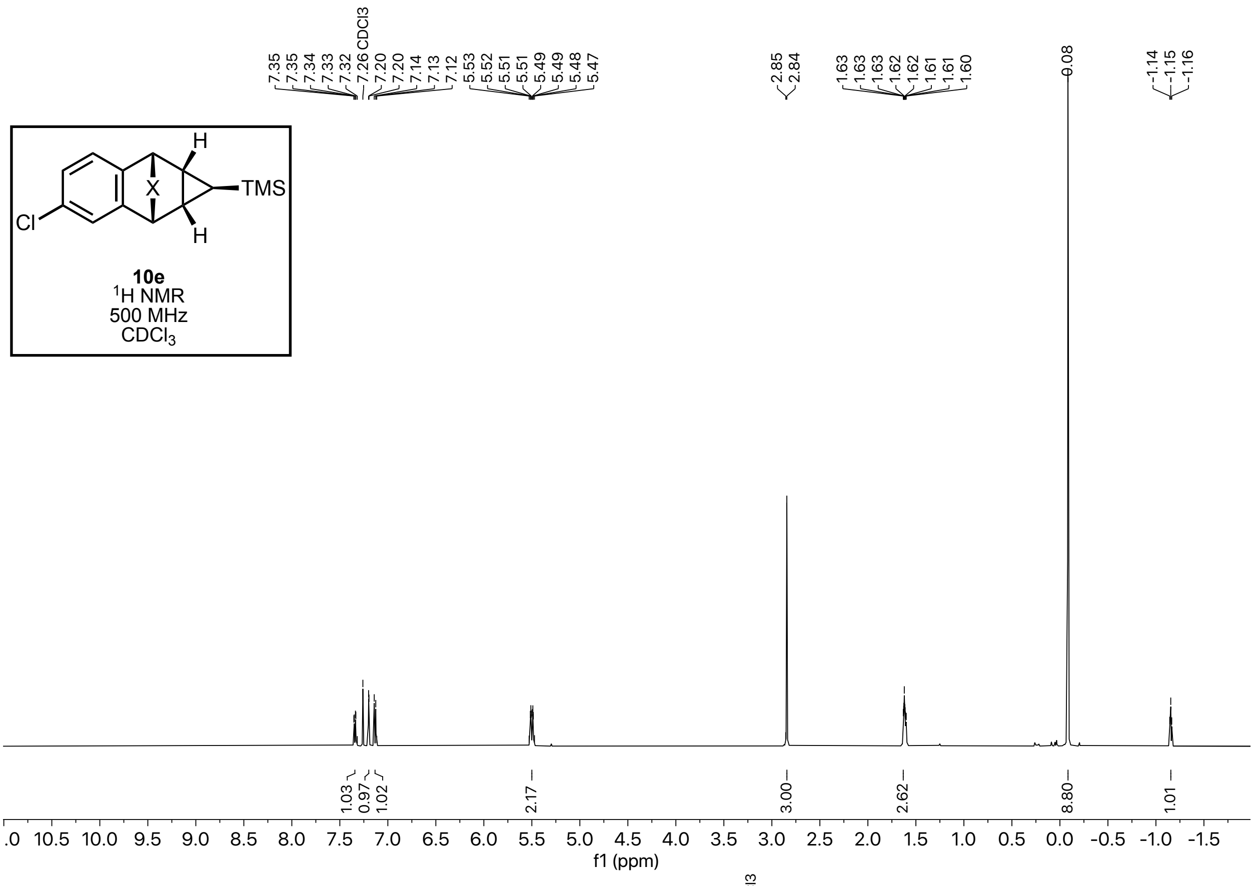
7.35 7.35 7.34 7.33 7.32 7.26 CDCl3 7.20 7.20 7.14 7.13 7.12 5.53 5.52 5.51 5.51 5.49 5.49 5.48 5.47
2.85 2.84
1.63 1.63 1.63 1.62 1.62 1.61 1.61 1.60
0.08
-1.14 -1.15 -1.16
H
X
TMS
Cl
H
10e
1H NMR
500 MHz
CDCl3
1.03 0.97 1.02
2.17
3.00
2.62
8.80
1.01
f1 (ppm)

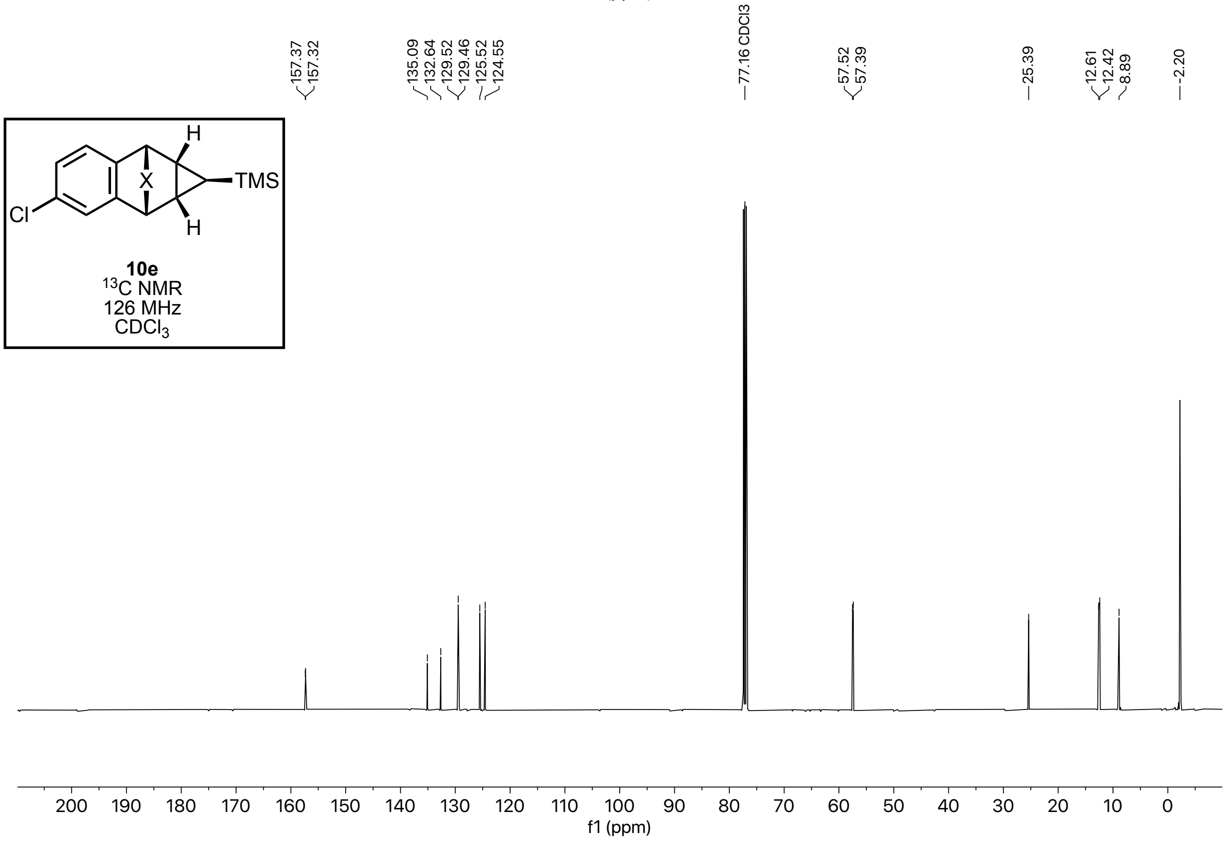
157.37 157.32
135.09 132.64 129.52 129.46 125.52 124.55
77.16 CDCl3
57.52 57.39
25.39
12.61 12.42 8.89
-2.20
H
X
TMS
Cl
H
10e
13C NMR
126 MHz
CDCl3
f1 (ppm)

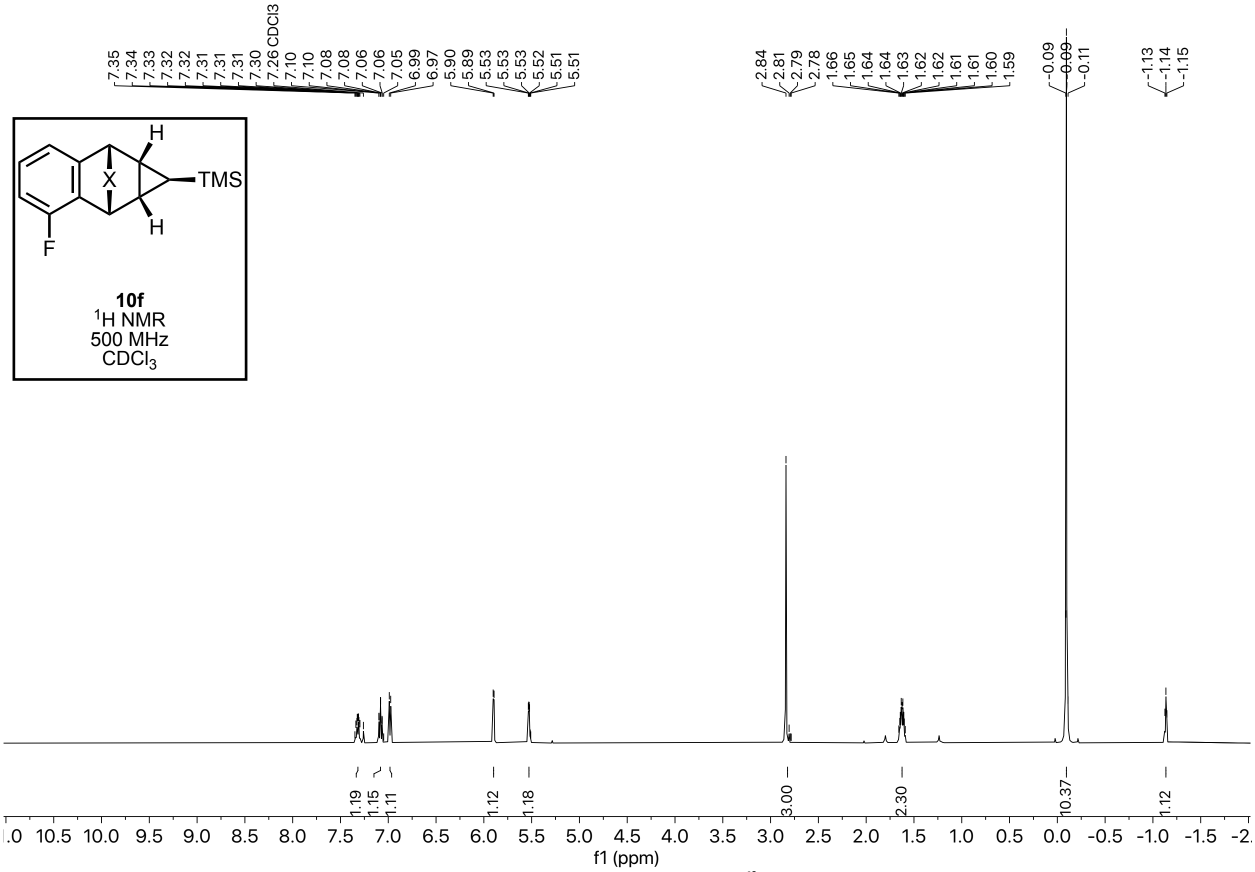
10f
$^1$H NMR
500 MHz
$CDCl_3$
TMS
H
H
X
F
f1 (ppm)

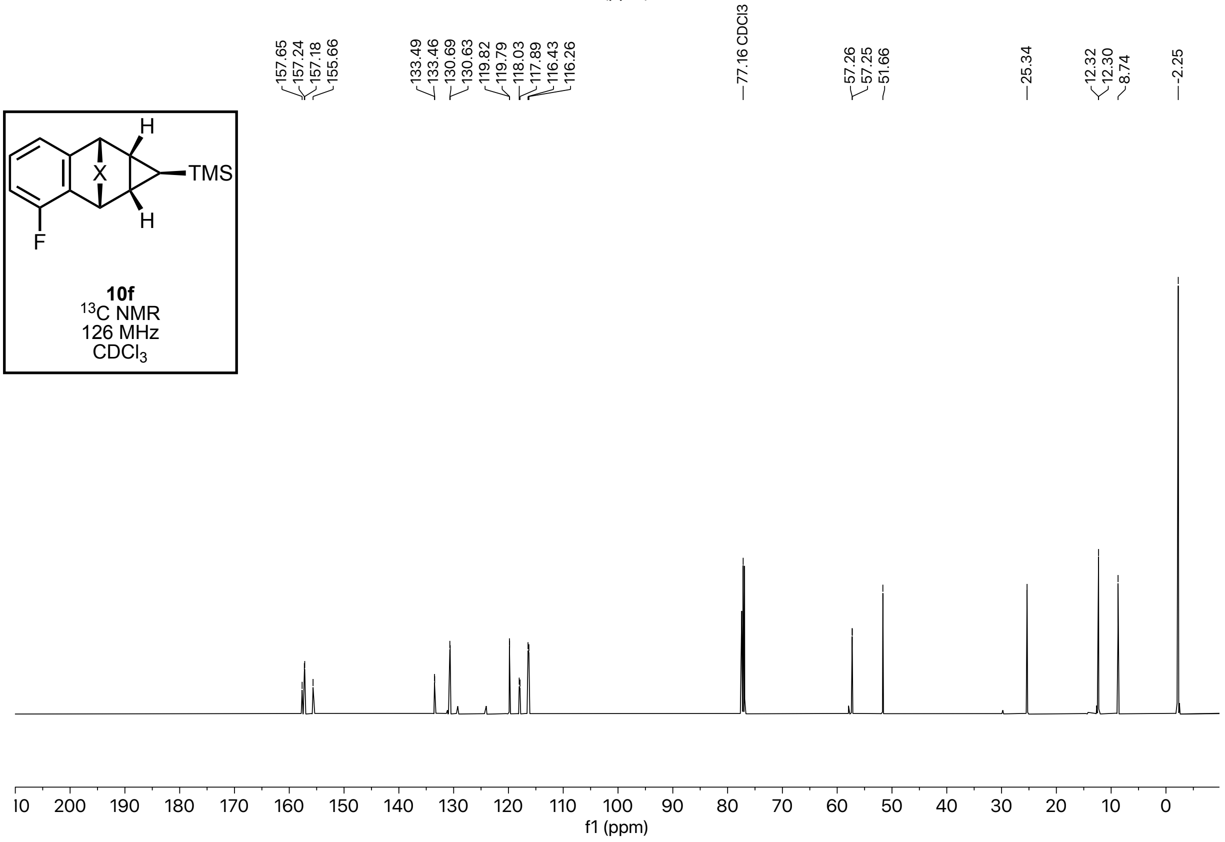
10f
$^{13}$C NMR
126 MHz
$CDCl_3$
TMS
H
H
X
F
f1 (ppm)

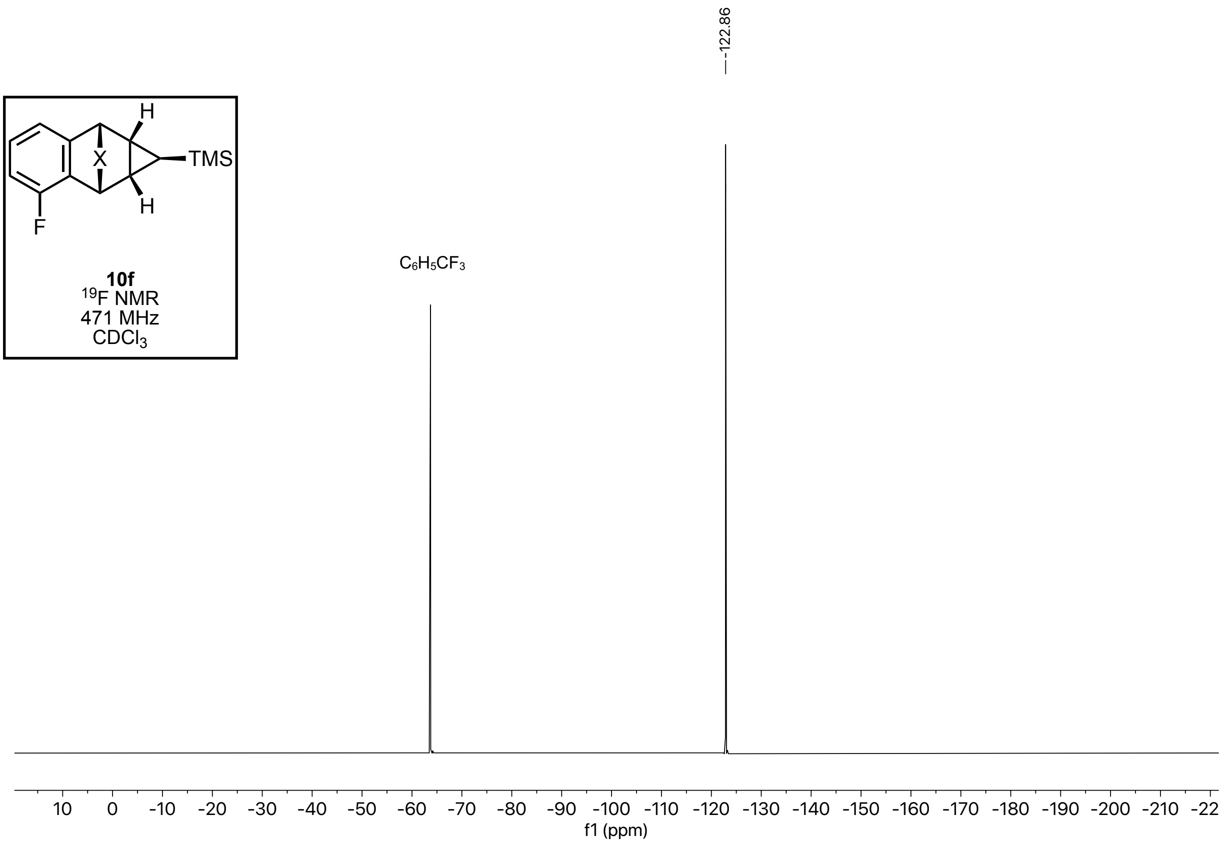
-122.86
H
X
TMS
H
F
10f
19F NMR
471 MHz
CDCl3
C6H5CF3
10 0 -10 -20 -30 -40 -50 -60 -70 -80 -90 -100 -110 -120 -130 -140 -150 -160 -170 -180 -190 -200 -210 -22
f1 (ppm)

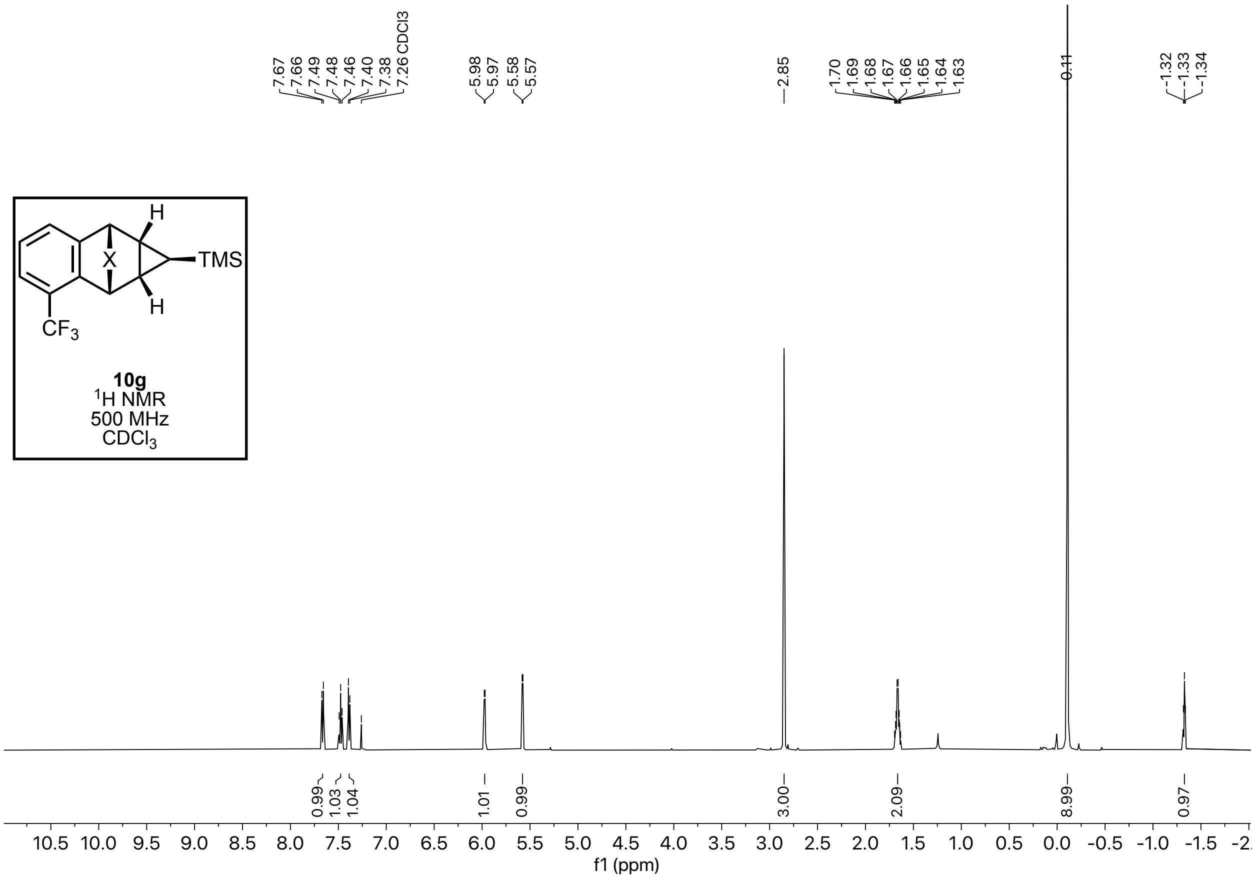
7.67
7.66
7.49
7.48
7.46
7.40
7.38
7.26 CDCl3
5.98
5.97
5.58
5.57
2.85
1.70
1.69
1.68
1.67
1.66
1.65
1.64
1.63
0.11
-1.32
-1.33
-1.34
H
X
TMS
H
CF3
10g
1H NMR
500 MHz
CDCl3
0.99
1.03
1.04
1.01
0.99
3.00
2.09
8.99
0.97
10.5 10.0 9.5 9.0 8.5 8.0 7.5 7.0 6.5 6.0 5.5 5.0 4.5 4.0 3.5 3.0 2.5 2.0 1.5 1.0 0.5 0.0 -0.5 -1.0 -1.5 -2.
f1 (ppm)

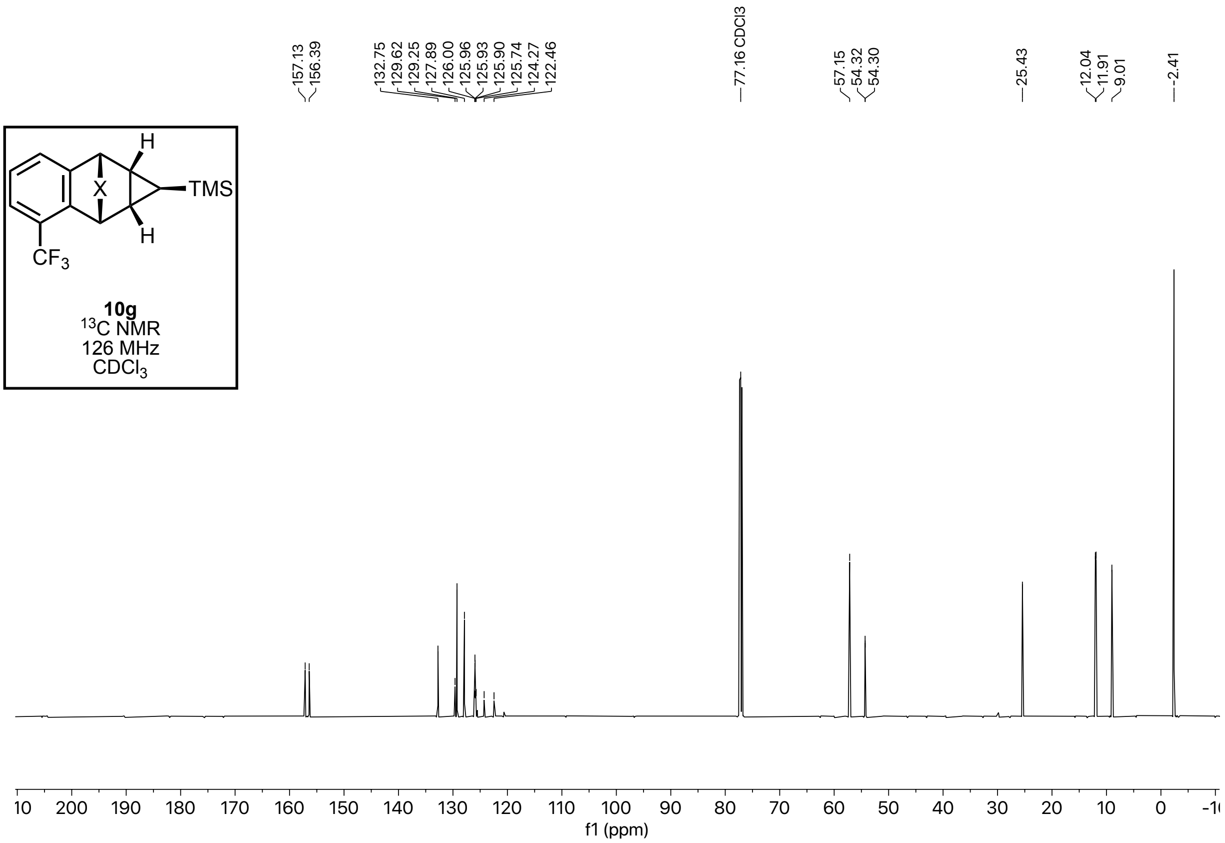
157.13
156.39
132.75
129.62
129.25
127.89
126.00
125.96
125.93
125.90
125.74
124.27
122.46
77.16 CDCl3
57.15
54.32
54.30
25.43
12.04
11.91
9.01
-2.41
H
X
TMS
H
CF3
10g
13C NMR
126 MHz
CDCl3
f1 (ppm)

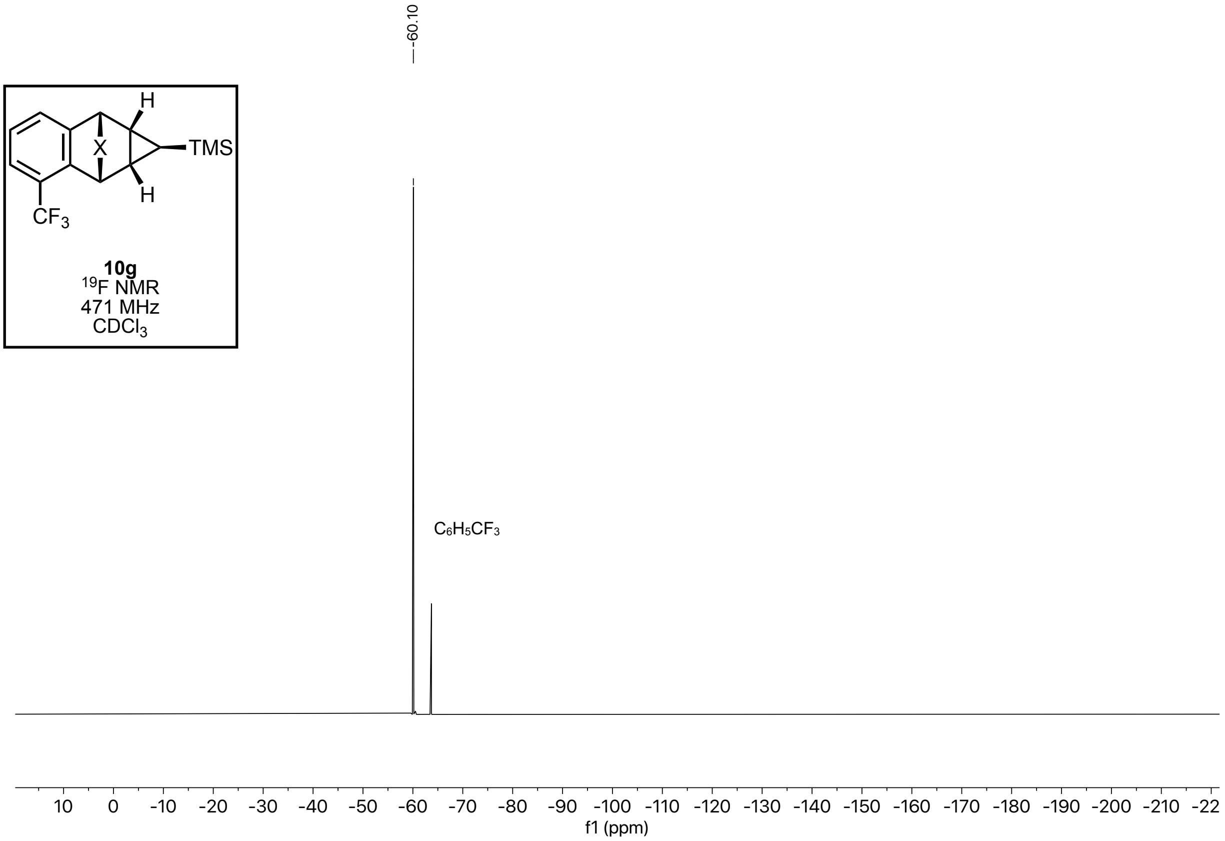
-60.10
H
X
TMS
H
CF3
10g
19F NMR
471 MHz
CDCl3
C6H5CF3
f1 (ppm)

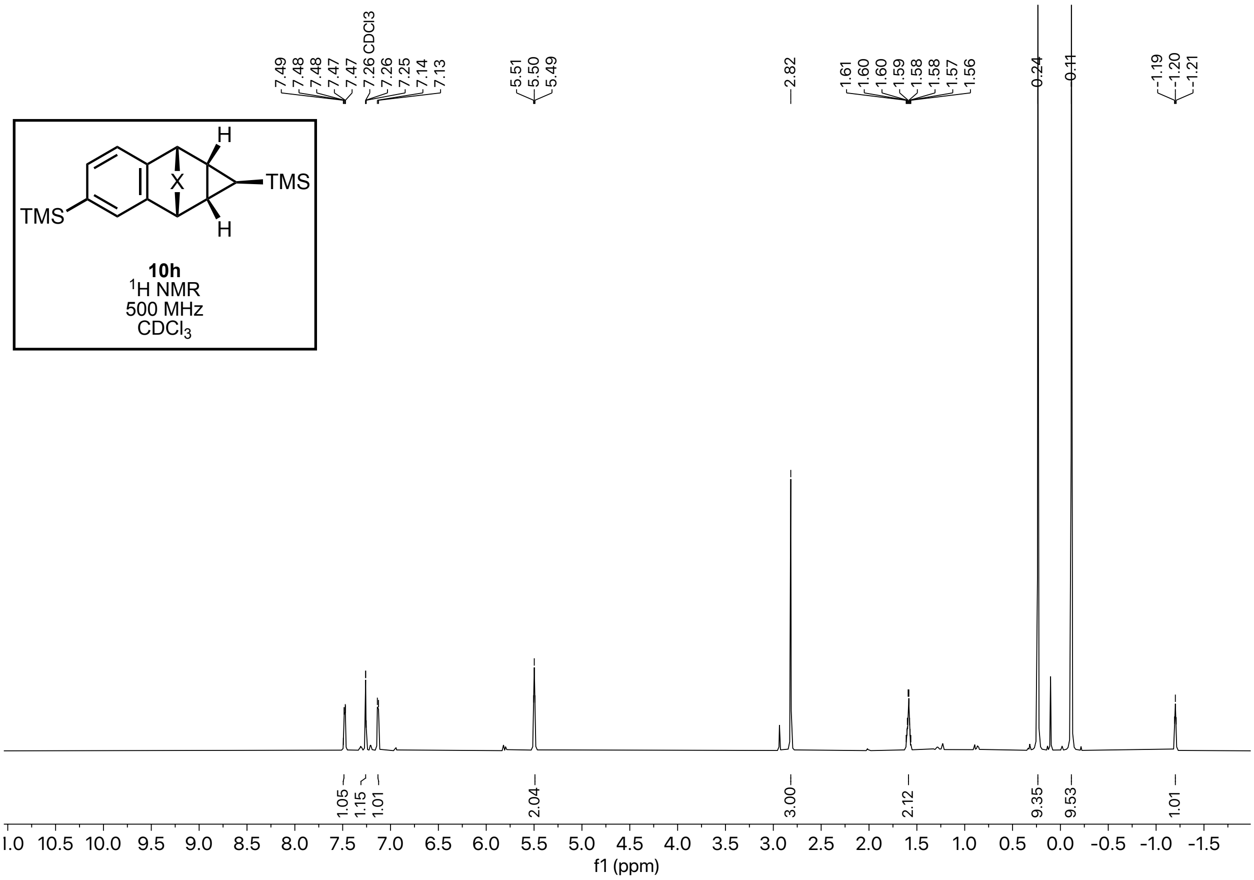
7.49
7.48
7.48
7.47
7.47
7.26 CDCl3
7.26
7.25
7.14
7.13
5.51
5.50
5.49
2.82
1.61
1.60
1.60
1.59
1.58
1.58
1.57
1.56
0.24
0.11
-1.19
-1.20
-1.21
TMS
TMS
H
H
X
10h
1H NMR
500 MHz
CDCl3
1.05
1.15
1.01
2.04
3.00
2.12
9.35
9.53
1.01
f1 (ppm)

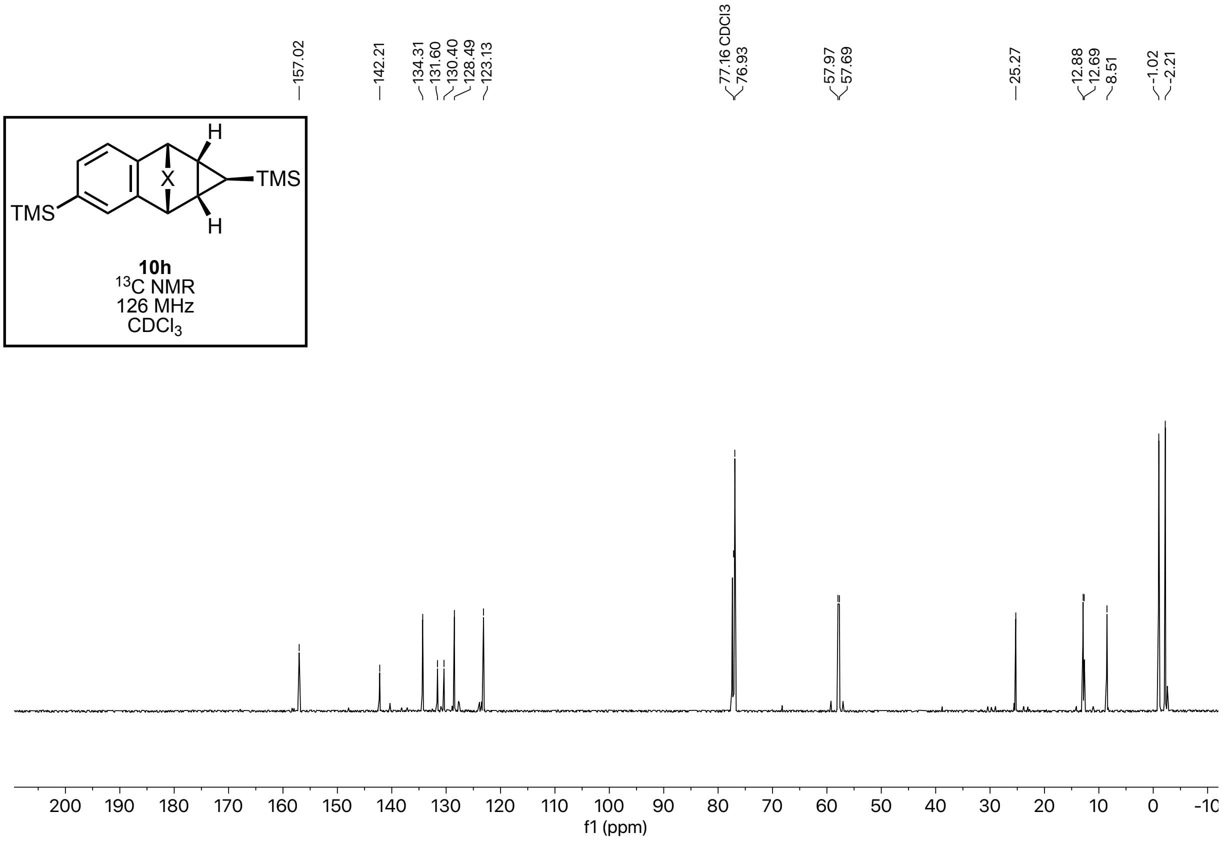
157.02
142.21
134.31
131.60
130.40
128.49
123.13
77.16 CDCl3
76.93
57.97
57.69
25.27
12.88
12.69
8.51
-1.02
-2.21
TMS
TMS
H
H
X
10h
13C NMR
126 MHz
CDCl3
f1 (ppm)

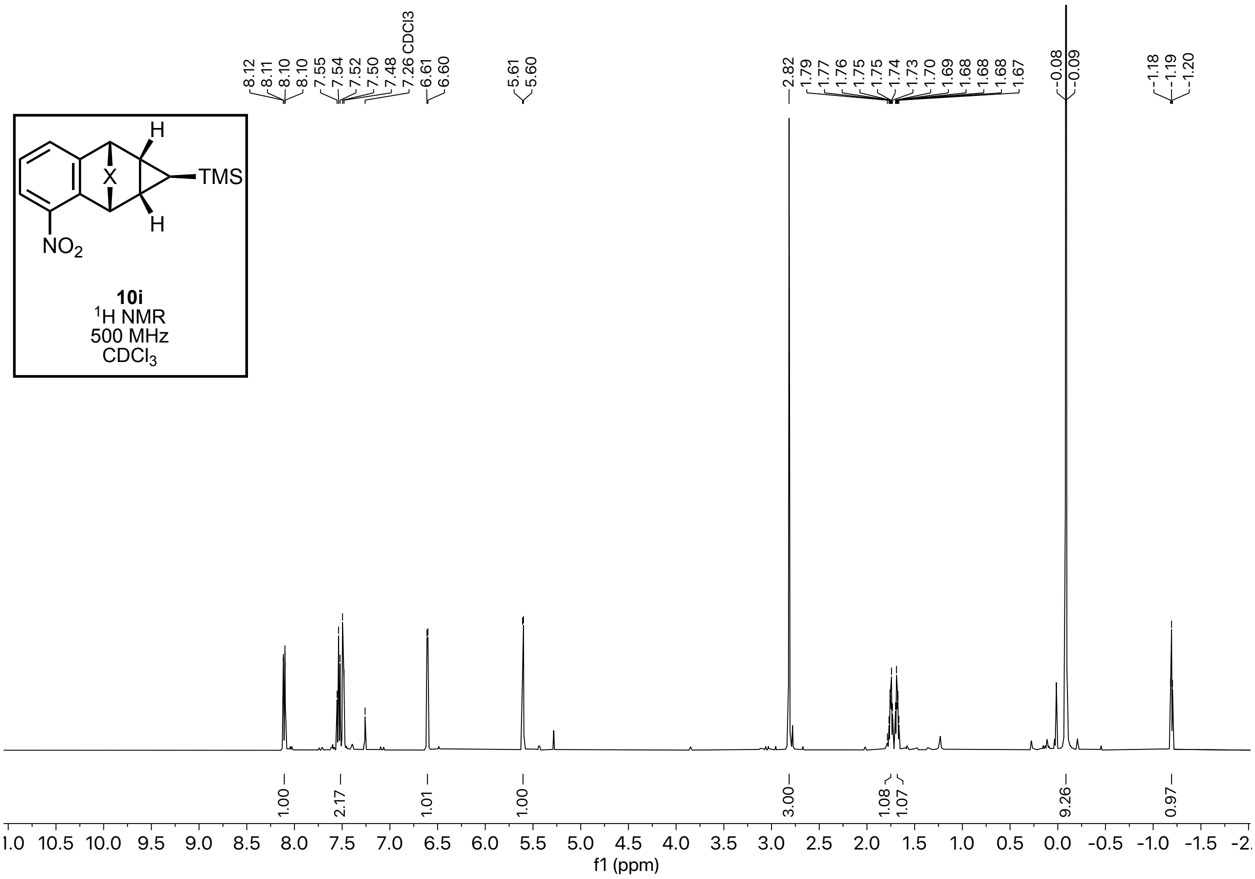
TMS
NO2
X
H
H
10i
1H NMR
500 MHz
CDCl3
8.12
8.11
8.10
8.10
7.55
7.54
7.52
7.50
7.48
7.26 CDCl3
6.61
6.60
5.61
5.60
2.82
1.79
1.77
1.76
1.75
1.75
1.74
1.73
1.70
1.69
1.68
1.68
1.68
1.67
-0.08
-0.09
-1.18
-1.19
-1.20
1.00
2.17
1.01
1.00
3.00
1.08
1.07
9.26
0.97
f1 (ppm)

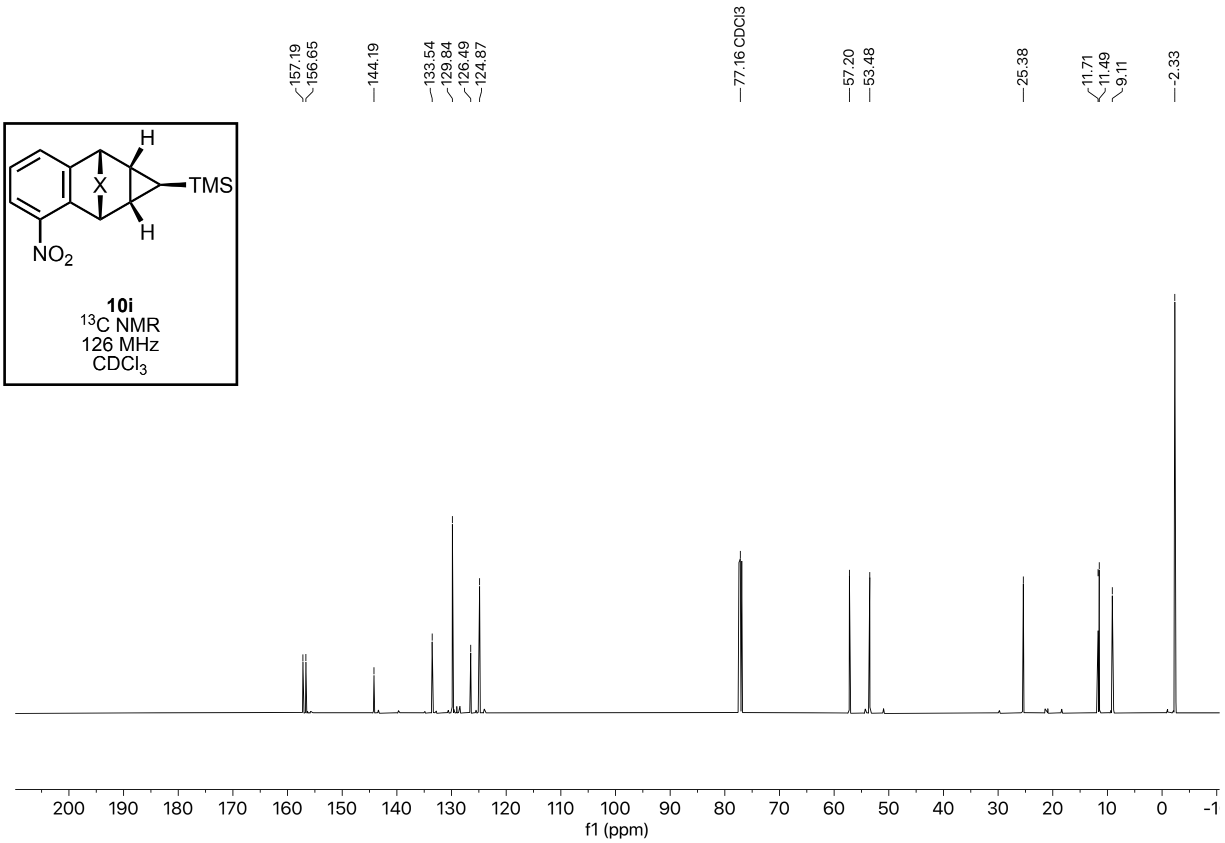
TMS
NO2
X
H
H
10i
13C NMR
126 MHz
CDCl3
157.19
156.65
144.19
133.54
129.84
126.49
124.87
77.16 CDCl3
57.20
53.48
25.38
11.71
11.49
9.11
-2.33
f1 (ppm)

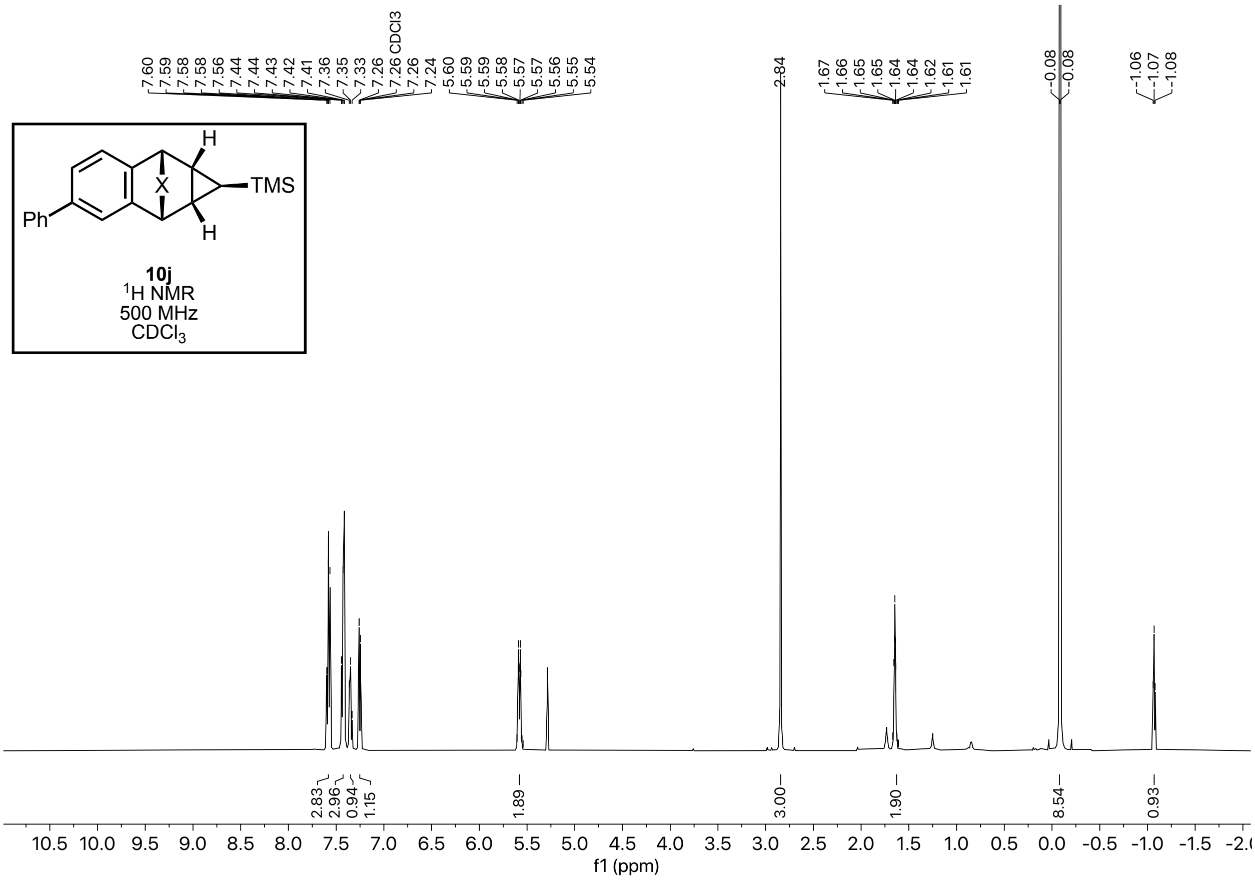
Ph
X
H
H
TMS
10j
1H NMR
500 MHz
CDCl3
7.60 7.59 7.58 7.58 7.56 7.44 7.44 7.43 7.42 7.41 7.36 7.35 7.33 7.26 7.26 CDCl3 7.26 7.24 5.60 5.59 5.59 5.58 5.57 5.57 5.56 5.55 5.54
2.84
1.67 1.66 1.65 1.65 1.64 1.64 1.62 1.61 1.61
-0.08 -0.08
-1.06 -1.07 -1.08
2.83 2.96 0.94 1.15 1.89 3.00 1.90 8.54 0.93
f1 (ppm)

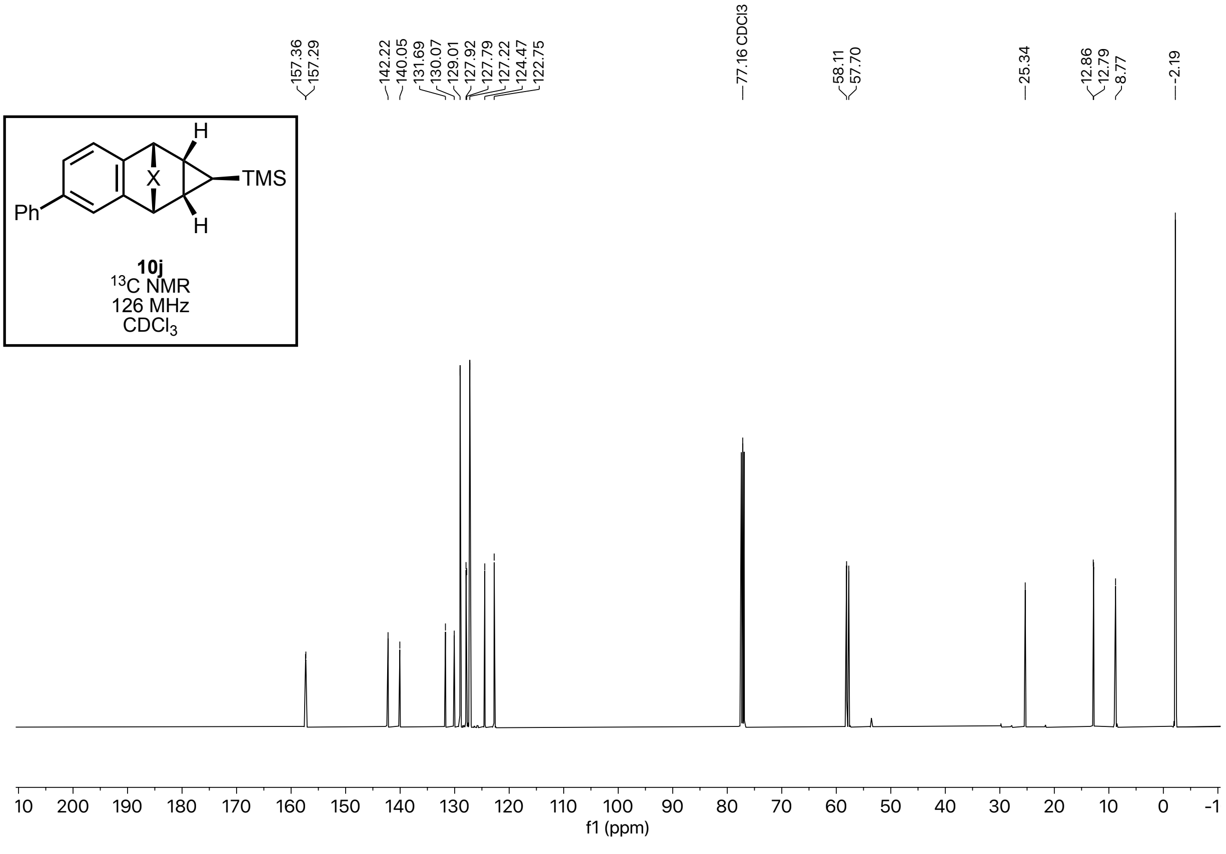
Ph
X
H
H
TMS
10j
13C NMR
126 MHz
CDCl3
157.36 157.29
142.22 140.05 131.69 130.07 129.01 127.92 127.79 127.22 124.47 122.75
77.16 CDCl3
58.11 57.70
25.34
12.86 12.79 8.77
-2.19
f1 (ppm)

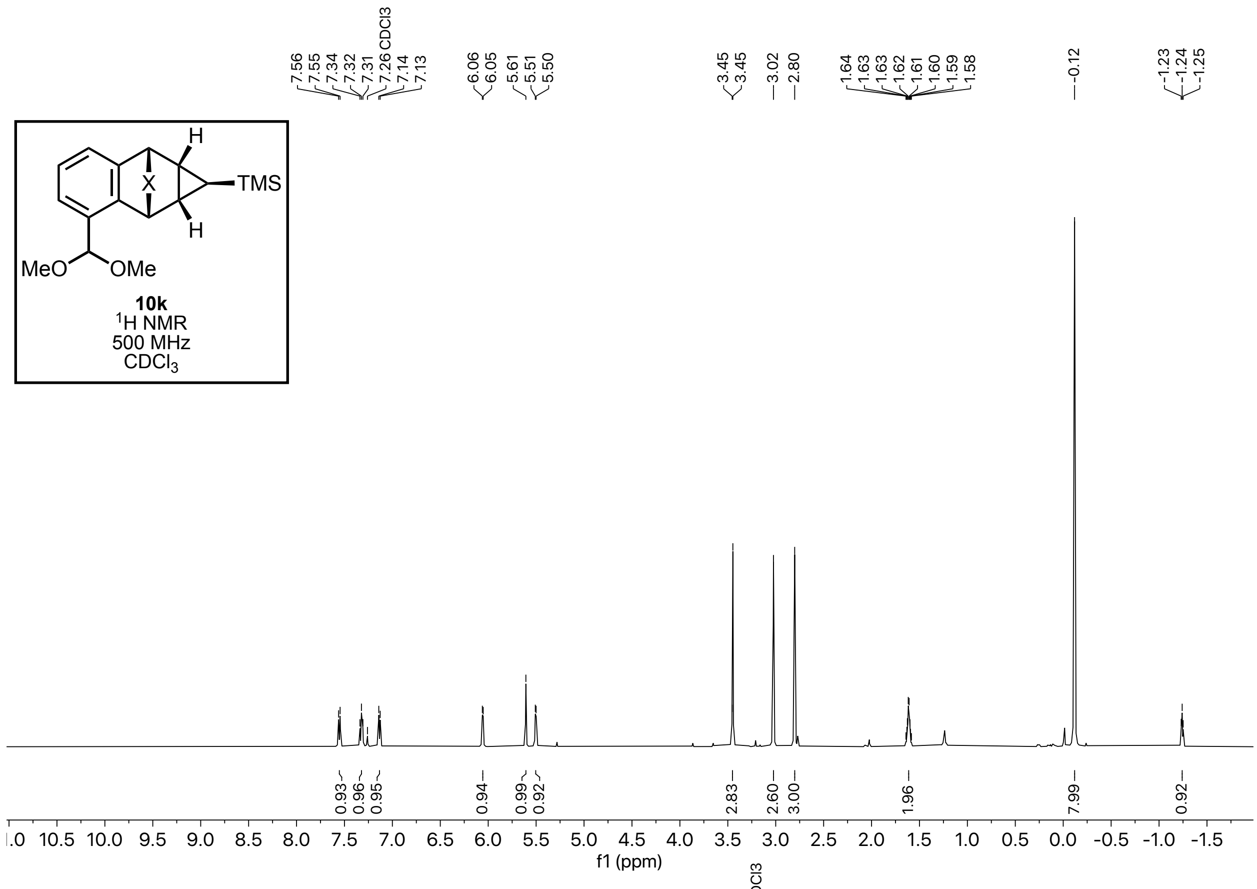
10k
$^1$H NMR
500 MHz
$CDCl_3$
TMS
MeO
OMe
f1 (ppm)

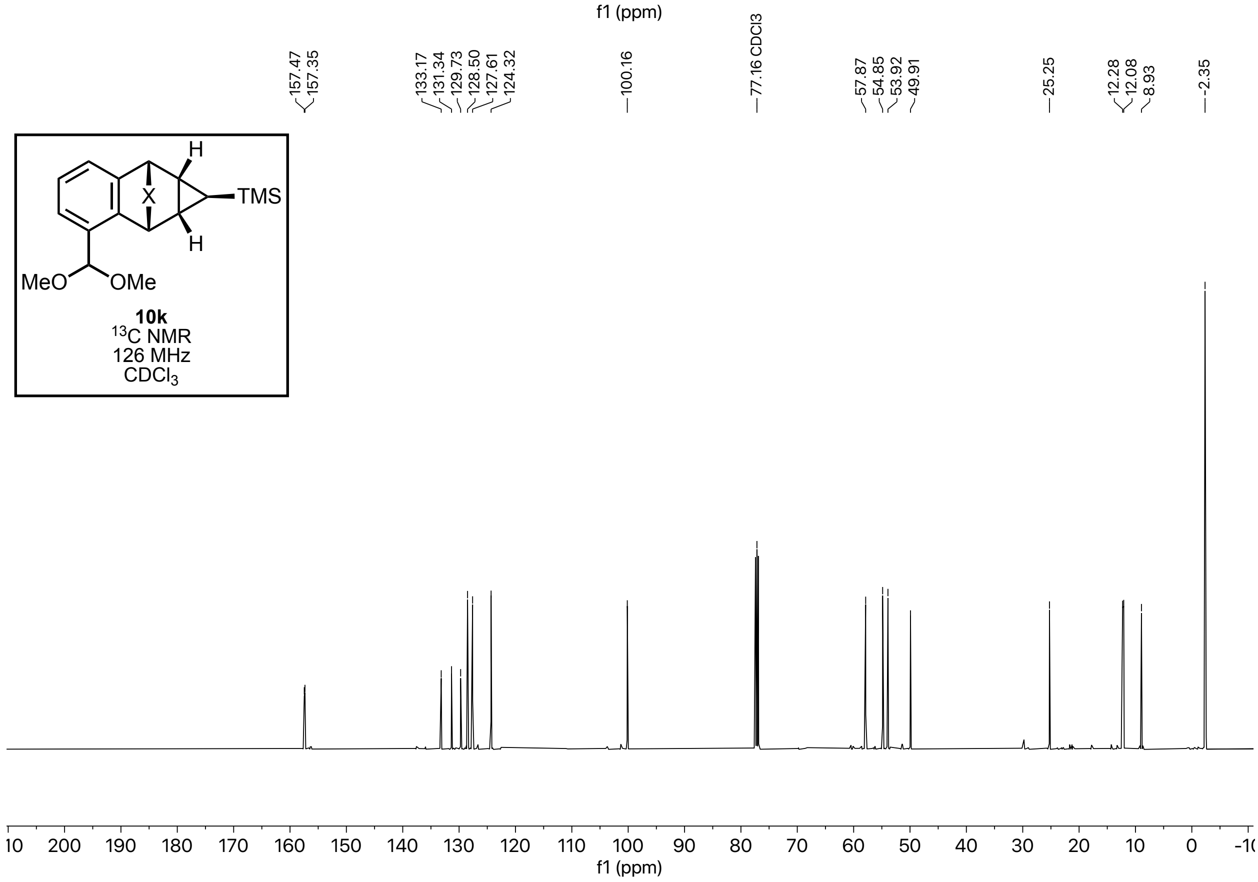
10k
$^{13}$C NMR
126 MHz
$CDCl_3$
TMS
MeO
OMe
f1 (ppm)

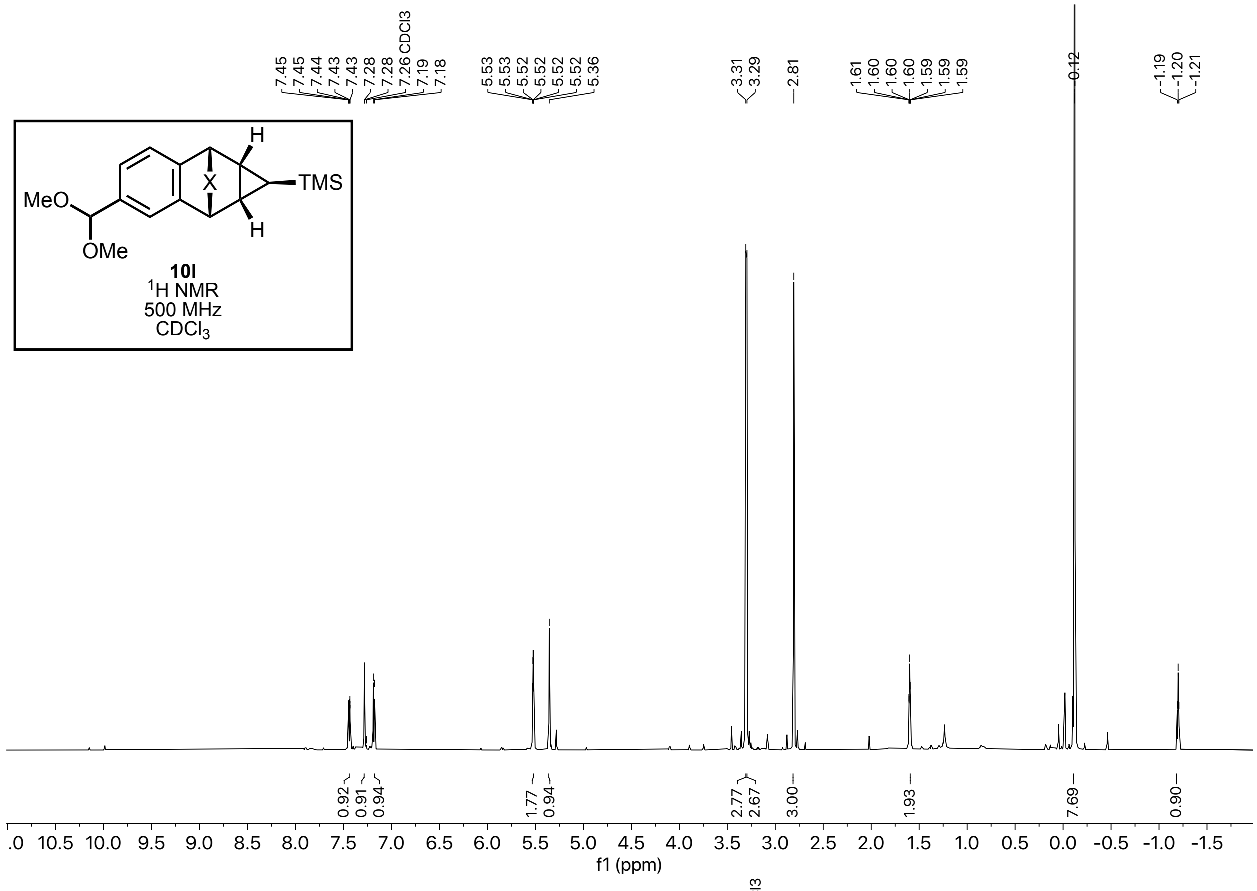
7.45
7.45
7.44
7.43
7.43
7.28
7.28
7.26 CDCl3
7.19
7.18
5.53
5.53
5.52
5.52
5.52
5.52
5.36
3.31
3.29
2.81
1.61
1.60
1.60
1.60
1.59
1.59
1.59
0.12
-1.19
-1.20
-1.21
H
X
TMS
MeO
H
OMe
10l
1H NMR
500 MHz
CDCl3
0.92
0.91
0.94
1.77
0.94
2.77
2.67
3.00
1.93
7.69
0.90
f1 (ppm)

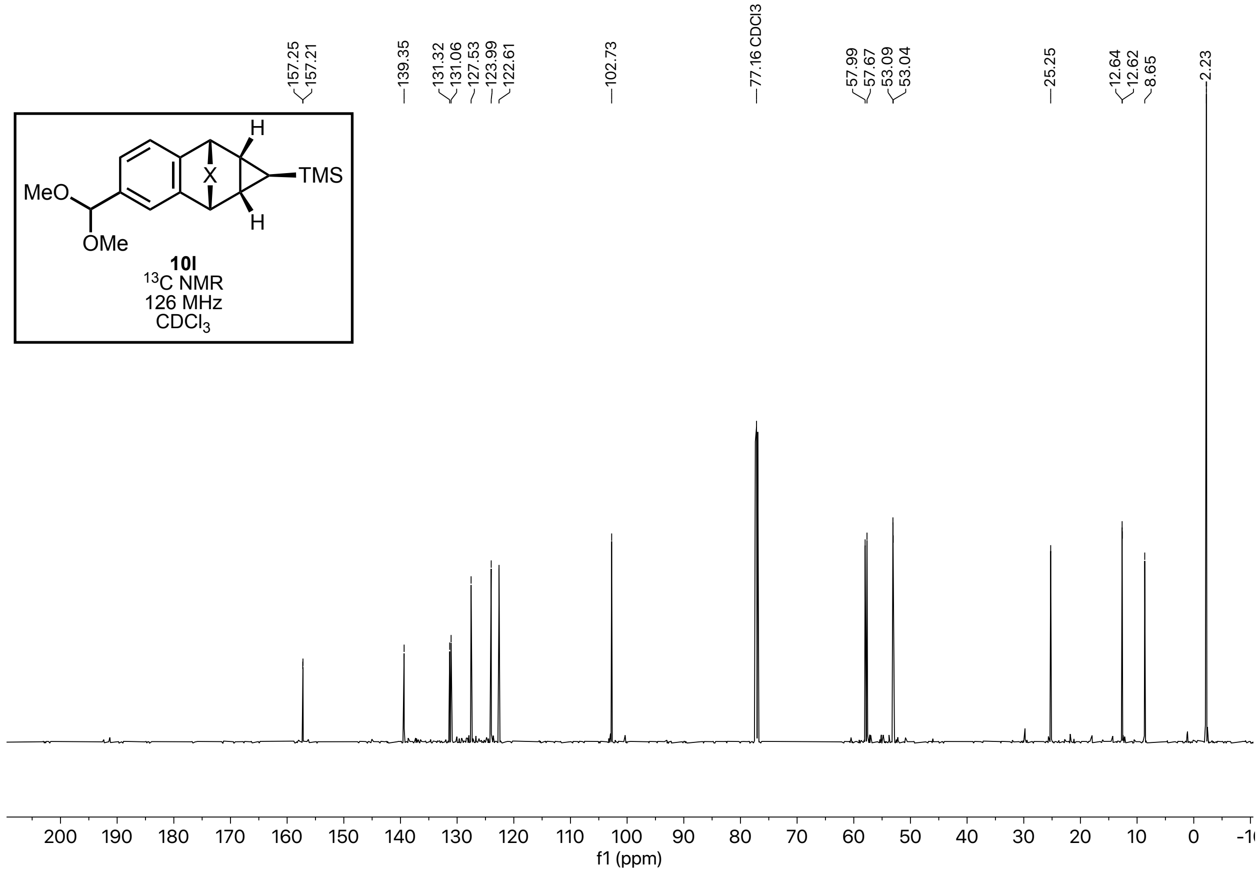
157.25
157.21
139.35
131.32
131.06
127.53
123.99
122.61
102.73
77.16 CDCl3
57.99
57.67
53.09
53.04
25.25
12.64
12.62
8.65
-2.23
H
X
TMS
MeO
H
OMe
10l
13C NMR
126 MHz
CDCl3
f1 (ppm)

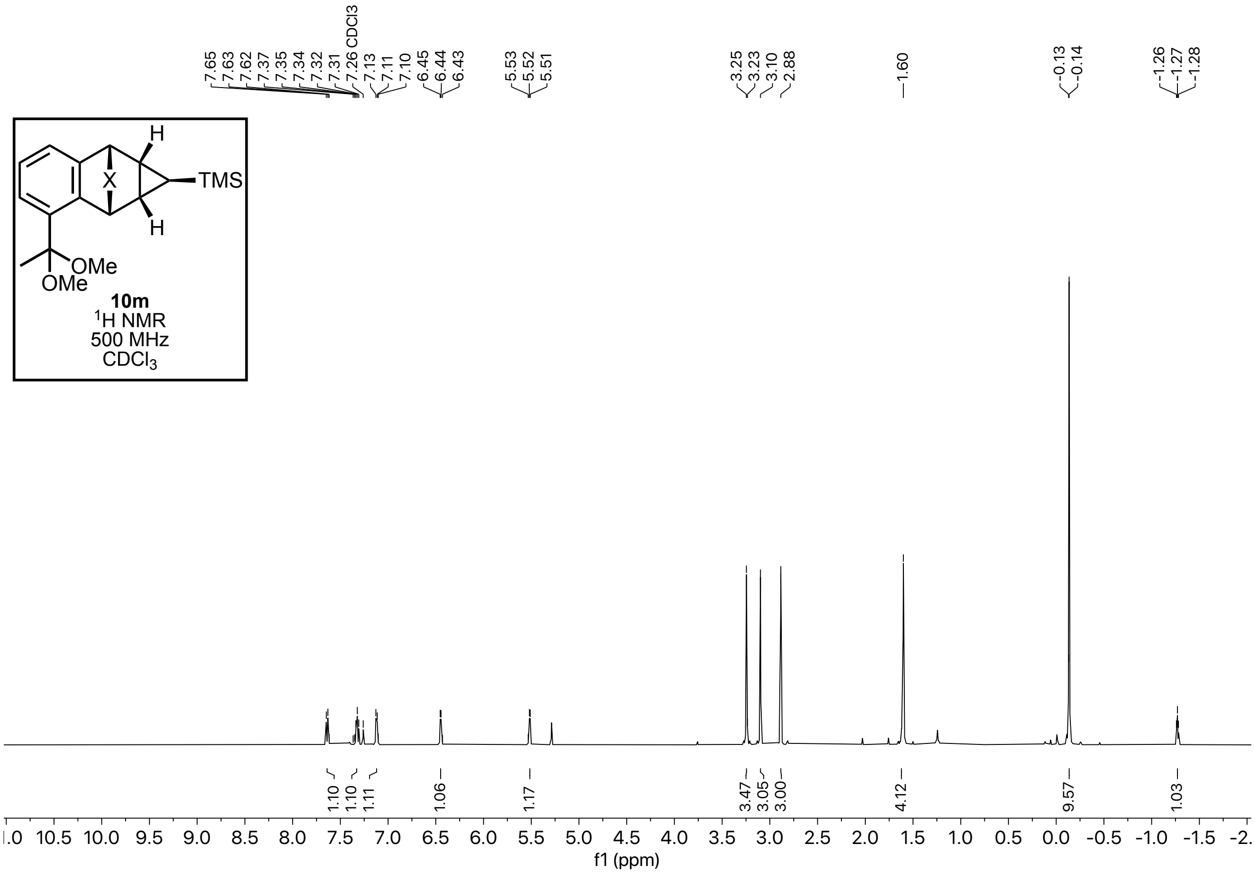
7.65
7.63
7.62
7.37
7.35
7.34
7.32
7.31
7.26 CDCl3
7.13
7.11
7.10
6.45
6.44
6.43
5.53
5.52
5.51
3.25
3.23
3.10
2.88
1.60
-0.13
-0.14
-1.26
-1.27
-1.28
H
X
TMS
H
OMe
OMe
10m
$^{1}$H NMR
500 MHz
$CDCl_3$
1.10
1.10
1.11
1.06
1.17
3.47
3.05
3.00
4.12
9.57
1.03
f1 (ppm)

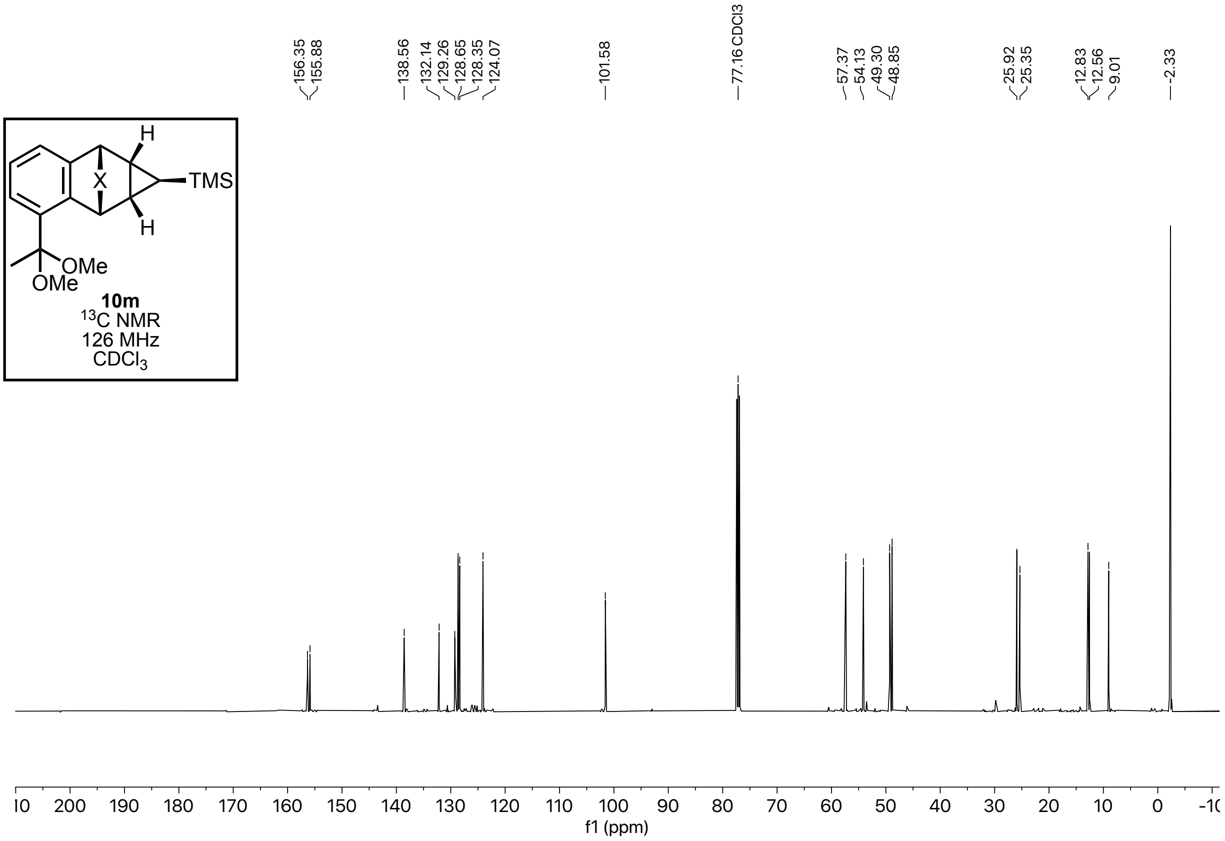
156.35
155.88
138.56
132.14
129.26
128.65
128.35
124.07
101.58
77.16 CDCl3
57.37
54.13
49.30
48.85
25.92
25.35
12.83
12.56
9.01
-2.33
H
X
TMS
H
OMe
OMe
10m
$^{13}$C NMR
126 MHz
$CDCl_3$
f1 (ppm)

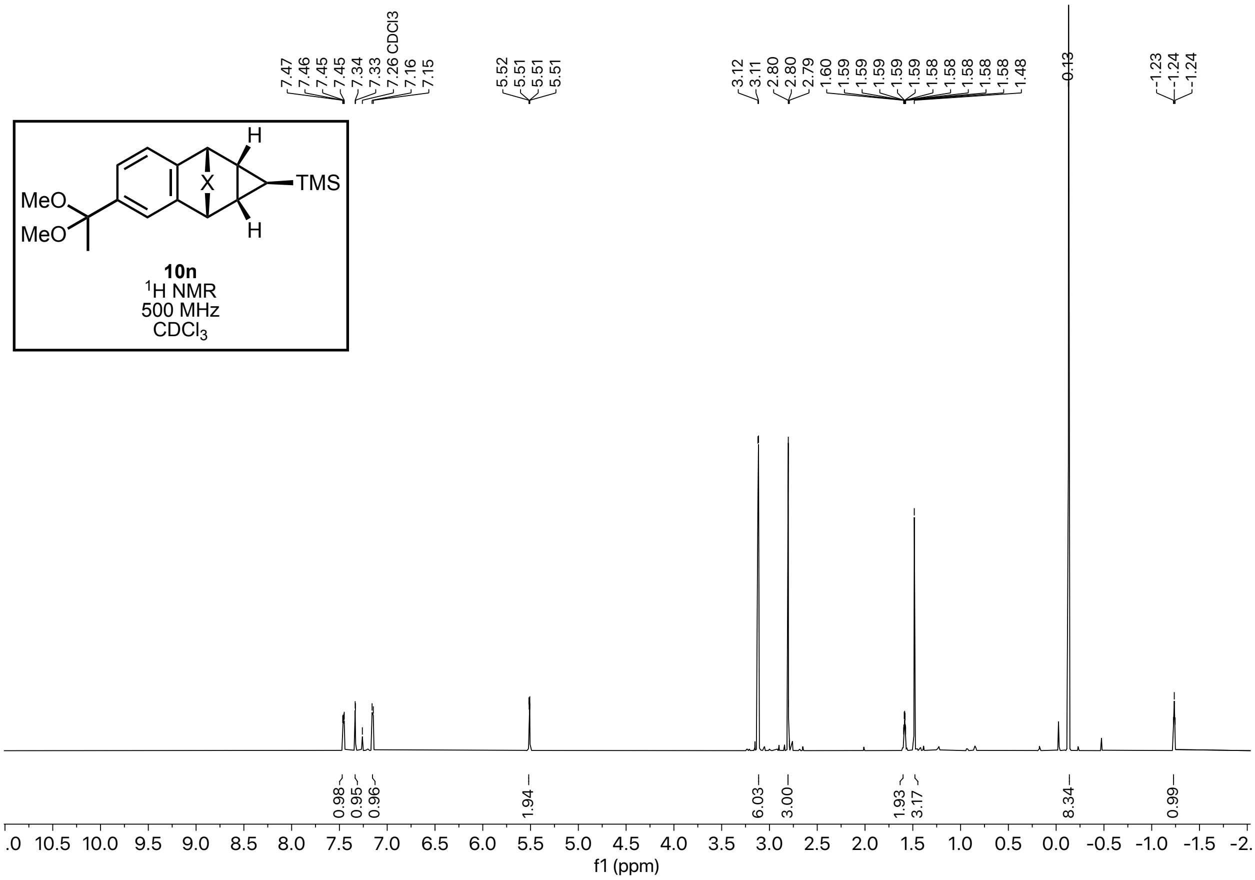
7.47
7.46
7.45
7.45
7.34
7.33
7.26 CDCl3
7.16
7.15
5.52
5.51
5.51
5.51
3.12
3.11
2.80
2.80
2.79
1.60
1.59
1.59
1.59
1.59
1.59
1.58
1.58
1.58
1.58
1.58
1.48
0.13
-1.23
-1.24
-1.24
MeO
MeO
TMS
H
H
X
10n
1H NMR
500 MHz
CDCl3
0.98
0.95
0.96
1.94
6.03
3.00
1.93
3.17
8.34
0.99
f1 (ppm)

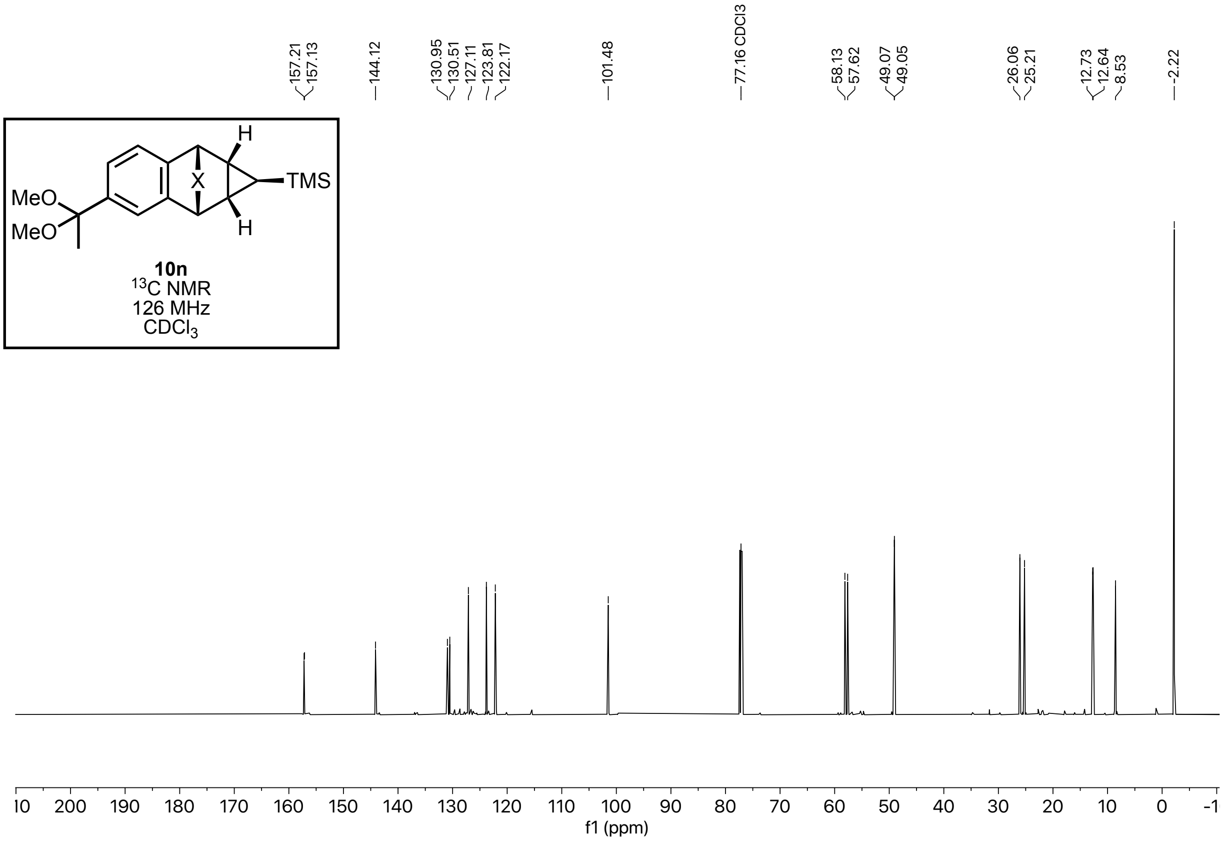
157.21
157.13
144.12
130.95
130.51
127.11
123.81
122.17
101.48
77.16 CDCl3
58.13
57.62
49.07
49.05
26.06
25.21
12.73
12.64
8.53
-2.22
MeO
MeO
TMS
H
H
X
10n
13C NMR
126 MHz
CDCl3
f1 (ppm)

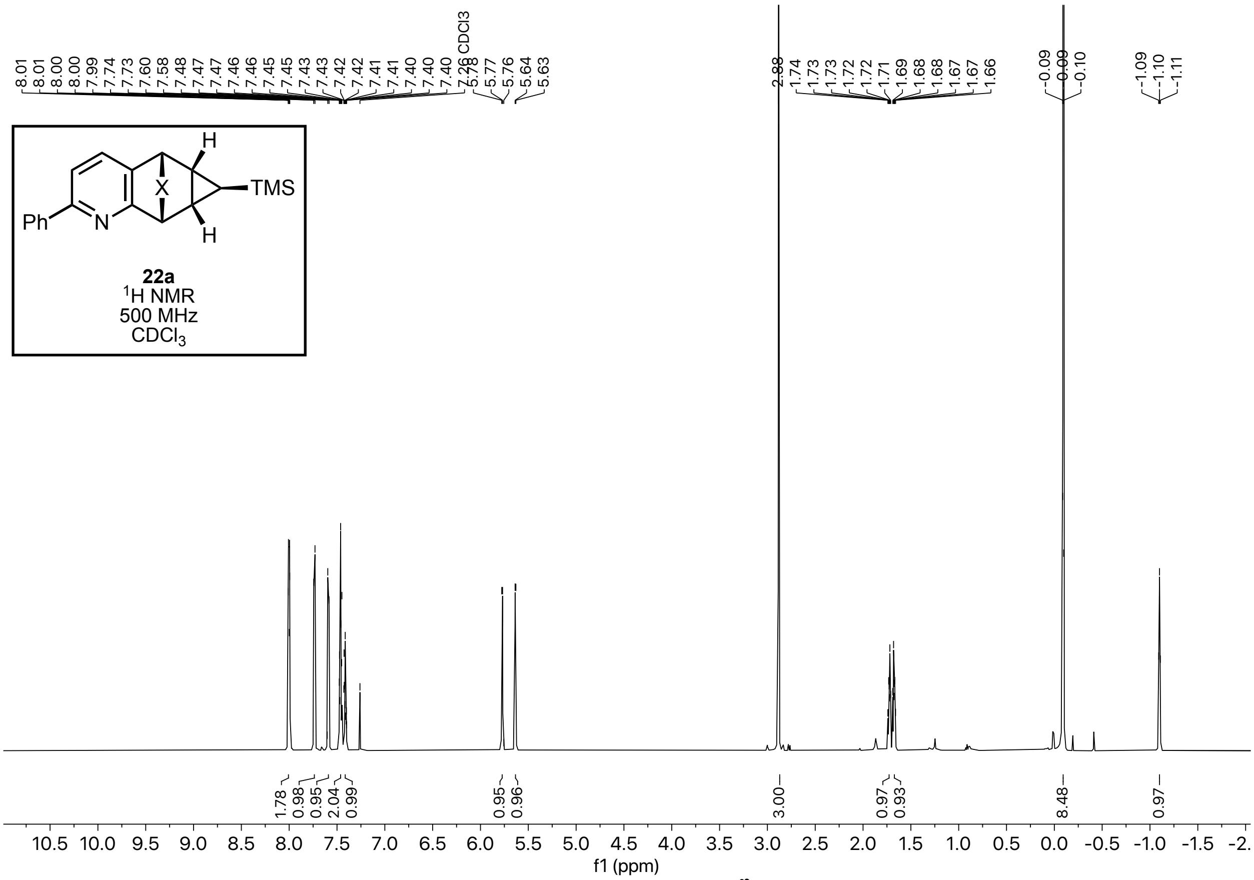
22a
$^1$H NMR
500 MHz
$CDCl_3$
f1 (ppm)

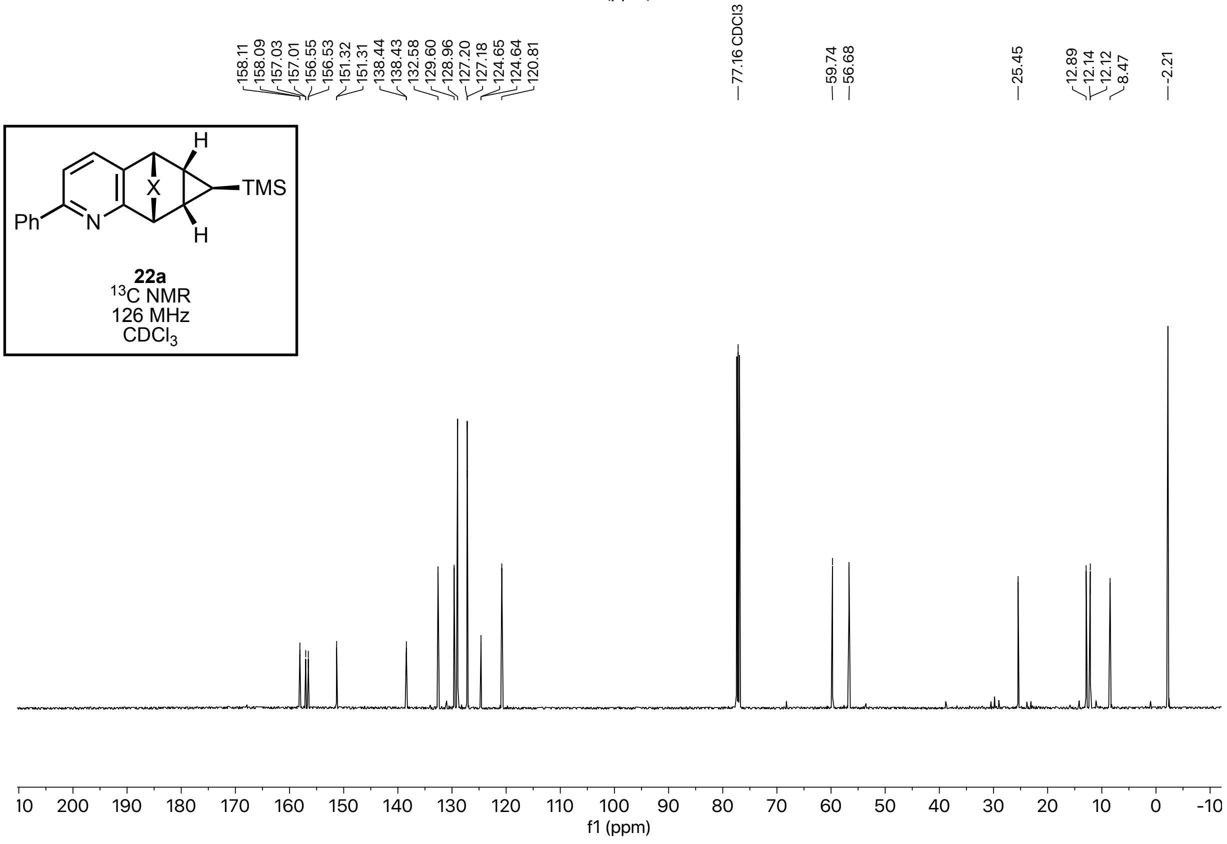
22a
$^{13}$C NMR
126 MHz
$CDCl_3$
f1 (ppm)

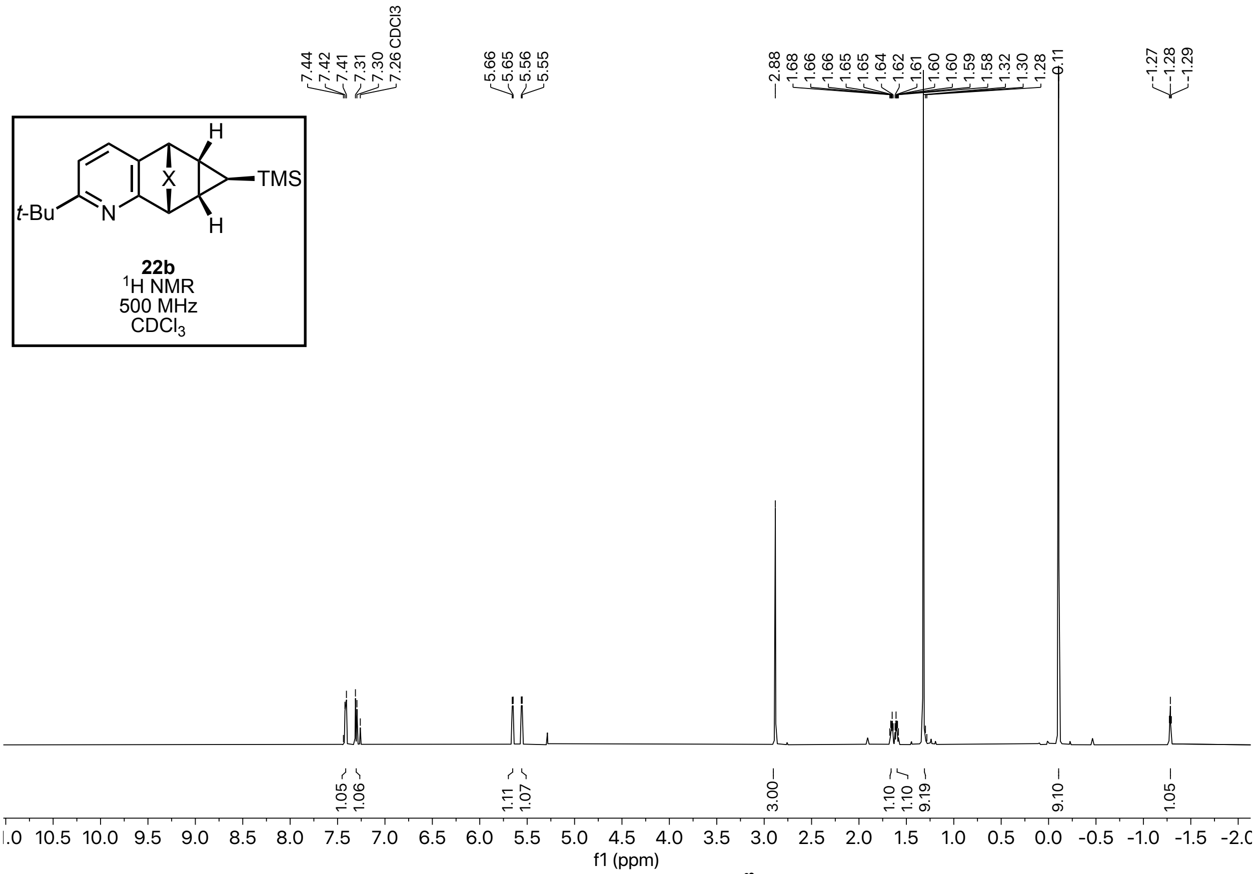
7.44
7.42
7.41
7.31
7.30
7.26 CDCl3
5.66
5.65
5.56
5.55
2.88
1.68
1.66
1.66
1.65
1.65
1.64
1.62
1.61
1.60
1.60
1.59
1.58
1.32
1.30
1.28
0.11
-1.27
-1.28
-1.29
H
TMS
X
t-Bu
N
H
22b
1H NMR
500 MHz
CDCl3
1.05
1.06
1.11
1.07
3.00
1.10
1.10
9.19
9.10
1.05
f1 (ppm)

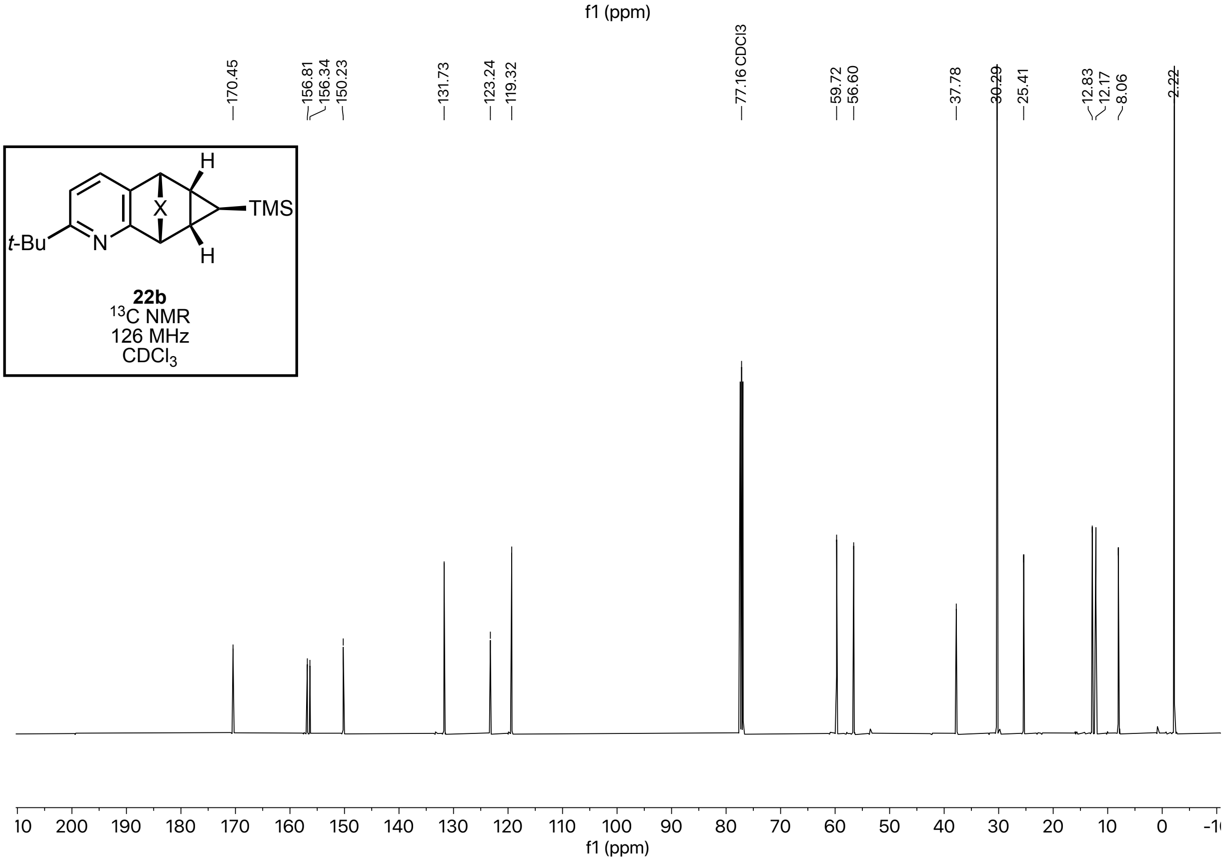
170.45
156.81
156.34
150.23
131.73
123.24
119.32
77.16 CDCl3
59.72
56.60
37.78
30.29
25.41
12.83
12.17
8.06
2.22
H
TMS
X
t-Bu
N
H
22b
13C NMR
126 MHz
CDCl3
f1 (ppm)

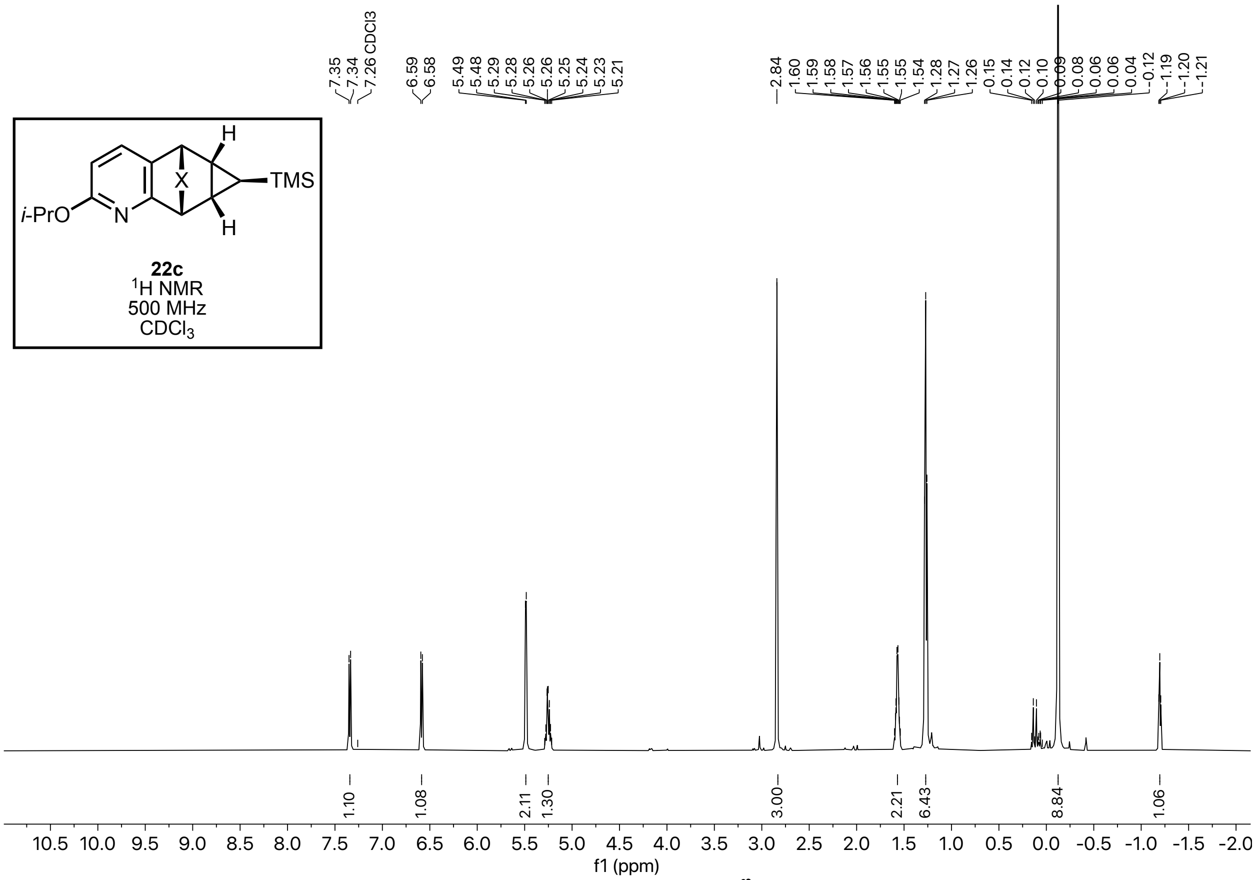
7.35
7.34
7.26 CDCl3
6.59
6.58
5.49
5.48
5.29
5.28
5.26
5.26
5.25
5.24
5.23
5.21
2.84
1.60
1.59
1.58
1.57
1.56
1.55
1.55
1.54
1.28
1.27
1.26
0.15
0.14
0.12
0.10
0.09
0.08
0.06
0.06
0.04
-0.12
-1.19
-1.20
-1.21
H
X
TMS
i-PrO
N
H
22c
1H NMR
500 MHz
CDCl3
1.10
1.08
2.11
1.30
3.00
2.21
6.43
8.84
1.06
10.5 10.0 9.5 9.0 8.5 8.0 7.5 7.0 6.5 6.0 5.5 5.0 4.5 4.0 3.5 3.0 2.5 2.0 1.5 1.0 0.5 0.0 -0.5 -1.0 -1.5 -2.0
f1 (ppm)

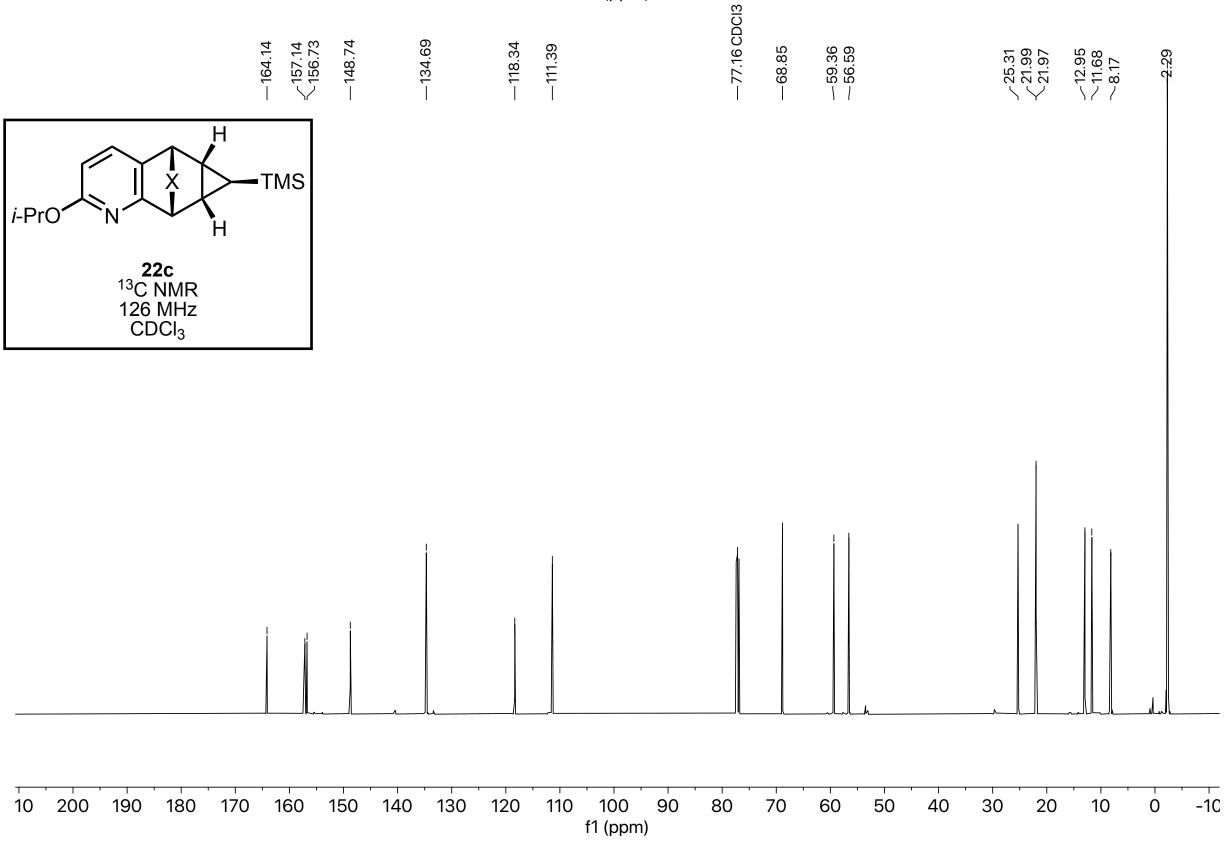
164.14
157.14
156.73
148.74
134.69
118.34
111.39
77.16 CDCl3
68.85
59.36
56.59
25.31
21.99
21.97
12.95
11.68
8.17
-2.29
H
X
TMS
i-PrO
N
H
22c
13C NMR
126 MHz
CDCl3
10 200 190 180 170 160 150 140 130 120 110 100 90 80 70 60 50 40 30 20 10 0 -10
f1 (ppm)

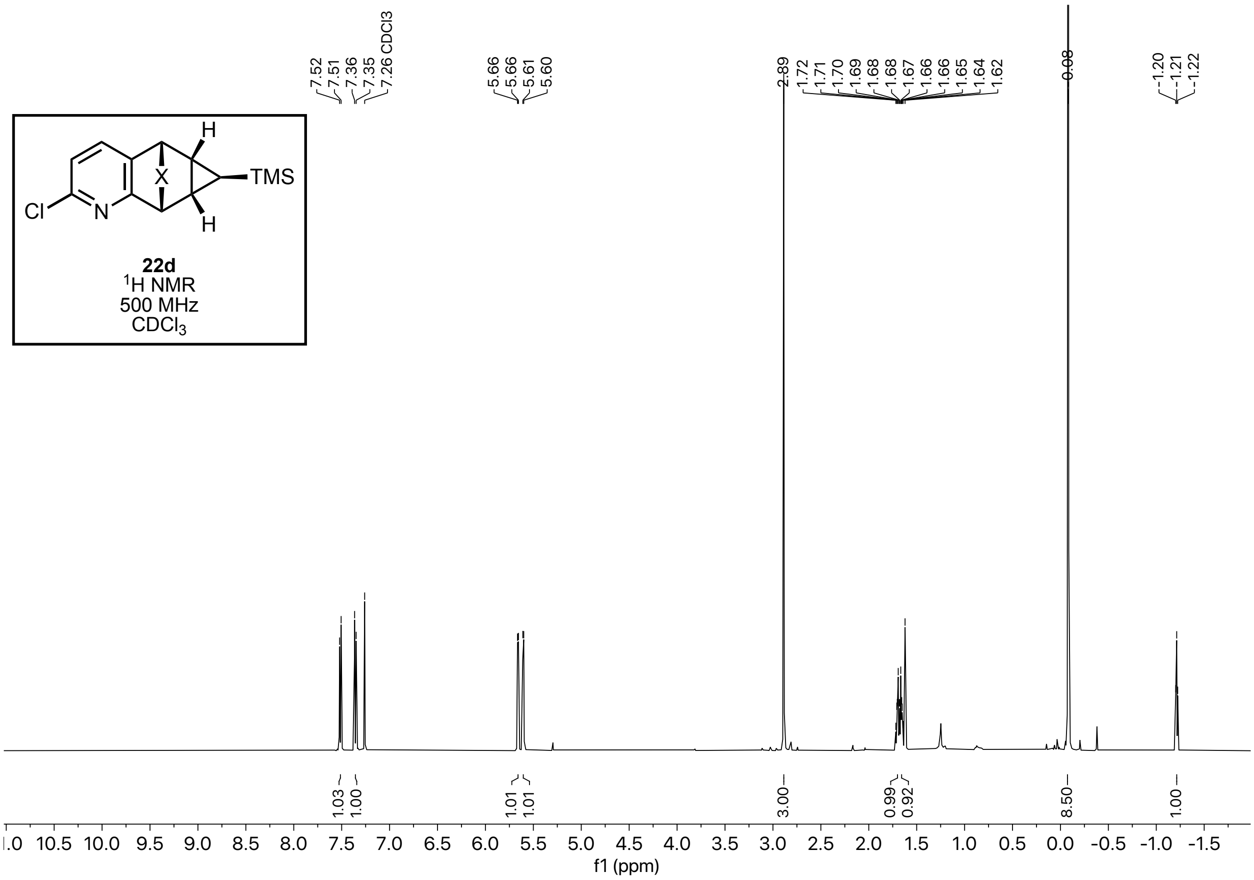
7.52
7.51
7.36
7.35
7.26 CDCl3
5.66
5.66
5.61
5.60
2.89
1.72
1.71
1.70
1.69
1.68
1.68
1.67
1.66
1.66
1.65
1.64
1.62
0.08
-1.20
-1.21
-1.22
H
X
TMS
Cl
N
H
22d
1H NMR
500 MHz
CDCl3
1.03
1.00
1.01
1.01
3.00
0.99
0.92
8.50
1.00
f1 (ppm)

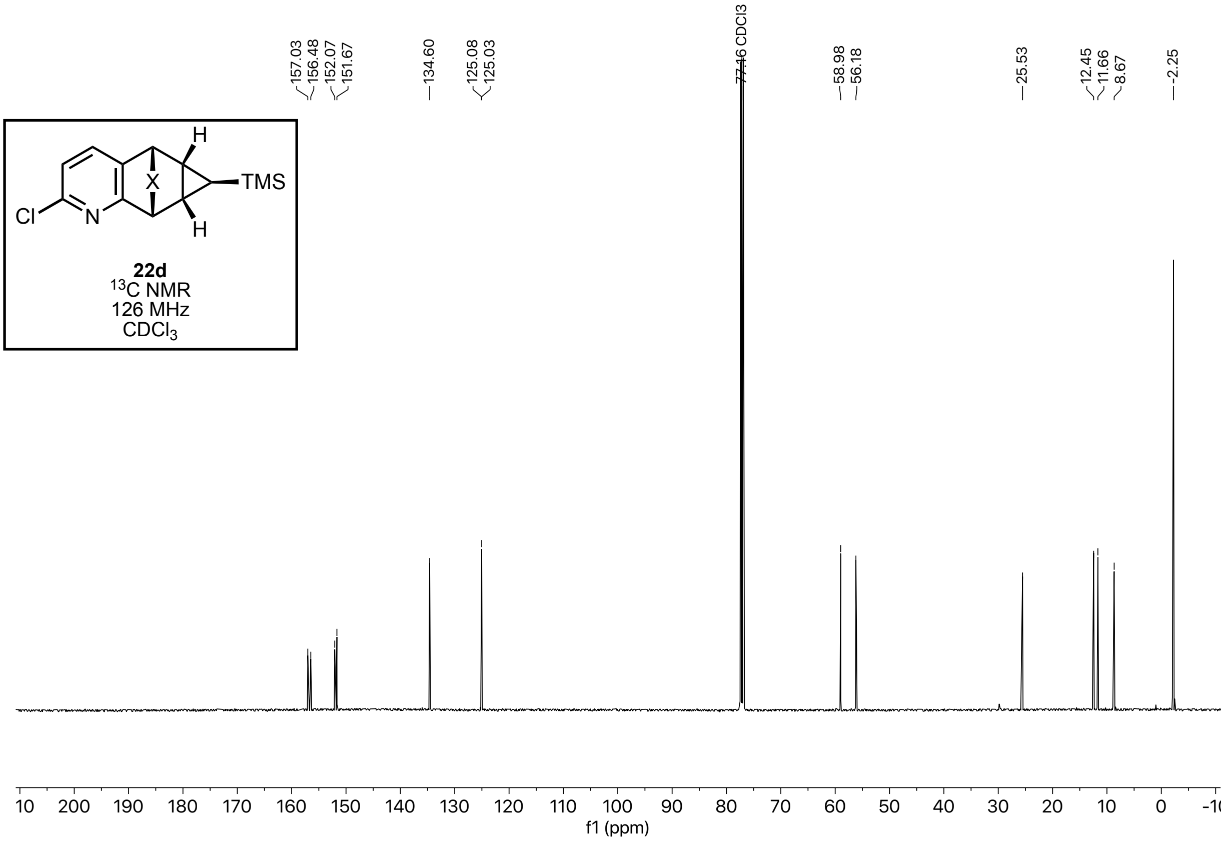
157.03
156.48
152.07
151.67
134.60
125.08
125.03
77.16 CDCl3
58.98
56.18
25.53
12.45
11.66
8.67
-2.25
H
X
TMS
Cl
N
H
22d
13C NMR
126 MHz
CDCl3
f1 (ppm)

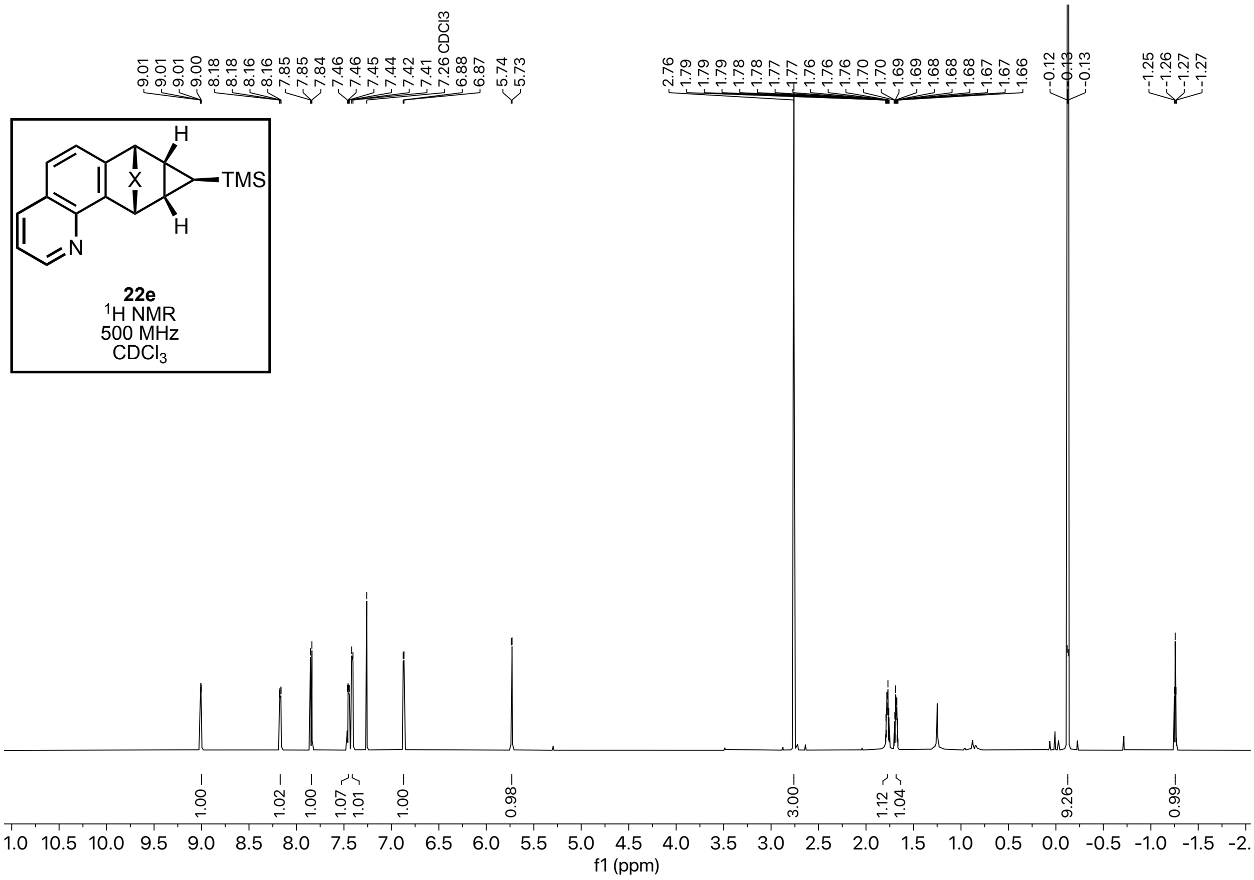
9.01
9.01
9.01
9.00
8.18
8.18
8.16
8.16
7.85
7.84
7.46
7.46
7.45
7.44
7.42
7.41
7.26 CDCl3
6.88
6.87
5.74
5.73
2.76
1.79
1.79
1.79
1.78
1.78
1.77
1.77
1.76
1.76
1.76
1.70
1.70
1.69
1.69
1.68
1.68
1.68
1.67
1.67
1.66
-0.12
-0.13
-0.13
-1.25
-1.26
-1.27
-1.27
H
X
TMS
H
N
22e
¹H NMR
500 MHz
CDCl3
1.00
1.02
1.00
1.07
1.01
1.00
0.98
3.00
1.12
1.04
9.26
0.99
f1 (ppm)

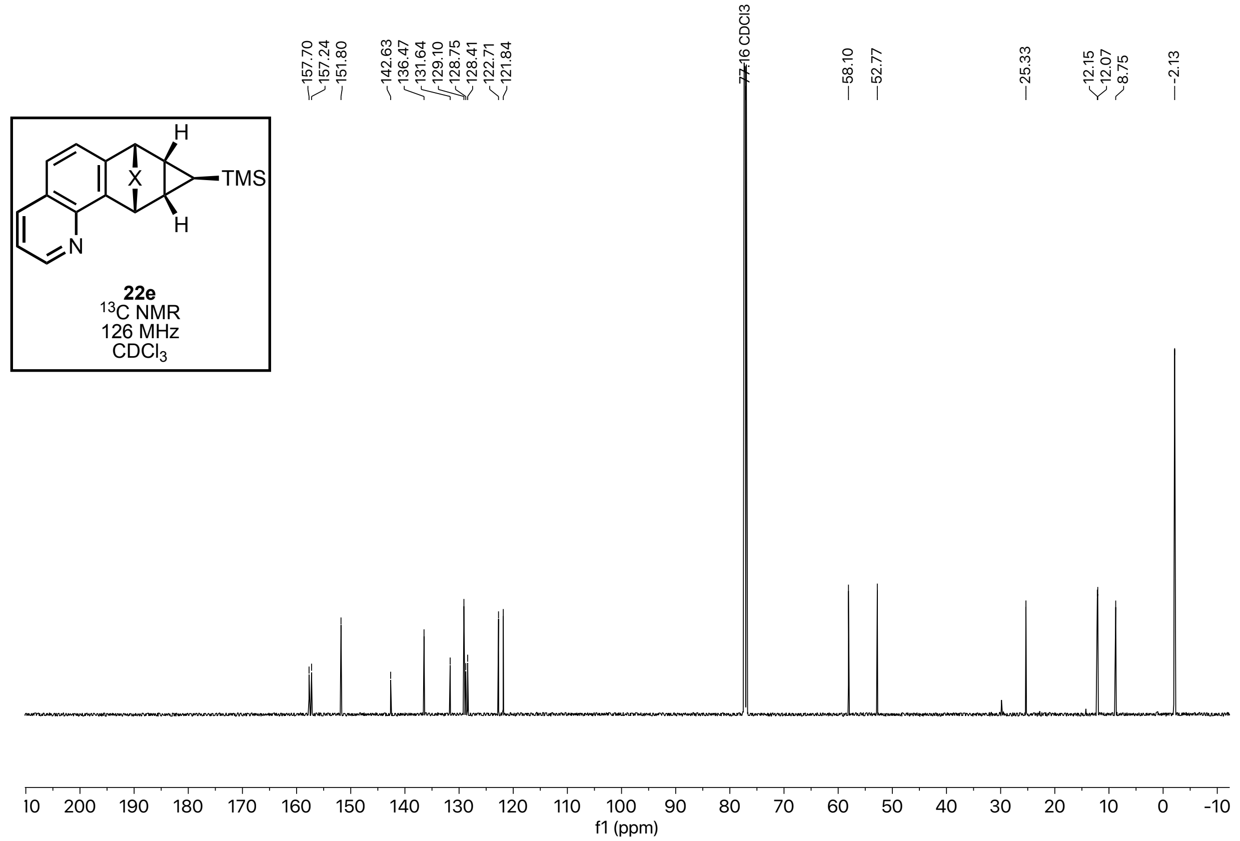
157.70
157.24
151.80
142.63
136.47
131.64
129.10
128.75
128.41
122.71
121.84
77.16 CDCl3
58.10
52.77
25.33
12.15
12.07
8.75
-2.13
H
X
TMS
H
N
22e
¹³C NMR
126 MHz
CDCl3
f1 (ppm)

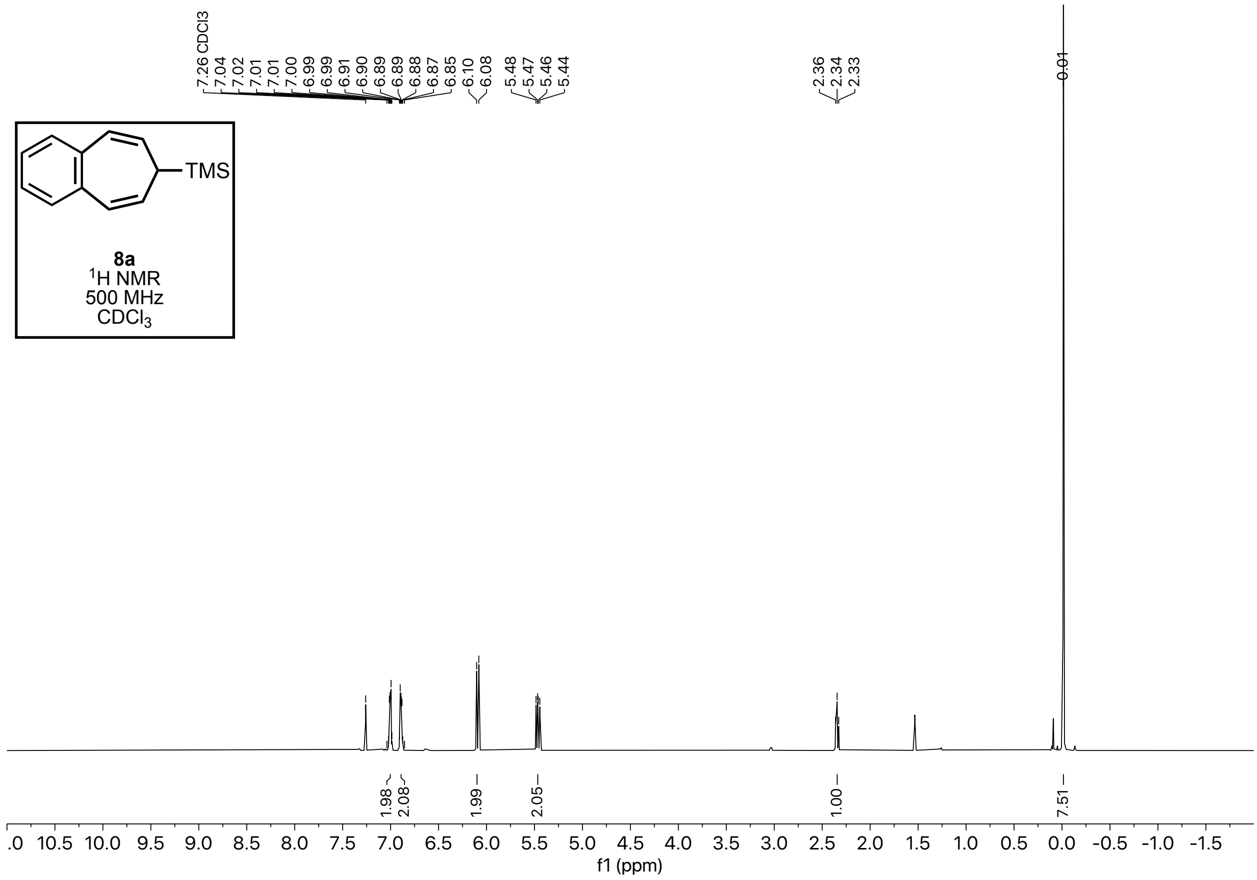
7.26 CDCl3
7.04
7.02
7.01
7.01
7.00
6.99
6.99
6.91
6.90
6.89
6.89
6.88
6.87
6.85
6.10
6.08
5.48
5.47
5.46
5.44
2.36
2.34
2.33
0.01
TMS
8a
$^{1}$H NMR
500 MHz
$CDCl_3$
1.98
2.08
1.99
2.05
1.00
7.51
f1 (ppm)

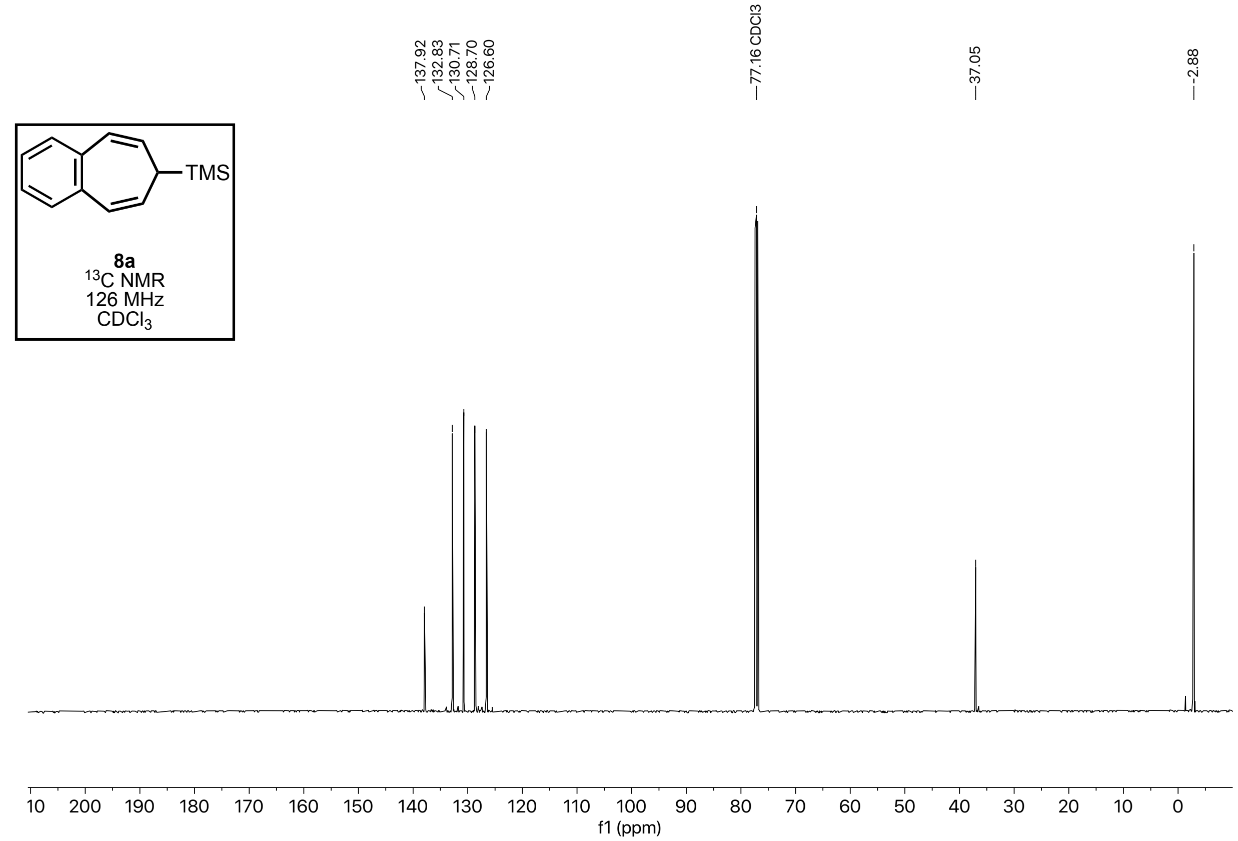
137.92
132.83
130.71
128.70
126.60
77.16 CDCl3
37.05
-2.88
TMS
8a
$^{13}$C NMR
126 MHz
$CDCl_3$
f1 (ppm)

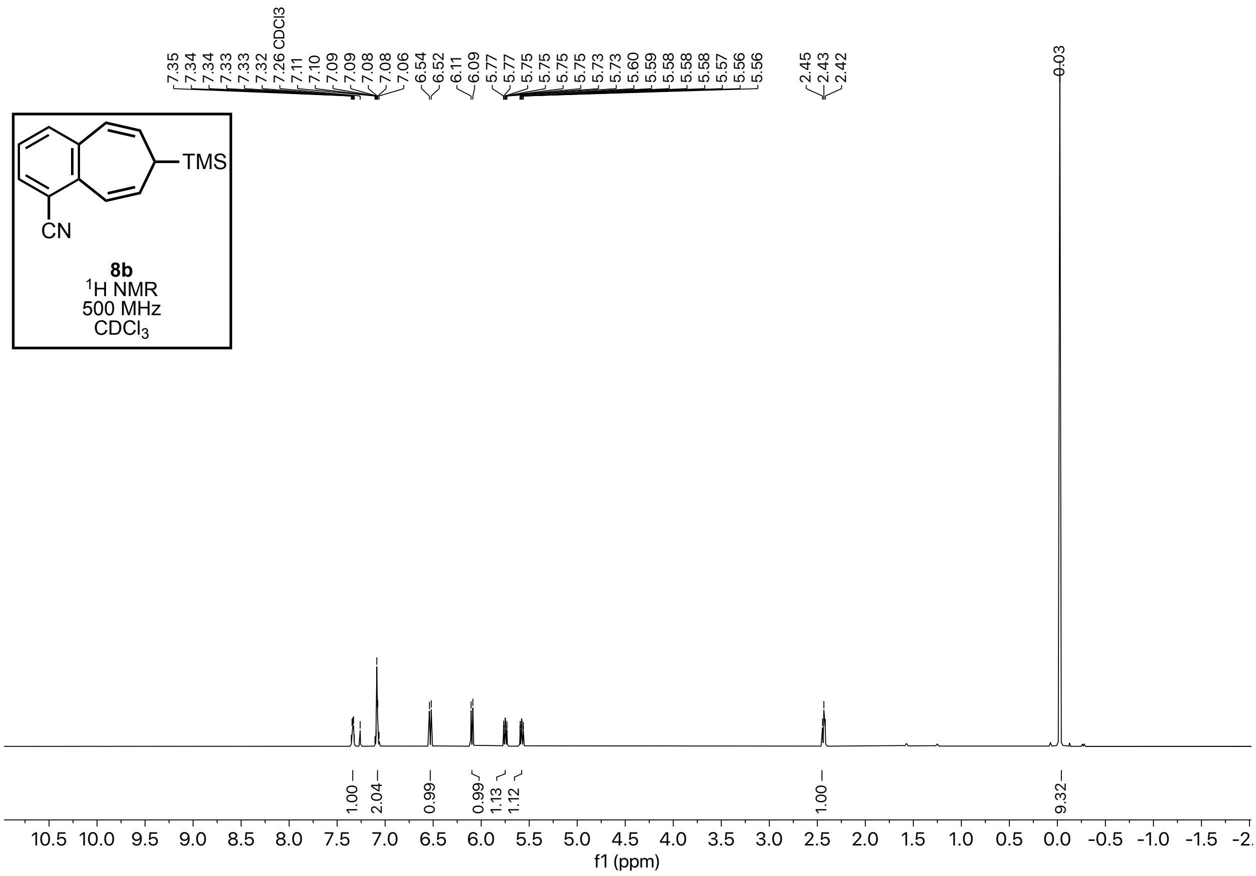
7.35
7.34
7.34
7.33
7.33
7.32
7.26 CDCl3
7.11
7.10
7.09
7.09
7.08
7.08
7.06
6.54
6.52
6.11
6.09
5.77
5.77
5.75
5.75
5.75
5.75
5.73
5.73
5.60
5.59
5.58
5.58
5.58
5.57
5.56
5.56
2.45
2.43
2.42
0.03
TMS
CN
8b
1H NMR
500 MHz
CDCl3
1.00
2.04
0.99
0.99
1.13
1.12
1.00
9.32
f1 (ppm)

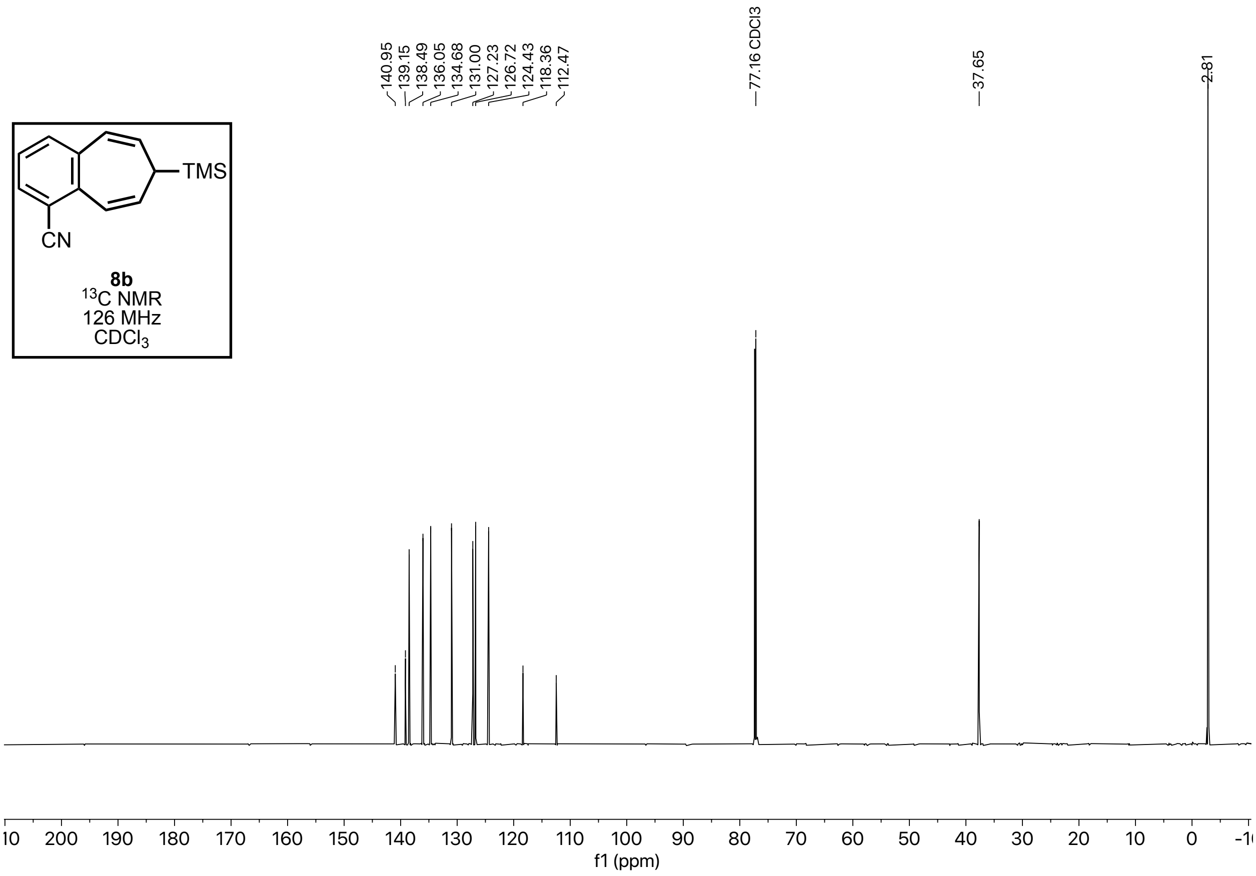
140.95
139.15
138.49
136.05
134.68
131.00
127.23
126.72
124.43
118.36
112.47
77.16 CDCl3
37.65
-2.81
TMS
CN
8b
13C NMR
126 MHz
CDCl3
f1 (ppm)

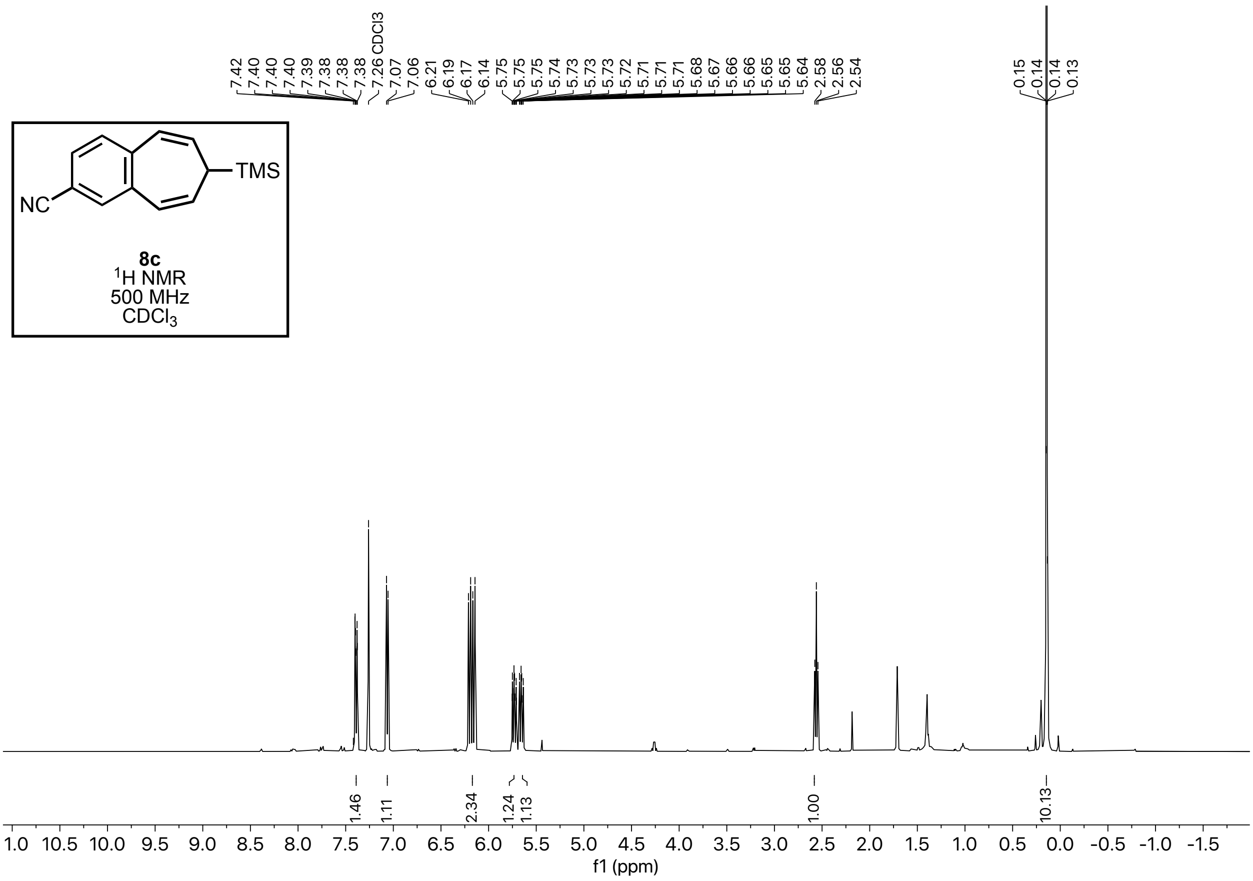
7.42 7.40 7.40 7.40 7.39 7.38 7.38 7.38 7.26 CDCl3 7.07 7.06 6.21 6.19 6.17 6.14 5.75 5.75 5.75 5.74 5.73 5.73 5.73 5.72 5.71 5.71 5.71 5.68 5.67 5.66 5.66 5.65 5.65 5.64 2.58 2.56 2.54
0.15 0.14 0.14 0.13
TMS
NC
8c
1H NMR
500 MHz
CDCl3
1.46 1.11 2.34 1.24 1.13 1.00 10.13
f1 (ppm)

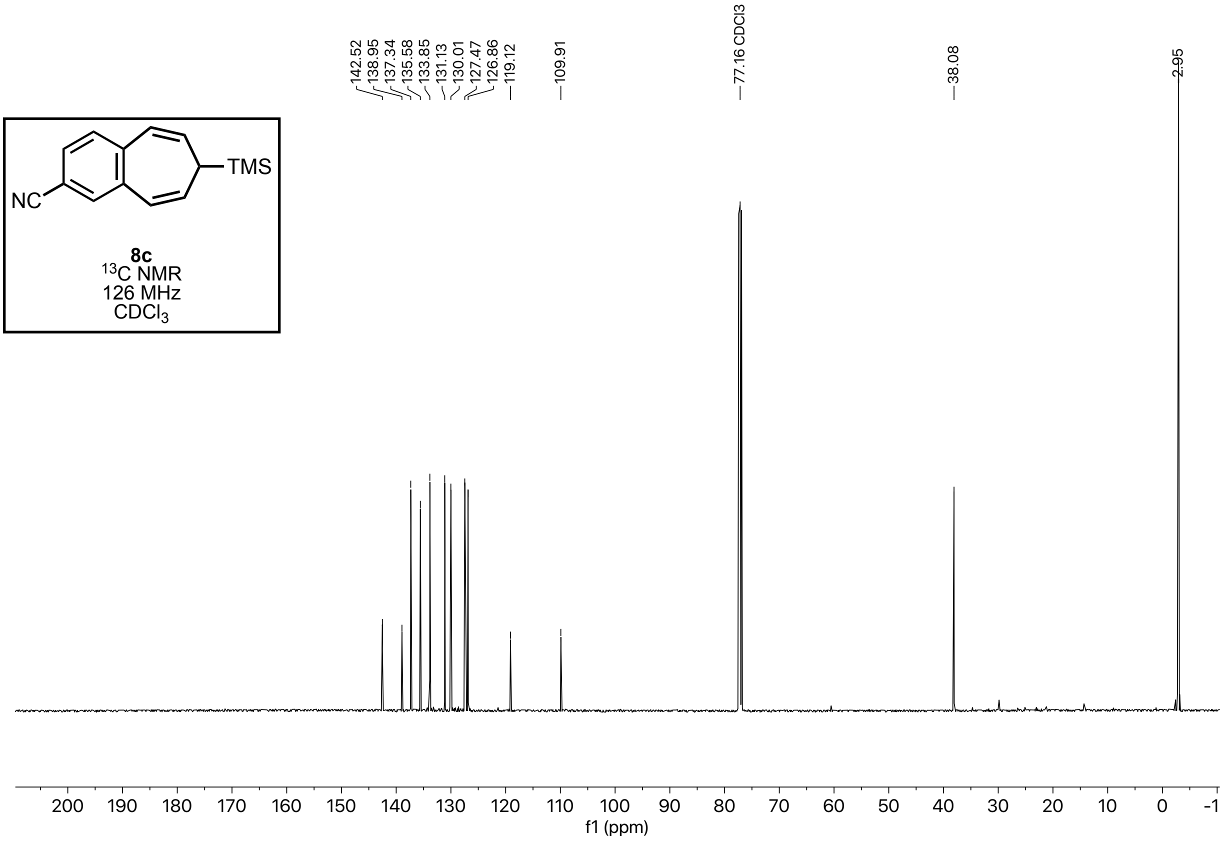
142.52 138.95 137.34 135.58 133.85 131.13 130.01 127.47 126.86 119.12 109.91
77.16 CDCl3
38.08
-2.95
TMS
NC
8c
13C NMR
126 MHz
CDCl3
f1 (ppm)

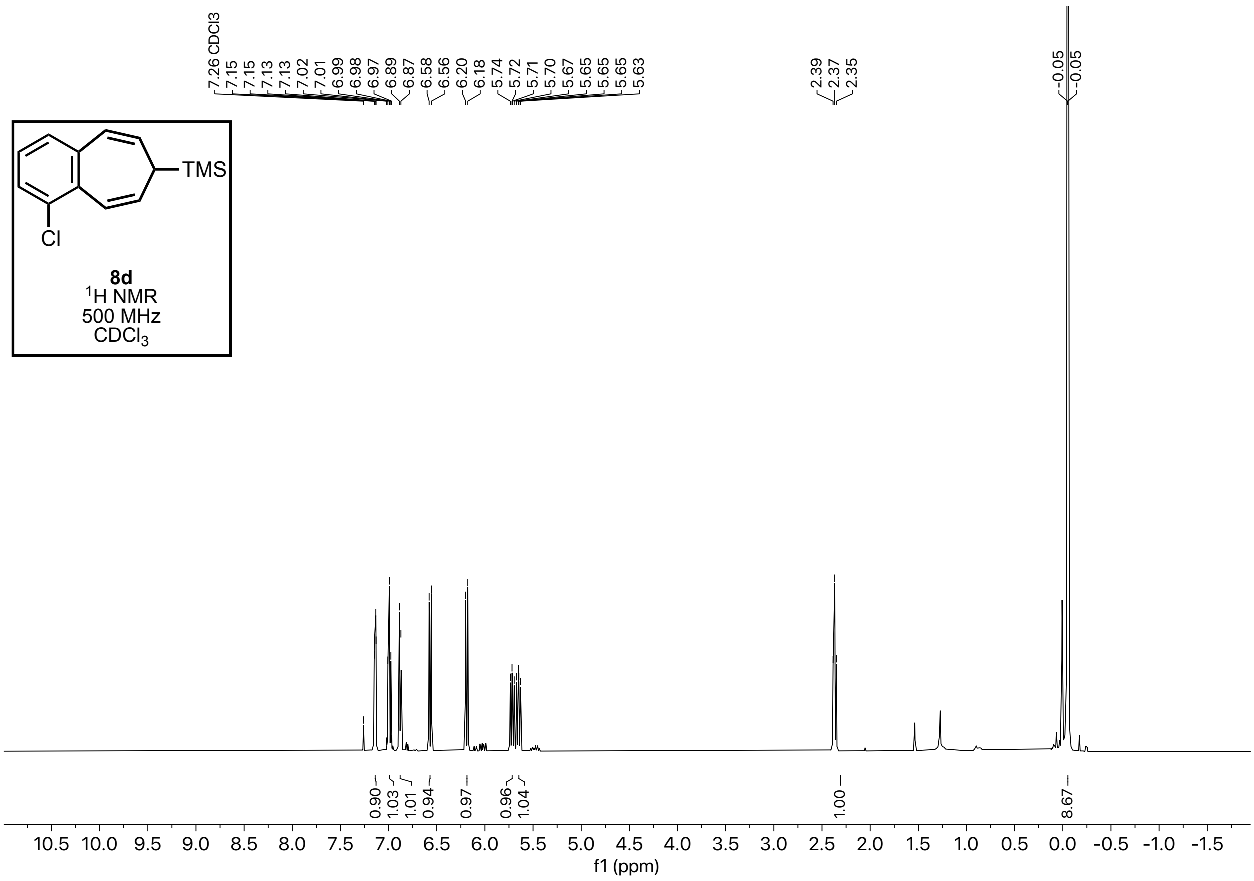
TMS
Cl
8d
$^1$H NMR
500 MHz
$CDCl_3$
7.26 CDCl3
7.15
7.15
7.13
7.13
7.02
7.01
6.99
6.98
6.97
6.89
6.87
6.58
6.56
6.20
6.18
5.74
5.72
5.71
5.70
5.67
5.65
5.65
5.65
5.63
2.39
2.37
2.35
-0.05
-0.05
0.90
1.03
1.01
0.94
0.97
0.96
1.04
1.00
8.67
f1 (ppm)

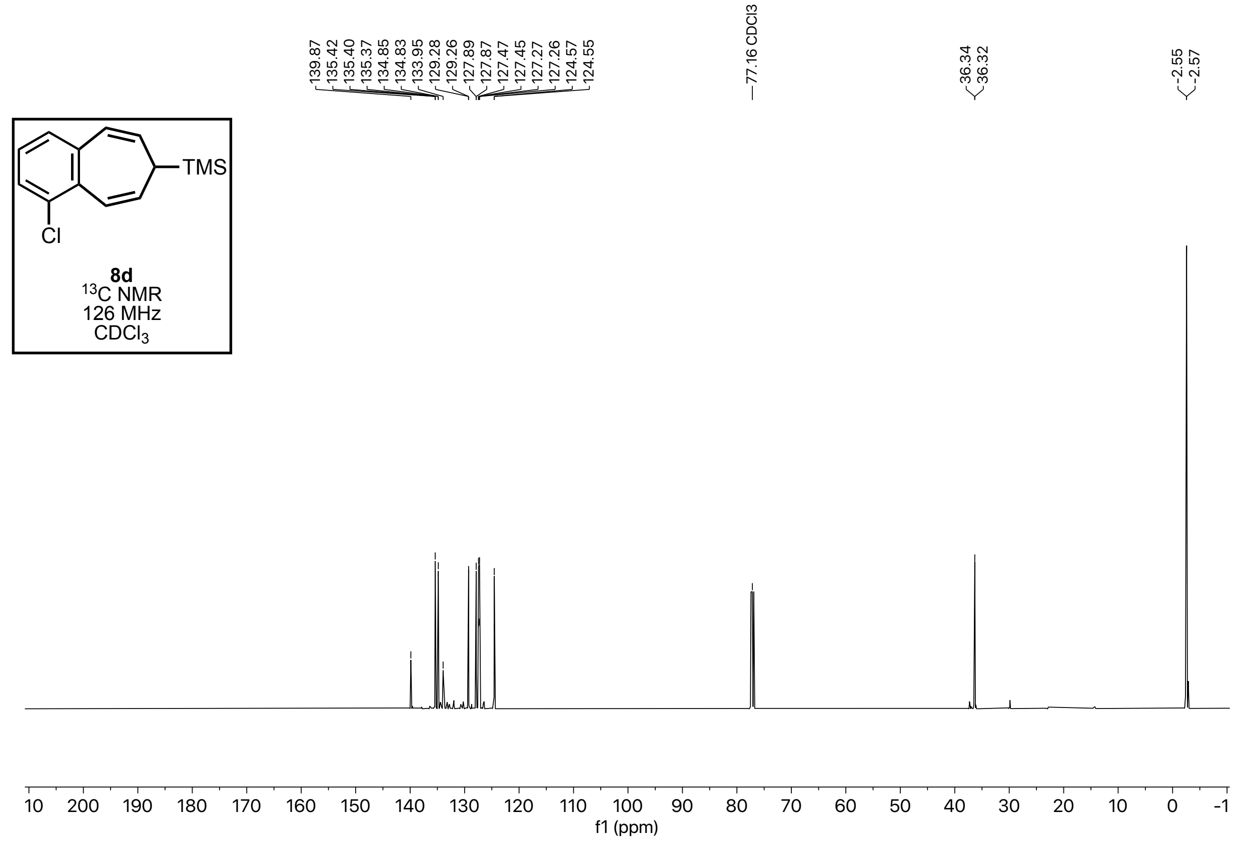
TMS
Cl
8d
$^{13}$C NMR
126 MHz
$CDCl_3$
139.87
135.42
135.40
135.37
134.85
134.83
133.95
129.28
129.26
127.89
127.87
127.47
127.45
127.27
127.26
124.57
124.55
77.16 CDCl3
36.34
36.32
-2.55
-2.57
f1 (ppm)

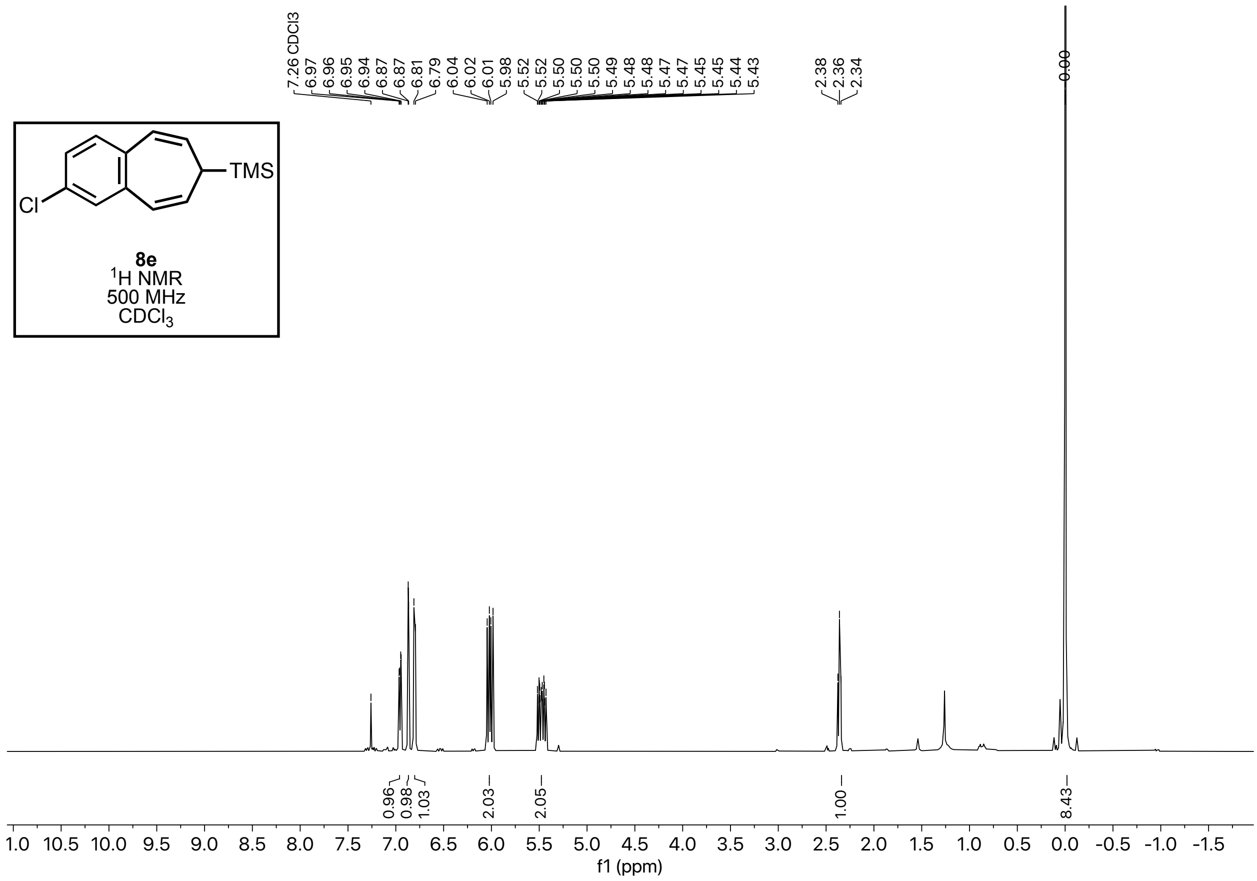
7.26 CDCl3
6.97
6.96
6.95
6.94
6.87
6.87
6.81
6.79
6.04
6.02
6.01
5.98
5.52
5.52
5.50
5.50
5.50
5.49
5.48
5.48
5.47
5.47
5.45
5.45
5.44
5.43
2.38
2.36
2.34
0.00
Cl
TMS
8e
1H NMR
500 MHz
CDCl3
0.96
0.98
1.03
2.03
2.05
1.00
8.43
f1 (ppm)

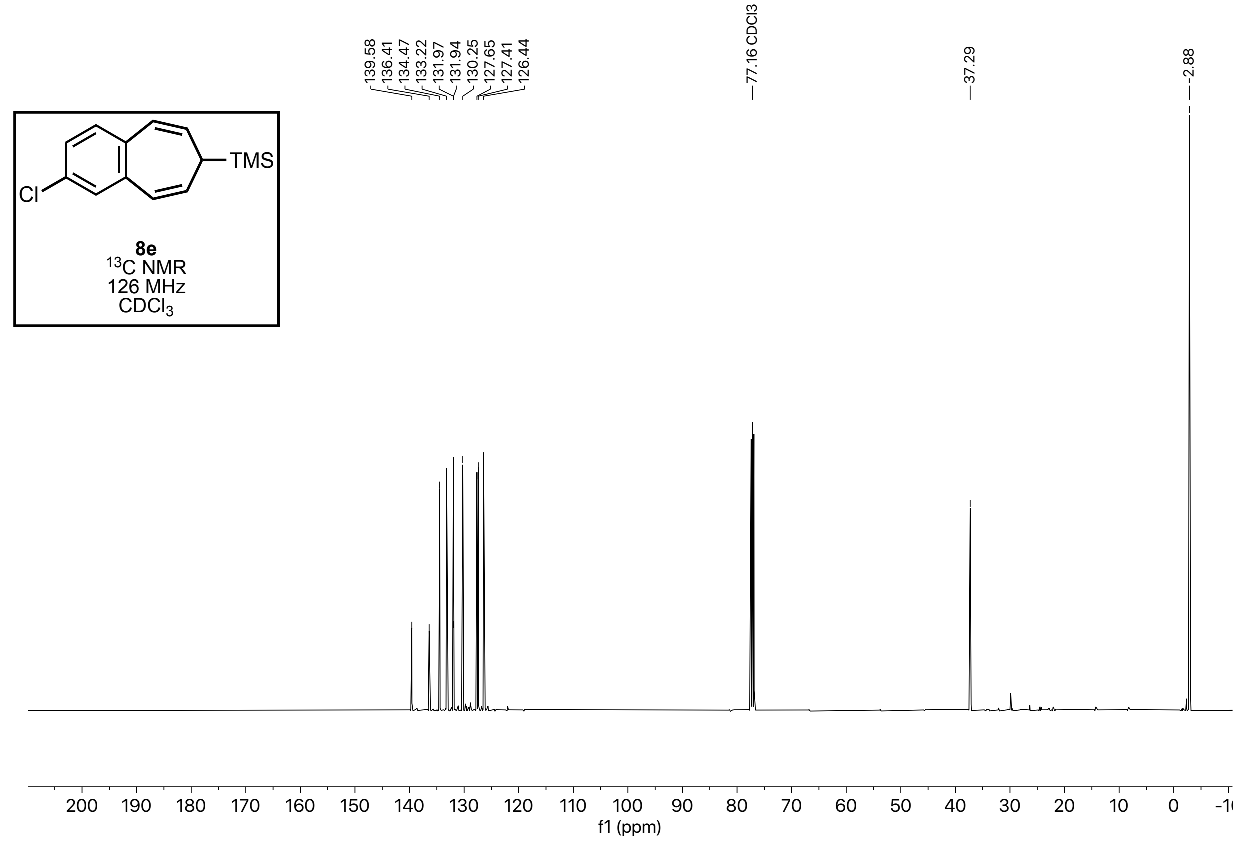
139.58
136.41
134.47
133.22
131.97
131.94
130.25
127.65
127.41
126.44
77.16 CDCl3
37.29
-2.88
Cl
TMS
8e
13C NMR
126 MHz
CDCl3
f1 (ppm)

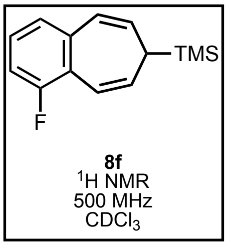
TMS
F
8f
$^1$H NMR
500 MHz
$CDCl_3$

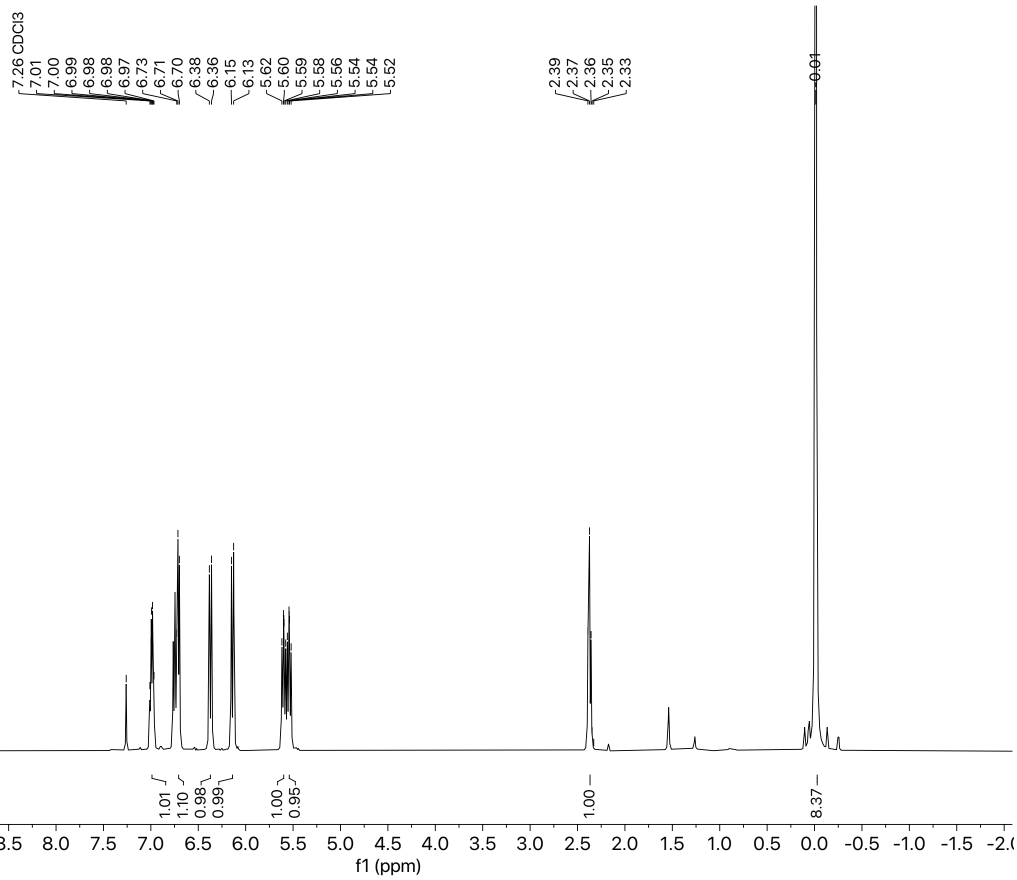

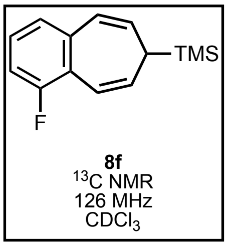
TMS
F
8f
$^{13}$C NMR
126 MHz
$CDCl_3$

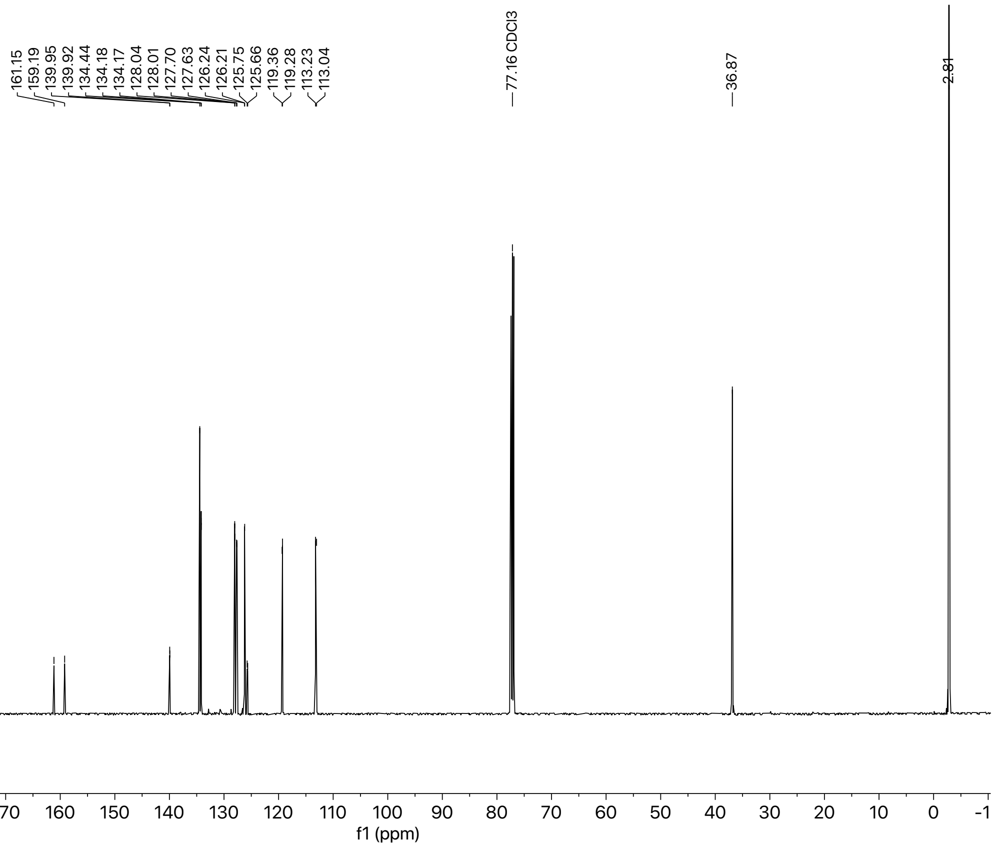

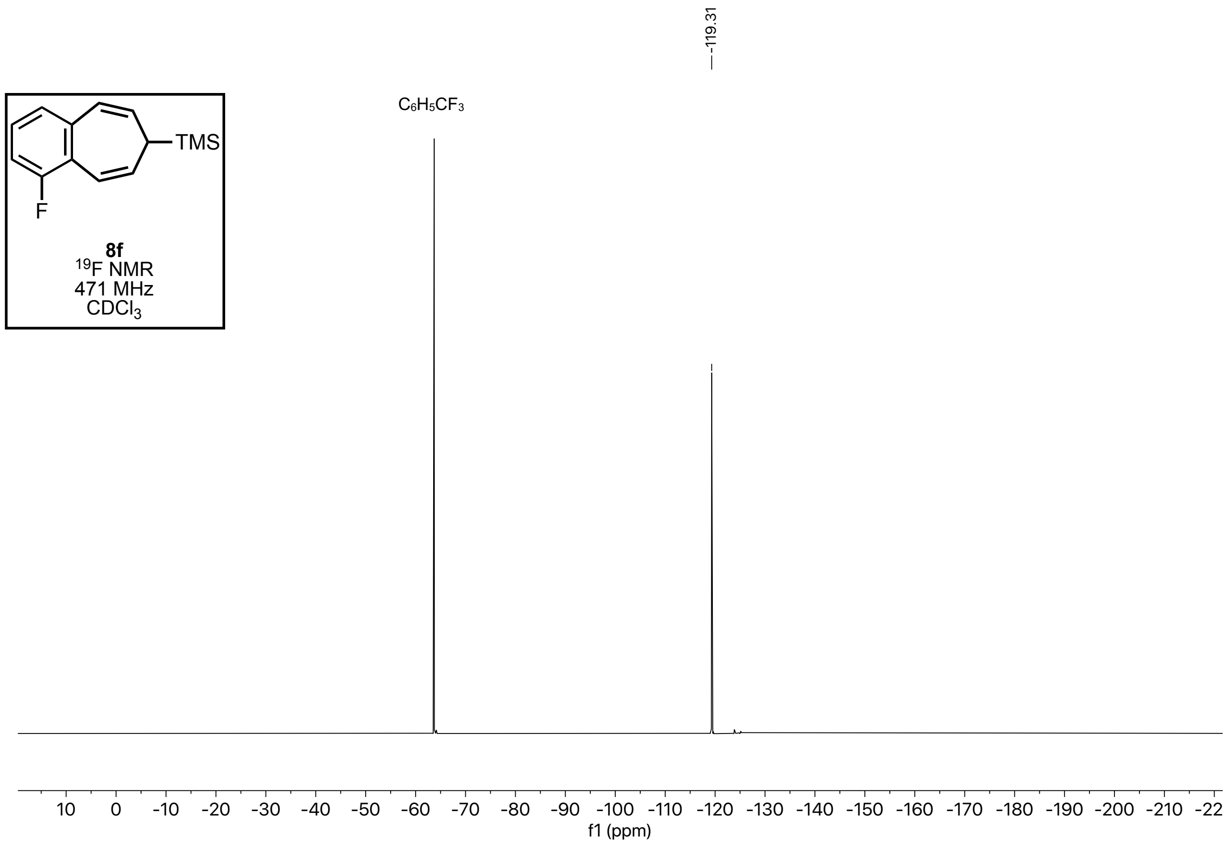
8f
19F NMR
471 MHz
CDCl3
TMS
F
C6H5CF3
-119.31
f1 (ppm)

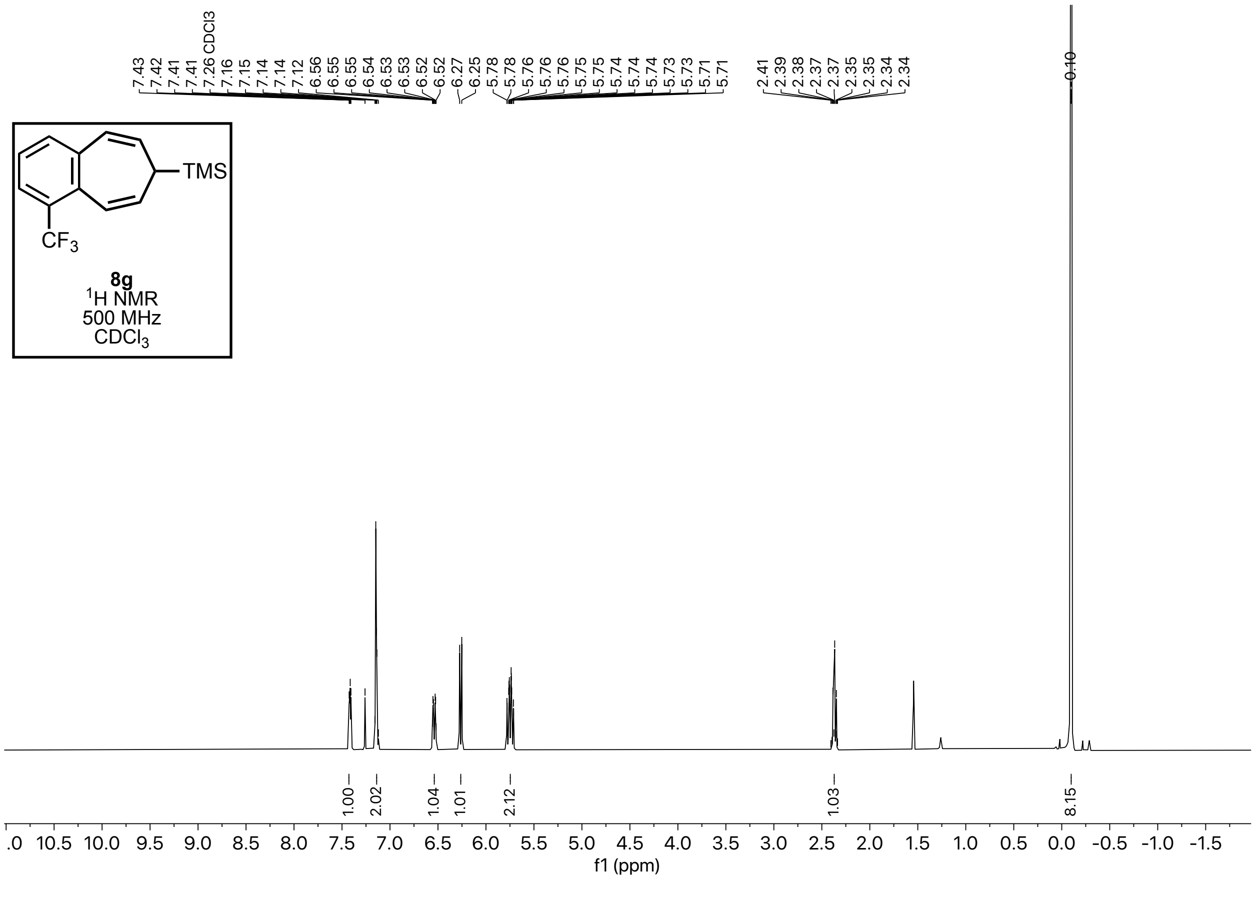
8g
1H NMR
500 MHz
CDCl3
TMS
CF3
CDCl3
f1 (ppm)

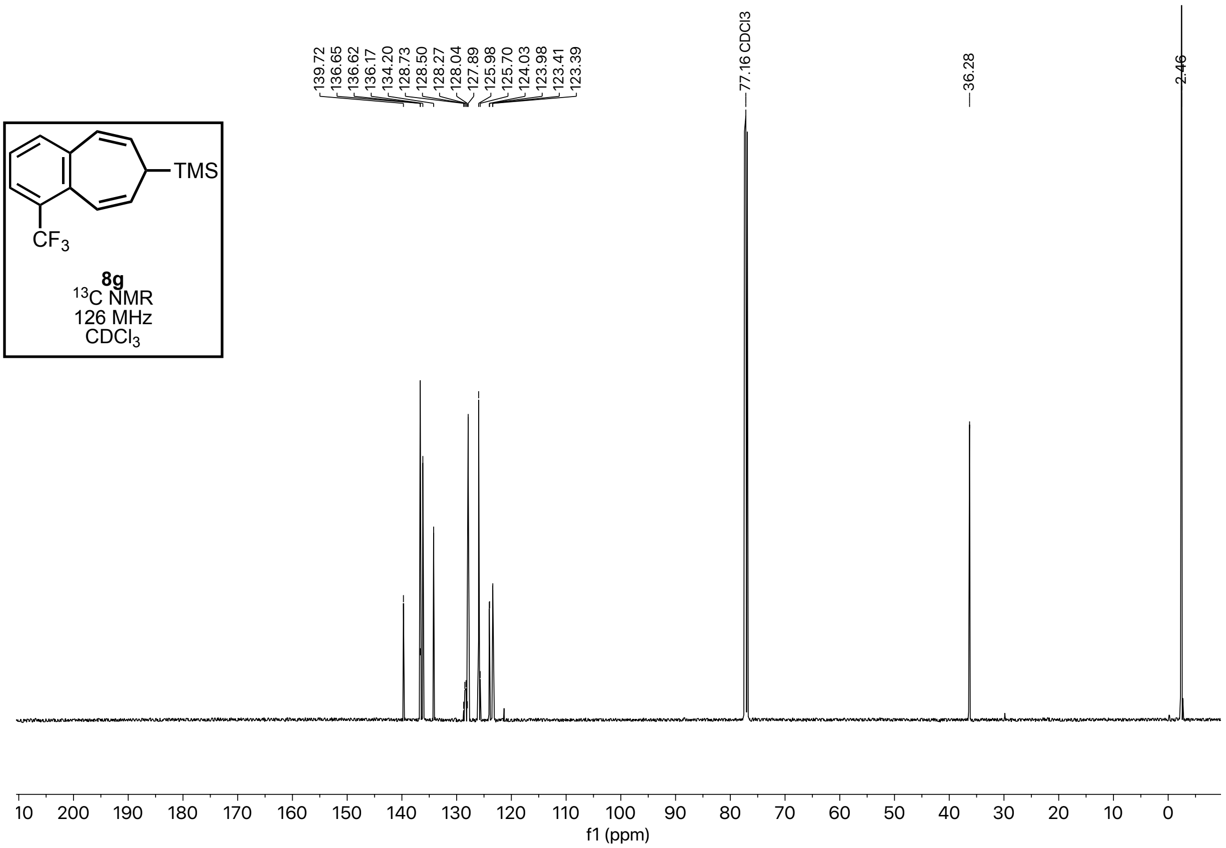
139.72
136.65
136.62
136.17
134.20
128.73
128.50
128.27
128.04
127.89
125.98
125.70
124.03
123.98
123.41
123.39
77.16 CDCl3
36.28
2.46
TMS
CF3
8g
13C NMR
126 MHz
CDCl3
f1 (ppm)

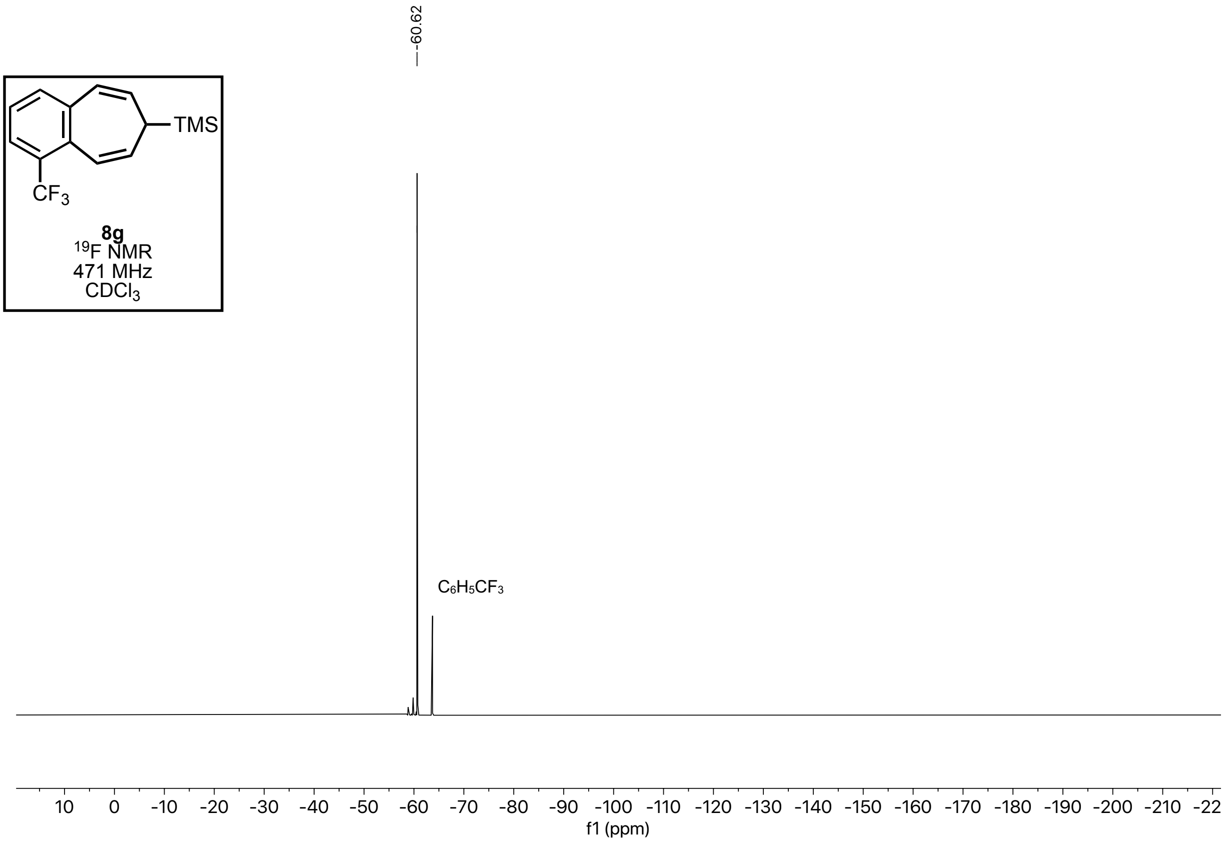
-60.62
TMS
CF3
8g
19F NMR
471 MHz
CDCl3
C6H5CF3
f1 (ppm)

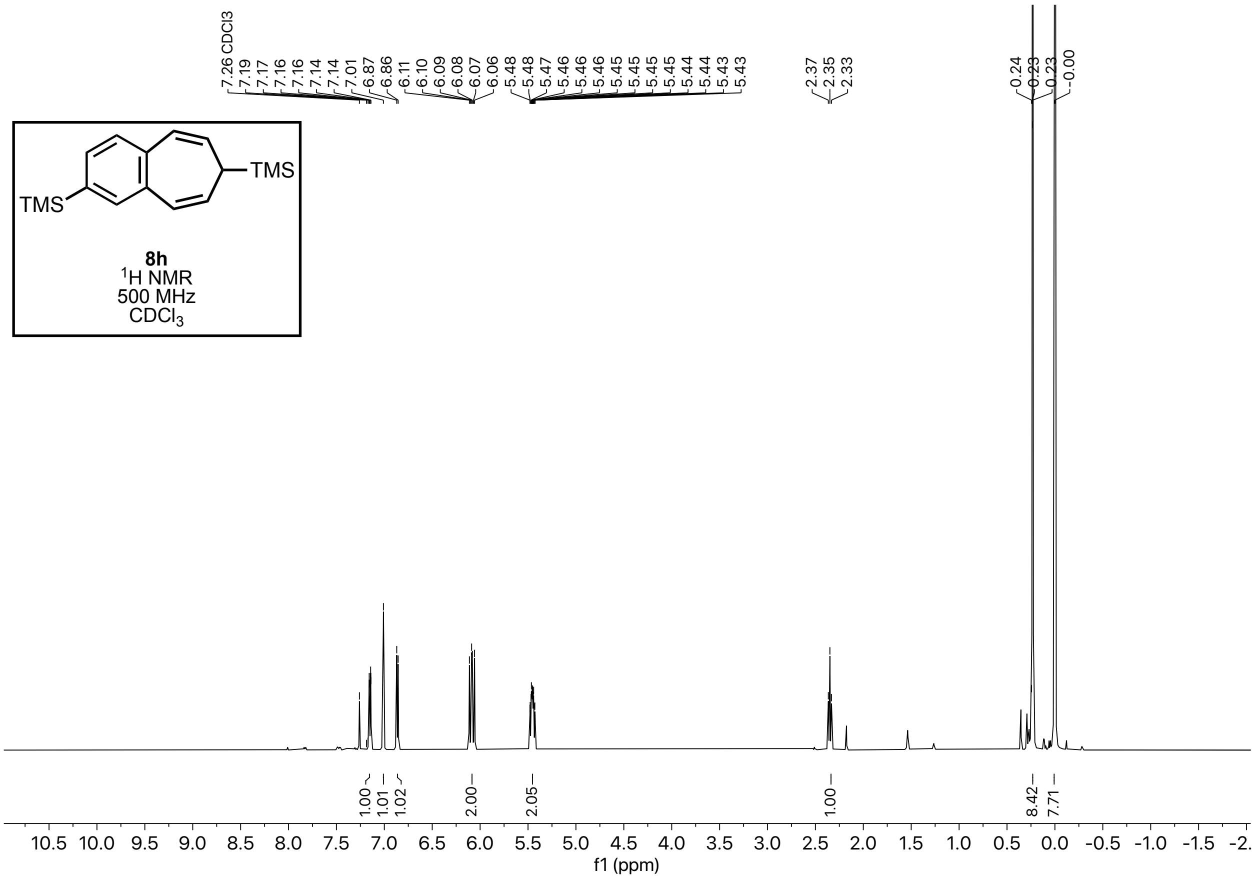
TMS
TMS
8h
$^{1}$H NMR
500 MHz
$CDCl_3$
7.26 CDCl3
7.19
7.17
7.16
7.16
7.14
7.14
7.01
6.87
6.86
6.11
6.10
6.09
6.08
6.07
6.06
5.48
5.48
5.47
5.46
5.46
5.46
5.45
5.45
5.45
5.45
5.44
5.44
5.43
5.43
2.37
2.35
2.33
0.24
0.23
0.23
-0.00
1.00
1.01
1.02
2.00
2.05
1.00
8.42
7.71
f1 (ppm)

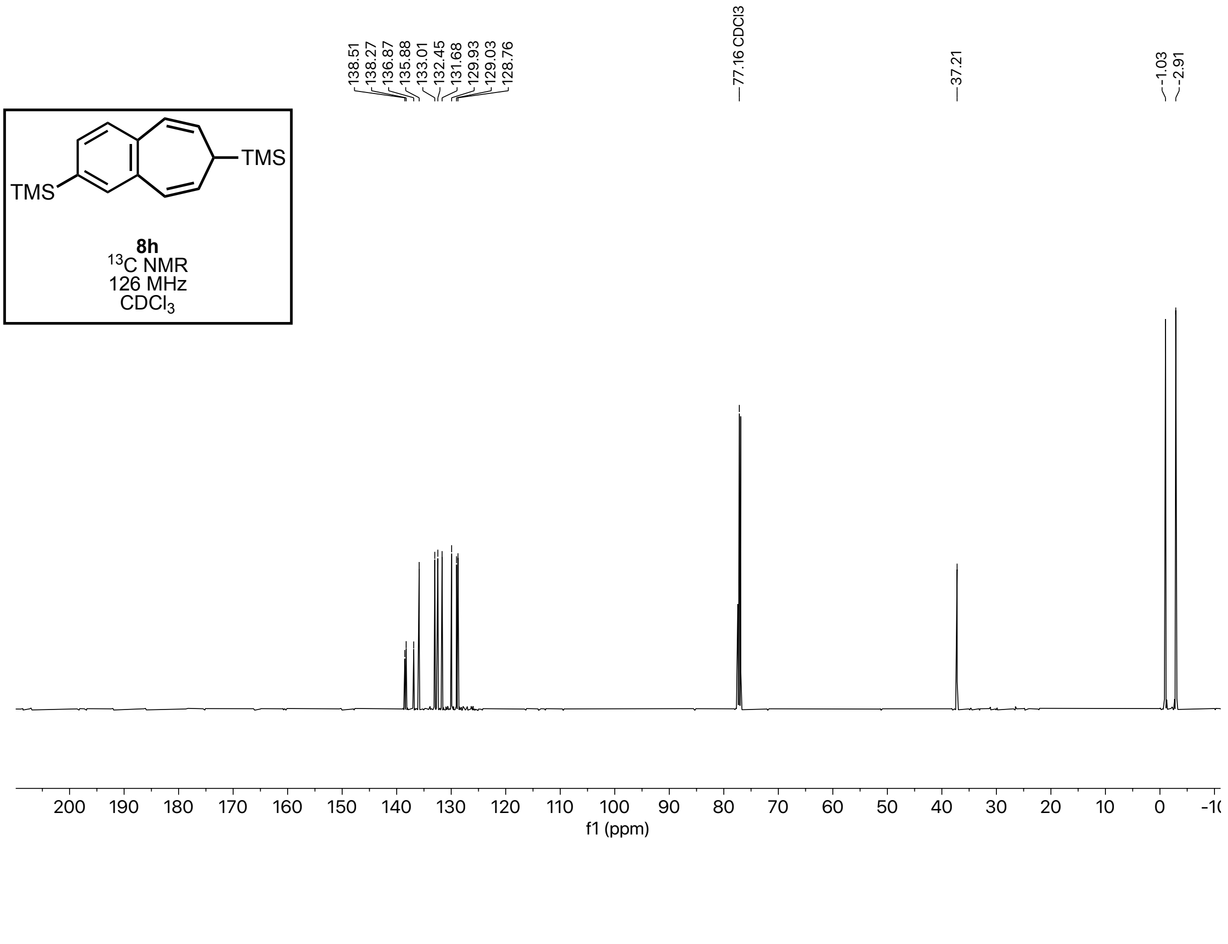
TMS
TMS
8h
$^{13}$C NMR
126 MHz
$CDCl_3$
138.51
138.27
136.87
135.88
133.01
132.45
131.68
129.93
129.03
128.76
77.16 CDCl3
37.21
-1.03
-2.91
f1 (ppm)

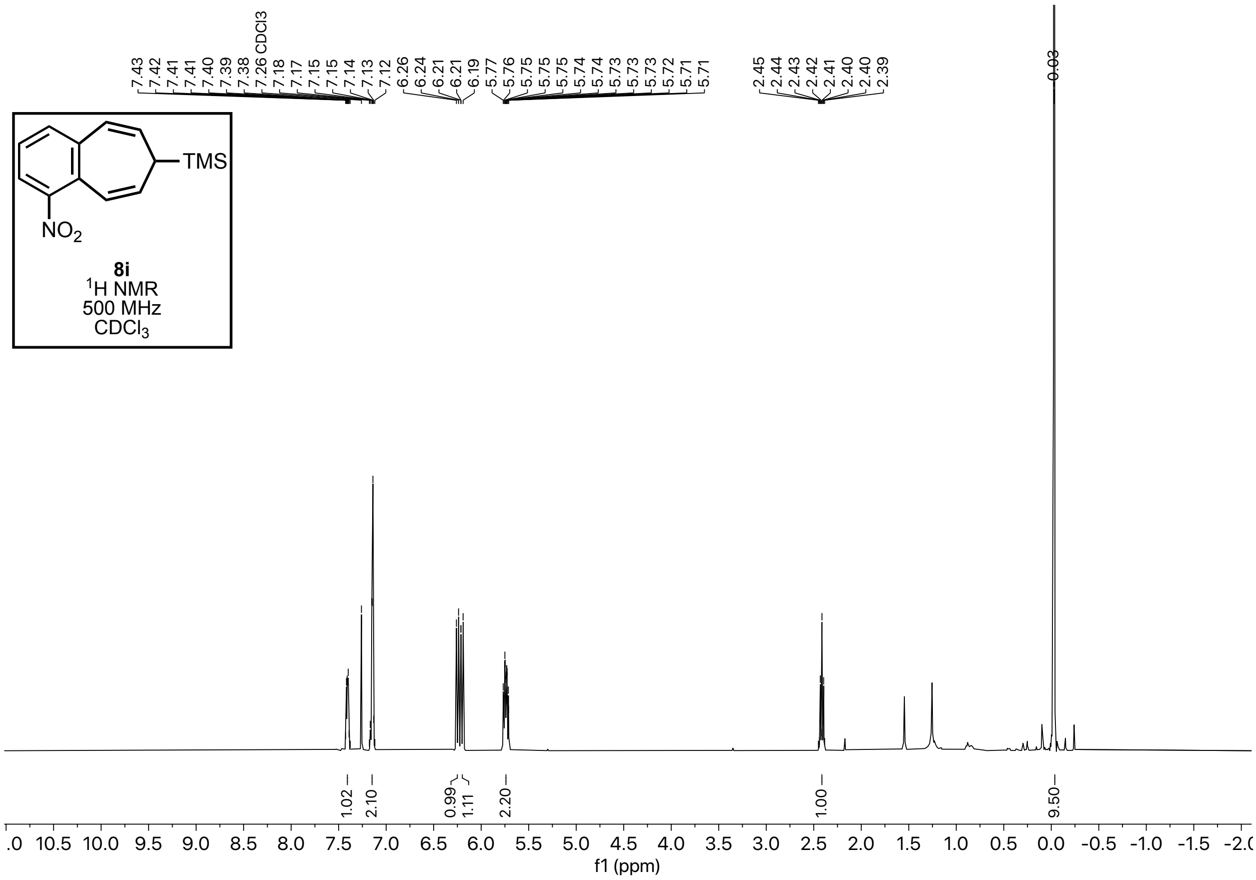
8i
$^{1}$H NMR
500 MHz
$CDCl_3$
TMS
$NO_2$
f1 (ppm)

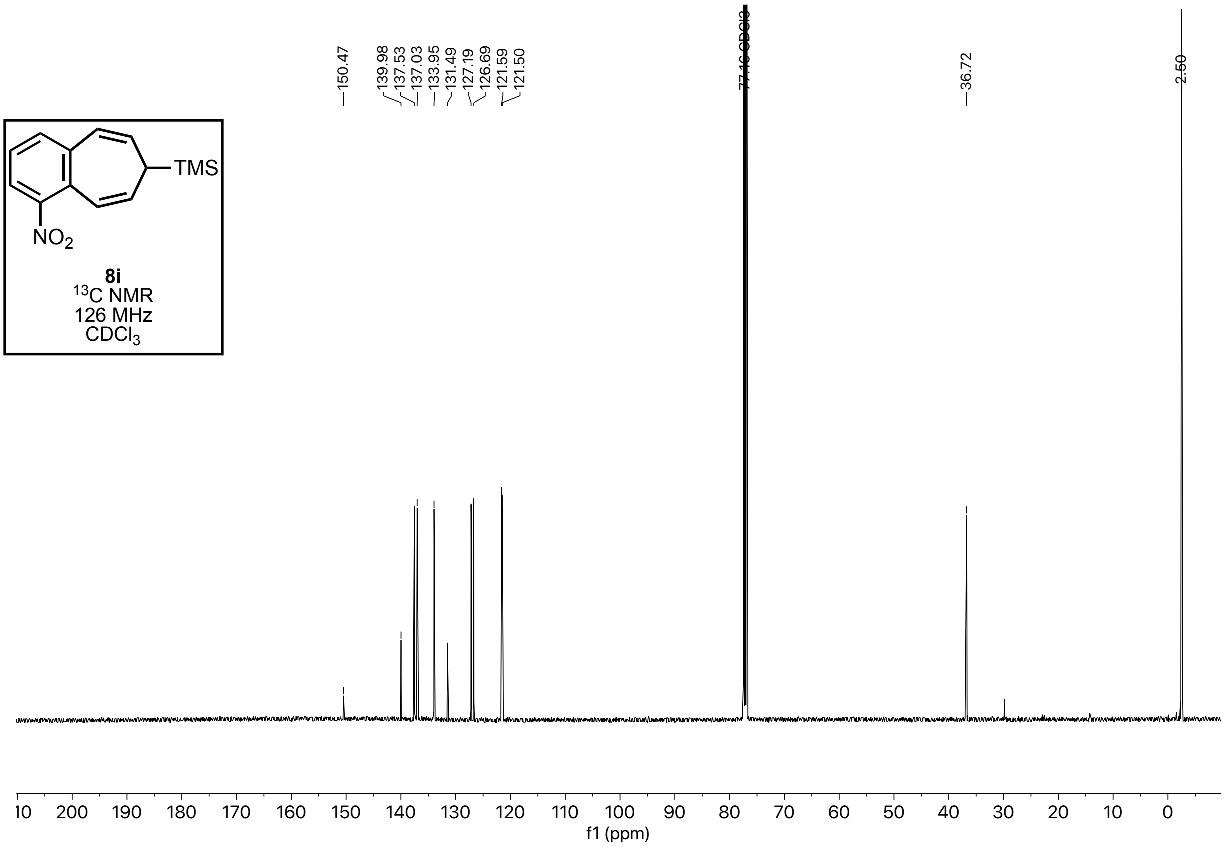
8i
$^{13}$C NMR
126 MHz
$CDCl_3$
TMS
$NO_2$
f1 (ppm)

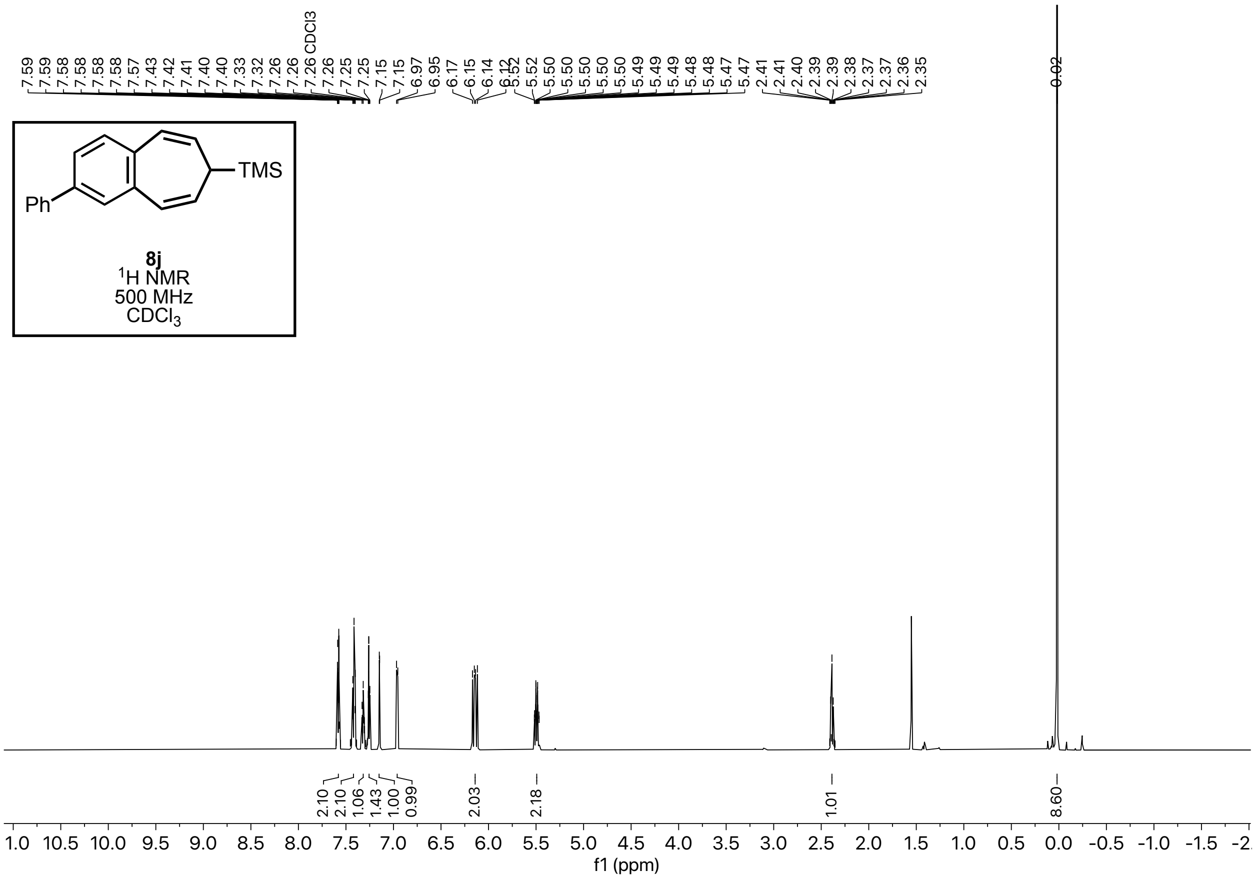
7.59
7.59
7.58
7.58
7.58
7.58
7.57
7.43
7.42
7.41
7.40
7.40
7.33
7.32
7.26
7.26 CDCl3
7.26
7.26
7.25
7.25
7.15
7.15
6.97
6.95
6.17
6.15
6.14
6.12
5.52
5.52
5.50
5.50
5.50
5.50
5.50
5.49
5.49
5.49
5.48
5.47
5.47
2.41
2.41
2.40
2.39
2.39
2.38
2.37
2.37
2.36
2.35
0.02
TMS
Ph
8j
$^{1}$H NMR
500 MHz
$CDCl_3$
2.10
2.10
1.06
1.43
1.00
0.99
2.03
2.18
1.01
8.60
f1 (ppm)

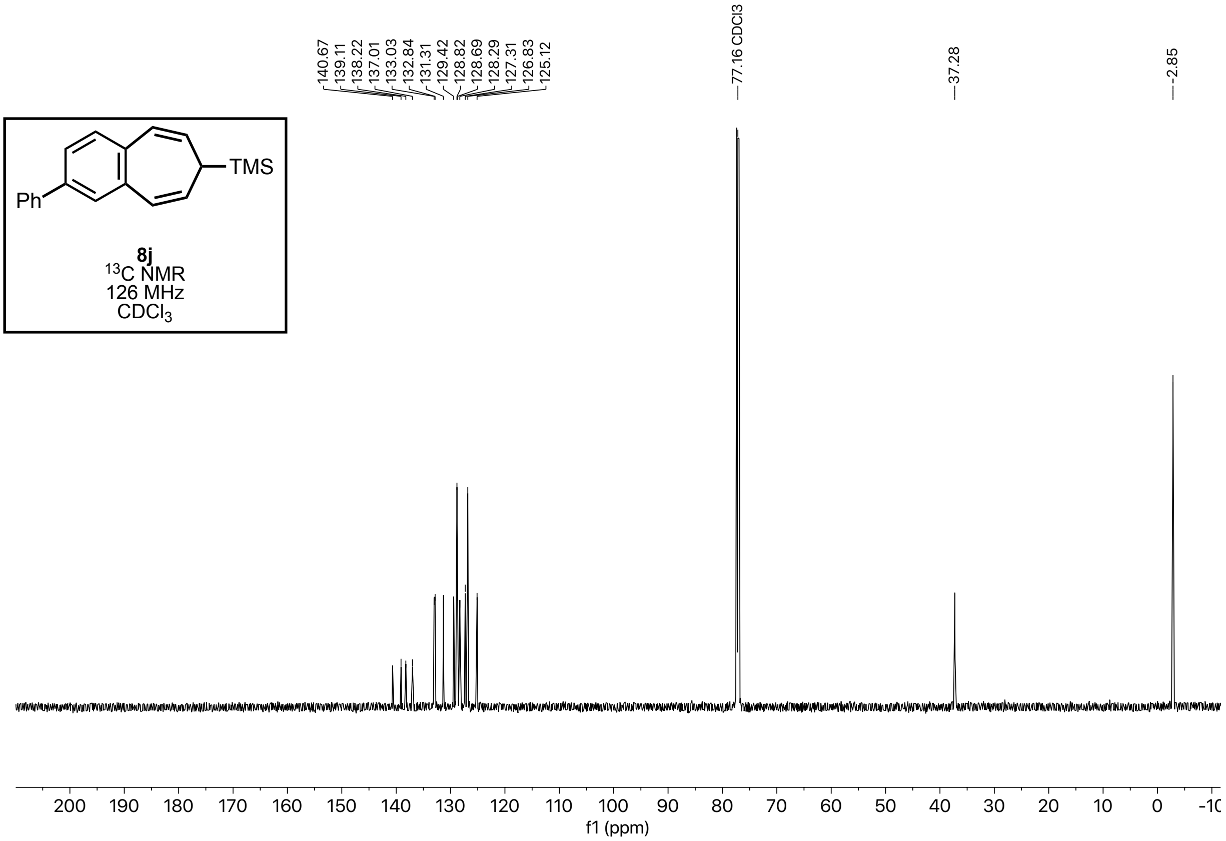
140.67
139.11
138.22
137.01
133.03
132.84
131.31
129.42
128.82
128.69
128.29
127.31
126.83
125.12
77.16 CDCl3
37.28
-2.85
TMS
Ph
8j
$^{13}$C NMR
126 MHz
$CDCl_3$
f1 (ppm)

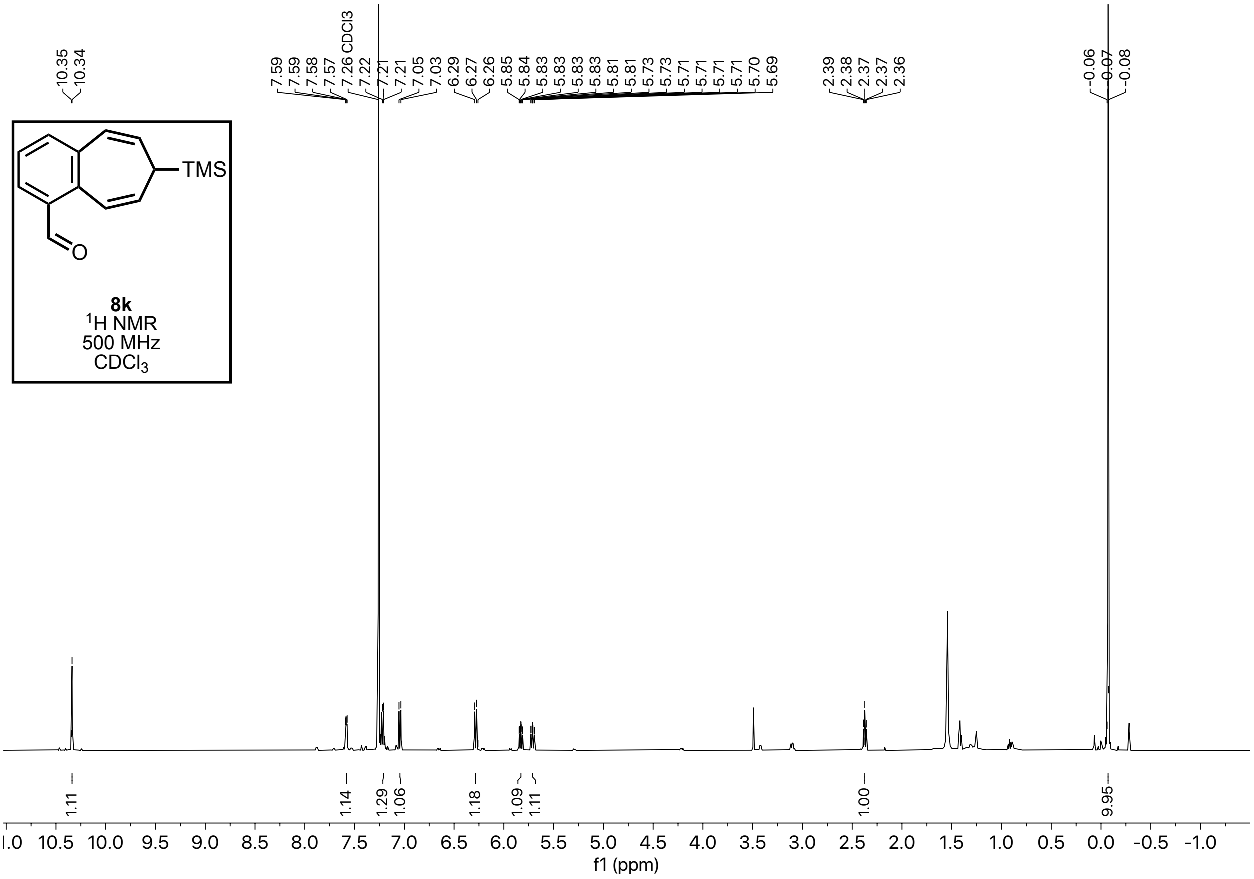
8k
$^1$H NMR
500 MHz
$CDCl_3$
TMS
O
f1 (ppm)

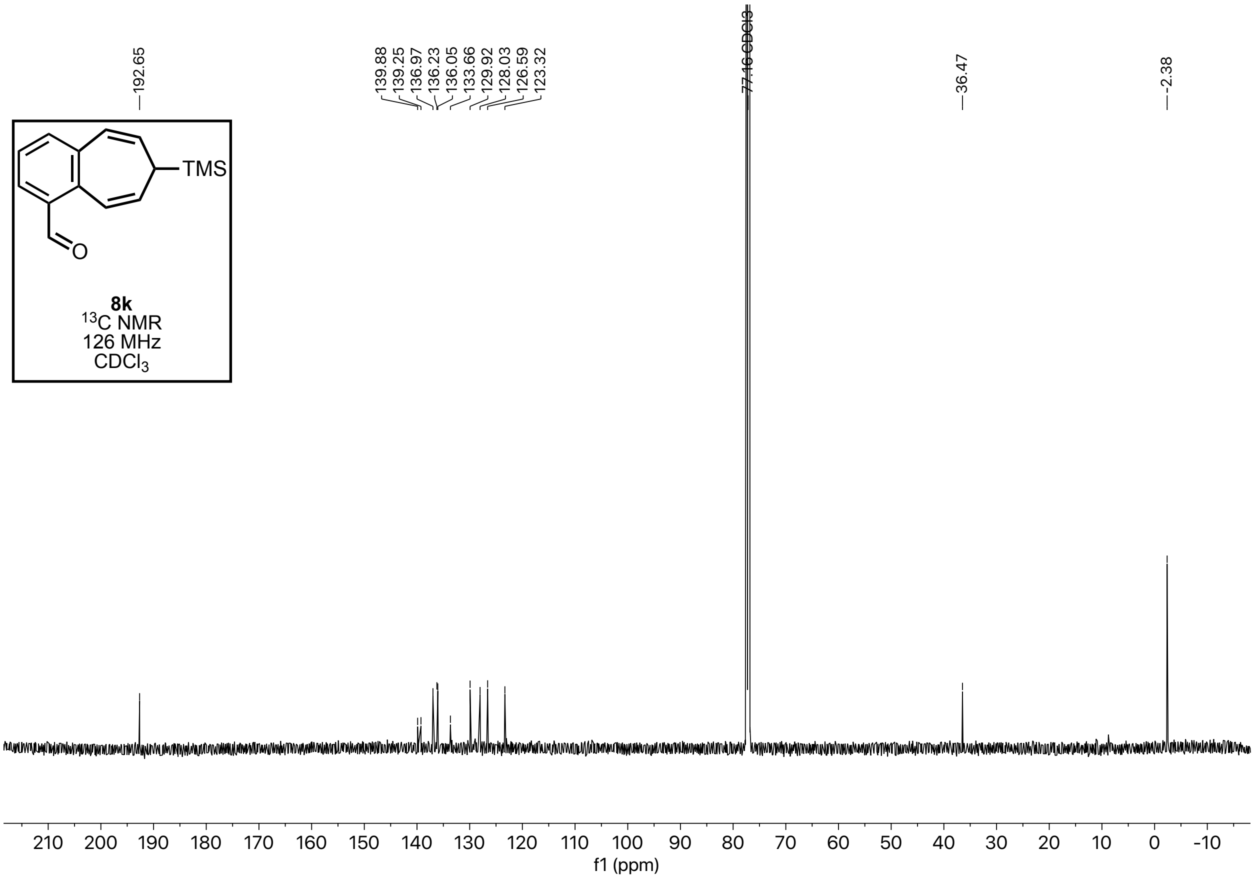
8k
$^{13}$C NMR
126 MHz
$CDCl_3$
TMS
O
f1 (ppm)

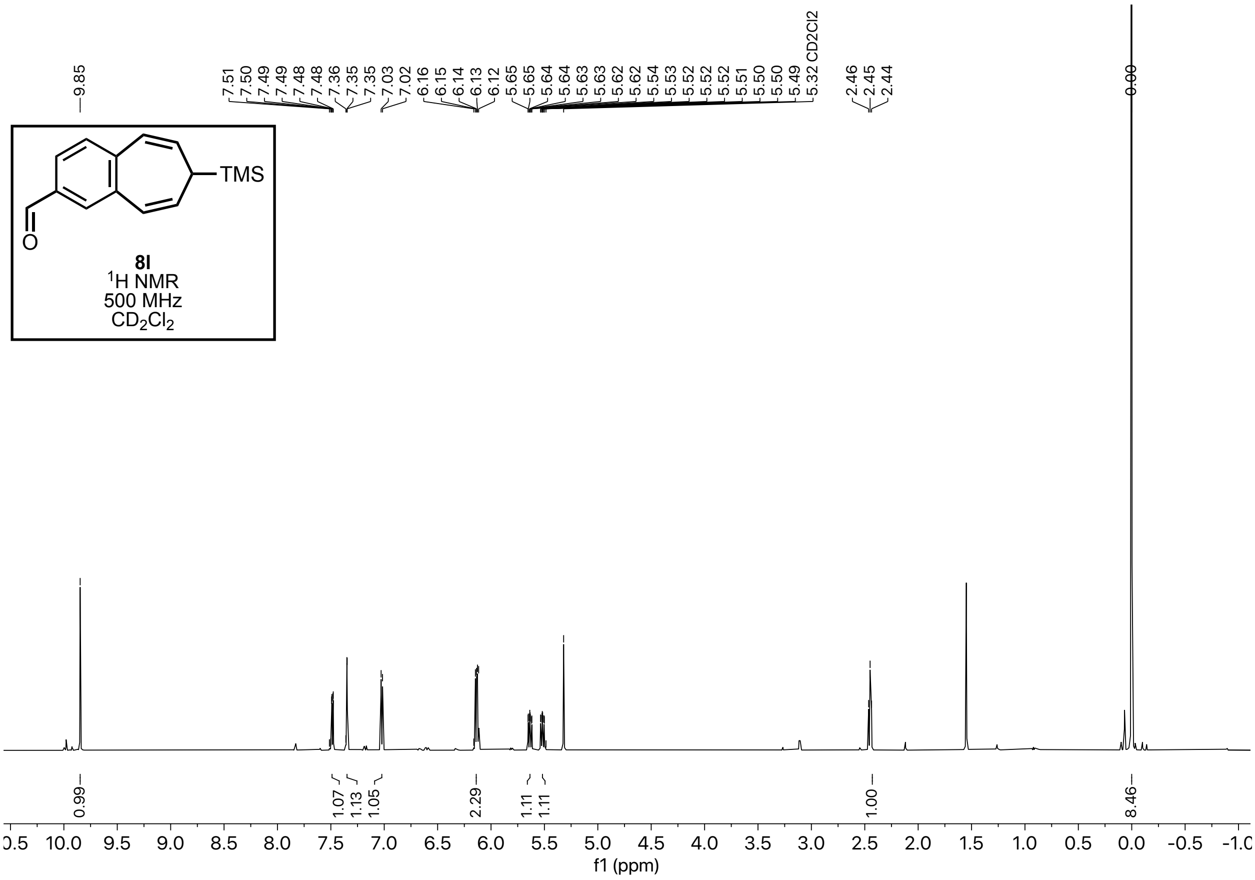
9.85
7.51
7.50
7.49
7.49
7.48
7.48
7.36
7.35
7.35
7.03
7.02
6.16
6.15
6.14
6.13
6.12
5.65
5.65
5.64
5.64
5.63
5.63
5.62
5.62
5.54
5.53
5.52
5.52
5.52
5.51
5.50
5.49
5.32 CD2Cl2
2.46
2.45
2.44
0.00
TMS
O
8l
$^1$H NMR
500 MHz
$CD_2Cl_2$
0.99
1.07
1.13
1.05
2.29
1.11
1.11
1.00
8.46
f1 (ppm)

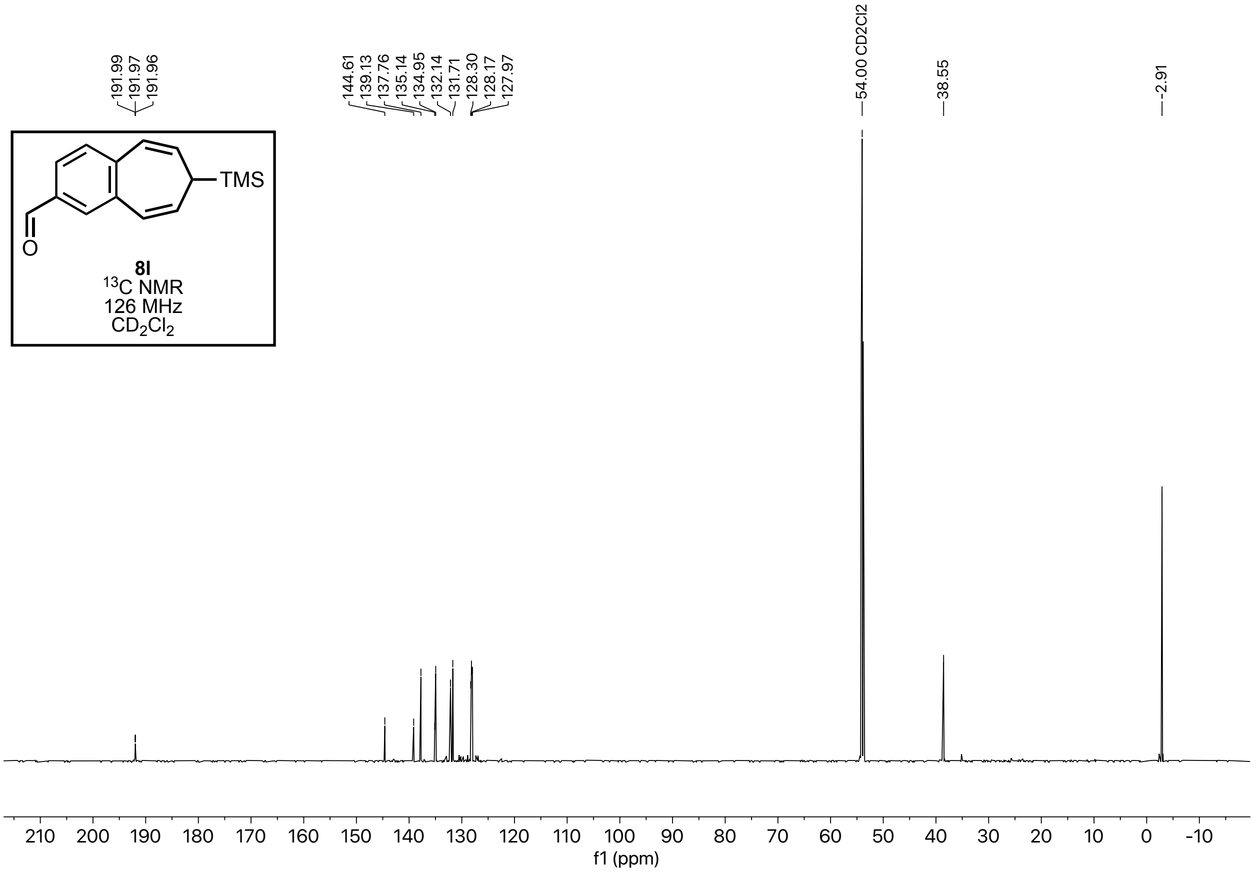
191.99
191.97
191.96
144.61
139.13
137.76
135.14
134.95
132.14
131.71
128.30
128.17
127.97
54.00 CD2Cl2
38.55
-2.91
TMS
O
8l
$^{13}$C NMR
126 MHz
$CD_2Cl_2$
f1 (ppm)

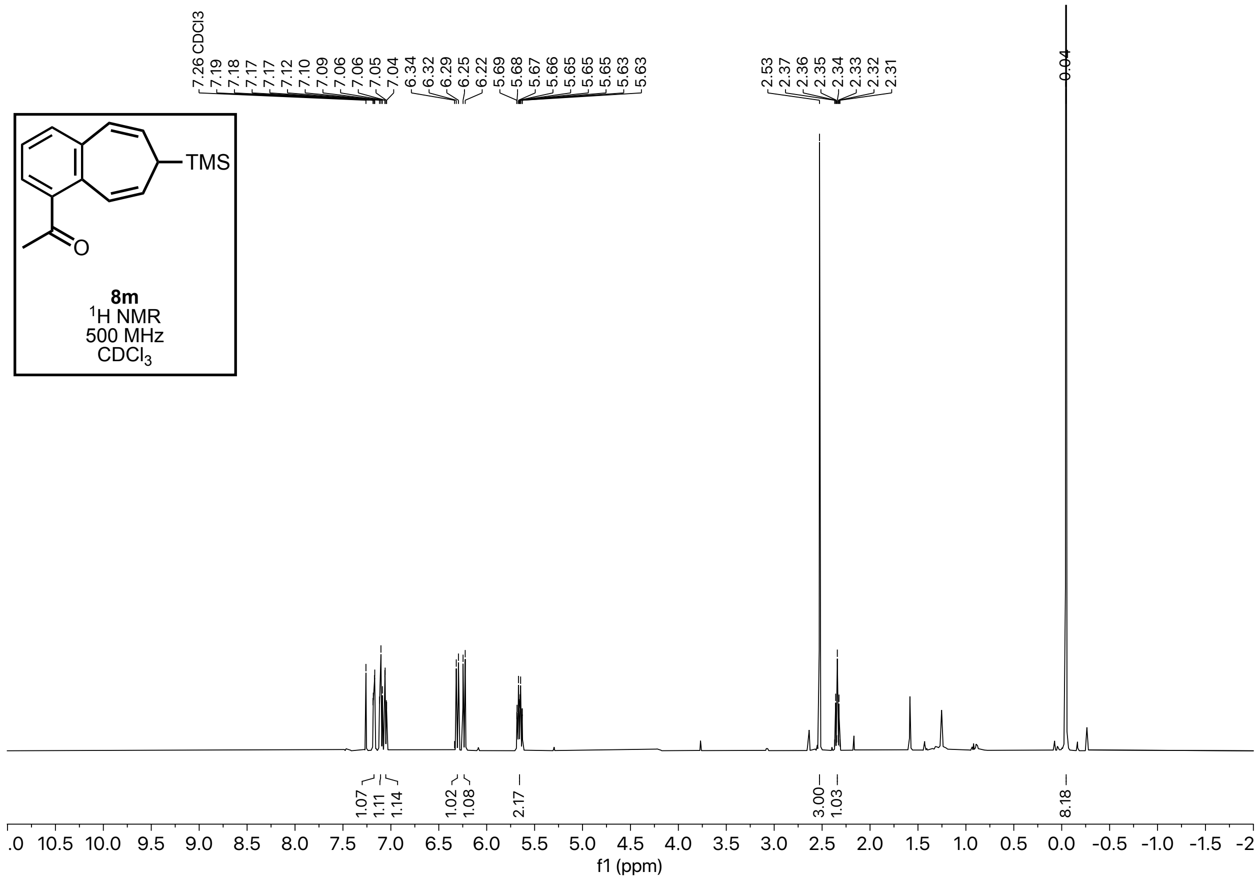
7.26 CDCl3
7.19
7.18
7.17
7.17
7.12
7.10
7.09
7.06
7.06
7.05
7.04
6.34
6.32
6.29
6.25
6.22
5.69
5.68
5.67
5.66
5.65
5.65
5.65
5.63
5.63
2.53
2.37
2.36
2.35
2.34
2.33
2.32
2.31
0.04
TMS
O
8m
1H NMR
500 MHz
CDCl3
1.07
1.11
1.14
1.02
1.08
2.17
3.00
1.03
8.18
f1 (ppm)

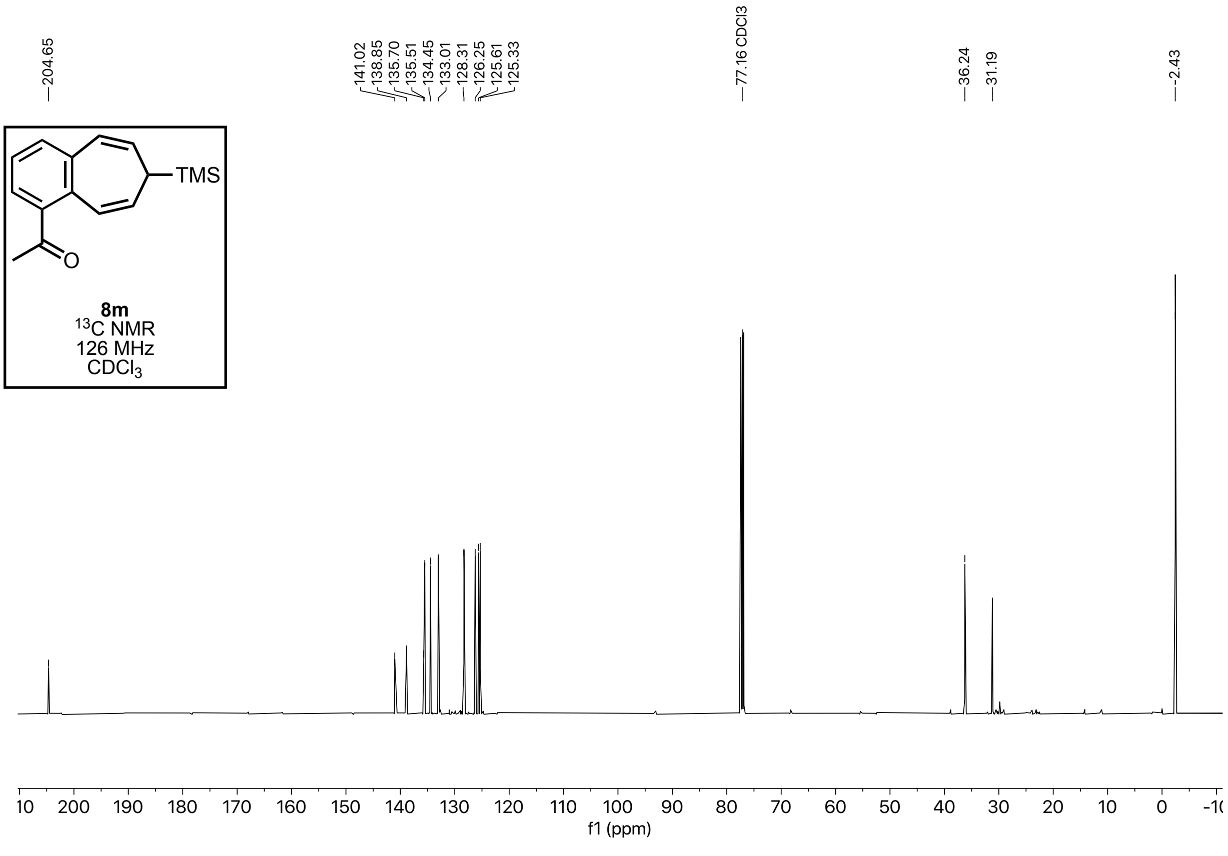
204.65
141.02
138.85
135.70
135.51
134.45
133.01
128.31
126.25
125.61
125.33
77.16 CDCl3
36.24
31.19
-2.43
TMS
O
8m
13C NMR
126 MHz
CDCl3
f1 (ppm)

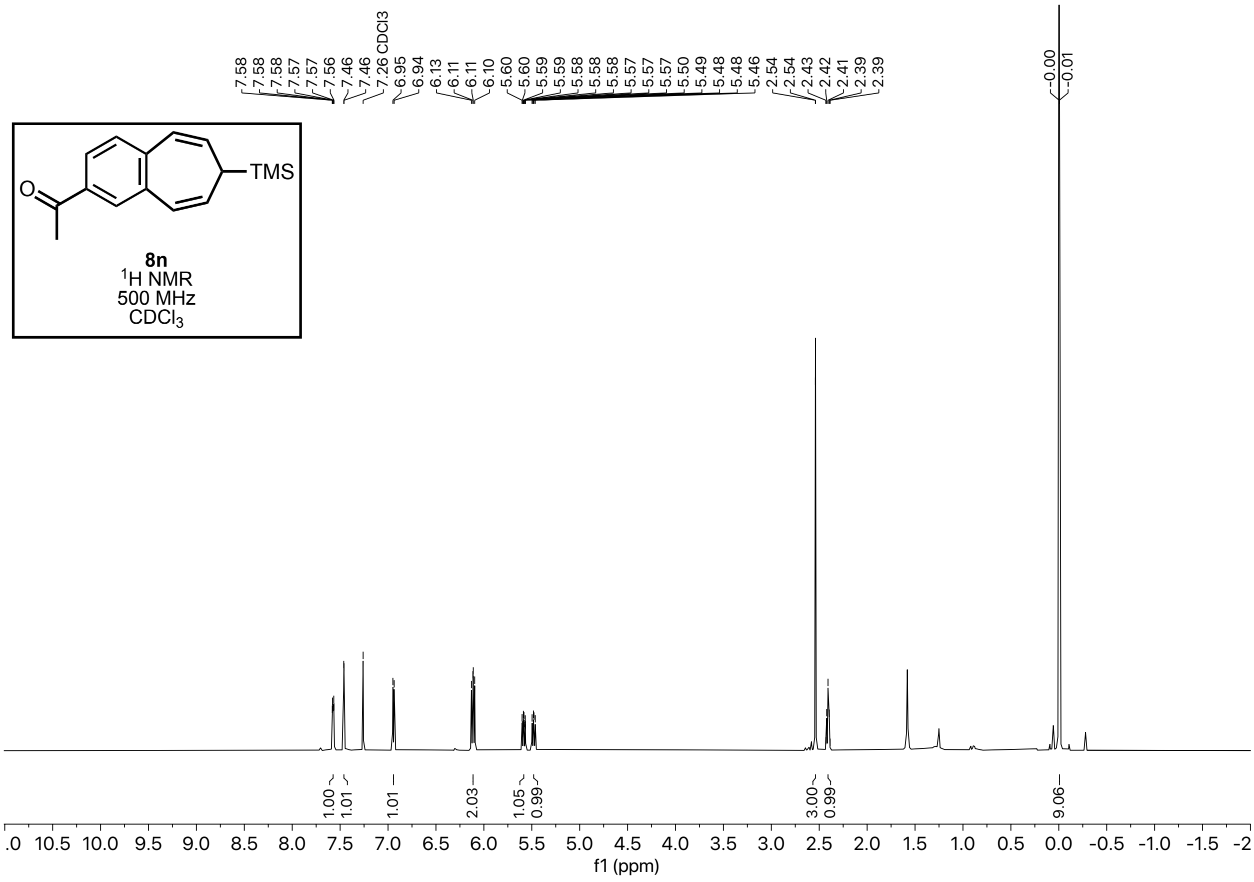
8n
$^1$H NMR
500 MHz
$CDCl_3$
TMS
7.26 CDCl3
-0.00
-0.01
1.00
1.01
1.01
2.03
1.05
0.99
3.00
0.99
9.06
f1 (ppm)

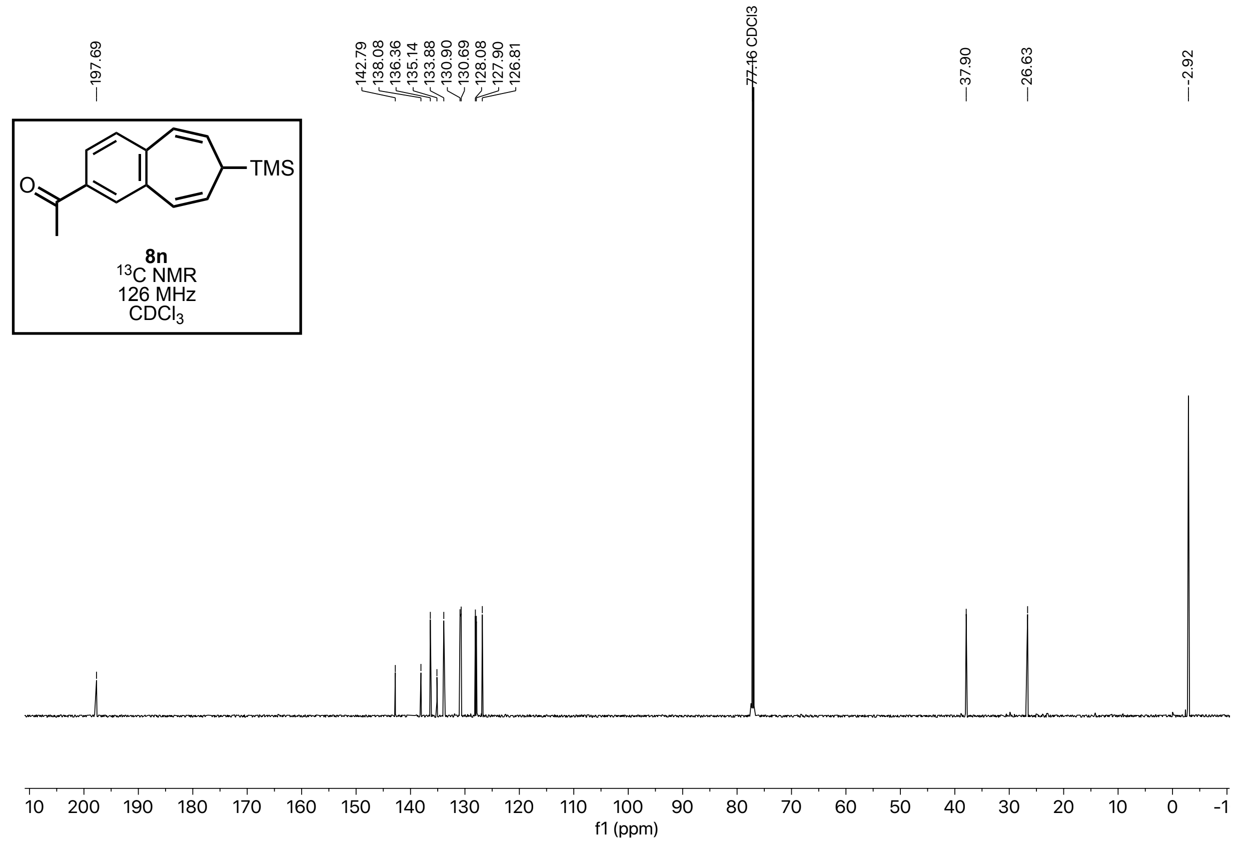
8n
$^{13}$C NMR
126 MHz
$CDCl_3$
TMS
197.69
142.79
138.08
136.36
135.14
133.88
130.90
130.69
128.08
127.90
126.81
77.16 CDCl3
37.90
26.63
-2.92
f1 (ppm)

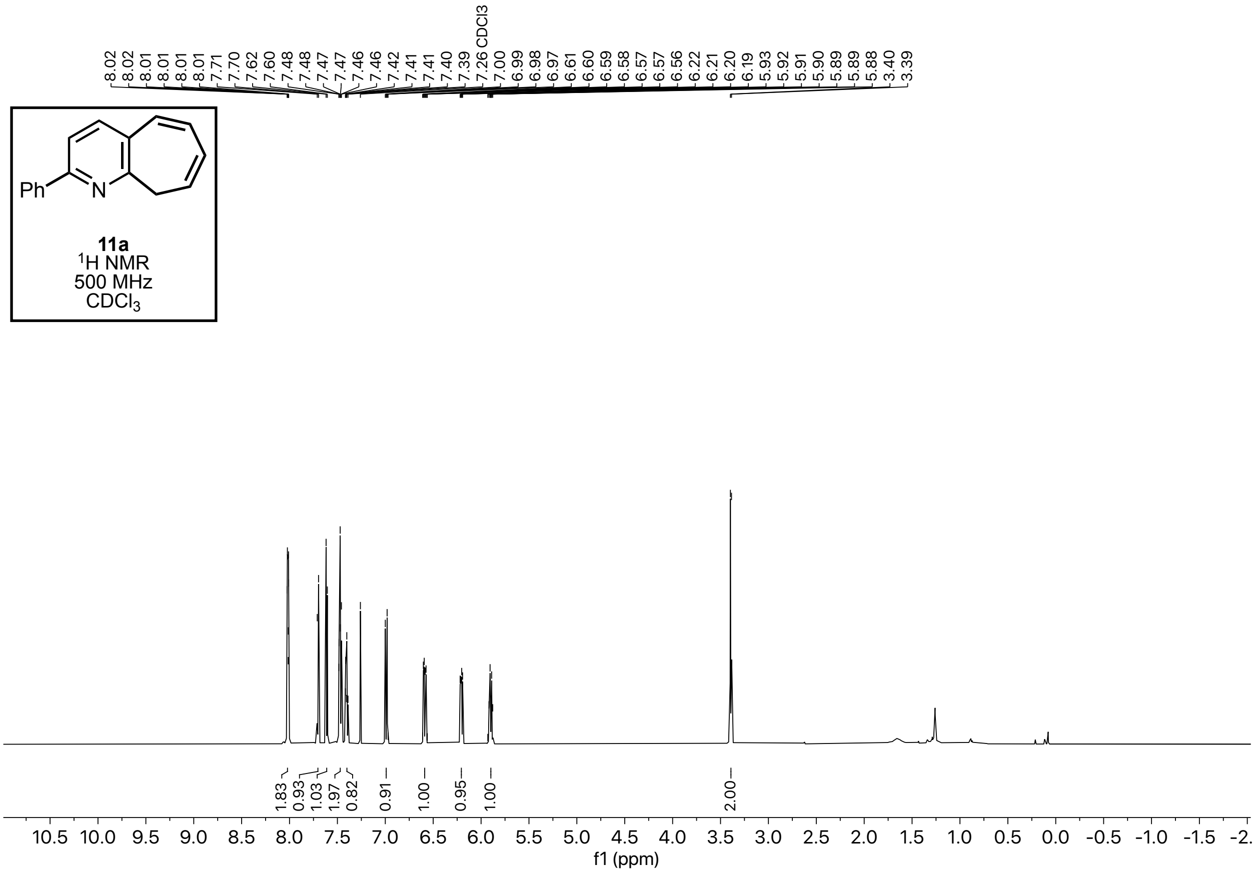
8.02
8.02
8.01
8.01
8.01
8.01
7.71
7.70
7.62
7.60
7.48
7.48
7.47
7.47
7.46
7.46
7.42
7.41
7.41
7.40
7.39
7.26 CDCl3
7.00
6.99
6.98
6.97
6.61
6.60
6.59
6.58
6.57
6.57
6.56
6.22
6.21
6.20
6.19
5.93
5.92
5.91
5.90
5.89
5.89
5.88
3.40
3.39
Ph
N
11a
1H NMR
500 MHz
CDCl3
1.83
0.93
1.03
1.97
0.82
0.91
1.00
0.95
1.00
2.00
f1 (ppm)

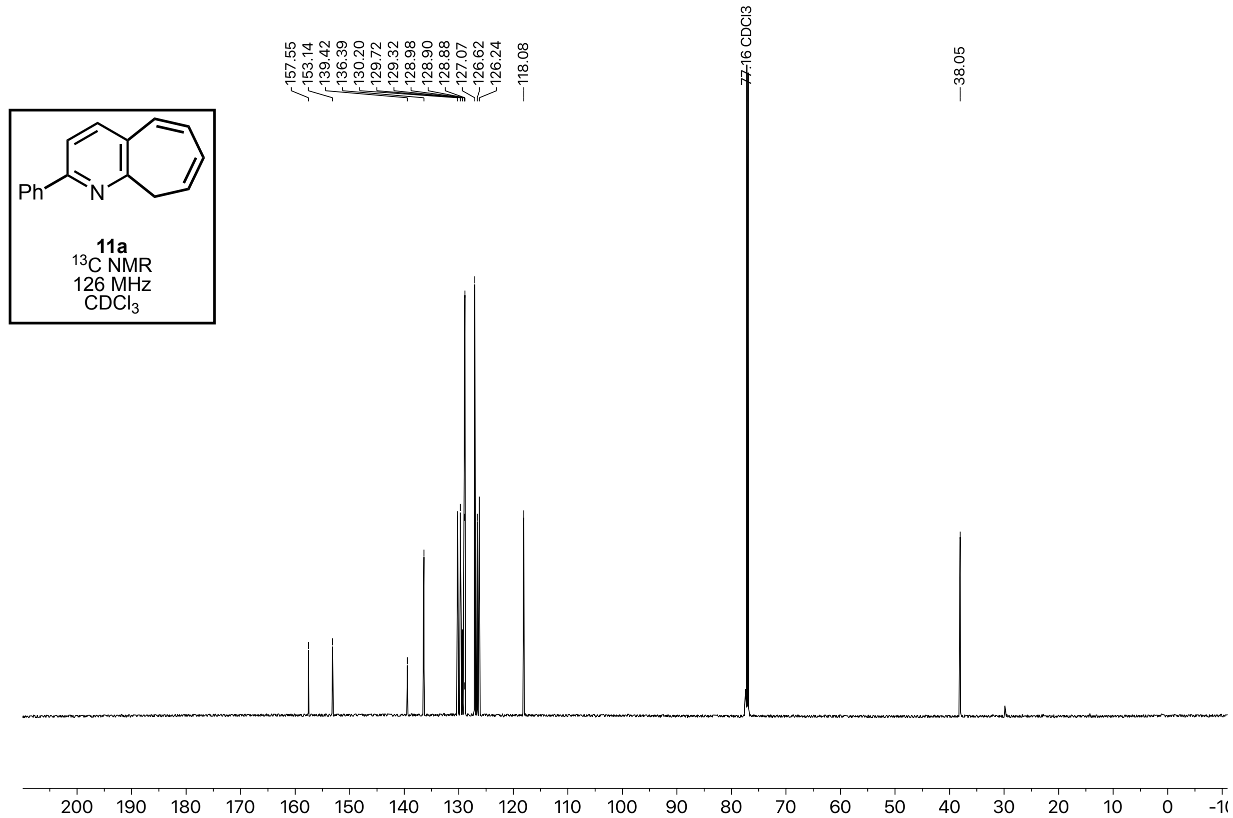
157.55
153.14
139.42
136.39
130.20
129.72
129.32
128.98
128.90
128.88
127.07
126.62
126.24
118.08
77.16 CDCl3
38.05
Ph
N
11a
13C NMR
126 MHz
CDCl3
f1 (ppm)

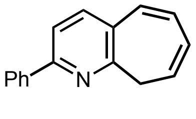

**11a**
$^1H-^1H$
COSY
500 MHz
$CDCl_3$

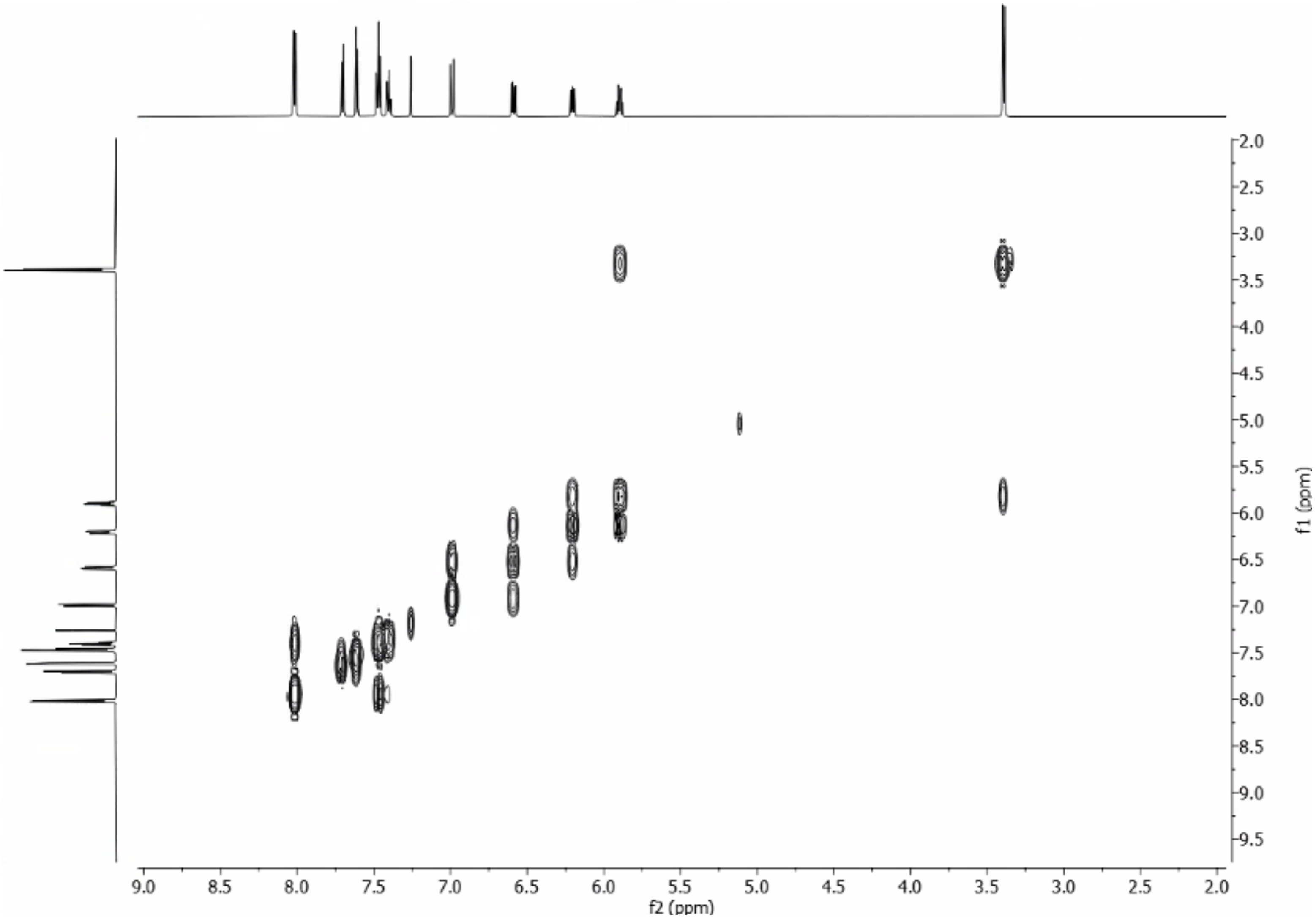

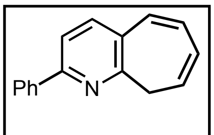


**11a**
$^{1}H-^{13}C$
HSQC
500 MHz
$CDCl_3$

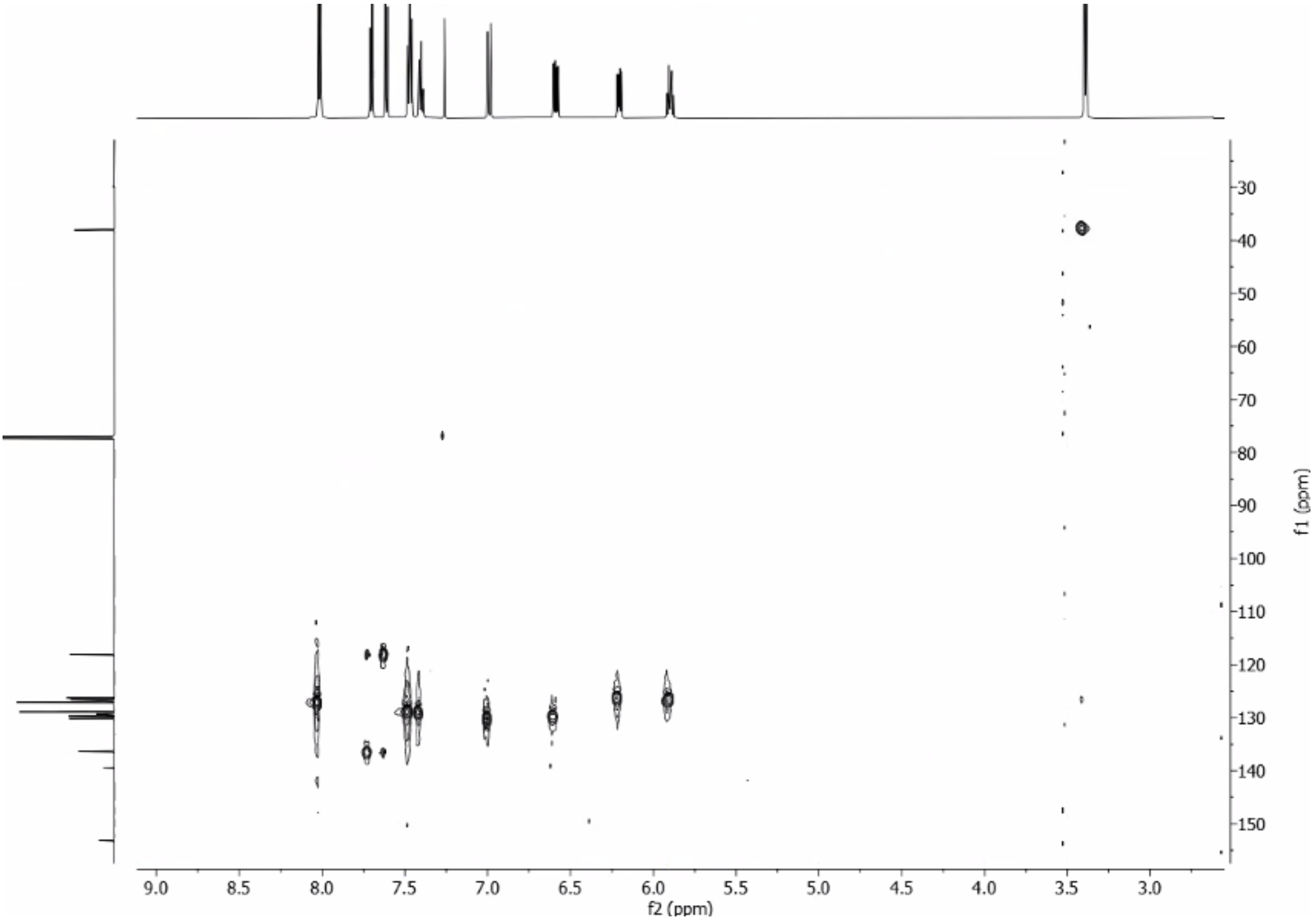

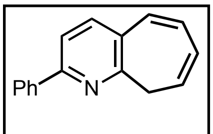


**11a**
$^{1}H-^{13}C$
HMBC
500 MHz
$CDCl_3$

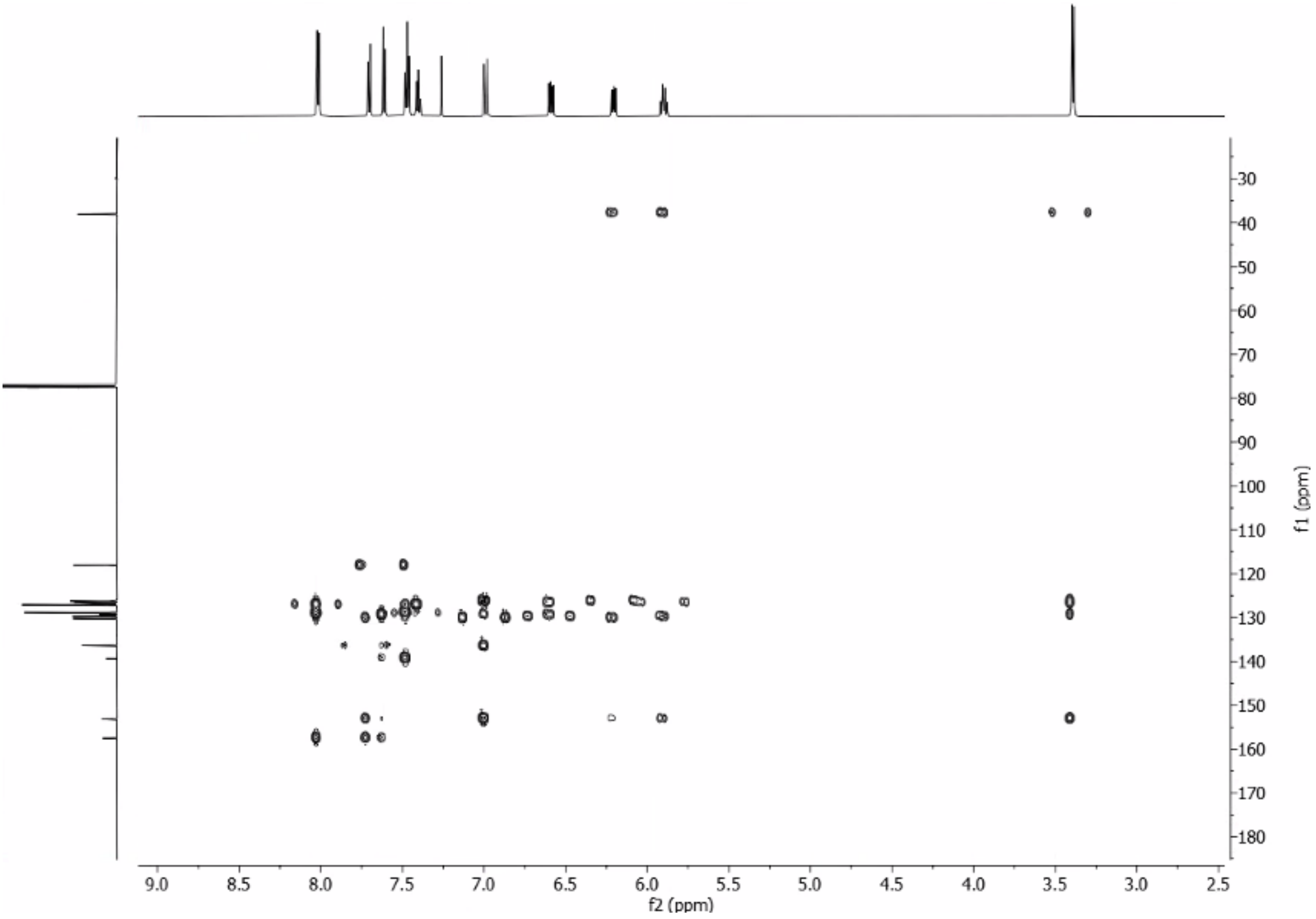

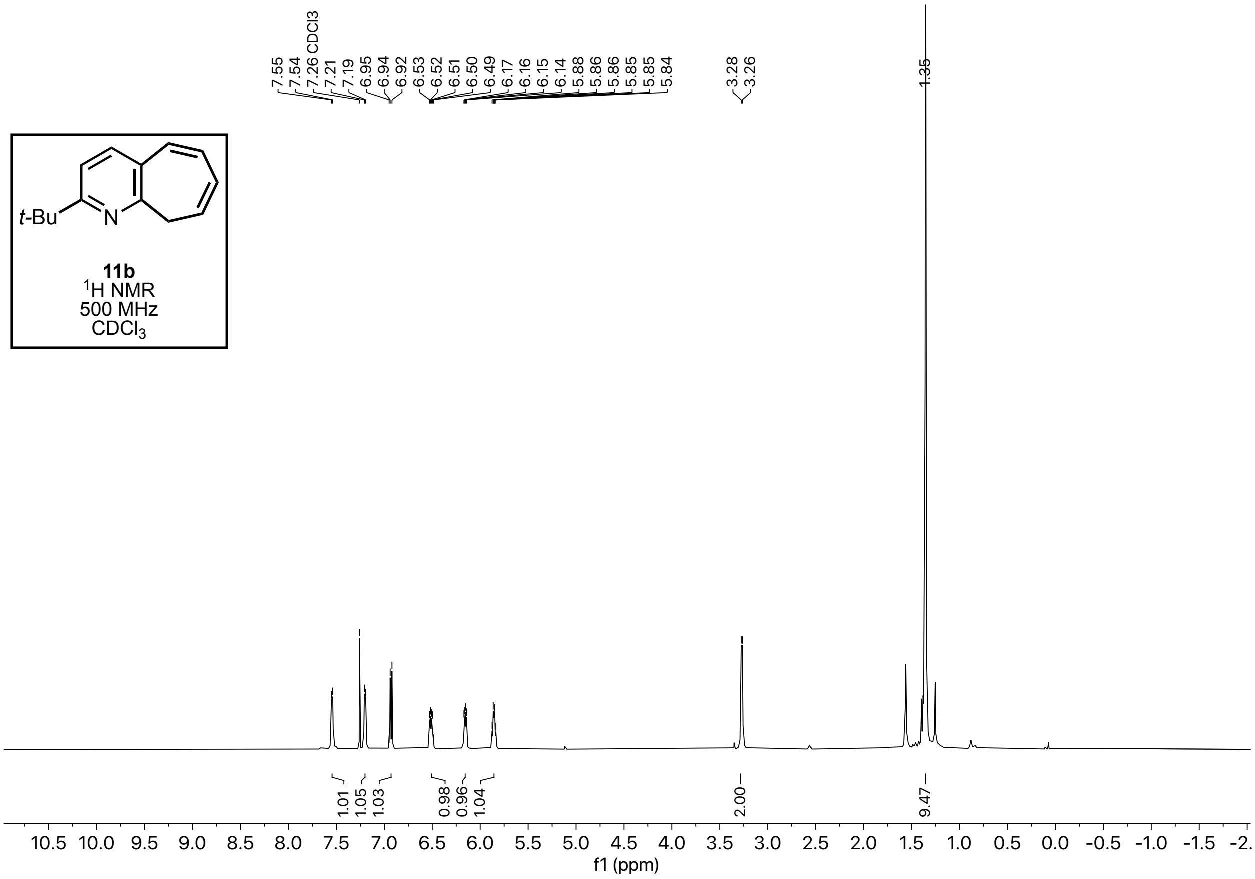
7.55
7.54
7.26 CDCl3
7.21
7.19
6.95
6.94
6.92
6.53
6.52
6.51
6.50
6.49
6.17
6.16
6.15
6.14
5.88
5.86
5.86
5.85
5.85
5.84
3.28
3.26
1.35
t-Bu
N
**11b**
$^{1}$H NMR
500 MHz
CDCl$_3$
1.01
1.05
1.03
0.98
0.96
1.04
2.00
9.47
10.5 10.0 9.5 9.0 8.5 8.0 7.5 7.0 6.5 6.0 5.5 5.0 4.5 4.0 3.5 3.0 2.5 2.0 1.5 1.0 0.5 0.0 -0.5 -1.0 -1.5 -2.
f1 (ppm)

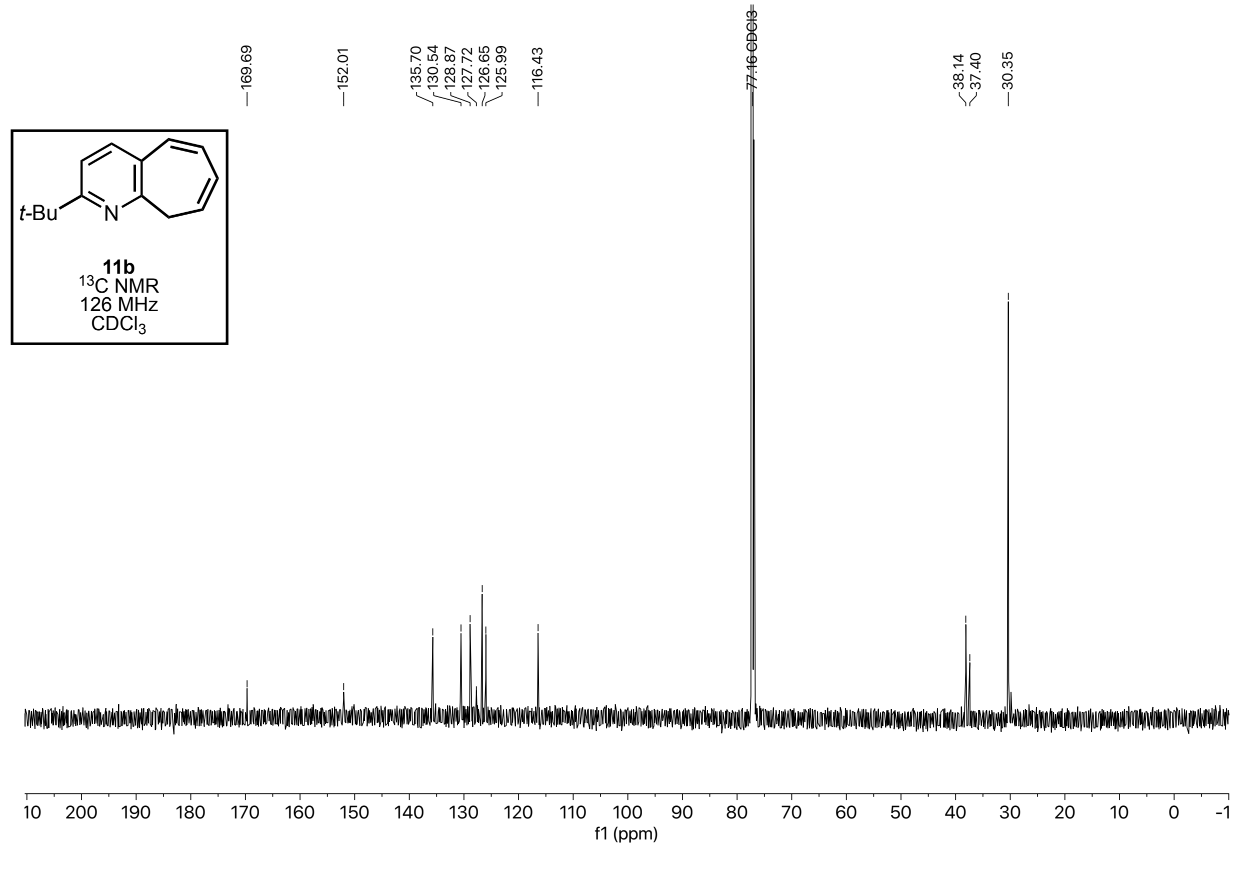
169.69
152.01
135.70
130.54
128.87
127.72
126.65
125.99
116.43
77.16 CDCl3
38.14
37.40
30.35
t-Bu
N
**11b**
$^{13}$C NMR
126 MHz
CDCl$_3$
10 200 190 180 170 160 150 140 130 120 110 100 90 80 70 60 50 40 30 20 10 0 -1
f1 (ppm)

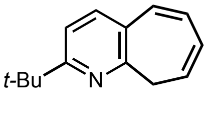


**11b**
$^1H-^1H$
COSY
500 MHz
$CDCl_3$

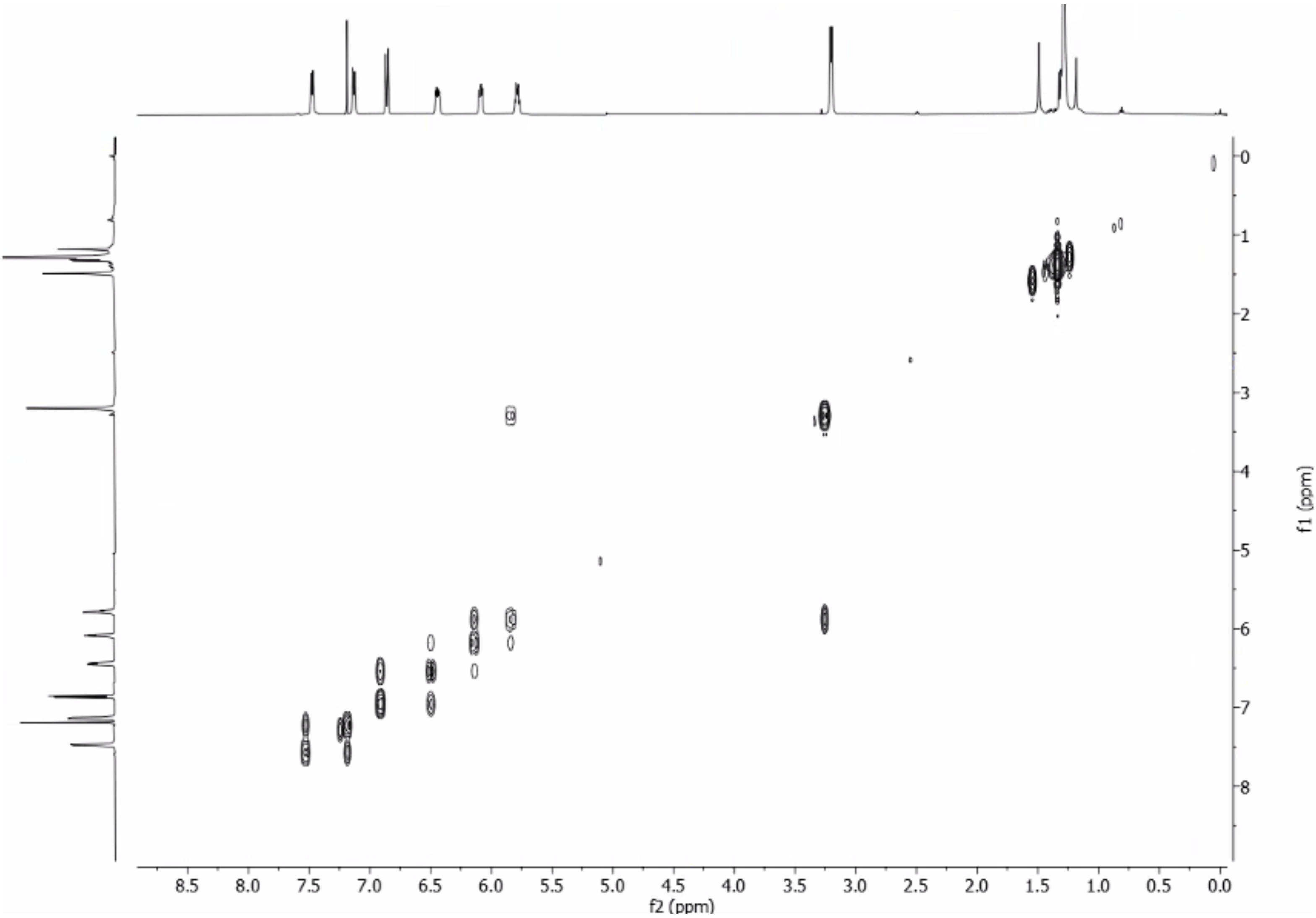

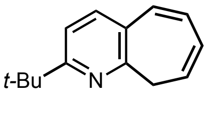


**11b**
$^{1}H-^{13}C$
HSQC
500 MHz
$CDCl_3$

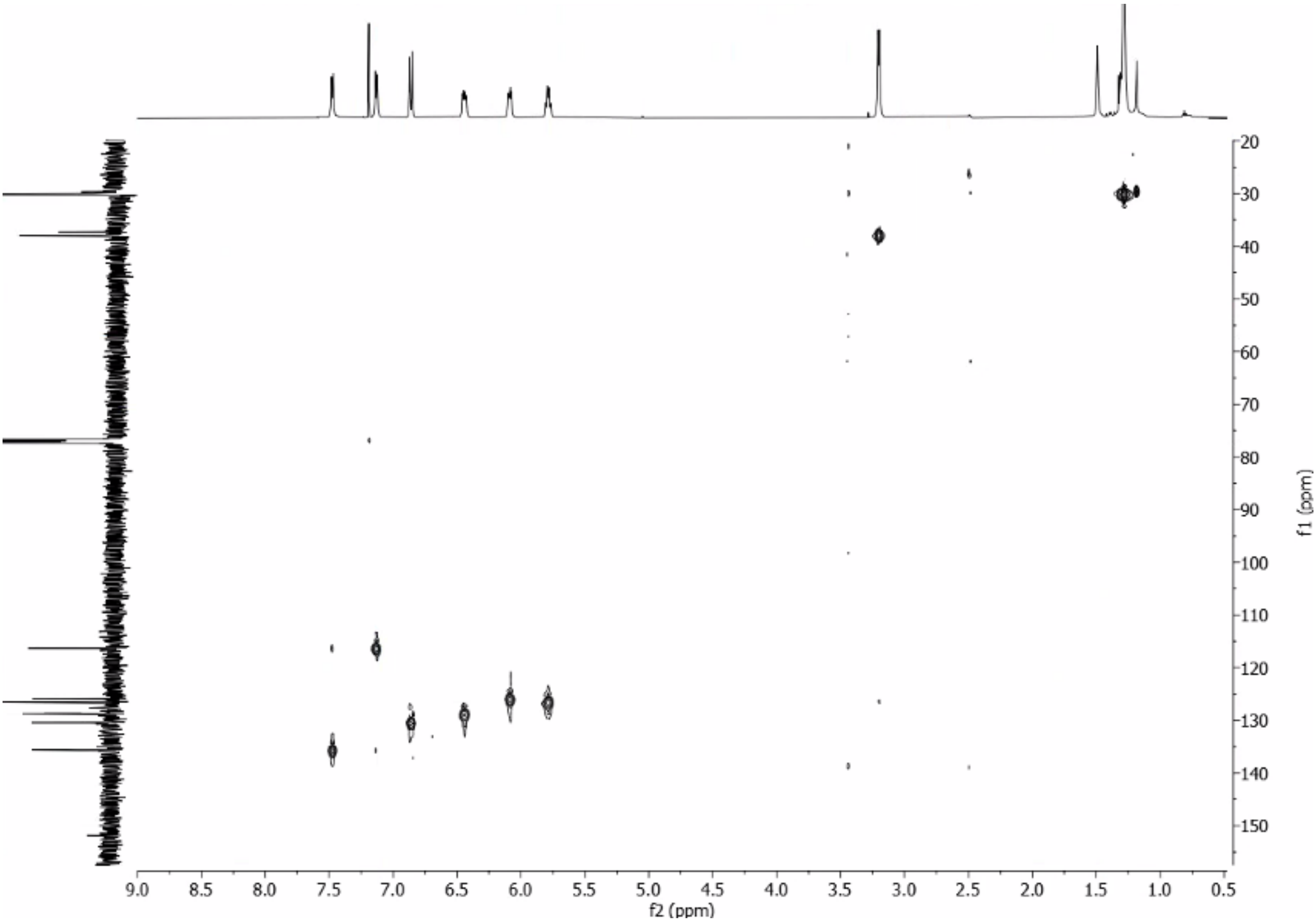

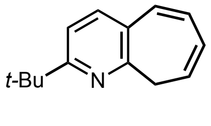


**11b**
$^1H-^{13}C$
HMBC
500 MHz
$CDCl_3$

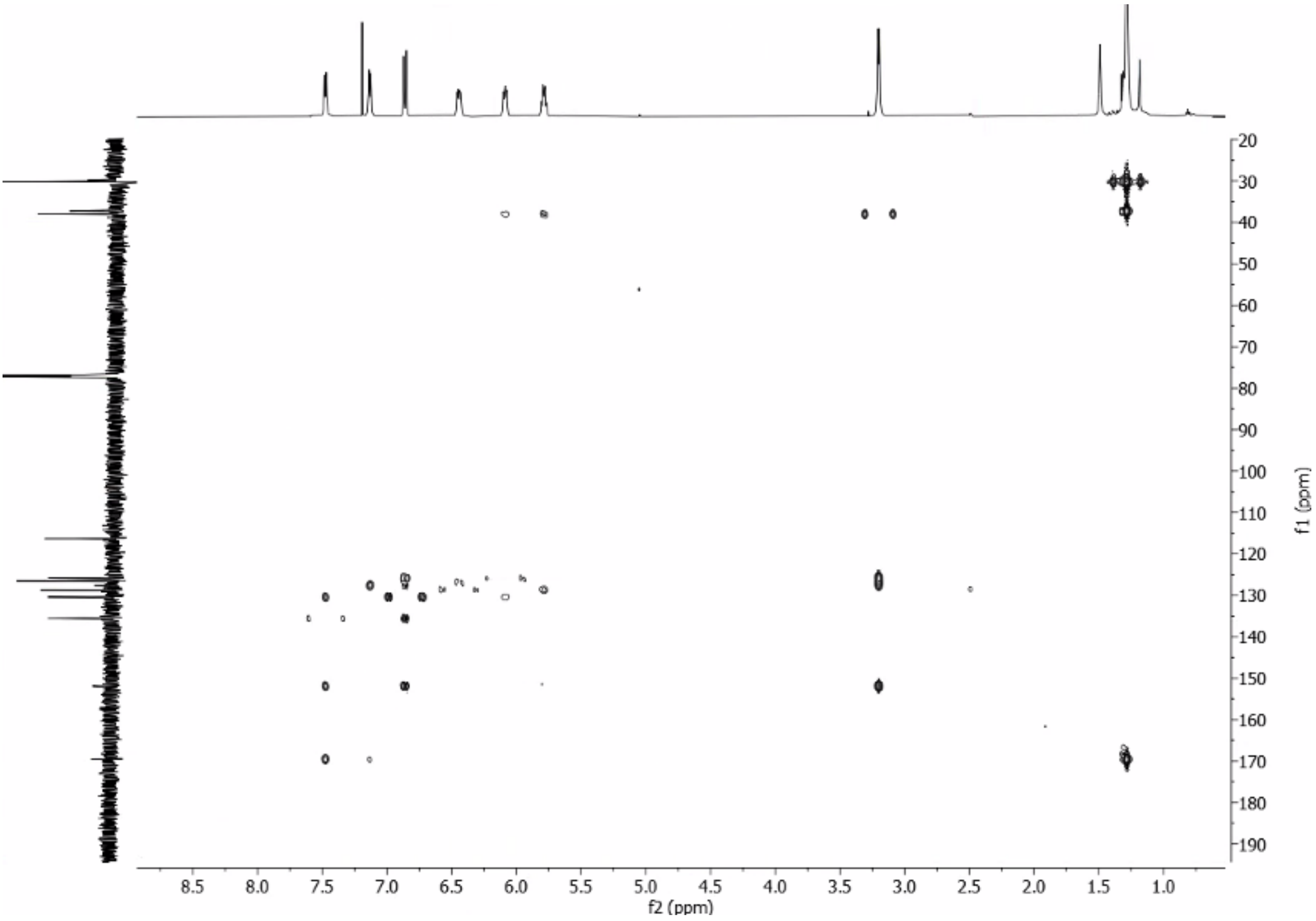

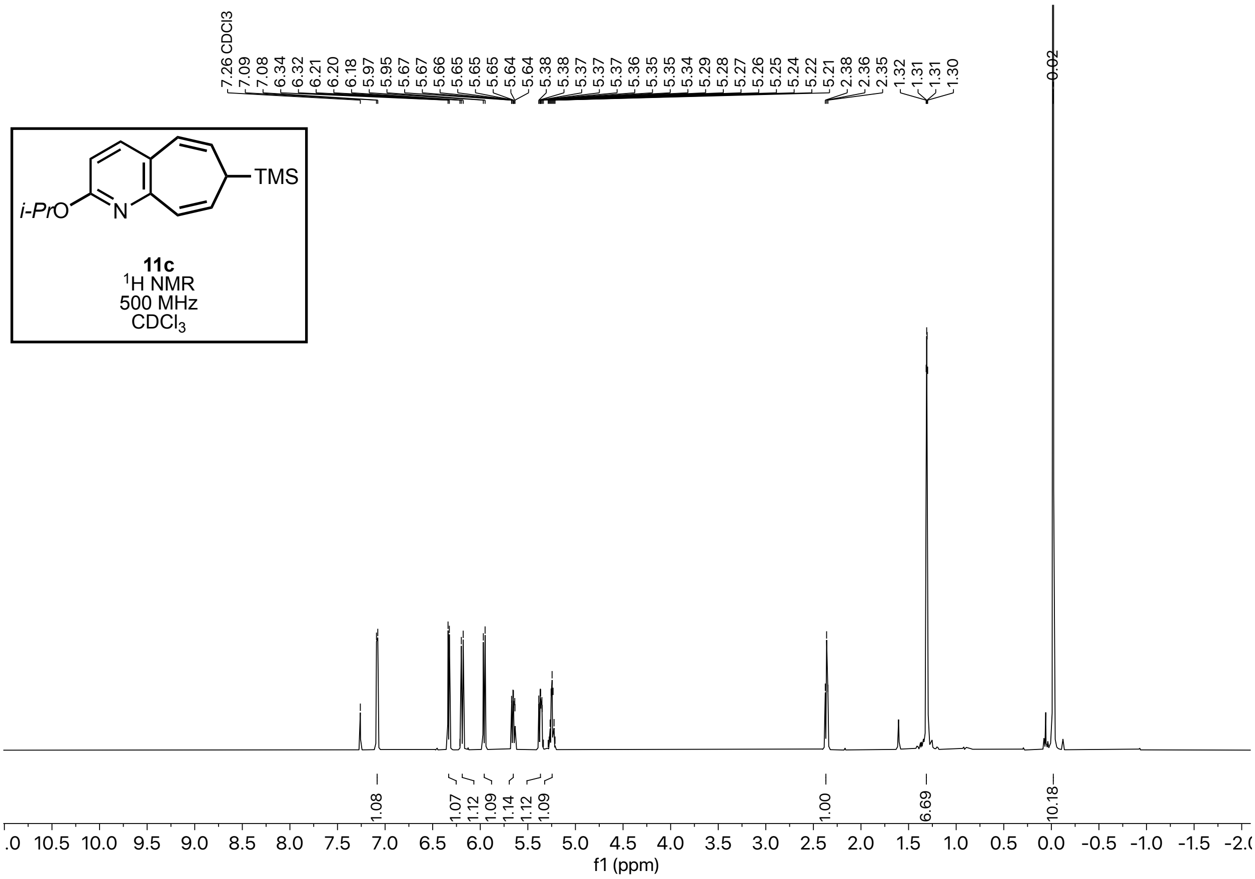
TMS
i-PrO
N
11c
1H NMR
500 MHz
CDCl3
7.26 CDCl3
7.09
7.08
6.34
6.32
6.21
6.20
6.18
5.97
5.95
5.67
5.67
5.66
5.65
5.65
5.65
5.64
5.64
5.38
5.38
5.37
5.37
5.36
5.35
5.35
5.34
5.29
5.28
5.27
5.26
5.25
5.24
5.22
5.21
2.38
2.36
2.35
1.32
1.31
1.31
1.30
0.02
1.08
1.07
1.12
1.09
1.14
1.12
1.09
1.00
6.69
10.18
f1 (ppm)

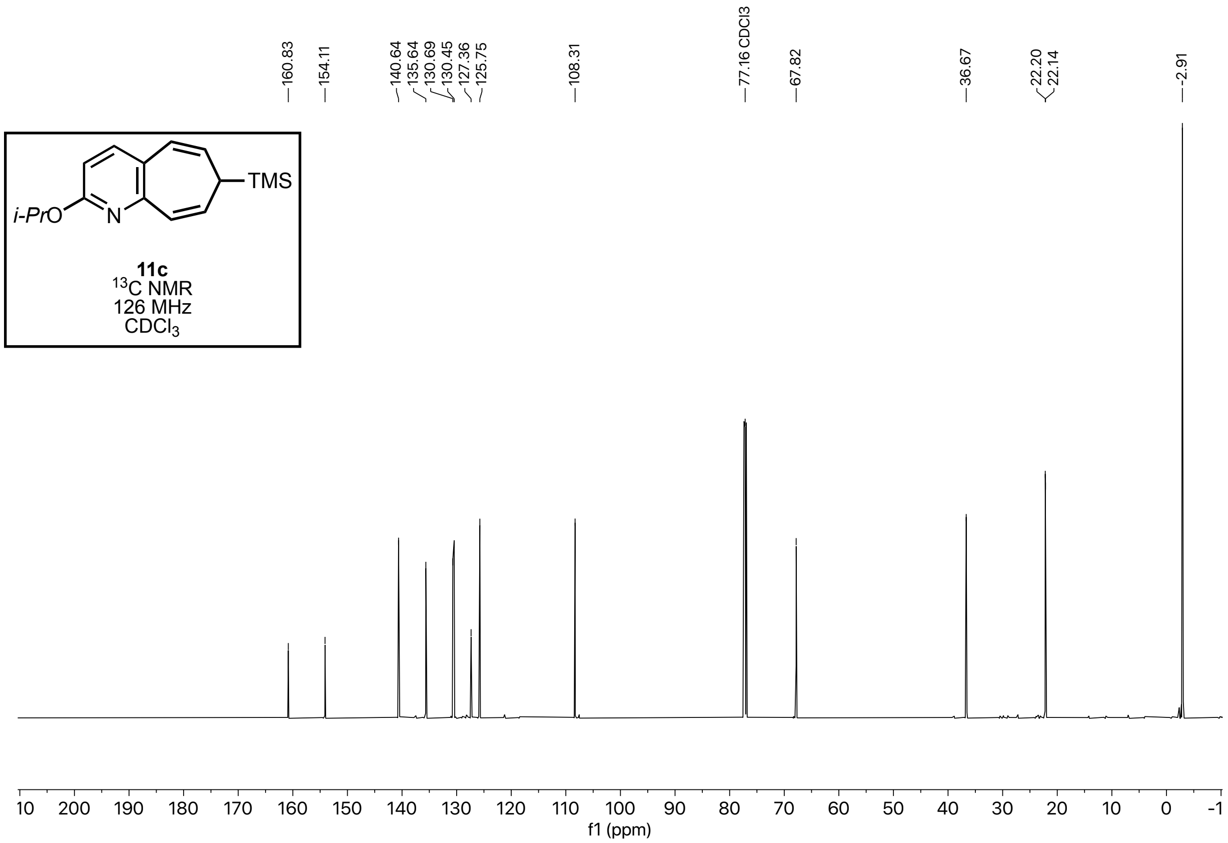
TMS
i-PrO
N
11c
13C NMR
126 MHz
CDCl3
160.83
154.11
140.64
135.64
130.69
130.45
127.36
125.75
108.31
77.16 CDCl3
67.82
36.67
22.20
22.14
-2.91
f1 (ppm)

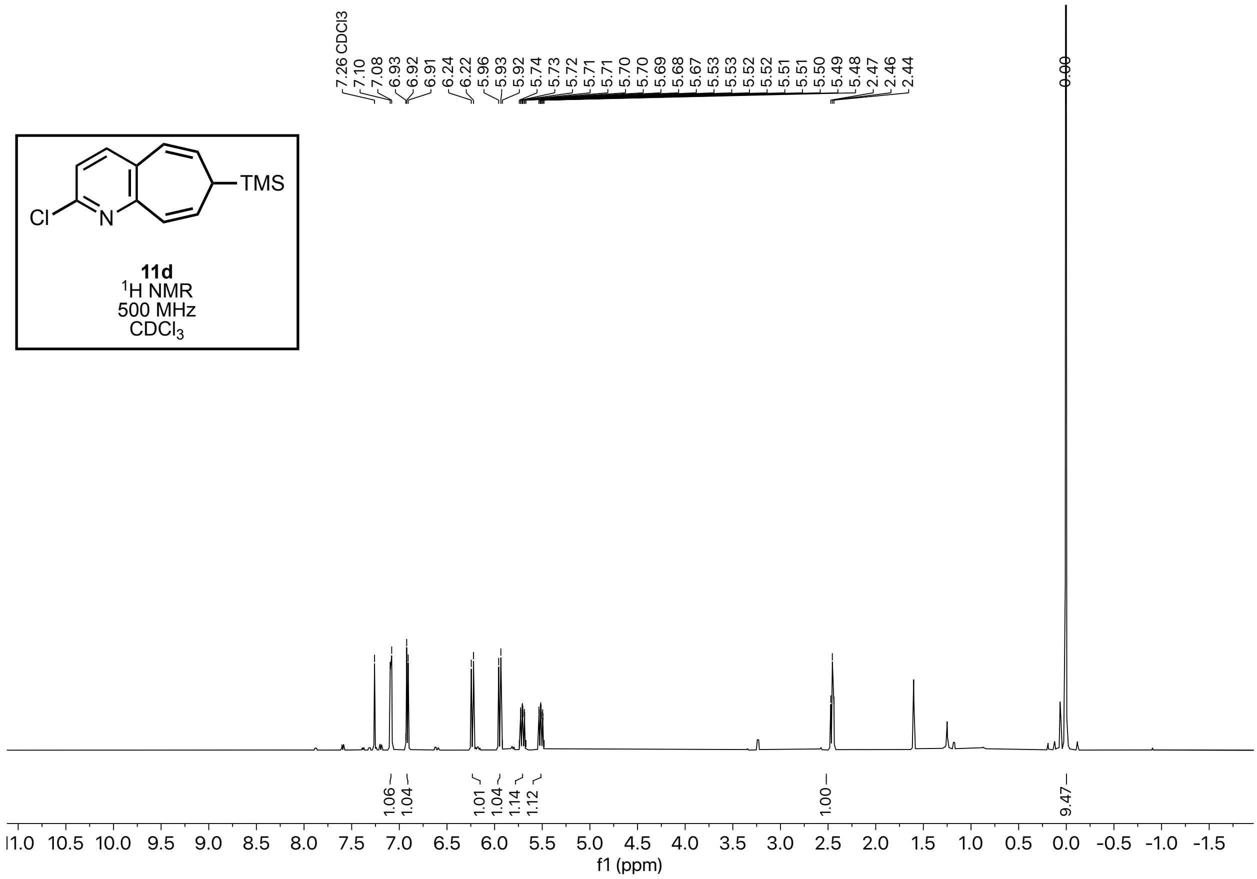
11d
$^1$H NMR
500 MHz
$CDCl_3$
TMS
Cl
N
7.26 CDCl3
0.00
f1 (ppm)

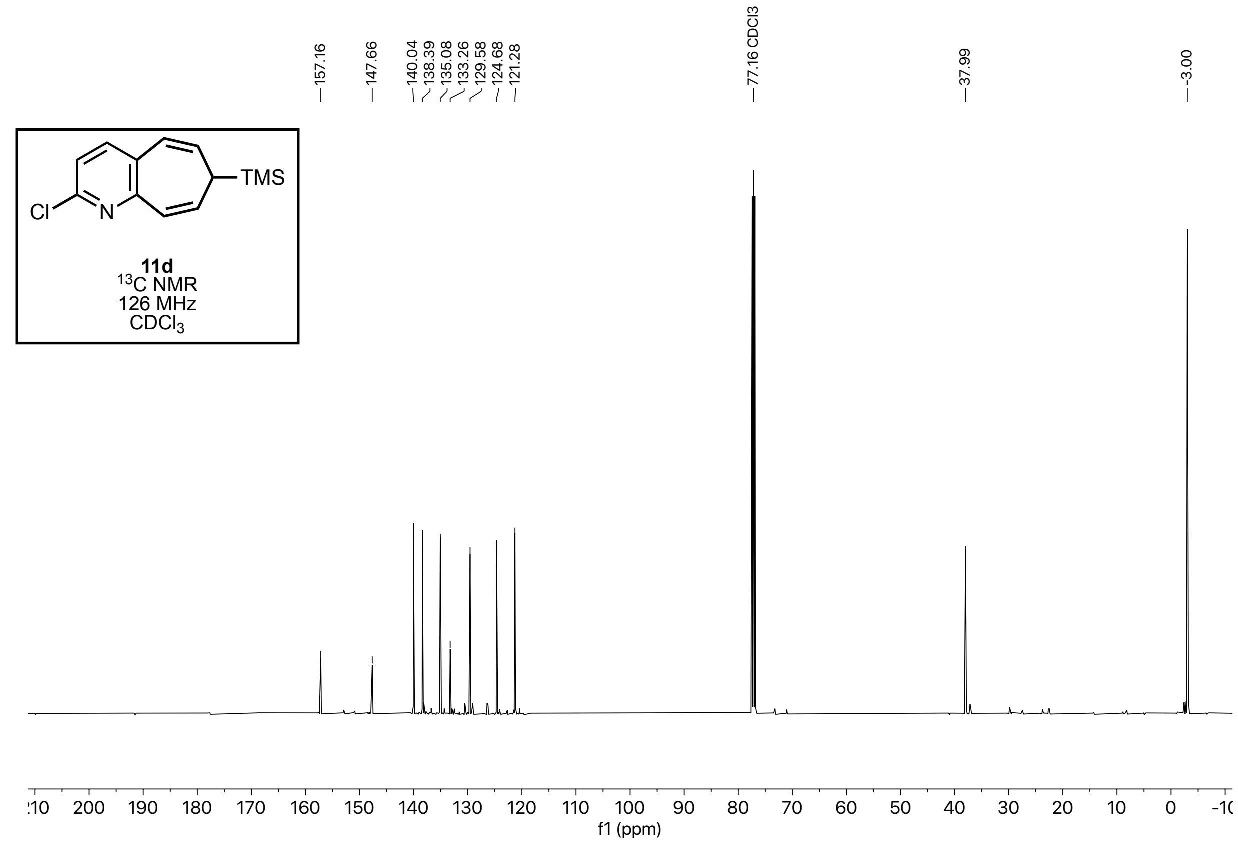
11d
$^{13}$C NMR
126 MHz
$CDCl_3$
TMS
Cl
N
157.16
147.66
140.04
138.39
135.08
133.26
129.58
124.68
121.28
77.16 CDCl3
37.99
-3.00
f1 (ppm)

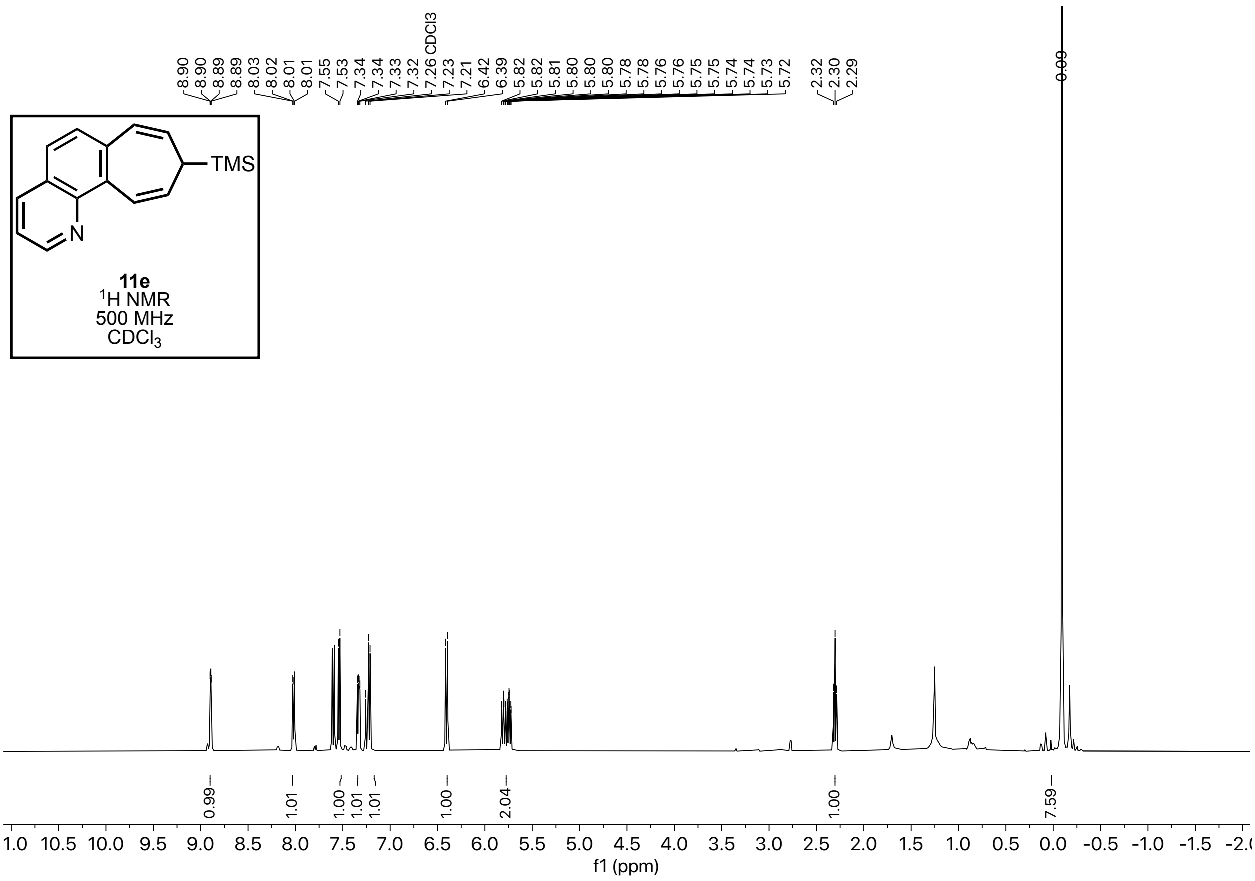
11e
1H NMR
500 MHz
CDCl3
TMS
N
f1 (ppm)

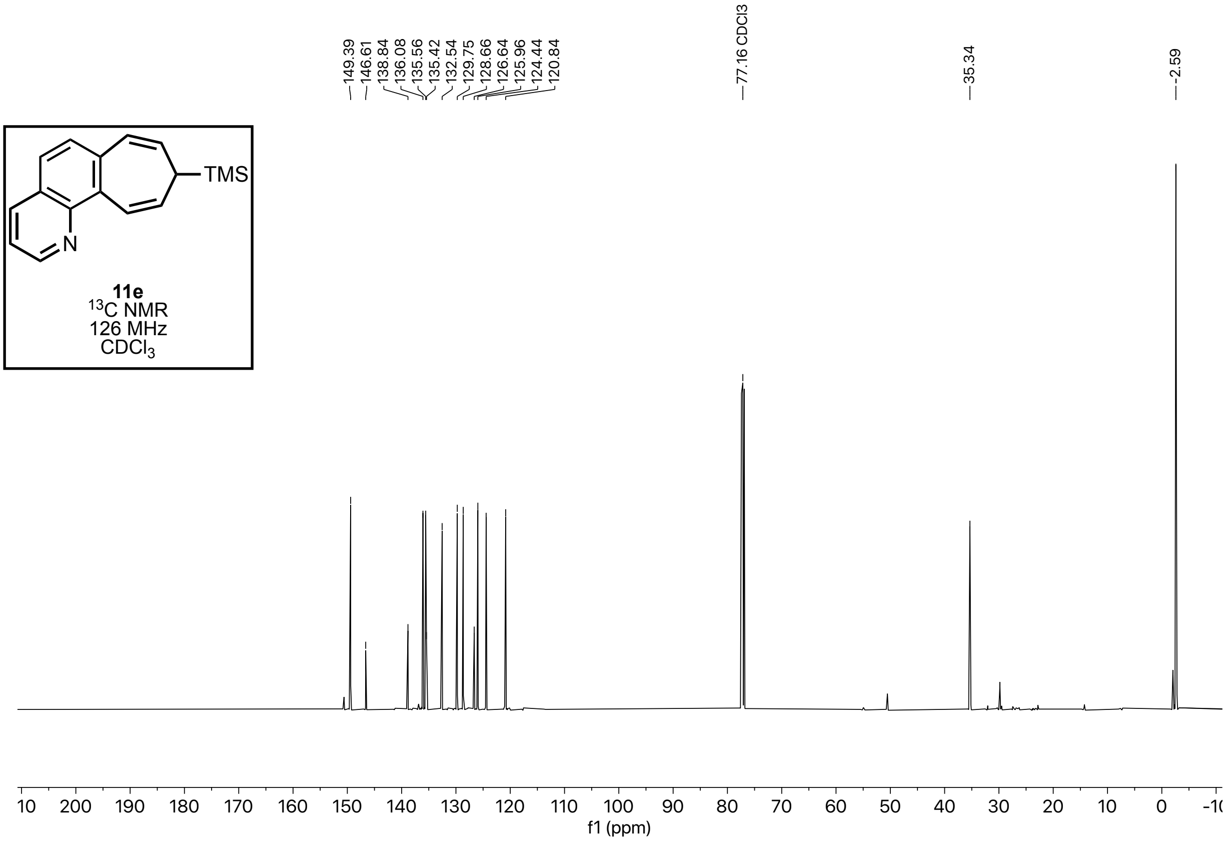
11e
13C NMR
126 MHz
CDCl3
TMS
N
f1 (ppm)

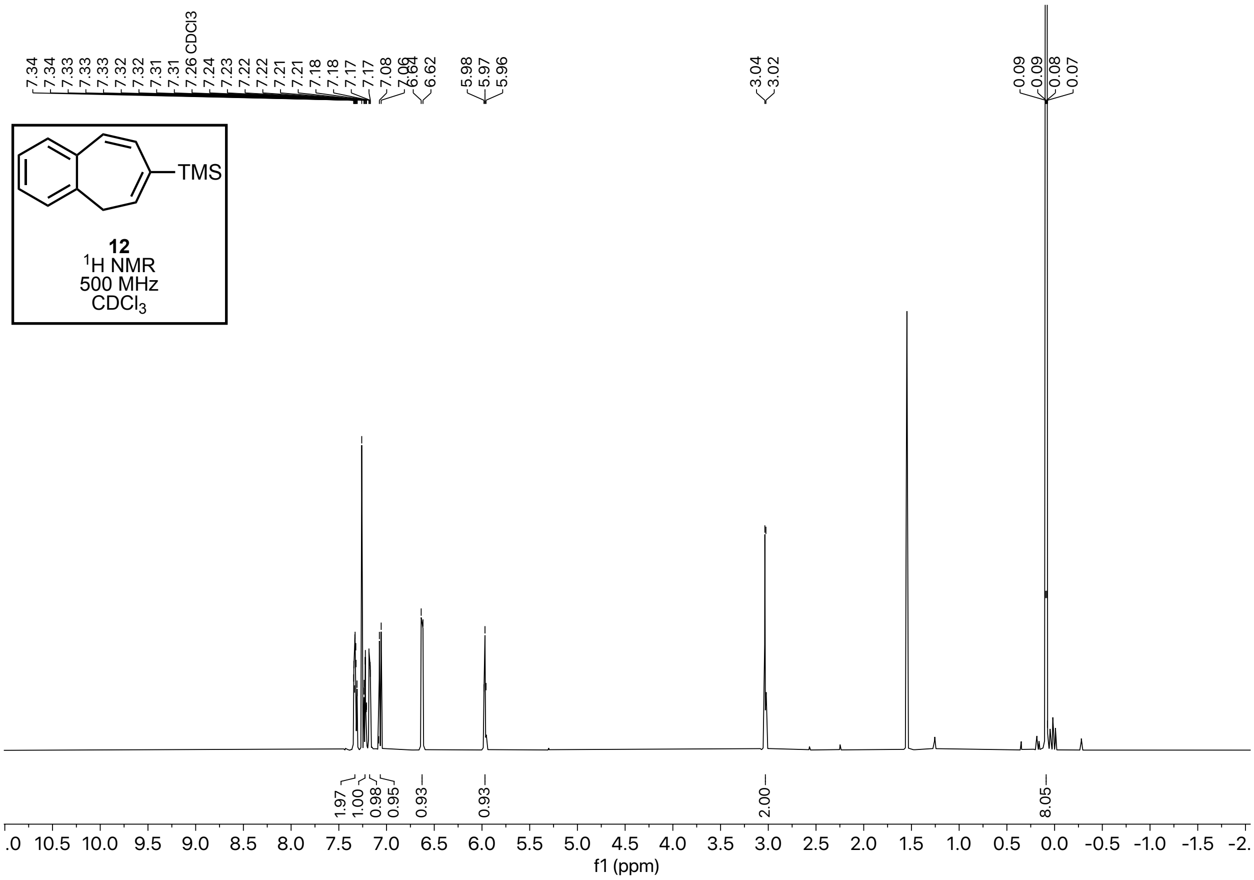
7.34
7.34
7.33
7.33
7.33
7.32
7.32
7.31
7.31
7.26 CDCl3
7.24
7.23
7.22
7.22
7.21
7.21
7.18
7.18
7.17
7.17
7.08
7.06
6.64
6.62
5.98
5.97
5.96
3.04
3.02
0.09
0.09
0.08
0.07
TMS
12
1H NMR
500 MHz
CDCl3
1.97
1.00
0.98
0.95
0.93
0.93
2.00
8.05
f1 (ppm)

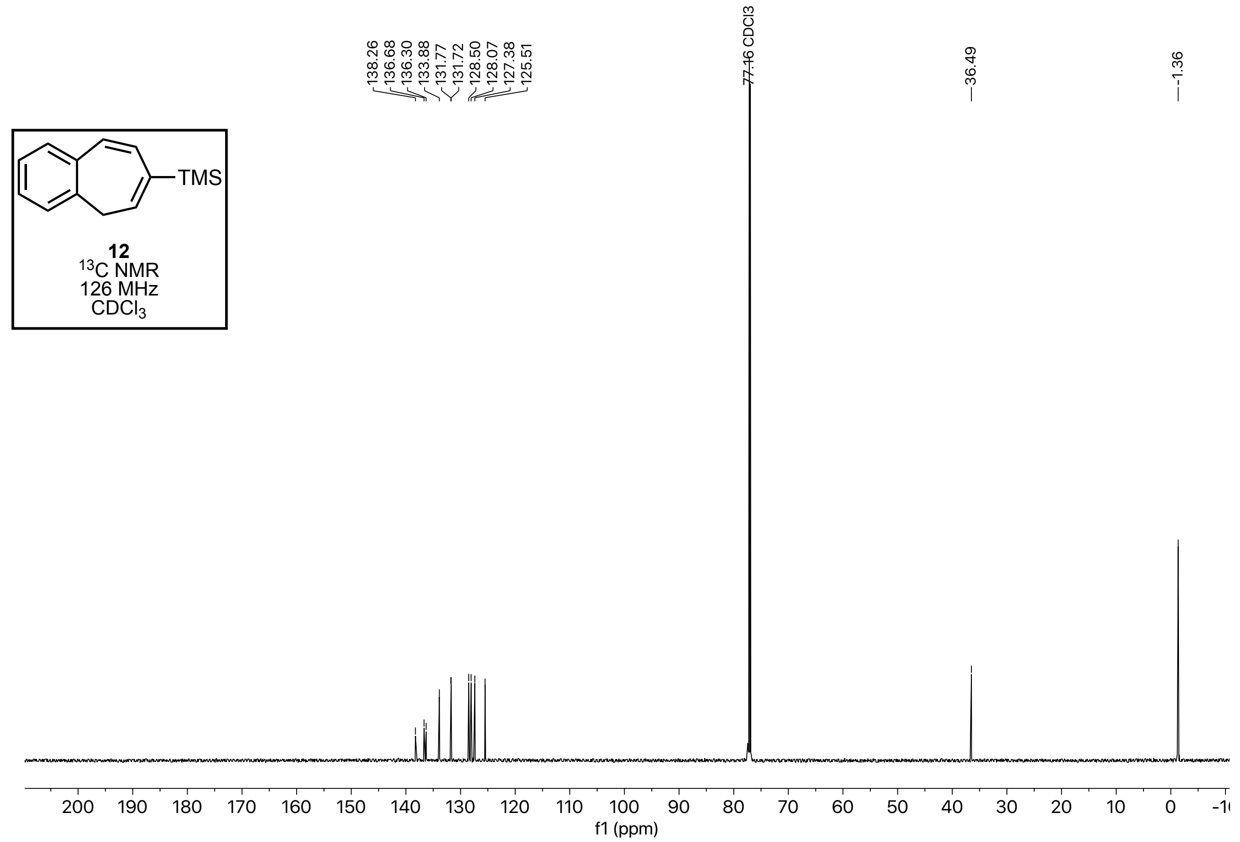
138.26
136.68
136.30
133.88
131.77
131.72
128.50
128.07
127.38
125.51
77.16 CDCl3
36.49
-1.36
TMS
12
13C NMR
126 MHz
CDCl3
f1 (ppm)

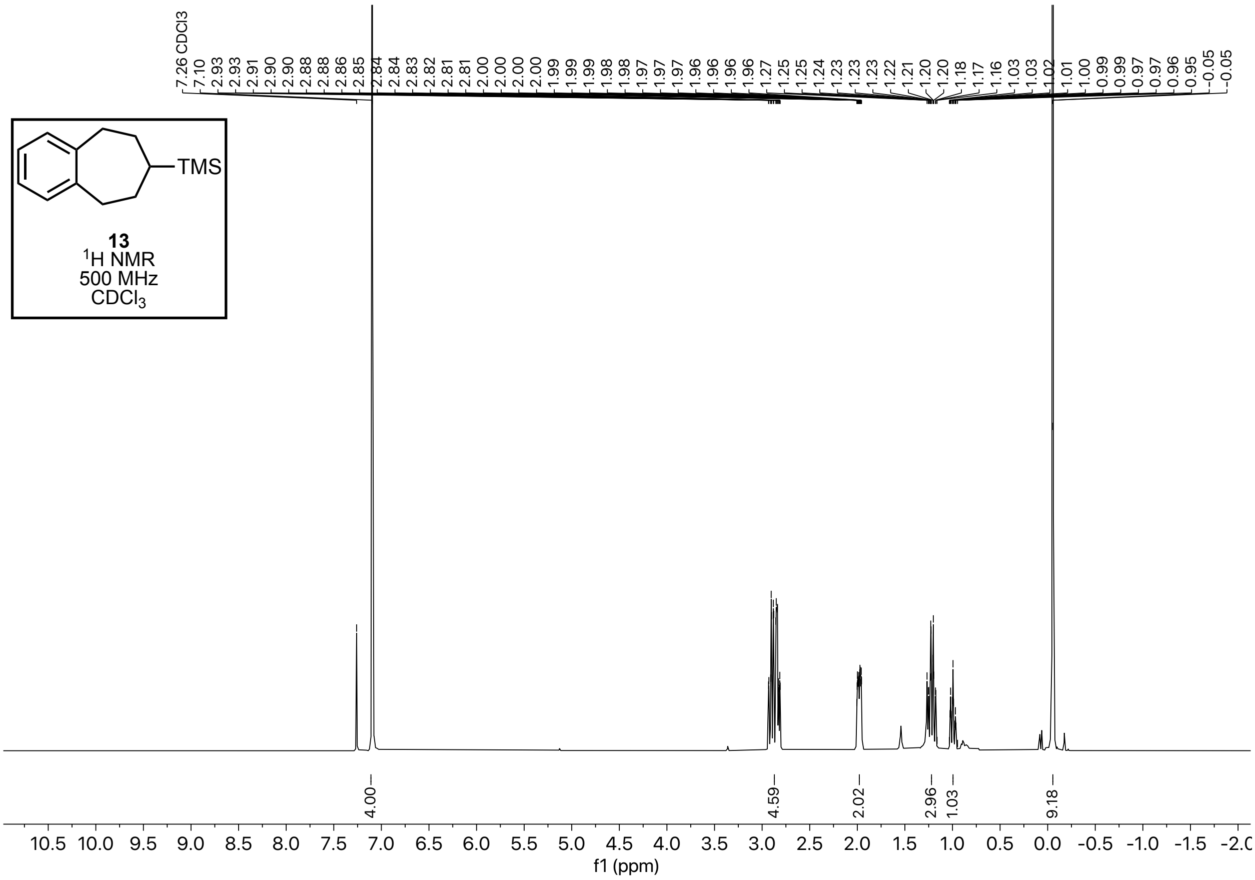
7.26 CDCl3
7.10
2.93
2.93
2.91
2.90
2.90
2.88
2.88
2.86
2.85
2.84
2.84
2.83
2.82
2.81
2.81
2.00
2.00
2.00
2.00
1.99
1.99
1.99
1.98
1.98
1.97
1.97
1.97
1.96
1.96
1.96
1.96
1.27
1.25
1.25
1.24
1.23
1.23
1.23
1.22
1.21
1.20
1.20
1.18
1.17
1.16
1.03
1.03
1.02
1.01
1.00
0.99
0.99
0.97
0.97
0.96
0.95
-0.05
-0.05
TMS
13
$^1$H NMR
500 MHz
$CDCl_3$
4.00
4.59
2.02
2.96
1.03
9.18
f1 (ppm)

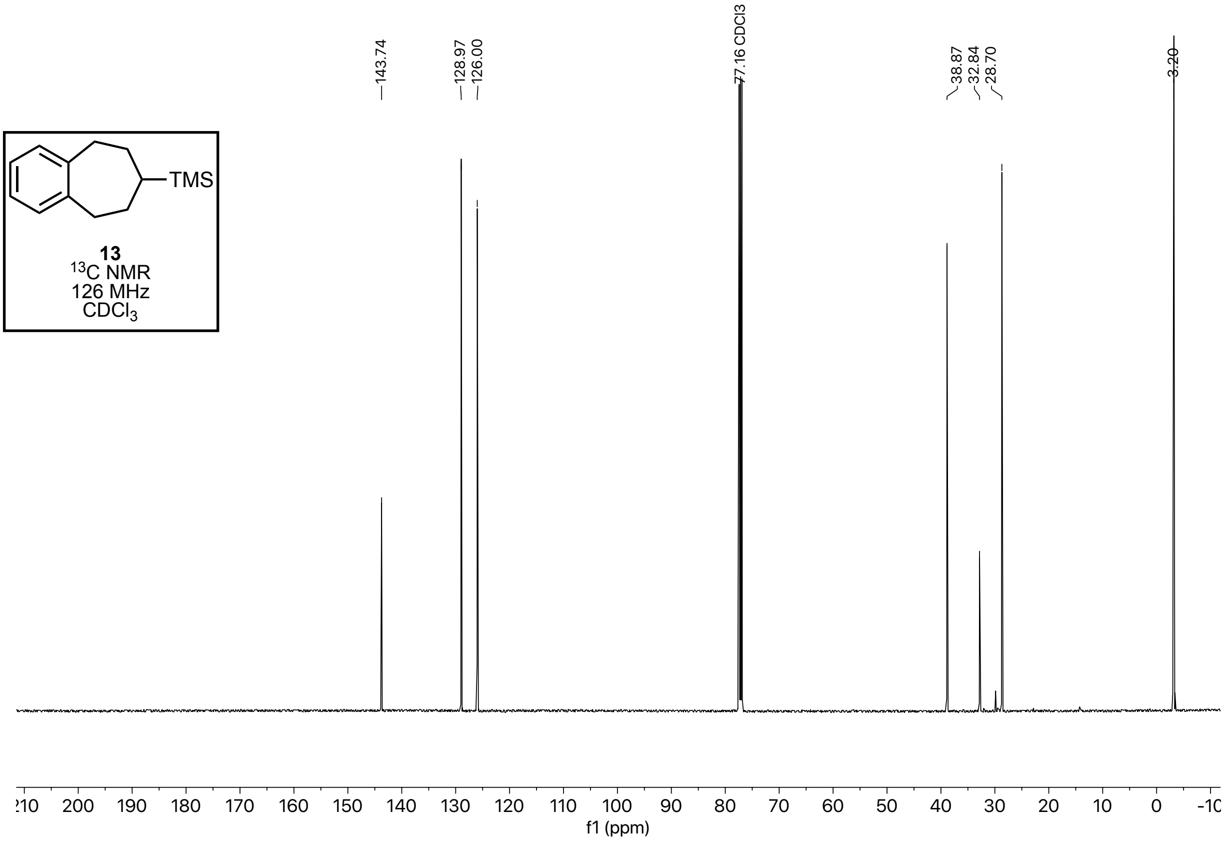
143.74
128.97
126.00
77.16 CDCl3
38.87
32.84
28.70
3.20
TMS
13
$^{13}$C NMR
126 MHz
$CDCl_3$
f1 (ppm)

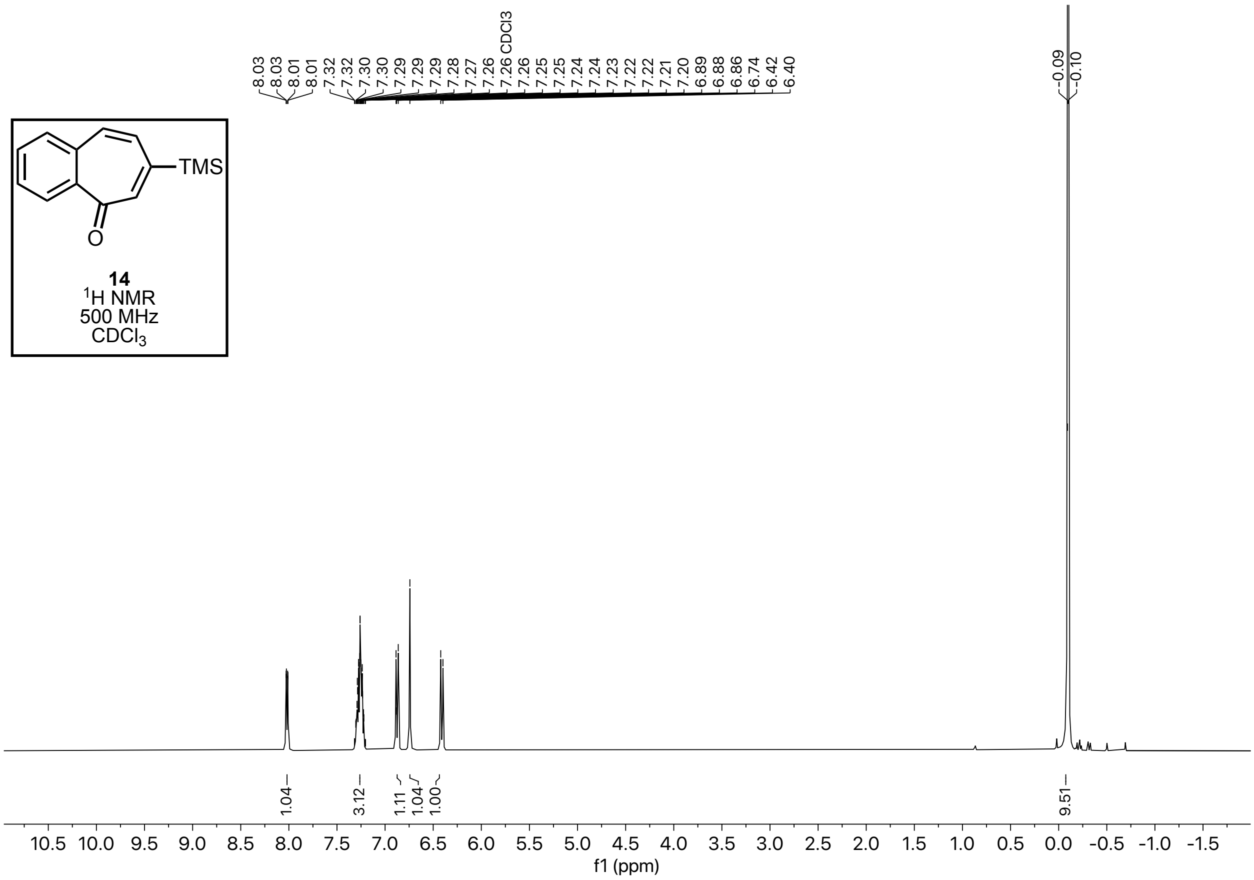
8.03
8.03
8.01
8.01
7.32
7.32
7.30
7.30
7.29
7.29
7.29
7.28
7.27
7.26 CDCl3
7.26
7.25
7.25
7.24
7.24
7.23
7.22
7.22
7.21
7.20
6.89
6.88
6.86
6.74
6.42
6.40
-0.09
-0.10
TMS
O
14
1H NMR
500 MHz
CDCl3
1.04
3.12
1.11
1.04
1.00
9.51
10.5 10.0 9.5 9.0 8.5 8.0 7.5 7.0 6.5 6.0 5.5 5.0 4.5 4.0 3.5 3.0 2.5 2.0 1.5 1.0 0.5 0.0 -0.5 -1.0 -1.5
f1 (ppm)

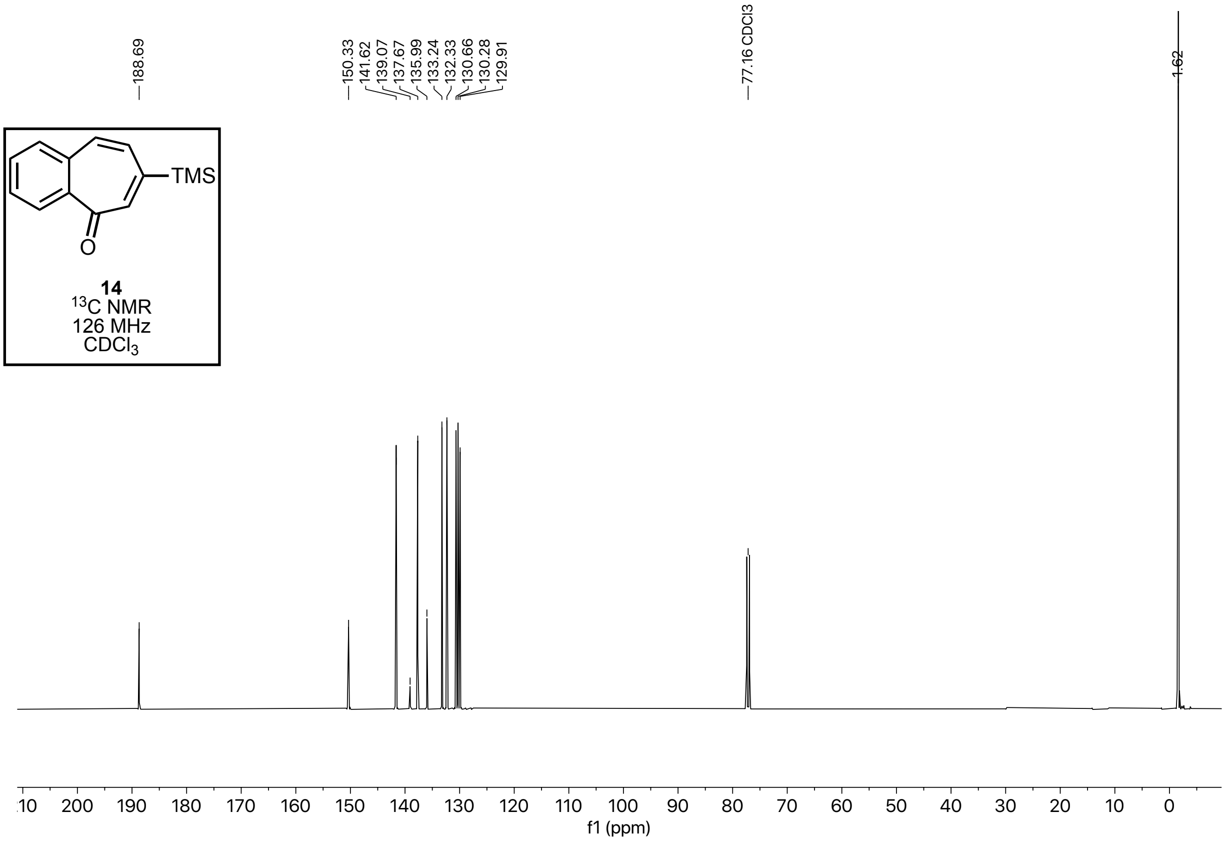
188.69
150.33
141.62
139.07
137.67
135.99
133.24
132.33
130.66
130.28
129.91
77.16 CDCl3
1.62
TMS
O
14
13C NMR
126 MHz
CDCl3
10 200 190 180 170 160 150 140 130 120 110 100 90 80 70 60 50 40 30 20 10 0
f1 (ppm)

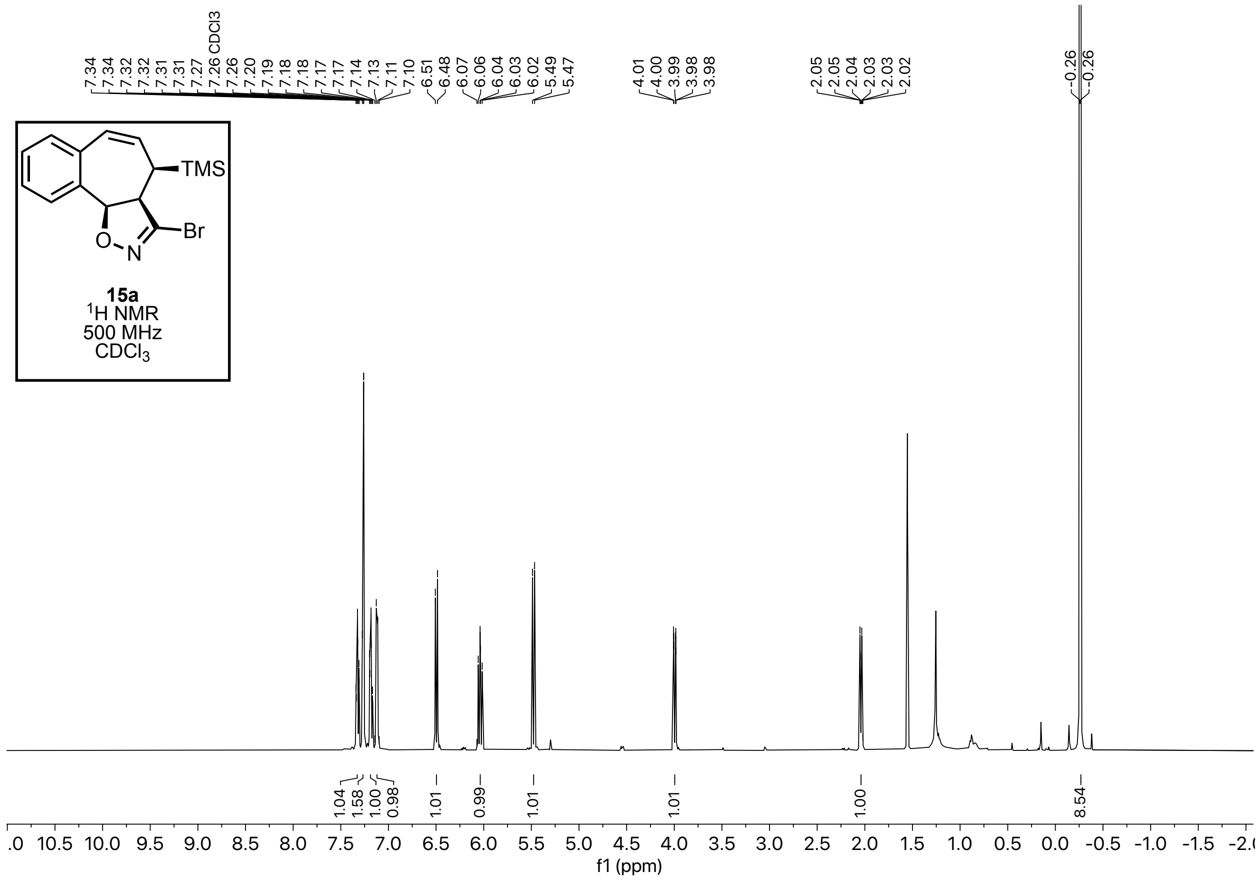
7.34
7.34
7.32
7.32
7.31
7.31
7.27
7.26 CDCl3
7.26
7.20
7.19
7.18
7.18
7.17
7.17
7.14
7.13
7.11
7.10
6.51
6.48
6.07
6.06
6.04
6.03
6.02
5.49
5.47
4.01
4.00
3.99
3.98
3.98
2.05
2.05
2.04
2.03
2.03
2.02
-0.26
-0.26
TMS
Br
O
N
15a
1H NMR
500 MHz
CDCl3
1.04
1.58
1.00
0.98
1.01
0.99
1.01
1.01
1.00
8.54
f1 (ppm)

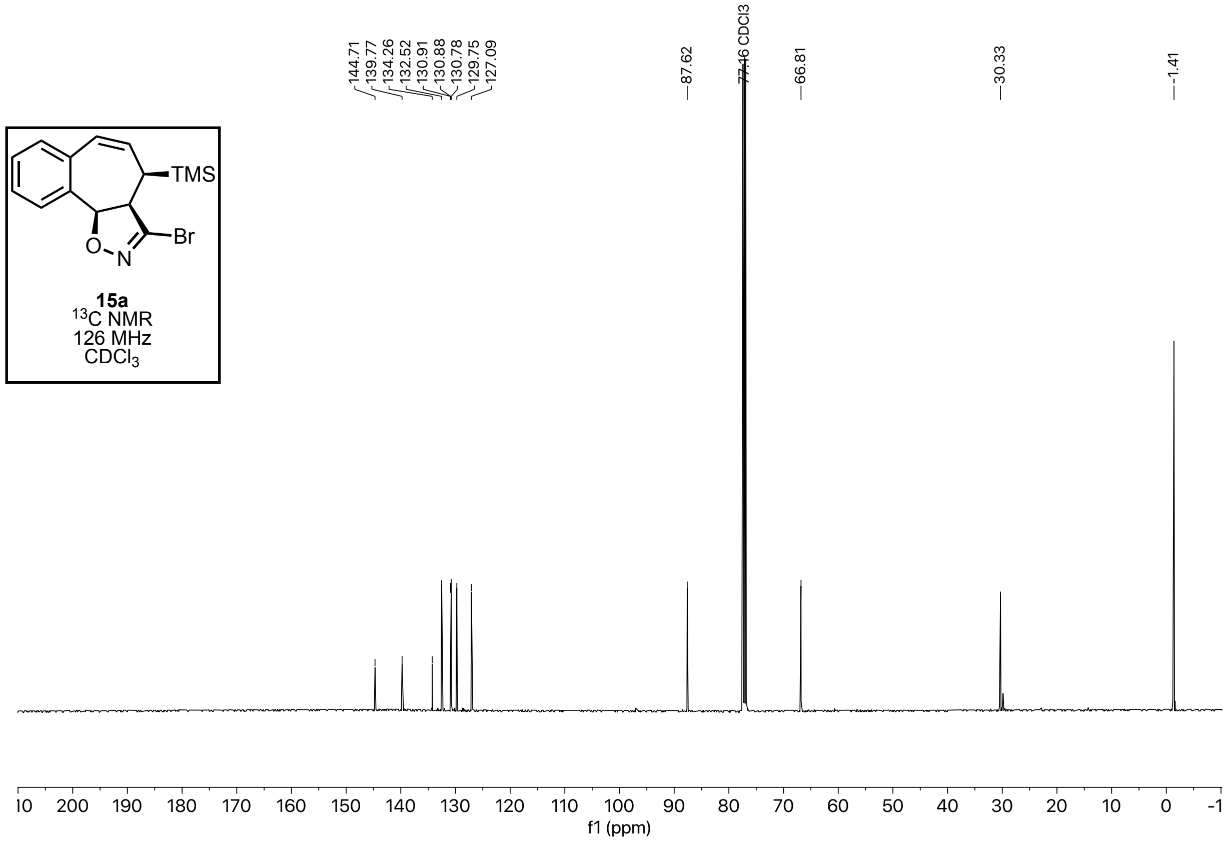
144.71
139.77
134.26
132.52
130.91
130.88
130.78
129.75
127.09
87.62
77.16 CDCl3
66.81
30.33
-1.41
TMS
Br
O
N
15a
13C NMR
126 MHz
CDCl3
f1 (ppm)

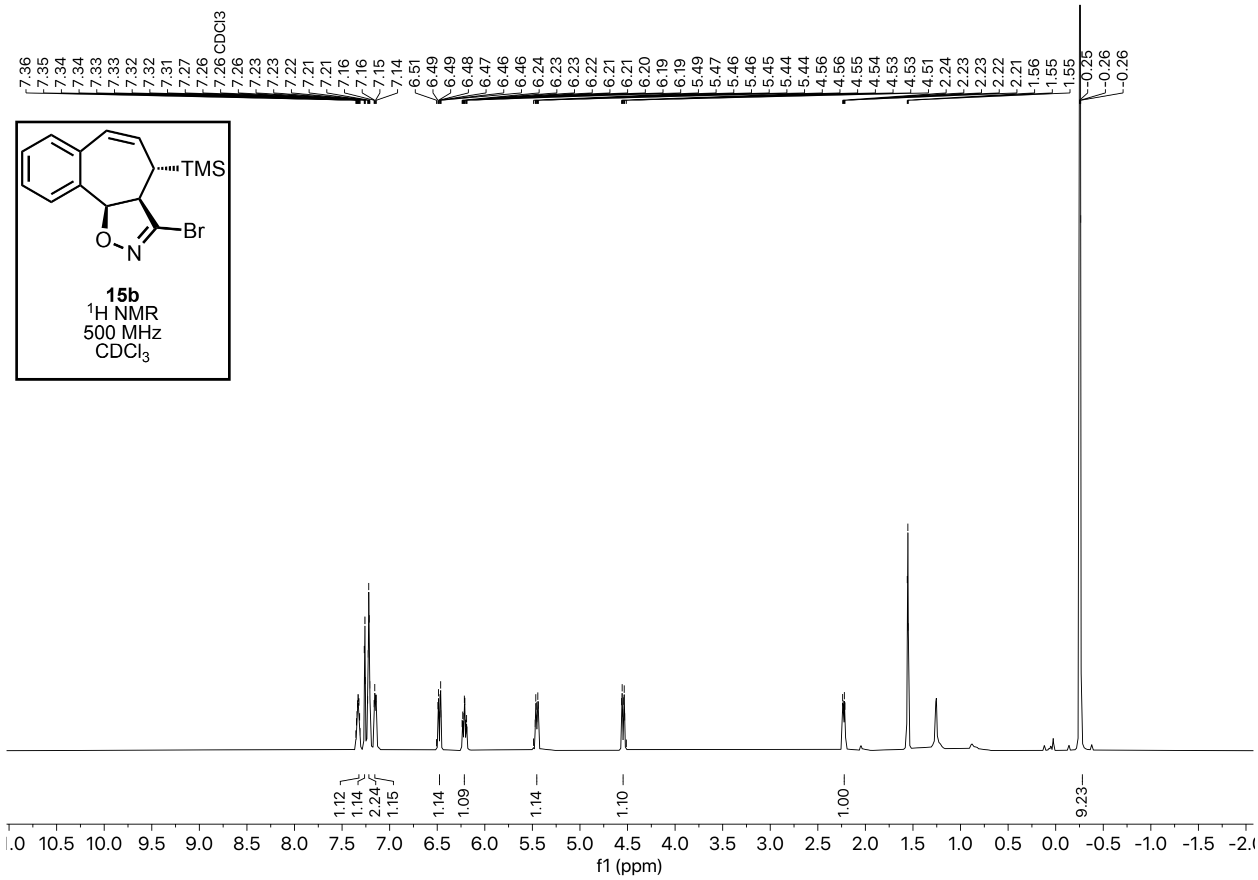
TMS
Br
O
N
15b
1H NMR
500 MHz
CDCl3
f1 (ppm)

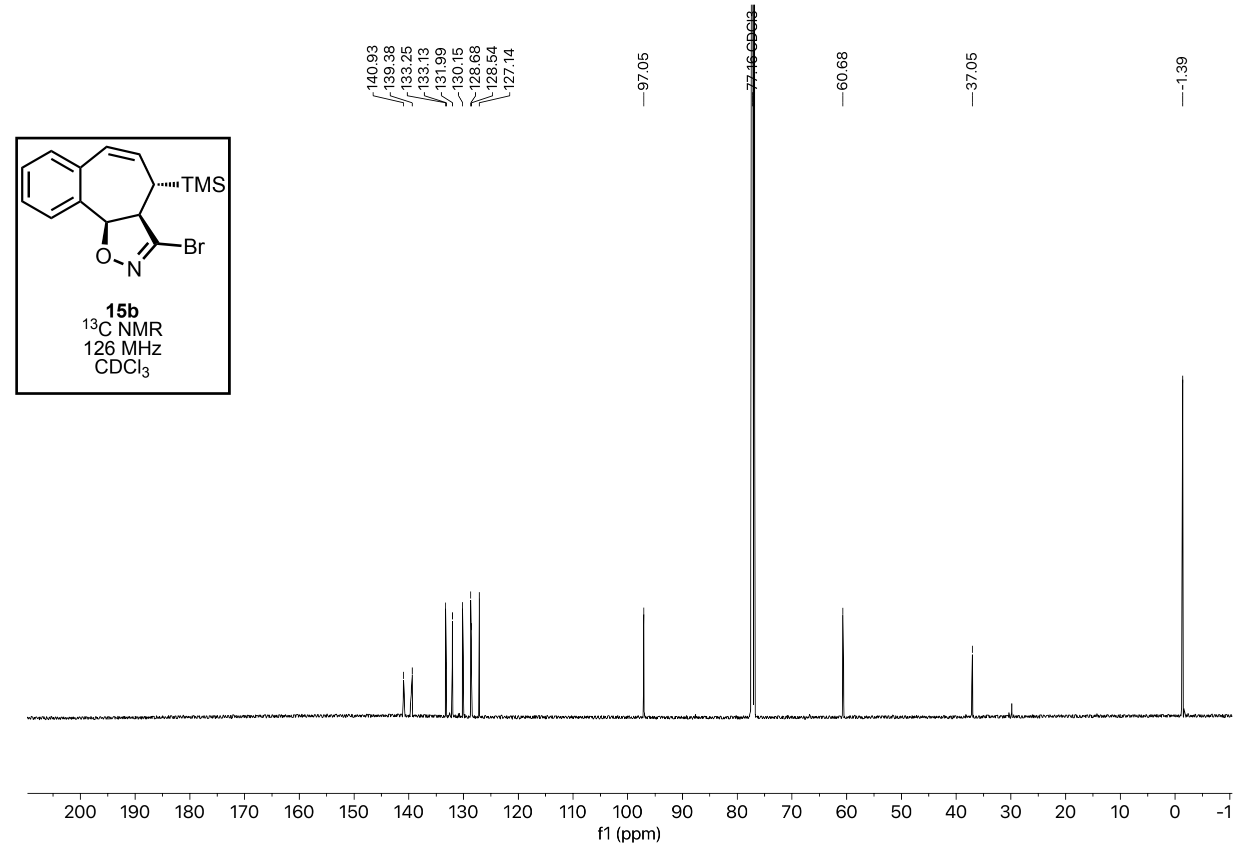
TMS
Br
O
N
15b
13C NMR
126 MHz
CDCl3
140.93
139.38
133.25
133.13
131.99
130.15
128.68
128.54
127.14
97.05
77.16 CDCl3
60.68
37.05
-1.39
f1 (ppm)

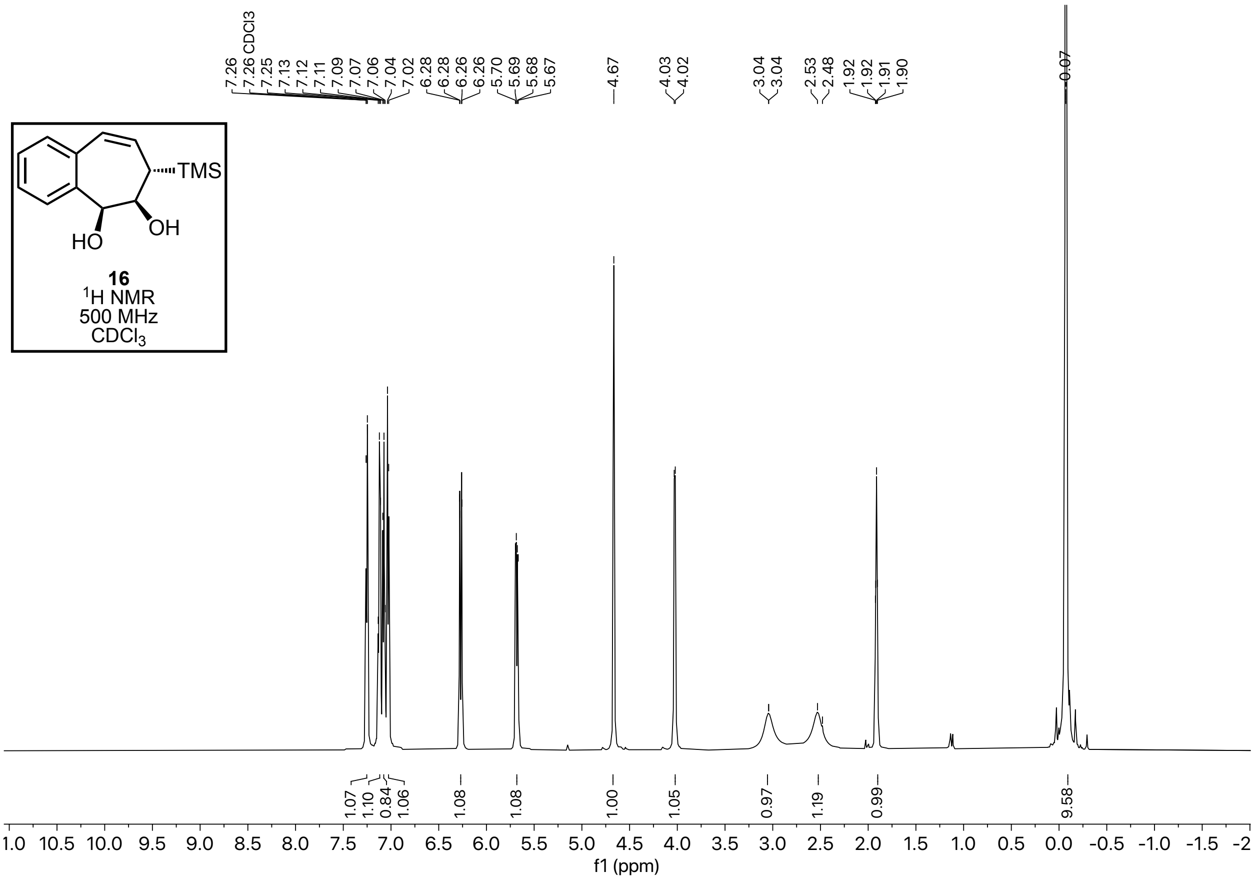
7.26
7.26 CDCl3
7.25
7.13
7.12
7.11
7.09
7.07
7.06
7.04
7.02
6.28
6.28
6.26
6.26
5.70
5.69
5.68
5.67
4.67
4.03
4.02
3.04
3.04
2.53
2.48
1.92
1.92
1.91
1.90
0.07
16
$^1$H NMR
500 MHz
$CDCl_3$
1.07
1.10
0.84
1.06
1.08
1.08
1.00
1.05
0.97
1.19
0.99
9.58
f1 (ppm)

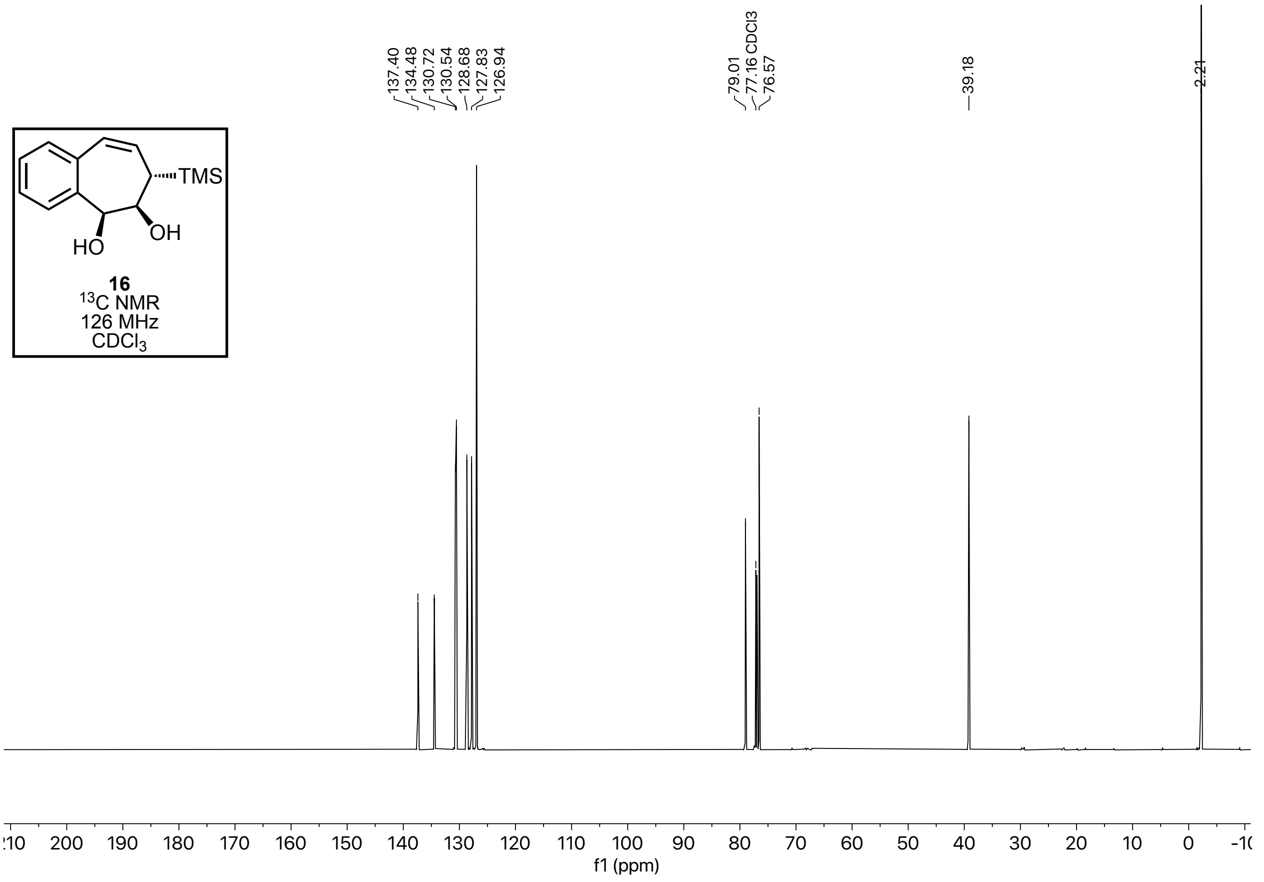
137.40
134.48
130.72
130.54
128.68
127.83
126.94
79.01
77.16 CDCl3
76.57
39.18
2.21
16
$^{13}$C NMR
126 MHz
$CDCl_3$
f1 (ppm)

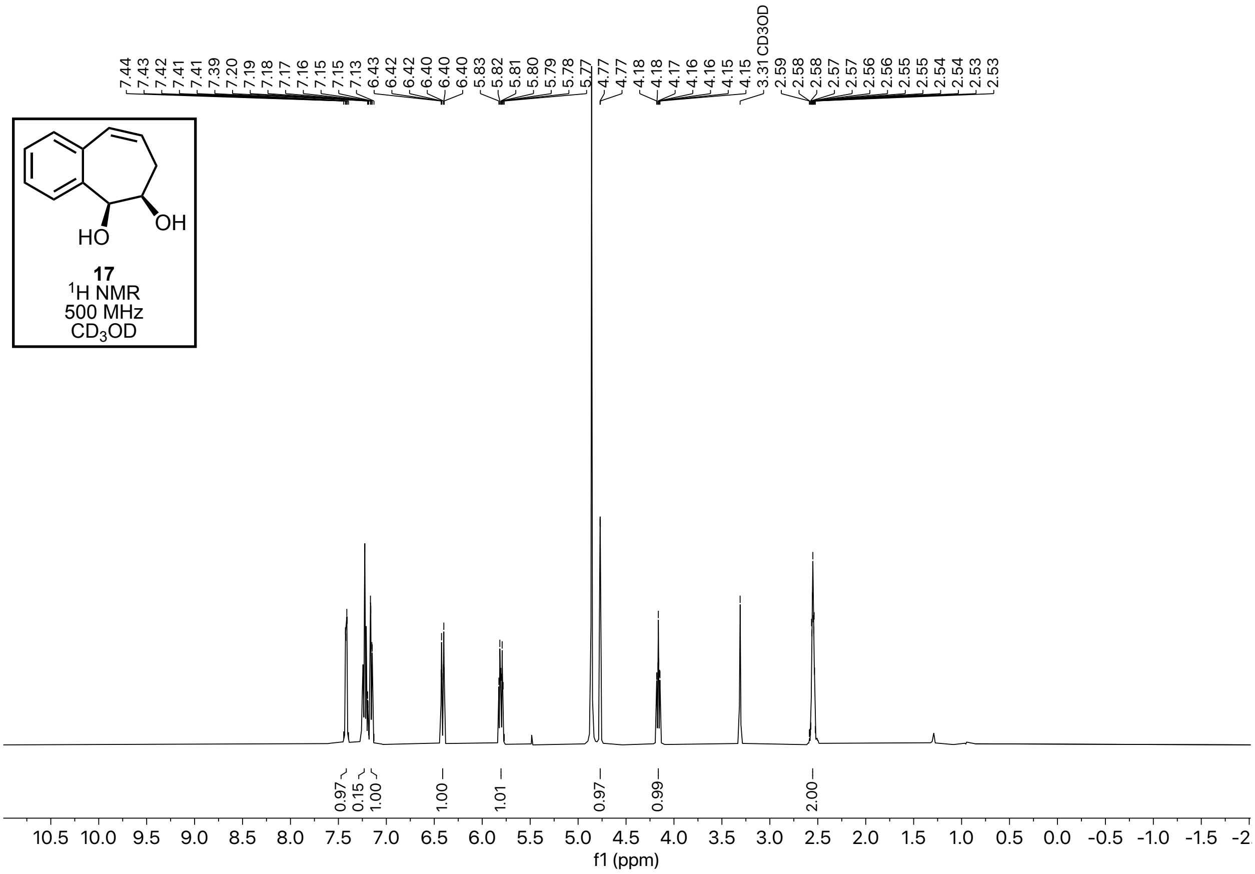
HO
OH
17
$^{1}$H NMR
500 MHz
$CD_3OD$
7.44 7.43 7.42 7.41 7.41 7.39 7.20 7.19 7.18 7.17 7.16 7.15 7.15 7.13 6.43 6.42 6.42 6.40 6.40 6.40 5.83 5.82 5.81 5.80 5.79 5.78 5.77 4.77 4.77 4.18 4.18 4.17 4.16 4.16 4.15 4.15 3.31 CD3OD 2.59 2.58 2.58 2.57 2.57 2.56 2.56 2.55 2.55 2.54 2.54 2.53 2.53
0.97 0.15 1.00 1.00 1.01 0.97 0.99 2.00
f1 (ppm)

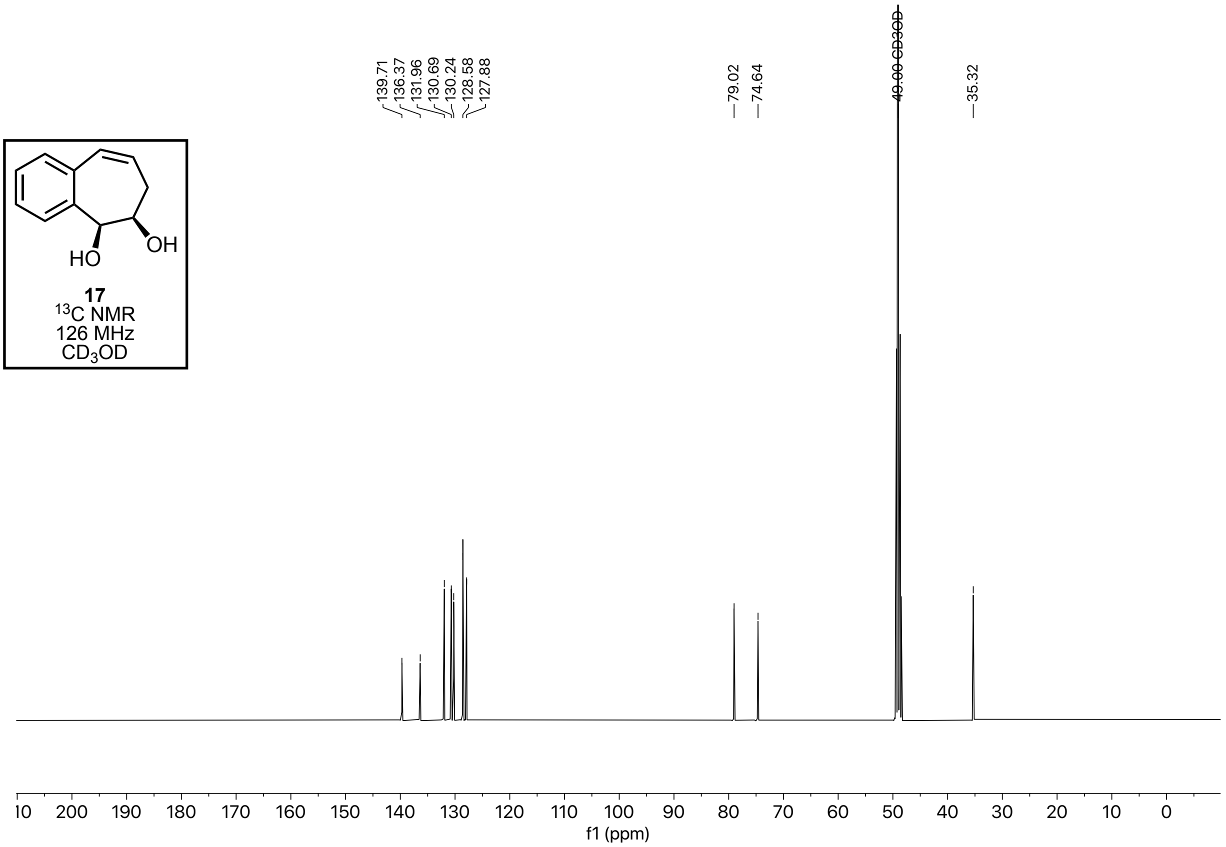
HO
OH
17
$^{13}$C NMR
126 MHz
$CD_3OD$
139.71 136.37 131.96 130.69 130.24 128.58 127.88
79.02
74.64
49.00 CD3OD
35.32
f1 (ppm)

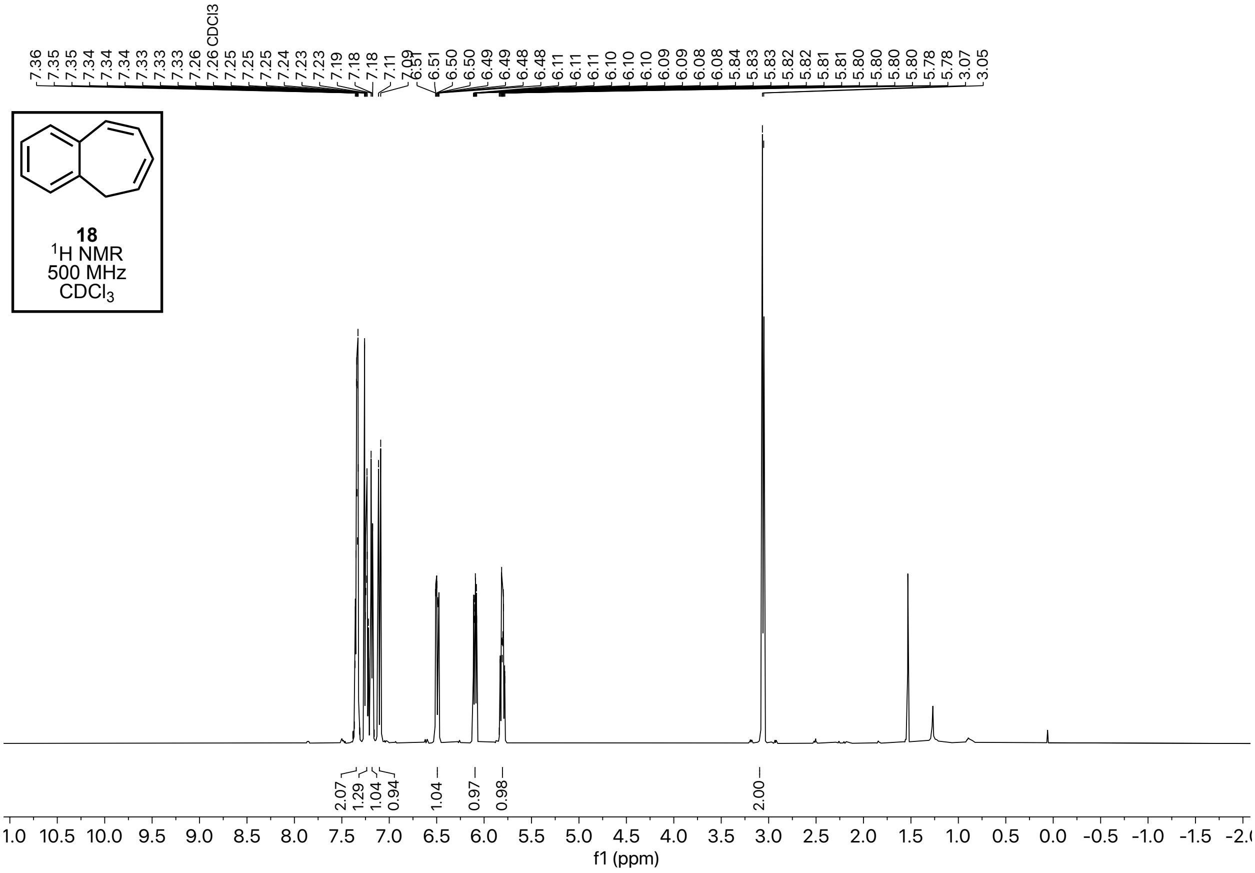
18
$^{1}$H NMR
500 MHz
$CDCl_3$
7.36 7.35 7.35 7.34 7.34 7.34 7.33 7.33 7.33 7.33 7.26 7.26 CDCl3 7.25 7.25 7.25 7.25 7.24 7.23 7.23 7.19 7.18 7.18 7.11 7.09 6.51 6.51 6.50 6.50 6.49 6.49 6.48 6.48 6.11 6.11 6.11 6.10 6.10 6.10 6.09 6.09 6.08 6.08 5.84 5.83 5.83 5.82 5.82 5.81 5.81 5.80 5.80 5.80 5.80 5.78 3.07 3.05
2.07 1.29 1.04 0.94 1.04 0.97 0.98 2.00
f1 (ppm)

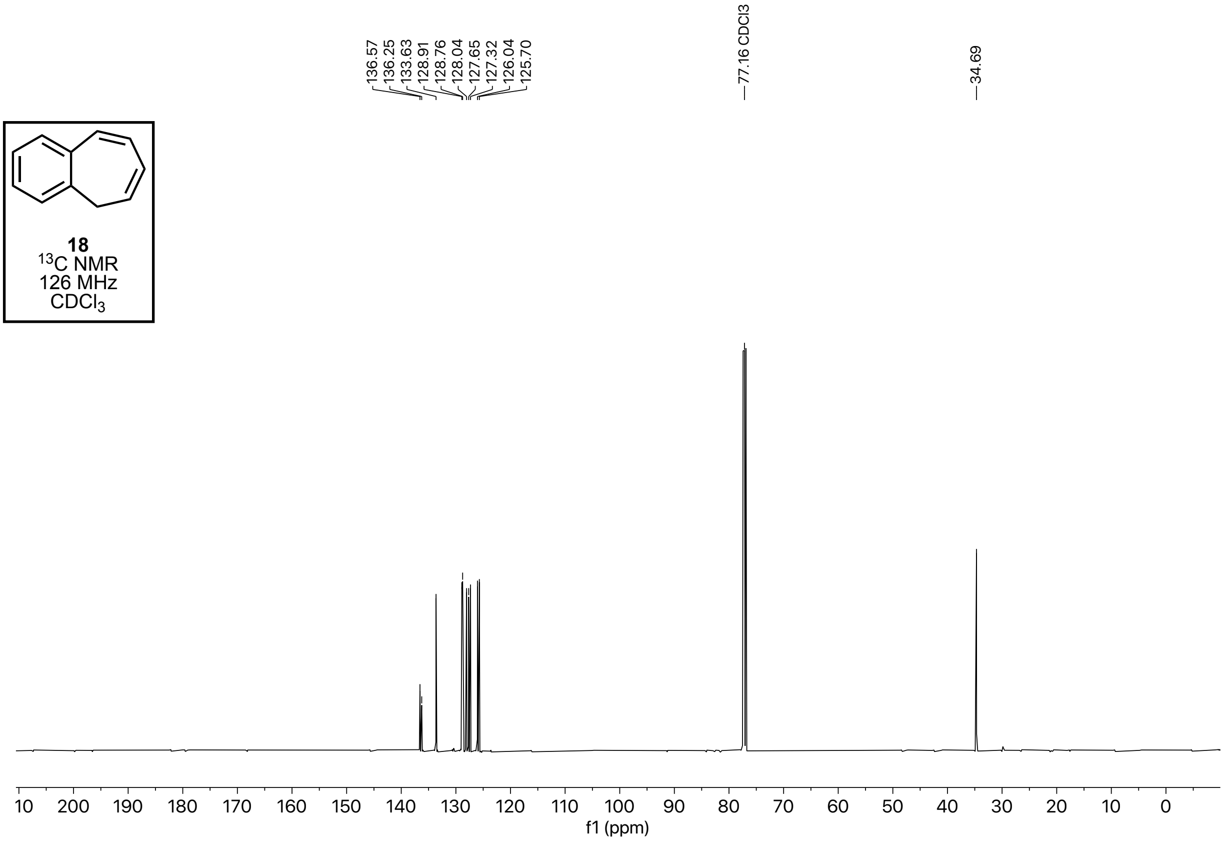
18
$^{13}$C NMR
126 MHz
$CDCl_3$
136.57 136.25 133.63 128.91 128.76 128.04 127.65 127.32 126.04 125.70
77.16 CDCl3
34.69
f1 (ppm)

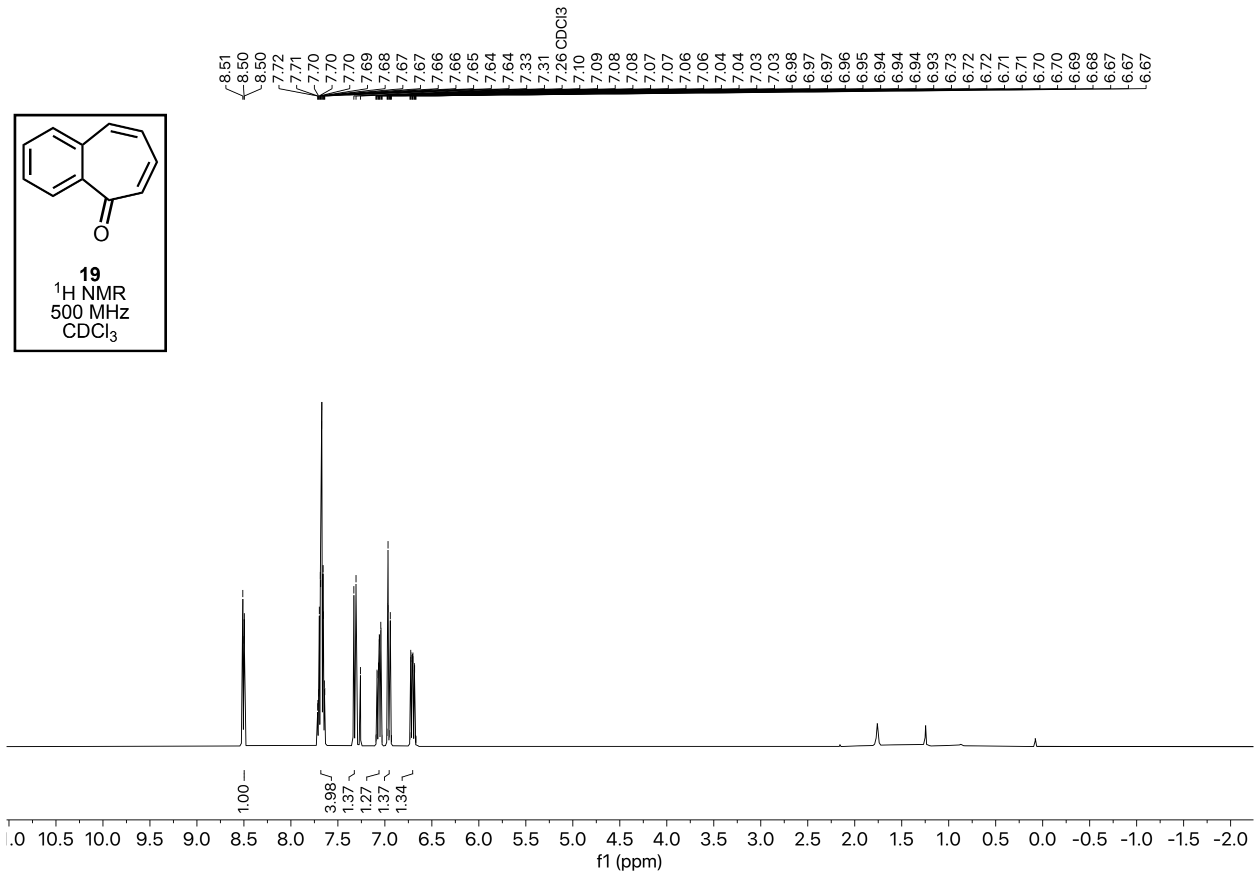

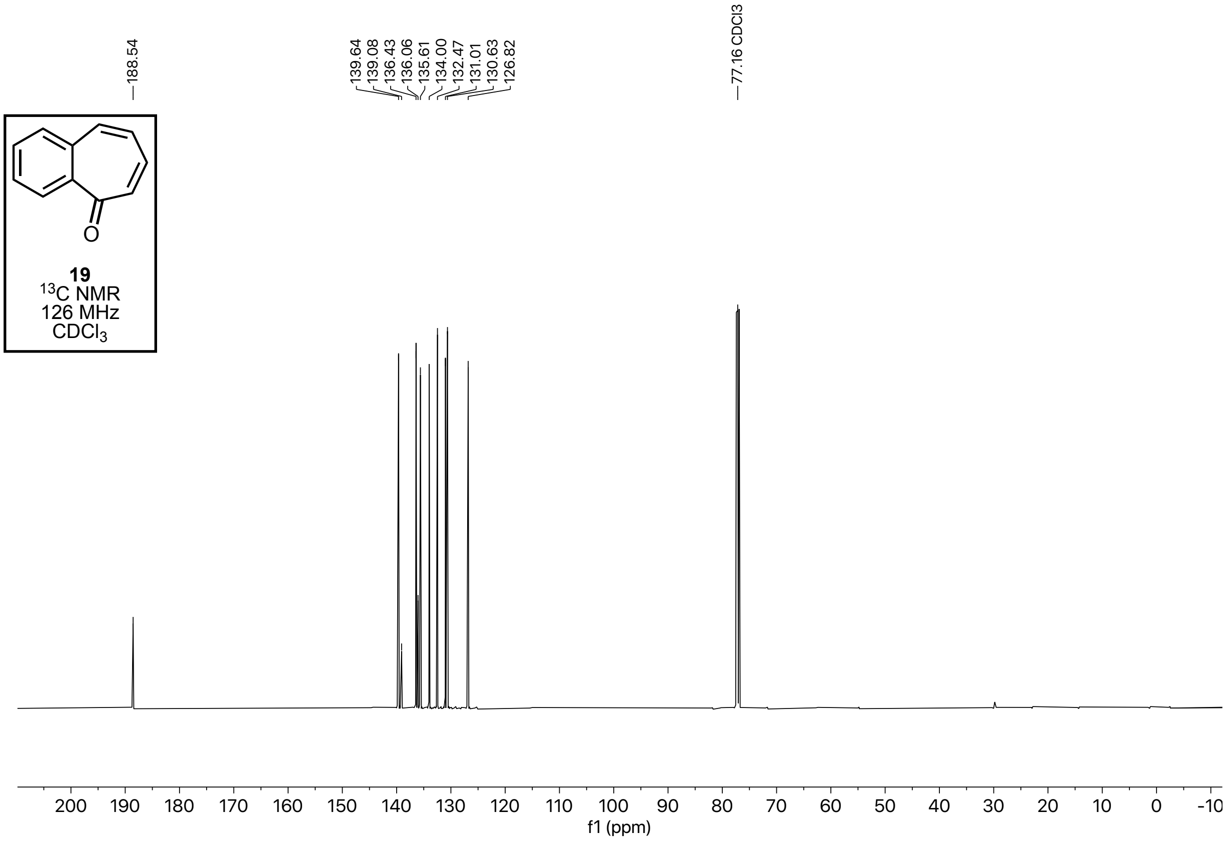

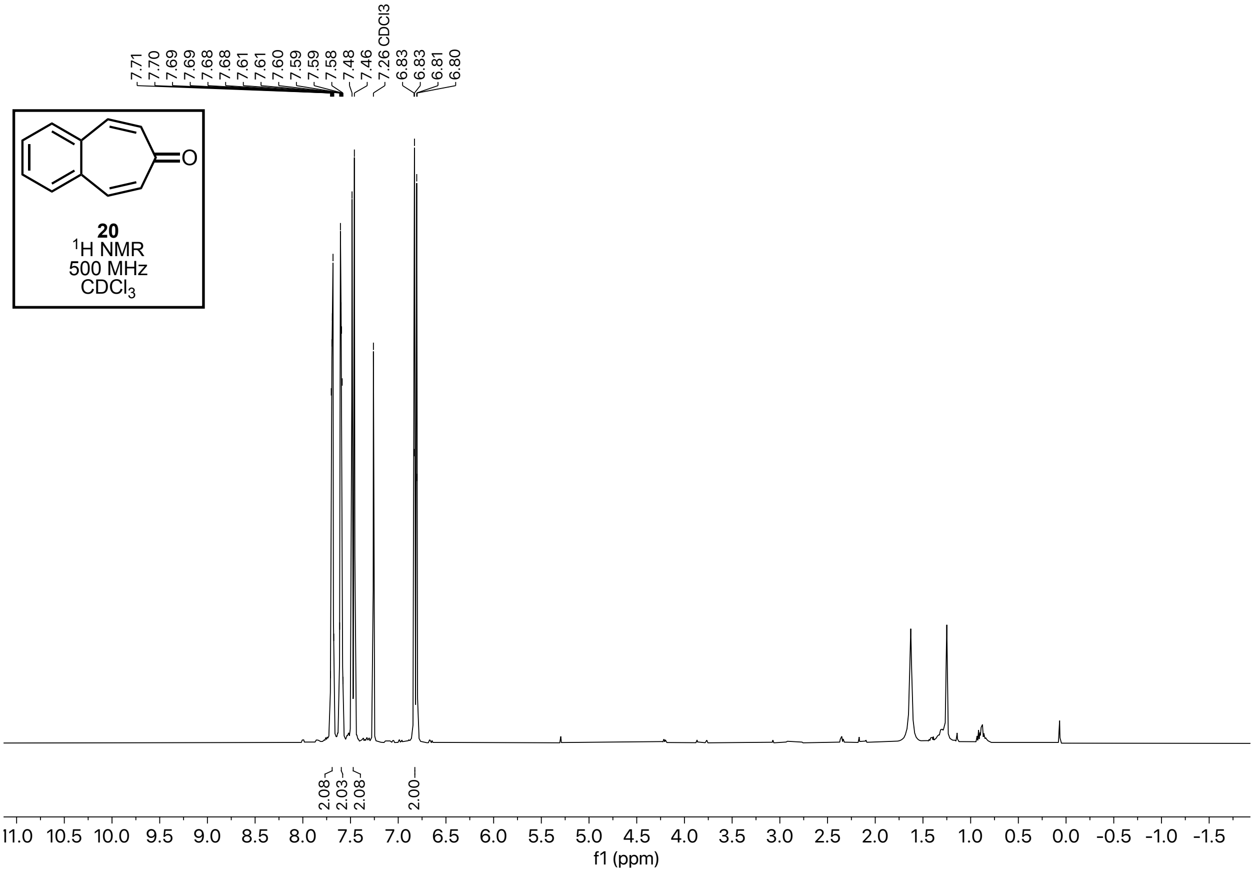
7.71
7.70
7.69
7.69
7.68
7.68
7.61
7.61
7.60
7.59
7.59
7.58
7.48
7.46
7.26 CDCl3
6.83
6.83
6.81
6.80
20
$^{1}$H NMR
500 MHz
$CDCl_3$
2.08
2.03
2.08
2.00
11.0 10.5 10.0 9.5 9.0 8.5 8.0 7.5 7.0 6.5 6.0 5.5 5.0 4.5 4.0 3.5 3.0 2.5 2.0 1.5 1.0 0.5 0.0 -0.5 -1.0 -1.5
f1 (ppm)

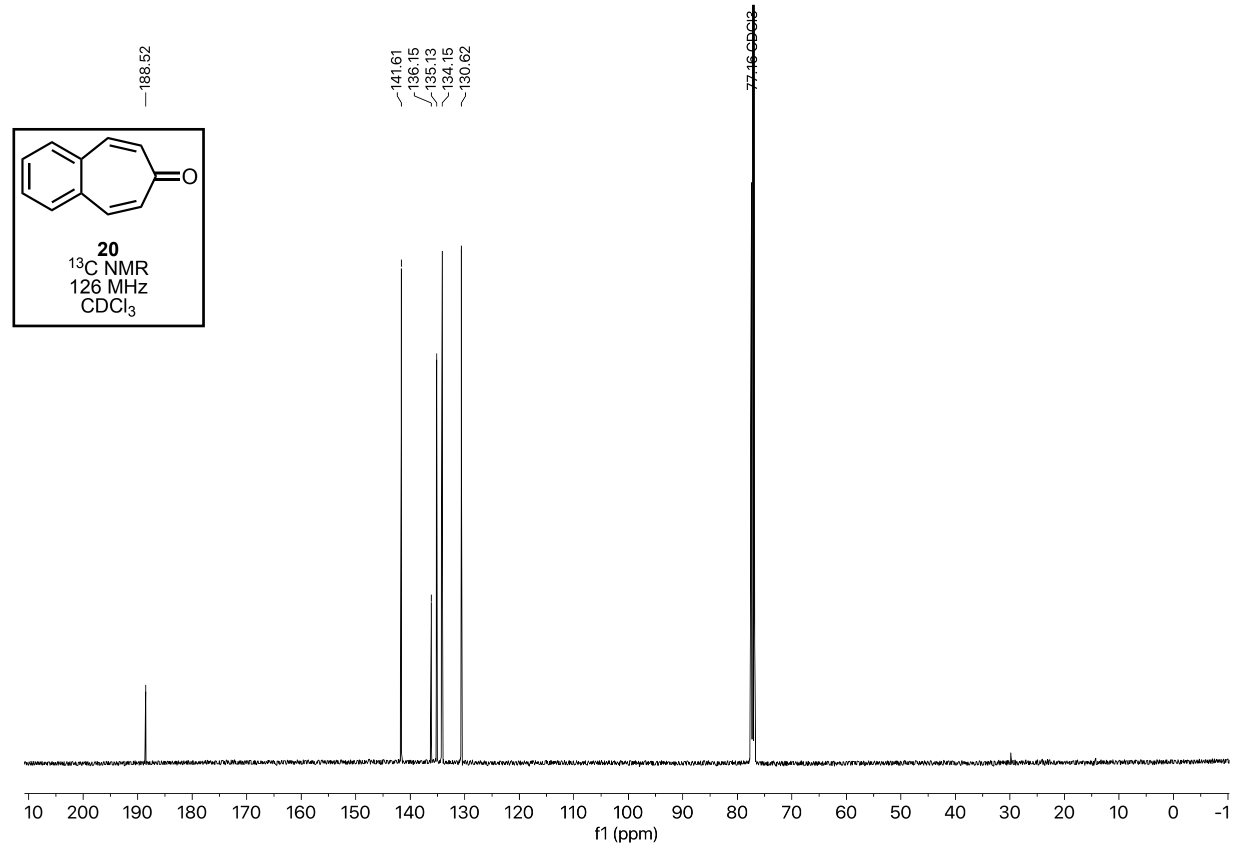
188.52
141.61
136.15
135.13
134.15
130.62
77.16 CDCl3
20
$^{13}$C NMR
126 MHz
$CDCl_3$
10 200 190 180 170 160 150 140 130 120 110 100 90 80 70 60 50 40 30 20 10 0 -1
f1 (ppm)

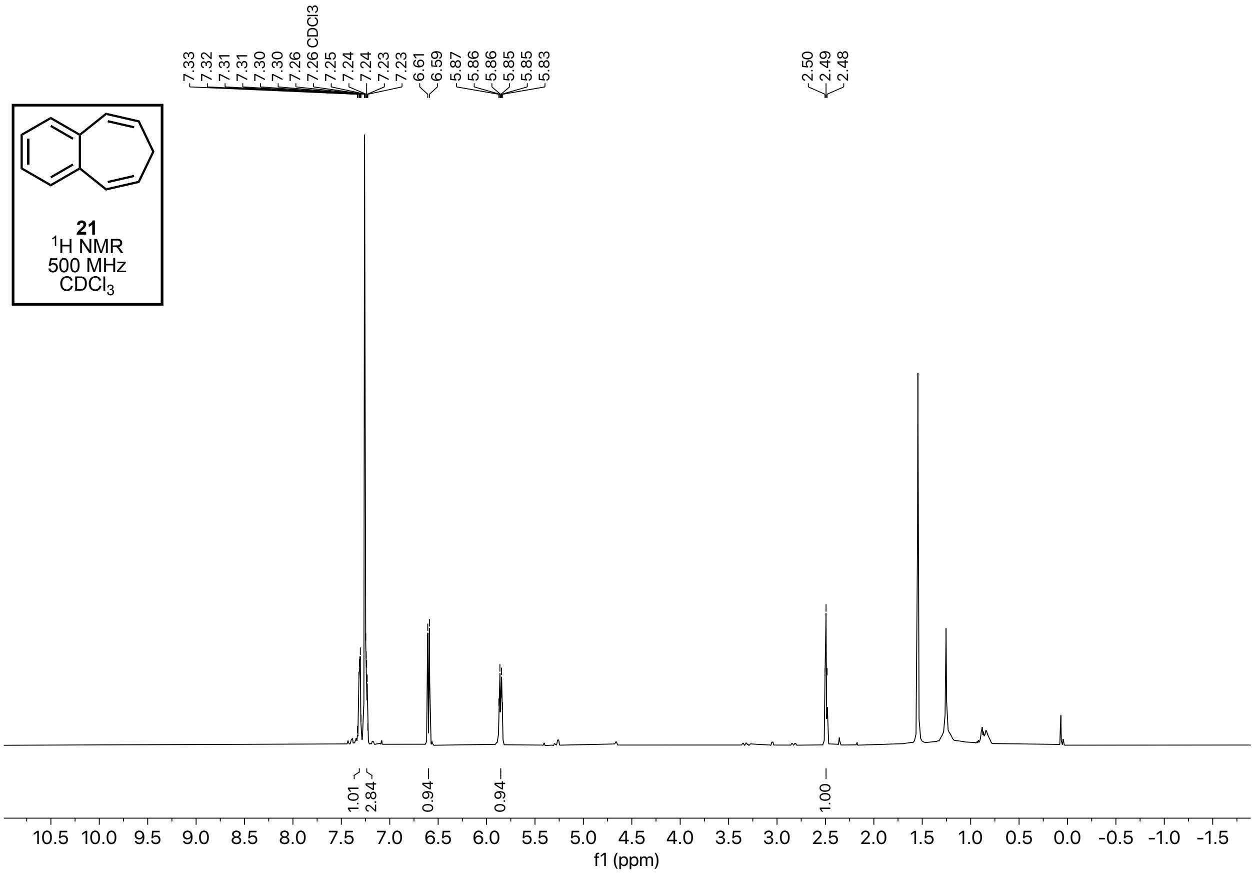
7.33
7.32
7.31
7.31
7.30
7.30
7.26 CDCl3
7.25
7.24
7.24
7.23
7.23
6.61
6.59
5.87
5.86
5.86
5.85
5.85
5.83
2.50
2.49
2.48
21
1H NMR
500 MHz
CDCl3
1.01
2.84
0.94
0.94
1.00
f1 (ppm)

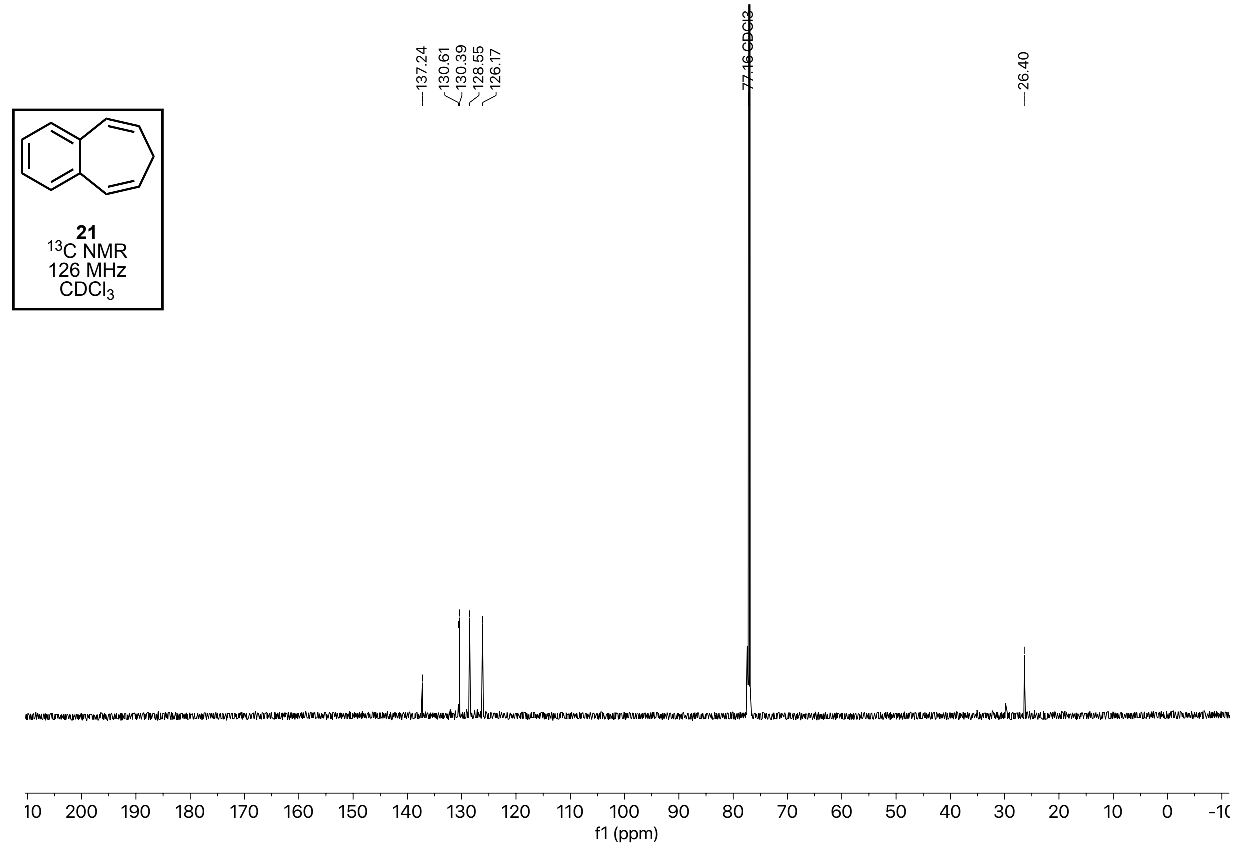
137.24
130.61
130.39
128.55
126.17
77.16 CDCl3
26.40
21
13C NMR
126 MHz
CDCl3
f1 (ppm)

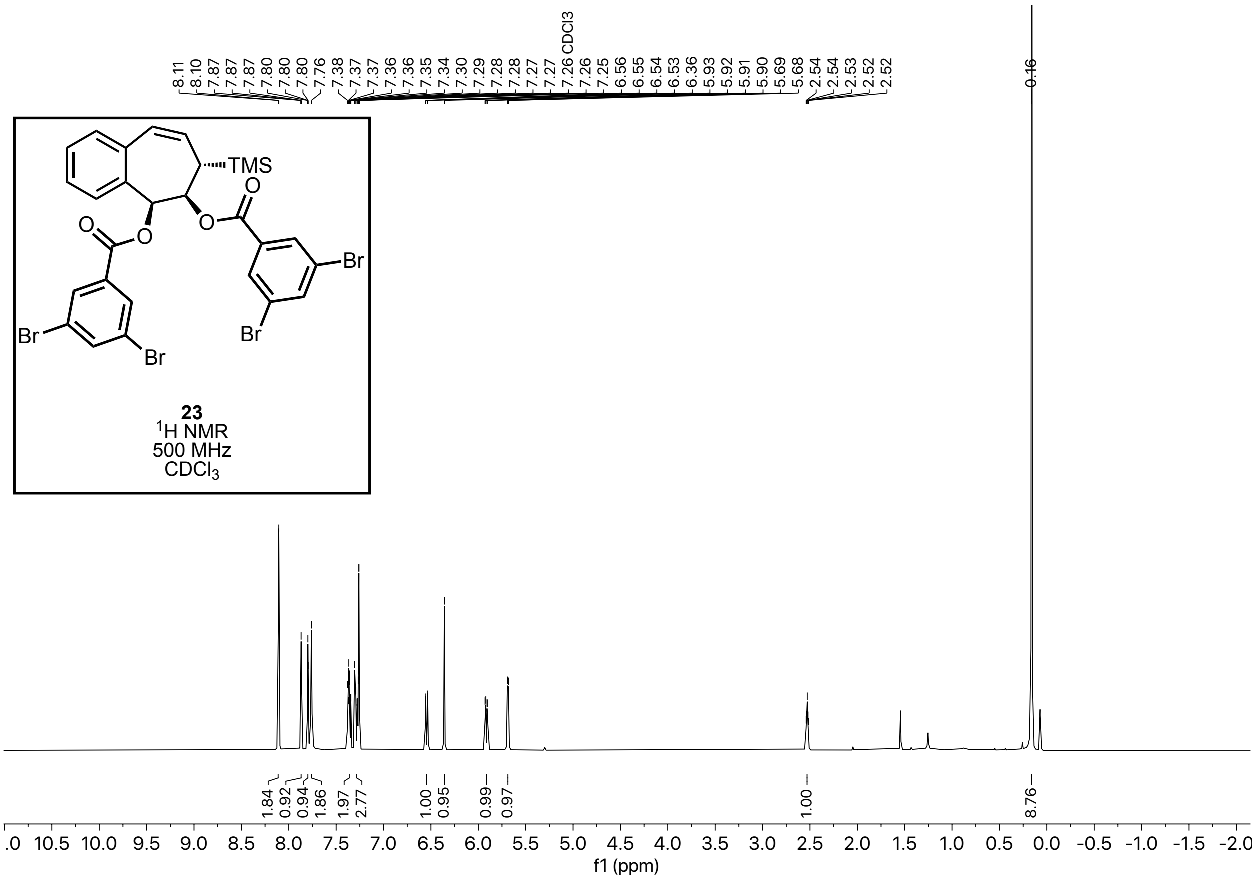
8.11
8.10
7.87
7.87
7.87
7.80
7.80
7.80
7.76
7.38
7.37
7.37
7.36
7.36
7.35
7.34
7.30
7.29
7.28
7.28
7.27
7.27
7.26 CDCl3
7.26
7.25
6.56
6.55
6.54
6.53
6.36
5.93
5.92
5.91
5.90
5.69
5.68
2.54
2.54
2.53
2.52
2.52
0.16
TMS
O
O
O
O
Br
Br
Br
Br
23
$^1$H NMR
500 MHz
$CDCl_3$
1.84
0.92
0.94
1.86
1.97
2.77
1.00
0.95
0.99
0.97
1.00
8.76
f1 (ppm)

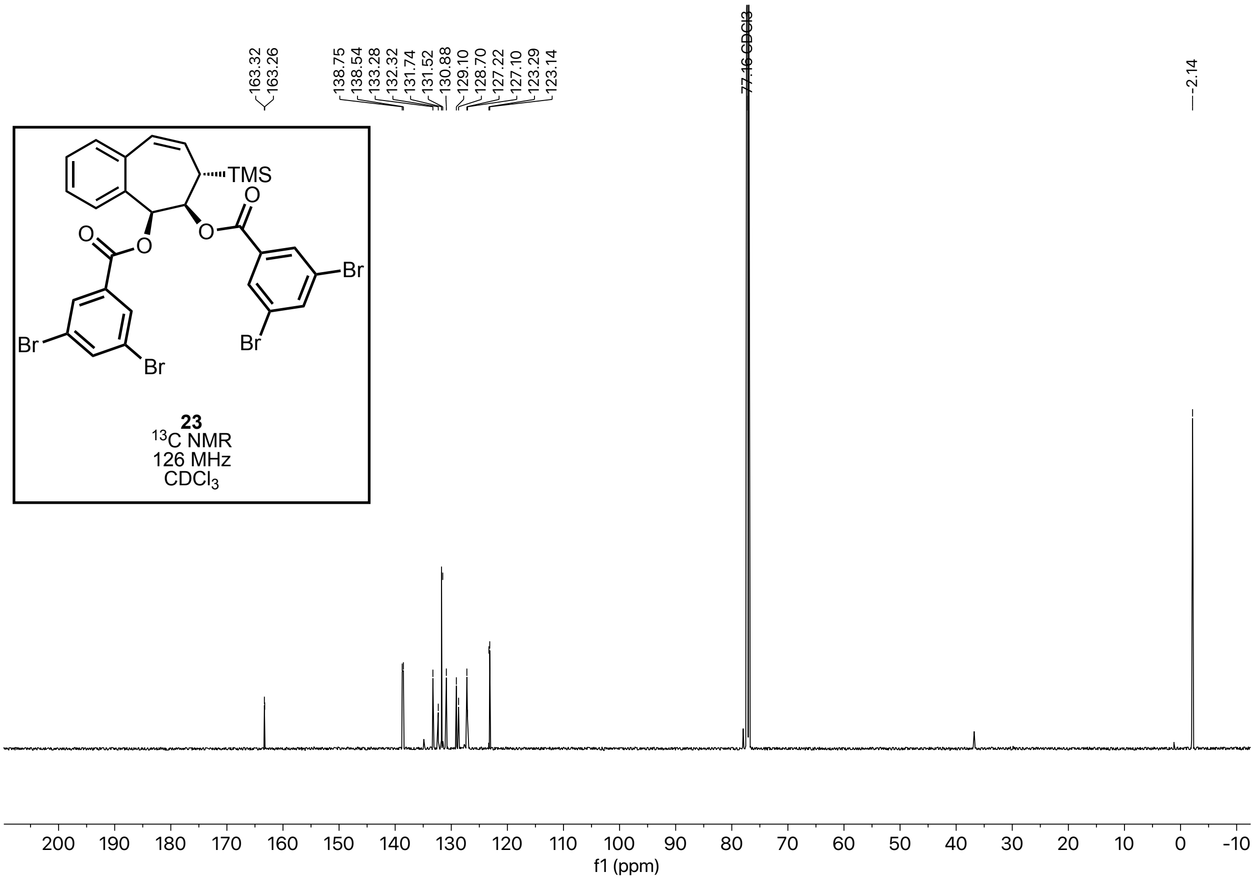
163.32
163.26
138.75
138.54
133.28
132.32
131.74
131.52
130.88
129.10
128.70
127.22
127.10
123.29
123.14
77.16 CDCl3
-2.14
TMS
O
O
O
O
Br
Br
Br
Br
23
$^{13}$C NMR
126 MHz
$CDCl_3$
f1 (ppm)
